# Enantioselective Total Synthesis of (+)‐Garsubellin A

**DOI:** 10.1002/anie.202109193

**Published:** 2021-09-09

**Authors:** Dongseok Jang, Minchul Choi, Jinglong Chen, Chulbom Lee

**Affiliations:** ^1^ Department of Chemistry Seoul National University Seoul 08826 Republic of Korea; ^2^ Department of Chemistry Princeton University Princeton New Jersey 08540 USA; ^3^ Current address: College of Materials Science and Engineering Fuzhou University Fuzhou 350108 China

**Keywords:** carbocycles, carbonylation, natural products, PPAP, total synthesis

## Abstract

Garsubellin A is a meroterpene capable of enhancing the enzyme choline acetyltransferase whose decreased level is believed to play a central role in the symptoms of Alzheimer's disease. Due to the potentially useful biological activity together with the novel bridged and fused cyclic molecular architecture, garsubellin A has garnered substantial synthetic interest, but its absolute stereostructure has been undetermined. We report here the first enantioselective total synthesis of (+)‐garsubellin A. Our synthesis relies on stereoselective fashioning of a cyclohexanone framework and double conjugate addition of 1,2‐ethanedithiol that promotes aldol cyclization to build the bicyclic [3.3.1] skeleton. The twelve‐step, protecting group‐free synthetic route has enabled the syntheses of both the natural (−)‐garsubellin A and its unnatural (+)‐antipode for biological evaluations.

## Graphical abstract

The first enantioselective total synthesis of garsubellin A establishes the absolute stereostructure of the plant‐derived meroterpene that enhances the choline acetyltransferase enzyme responsible for the biosynthesis of the neurotransmitter acetylcholine. The 12‐step, protecting group‐free synthesis features use of a dithiolane for efficient construction of the bridged and fused ring system.

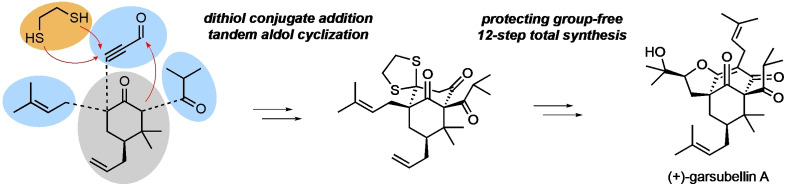

Garsubellin A (**1**) is a polycyclic polyprenylated acylphloroglucinol (PPAP) isolated from the wood of *Garcinia subelliptica*.^[1]^ It is a potent inducer of choline acetyltransferase (ChAT), the enzyme responsible for the biosynthesis of the neurotransmitter acetylcholine (ACh). As neurodegenerative pathologies are associated with an atrophy of cholinergic neurons and attenuation in ACh levels, this meroterpenoid could, in principle, function as a nonpeptidyl neuromodulatory agent for the treatment of Alzheimer's disease.^[2]^ Structurally, **1** features a highly congested [3.3.1] bicyclic skeleton, in which both of the bridgehead positions are quaternary stereocenters. This core motif, characteristically conserved among the natural products of the PPAP family,^[3]^ is further adorned with a fused tetrahydrofuran ring, posing formidable challenges to synthetic undertaking. Not surprisingly, the novel molecular architecture along with the potentially useful biological activity has rendered garsubellin A an attractive target of chemical synthesis investigations.^[4]^ Thus far, the groups of Shibasaki, Danishefsky, Nakada, and Maimone have accomplished total syntheses, each providing an ingenious synthetic road map to **1**.^[5]^ These feats, however, have been performed in racemic settings, and the absolute stereostructure of **1** remains to be established. In this regard, it is well to note that the C9 carbonyl bridge and the C7 prenyl or geranyl chain may be configured to be of both α‐ and β‐orientations in the PPAP biosynthesis (**2** to **1** in Scheme 1).^[6]^ Therefore, it is difficult to infer the absolute stereochemistry of any PPAP despite the high degree of structural homology existing within the family,^[7]^ as exemplified by the elegant chemical synthesis investigations on clusianone and nemorosone which have revealed the absolute configurational sense of these compounds to be antipodal to that of hyperforin.^[8]^ Reported here is the first enantioselective total synthesis of garsubellin A (**1**) that enabled determination of its absolute stereochemistry.

**Scheme 1:**
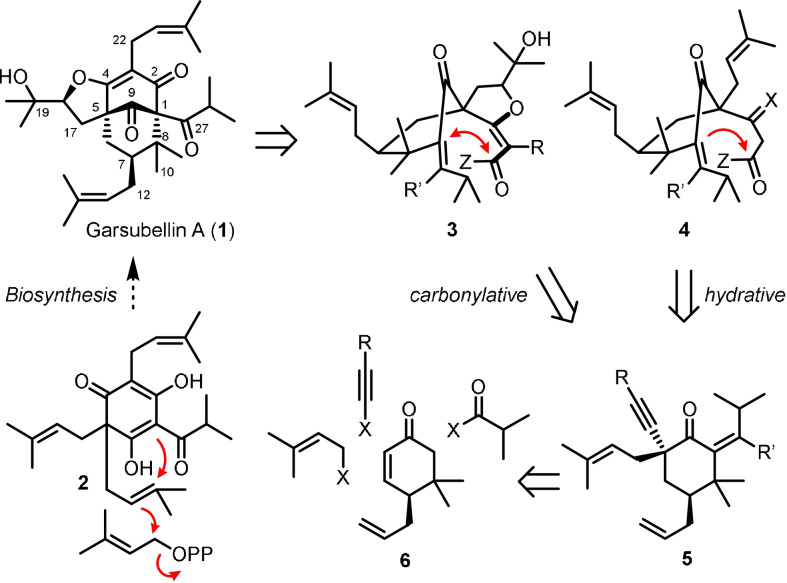
Structure and retrosynthesis of Garsubellin A.

The major synthetic challenge of **1** resides in the C1 region where a quaternary stereocenter is placed at the bridgehead position, surrounded by three ketones and an additional quaternary center of the *gem*‐dimethylated C8. Our strategy evolved from the notion that the C1−C2 connection constituting a bridgehead stereocenter might be forged via bicyclic ring closure,^[9]^ also establishing the novel confluence of the three carbonyls (Scheme 1). Hence, the challenge of constructing the decorated [3.3.1] system could be reduced to positioning suitable C1 and C2 units in close proximity for bond formation, which would resolve most of the synthetic issues in the northern half of **1**. We envisioned that such a scenario could be implemented with a carbonyl intermediate of *seco*‐tricycle **3** or *seco*‐bicycle **4**, arising each from an intra‐ or intermolecular alkyne fashioning process. The requisite alkyne **5** was then expected to be assembled by stereocontrolled incorporation of isobutylidene, prenyl, and alkynyl units into cyclohexenone **6**, which in turn could be prepared in an enantiomeric form from readily available building blocks.

Our synthetic studies were first focused on enantio‐defined preparation of cyclohexenone **6** which would serve as an initial staging post (Scheme 2). Starting with the known enol ether **7**, prepared from dimedone and L‐menthol,^[10]^ the route employed the Stork–Danheiser protocol for the allylation and reductive ketone transposition.^[11]^ While the allylation of **7** furnished **8** as a 1:1 diastereomeric mixture, the β‐allylated enone **8 b** could be accessed in reliable yield (>70 %, *dr*>100:1) on multi‐decagram scales after a few rounds of recycling of **8 a** through base promoted epimerization. The configuration of the allyl‐attached C7 center was established by X‐ray crystallographic analysis of iodide **8 c**,^[12]^ which correlated with **8 b** upon reductive deiodination.

**Scheme 2:**
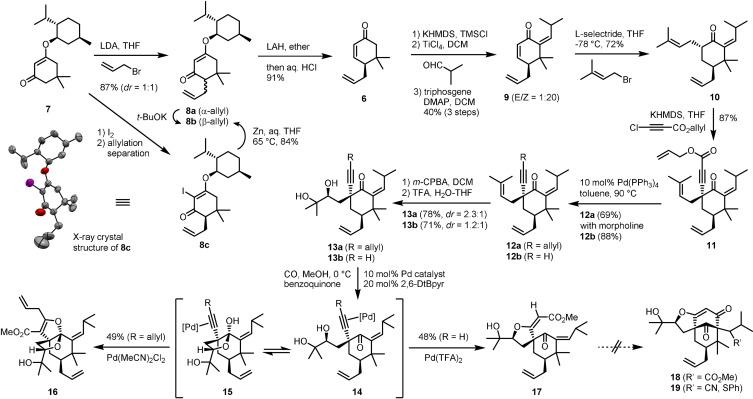
Alkoxycarbonylation approach to Garsubellin A. LDA=lithium diisopropylamide, LAH=lithium aluminum hydride, HMDS=hexamethyldisilazide, TMSCl=trimethylsilyl chloride, DMAP=4‐dimethylaminopyridine, L‐selectride=lithium tri‐*sec*‐butylborohydride, *m*‐CPBA=3‐chloroperbenzoic acid, TFA=trifluoroacetic acid, 2,6‐DtBpyr=2,6‐di‐*tert*‐butylpyridine.

Having procured **6** in high enantiopurity, we turned to the synthesis of the proposed key intermediate **5** (cf. **12**) and evaluation of the tandem cyclization via intermediate **3** (Z=Pd). Thus, enone **6** was subjected to a series of reactions that introduced isobutylidene, prenyl and alkynyl groups to the cyclohexanone framework. The Mukaiyama aldol reaction of **6** with isobutyraldehyde followed by elimination of the aldol adduct produced dienone **9** with high (*Z*)‐selectivity.[^13^, ^14^] The installation of a prenyl group was then carried out through the conjugate reduction‐enolate trapping process employing L‐selectride and prenyl bromide,^[15]^ which took place only at the endocyclic alkene with complete stereoselectivity to afford **10** as a single isomer. The strong α‐facial preference of the enolate was also manifested in the subsequent alkynylation,^[16]^ thus establishing the C5 quaternary stereogenic center that would become a bridgehead position. After decarboxylation of **11** under palladium catalysis to effect allylation (cf. **12 a**)^[17]^ as well as removal of the Alloc group (cf. **12 b**),^[18]^ oxidation of the prenyl chain with *m*‐CPBA and hydrolysis of the resulting epoxide furnished, albeit with low diastereoselectivity, diols **13 a** and **13 b** poised for alkoxycarbonylation.

With the requisite diols **13** in hand, the cascade carbonylative cyclization was probed for its potential to construct the bridged ring system. Subjection of the internal alkyne **13 a** to the Pd^II^‐catalyzed conditions for alkoxycarbonylation,^[19]^ however, did not give rise to the desired [3.3.1] skeleton but led instead to the formation of tricyclic ketal **16** via 5‐*endo* cyclization of hemi ketal **15** which might exist in equilibrium with ketone **14**.^[20]^ While the intended 5‐*exo* cyclization was feasible with the terminal alkyne substrate **13 b**, the reaction induced only monocyclization to give the spirocyclic methyl ester **17** without generating the desired carbonyl bridged ring system **18**. A series of attempts were further made to transform **17** into **19** making use of exogenous cyanide and thiolate nucleophiles. Unfortunately, the projected tandem Michael–Dieckmann approach, notwithstanding considerable experimentation, proved none too promising, as the *exo*‐alkene of **17** was recalcitrant toward conjugate addition while the methyl enoate was prone to facile *E* to *Z* isomerization.^[21]^

In light of the difficulty encountered in the oxycarbonylation approach based on a migratory insertion event of metal acyl **3**, we sought to explore an alternative strategy using carbonyl addition of **4** (Z=H). In pursuing this line of thought, it was anticipated that the formation of the critical C1−C2 bond would be facilitated by engaging a C2 carbonyl, devoid of the conjugation and spirocyclic scaffold, with an activated C1 alkene (formally R′=OH in **4**). Hence, our revised synthetic campaign commenced with the sequence comprising isobutyrylation of **6**, reductive prenylation of **20** and alkynylation of **21** that uneventfully produced diketone **22 a** (Scheme 3). Similar to the previous route to **11**, all of these C−C bond‐forming events occurred with exclusive diastereoselectivity owing again to the α‐facial preference of the enolate presumably exerted by the C7 β‐allyl chain in concert with the C8 *gem*‐dimethyl group.^[22]^ Notably, the alkynylation of **21** using *t*‐BuOK proceeded smoothly, despite the presence of two more potential reaction sites at C1 and C28, to place the alkynoate group only at C5. In contrast, the use of LiTMP as a base for the same reaction brought about exclusive alkynylation at C28 (Table S4 in the Supporting Information). Noteworthy in these alkynylation reactions was that neither condition affected the C1 methine flanked by two ketones,^[23]^ although **22 a** having an α‐isobutyryl group was found to isomerize easily to the more stable β‐epimer **22 b** upon warming prior to workup. As observed in the reductive prenylation of **20** using L‐selectride that gave **21** without implicating carbonyl reduction, treatment of **22 b** with Dibal‐H at −78 °C led to clean reduction of the ethyl ester to produce aldehyde **23**, leaving the two ketones intact.^[24]^

**Scheme 3:**
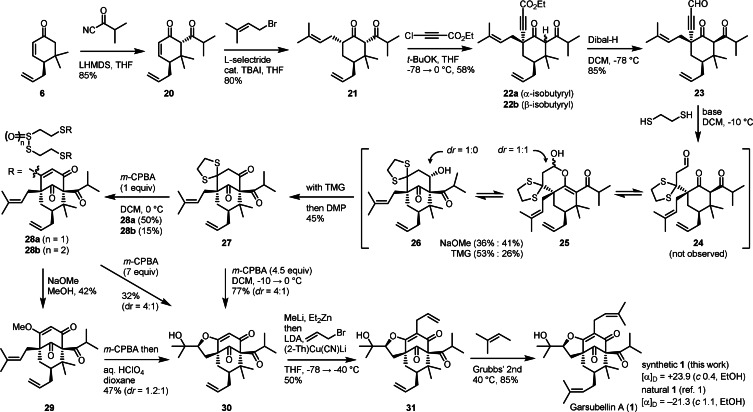
Enantioselective total synthesis of (+)‐Garsubellin A. TBAI=tetrabutylammonium iodide, Dibal‐H=diisobutylaluminum hydride, TMG=1,1,3,3‐tetramethylguanidine, DMP=Dess–Martin periodinane, Grubbs’ 2nd=Grubbs second‐generation catalyst.

Having established a robust and efficient route to **23**, wherein was the C2 unit in place as an aldehyde, we set out to examine the alkyne addition process that would impart a C4 functionality suitable for the construction of the THF ring while disposing the three‐carbon aldehyde chain amenable for aldol cyclization. Since a β‐ketoaldehyde which would derive from hydration of the ynal was deemed unapt for ring closure due to enolization, we resorted to dithioketal formation to effect formal hydration.^[25]^ Pleasingly, the conjugate addition of 1,2‐ethanedithiol to **23** under Ley's condition (NaOMe, MeOH‐CH_2_Cl_2_, −10 °C)^[25c]^ occurred smoothly to give rise directly to a mixture of lactol **25** (*dr*=1:1) and alcohol **26** (*dr*=1:0), rather than aldehyde **24**. The remarkable facility with which the aldol cyclization took place appeared to be the consequence of the steric buttressing effect of the 1,3‐dithiolane juxtaposed with the bridgehead quaternary center.^[26]^ While exposure of the purified **25** to a catalytic amount of a base resulted in redistribution into a 1.2:1 mixture of **25** and **26**, interestingly, this presumable thermodynamic ratio could be reoriented to 1:3 favoring aldol **26** in the presence of a stoichiometric amine base (Table S7 in the Supporting Information). From a set of screening experiments, TMG was identified to be an effective base that optimally promoted both the double conjugate addition and aldol cyclization to give **26**, which could be in situ oxidized with DMP to triketone **27**.

In advancing the [3.3.1] bicyclic intermediate **27** to the very end of the synthetic campaign, we first directed our efforts toward oxidation of the prenyl chain. Given the diastereoselectivity noted in the formation of **13** in favor, albeit slight (*dr*=1.2–2.3:1), of the desired C18 β‐epimer, we anticipated that this stereopreference might be enhanced by the presence of the 1,3‐dithiolane segment. Treatment of **27** with *m*‐CPBA, however, brought about rapid oxidation at a sulfur center, leading to the formation of a mixture of the dimeric thiosulfinate **28 a** and thiosulfonate **28 b** probably via disproportionation reactions of a ring‐opened sulfenic acid intermediate (Scheme S5 in the Supporting Information). Resubjection of **28 a** to the reaction with excess *m*‐CPBA indeed induced oxidation at the prenyl group to give, following workup, the THF‐fused enone **30** as the major product.^[27]^ The beneficial effect of the sulfur substituent, in terms of the stereoselectivity of the epoxidation and the facility of the subsequent THF ring formation, was evident when compared with the result of the corresponding reaction of methyl ether **29** which gave **30** with a lower diastereoselectivity following treatment with a strong acid. In the event, epoxidation and concomitant removal of the dithiolane ring could be accomplished by exposing **27** to 4.5 equivalents of *m*‐CPBA at −10 °C to produce **30** in 77 % yield with 4:1 diastereoselectivity. The final stage of the total synthesis involved installation of the C3 and C7 prenyl groups, which was achieved in two steps. As the direct alkenyl C−H allylation of **30** proved challenging without protection of the C19 alcohol, an in situ protocol was developed in which the copper mediated allylation was conducted,^[28]^ with the C19 alcohol transiently masked as an alkoxyzincate,^[29]^ to yield the penultimate bis‐allylated intermediate **31**. Finally, ruthenium‐catalyzed cross‐metathesis with 2‐methyl‐2‐butene delivered **1**,^[30]^ whose (+)‐sign of optical rotation revealed our synthetic compound to be the enantiomer of the natural garsubellin A. This result shows that the absolute stereostructure of the natural (−)‐garsubellin A is in line with those of (+)‐clusianone and (+)‐nemorosone, in which the C9 carbonyl bridge and C7 prenyl chain are both α‐oriented.

We have reported the first enantioselective total synthesis of garsubellin A (**1**). Our synthesis features high stereocontrol in fashioning a dimedone‐derived cyclohexane in the early phase and the late‐stage construction of the bicyclic core. Whereas the cascade oxycarbonylation approach was unsuccessful, the strategy based on the double conjugate addition of 1,2‐ethanedithiol proved effective to build the bicyclo[3.3.1]nonane framework. Notably, the 1,3‐dithiolane installed by the conjugate addition served to facilitate aldol cyclization, stereoselective epoxidation and THF ring fusion. Also noteworthy in this twelve‐step, protecting group‐free synthetic route is that the single stereocenter established at C7 in the initial stage controlled the configurations of the rest of the stereogenic centers. We have also completed the total synthesis of the natural (−)‐garsubellin A (Scheme S7 in the Supporting Information). Studies are underway to evaluate the biological performance of the unnatural antipode with an aim to identify a therapeutically relevant target and mode of action. The results will be reported in due course.

## Conflict of interest

The authors declare no conflict of interest.

## Supporting information

As a service to our authors and readers, this journal provides supporting information supplied by the authors. Such materials are peer reviewed and may be re‐organized for online delivery, but are not copy‐edited or typeset. Technical support issues arising from supporting information (other than missing files) should be addressed to the authors.

Supporting Information
