# Supplementary material for: Enantioselective Total Synthesis of (+)‐Garsubellin A
Source: Angew Chem Int Ed Engl. 2021 Sep 9;60(42):22735–9. doi: 10.1002/anie.202109193 (PMC8519110; doi:10.1002/anie.202109193)
Supplement: Supplementary file 1 — Supporting Information [file ANIE-60-22735-s001.pdf]

Angewandte Chemie
Eine Zeitschrift der Gesellschaft Deutscher Chemiker

Supporting Information

Enantioselective Total Synthesis of (+)-Garsubellin A

*Dongseok Jang, Minchul Choi, Jinglong Chen, and Chulbom Lee**

anie_202109193_sm_miscellaneous_information.pdf

Contents

1. General information……….…………………………………….......……….…….………….….….....…...S2

2. Overview of the synthetic pathways……………..…………………………………………………..……S3

3. Experimental procedures and compound characterization……………………………….……...….S5

3.1. Synthetic procedures for the enantio-defined preparation of the allylated cyclohexenone **6..........…………..….S5**

3.2. Synthetic procedures in the palladium-catalyzed alkoxycarbonylation approach**……………...…………….….S11**

3.3. Synthetic procedures in the cascade dithiol addition/cyclization approach to (+)-garsubellin A **……………S25**

3.4. Alternative approach toward tricyclic ketone **30……………….…………………………………..…S49**

3.5. Experimental results for the synthesis of (–)-garsubellin A and comparison of optical rotation values**……....S52**

4. Comparison with the reported spectral data of garsubellin A……………………………………….…S53

5. ^{1}H and ^{13}C NMR spectra for synthesized compounds…………………………………………………….S55

6. X-ray crystallographic information…………………………………….………………………………...S125

7. References………………………………………………………………………………………………..…S135

1. General Information

NMR spectra were obtained on Bruker DPX-300 (300 MHz), Agilent 400-MR DD2 Magnetic Resonance System (400 MHz) and Varian/Oxford As-500 (500 MHz) spectrophotometers. Chemical shift values were recorded as parts per million (δ ppm) relatives to tetramethylsilane as an internal standard unless otherwise indicated and coupling constants in Hertz (Hz). The following abbreviations (or combinations thereof) were used to explain the multiplicities: s = singlet, d = doublet, t = triplet, q = quartet, m = multiplet, br = broad. IR spectra were measured on a Thermo Scientific Nicolet 6700 spectrometer. High resolution mass spectra were recorded on a JEOL JMS-600W or a JEOL JMS-700 spectrometer using an electron impact (EI) or chemical ionization (CI) method. Optical Rotations were measured in a 50.00 mm cell with a Jasco P-1030 polarimeter equipped with a sodium lamp (589 nm).

The progress of reaction was checked on thin layer chromatography (TLC) plates (Merck 5554 Kiesel gel 60 F254), and the spots were visualized under 254 nm UV light and/or charring after dipping the TLC plate into a vanillin solution (15.0 g of vanillin and 2.5 mL of concentrated sulfuric acid in 250 mL of ethanol), a $KMnO_4$ solution (3.0 g of $KMnO_4$, 20.0 g of K_2CO_3, and 5.0 mL of 5% NaOH solution in 300 mL of water), or a phosphomolybdic acid solution (250 mg phosphomolybdic acid in 50 mL ethanol). Column chromatography was performed on silica gel (Merck 9385 Kiesel gel 60) using hexanes-EtOAc (v/v). All solvents were obtained by passing through activated alumina columns of solvent purification systems from Glass Contour.

2. Overviews of the synthetic pathways

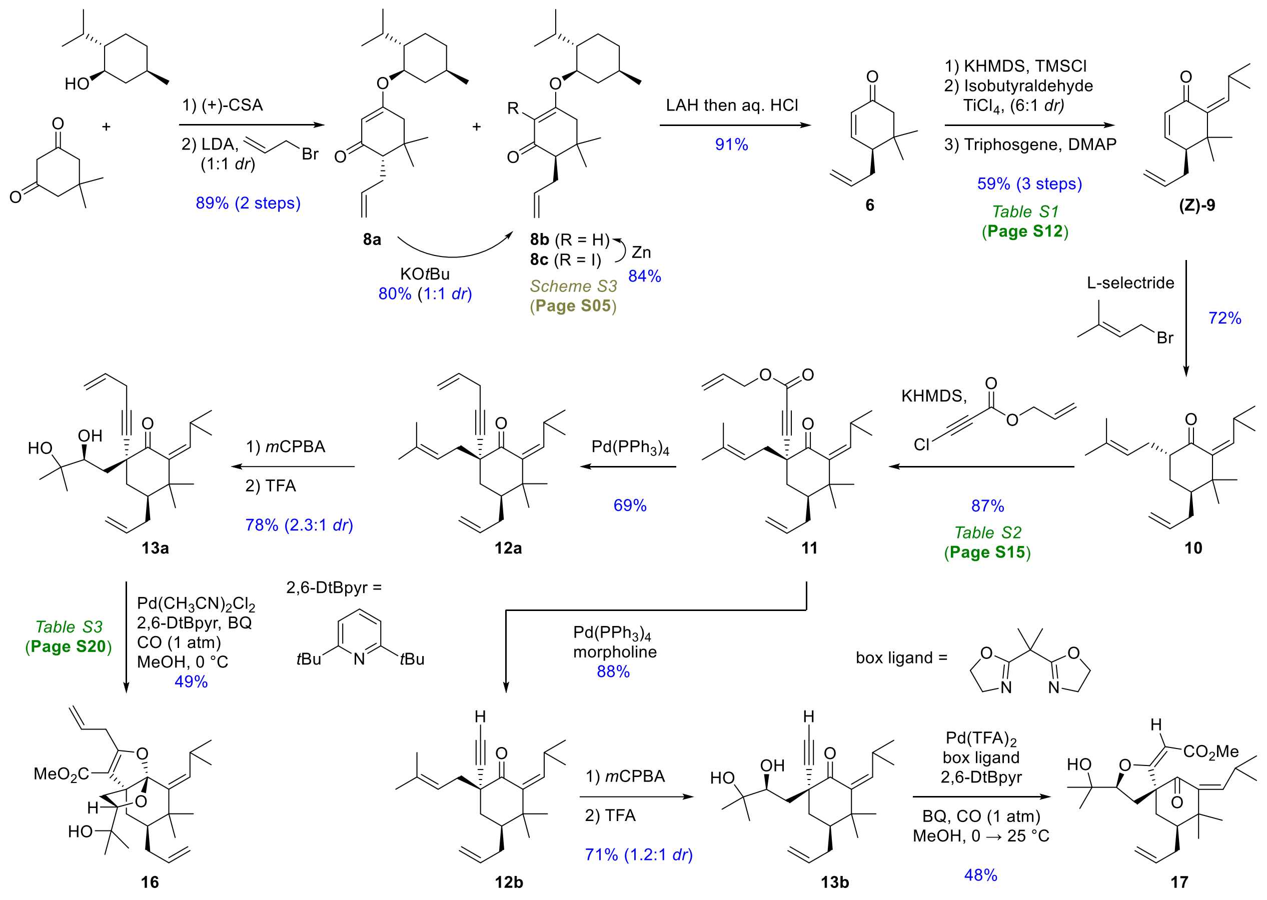

Scheme S1. Palladium-catalyzed oxycarbonylation approach

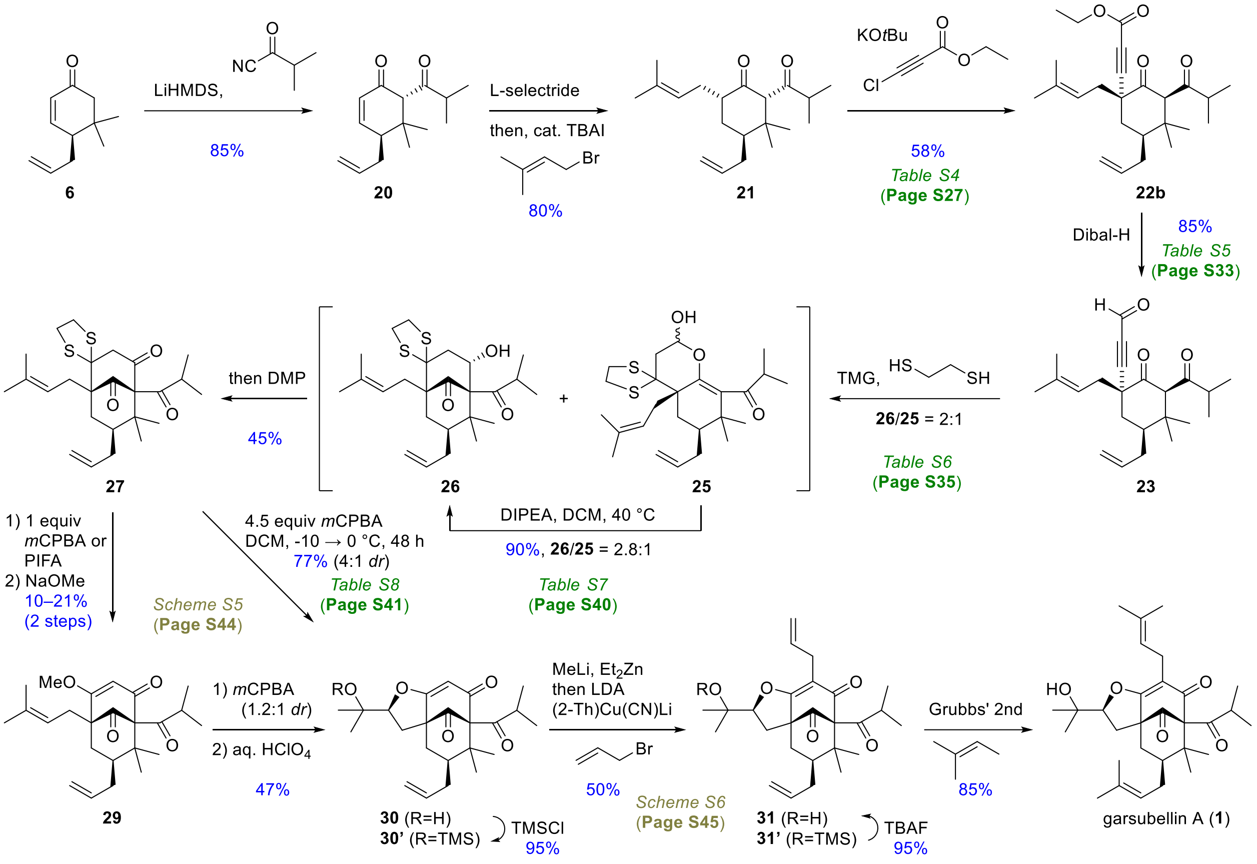

Scheme S2. Dithiol conjugate addition-aldol cyclization approach

3. Experimental procedures and compound characterization

3.1. Synthetic procedures for the enantio-defined preparation of the allylated cyclohexenone **6**

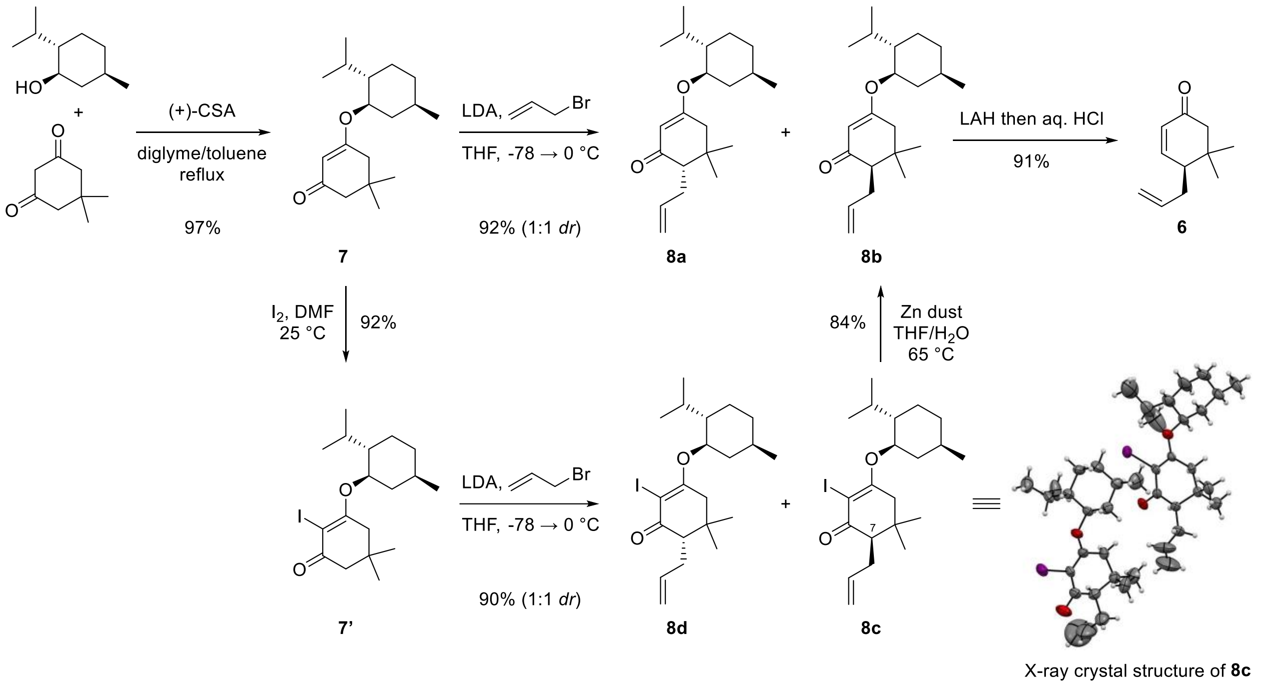

Scheme S3. Synthetic route to enone **6** and determination of the absolute stereochemistry at C7

Synthesis of enol ether 7 from dimedone and (L)-menthol

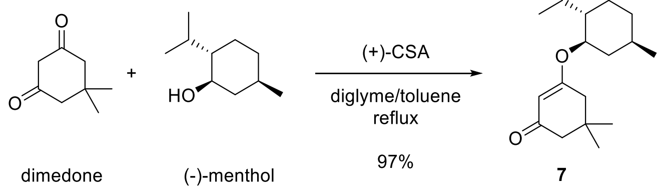

The enantiomer of enol ether **7** is a known compound reported in the literature.[1] A 1-L, one-necked, round bottomed flask was fitted with a Dean–Stark trap equipped with a reflux condenser and nitrogen inlet. The flask was then charged with 5,5-dimethyl-1,3-cyclohexanedione (dimedone, 11.65 g, 79 mmol, 1.0 equiv), (–)-menthol (14.8 g, 95 mmol, 1.2 equiv), 39.5 mL of diglyme, 168 mL of toluene, and (+)-camphorsulfonic acid (CSA, 1.84 g, 7.9 mmol, 0.1 equiv). The resulting suspension was heated to a rapid reflux, which, within 30 min, became a homogeneous, pale yellow solution. After 24 h, the resulting solution was allowed to cool to 25 °C and concentrated in vacuum to remove solvent. Then the residue was diluted with 300 mL of saturated $NaHCO_3$ solution. After extraction with two 300 mL portions of ether, the combined organic layers were dried over $MgSO_4$, filtered and concentrated. The crude oil was purified by flash column chromatography (silica gel, hexanes/EtOAc = 5/1 → 3/1) to give enol ether **7** (21.5 g, 77 mmol, 97% yield) as a pale-yellow solid.

R$_f$ 0.53 (hexanes/EtOAc = 2:1, PMA)

^{1}H NMR (500 MHz, $CDCl_3$) δ 5.40 (s, 1H), 3.98 (td, *J* = 10.7, 4.1 Hz, 1H), 2.25 (d, *J* = 3.5 Hz, 2H), 2.22 (s, 2H), 2.14 – 2.08 (m, 1H), 1.96 (dtd, *J* = 14.0, 7.0, 2.9 Hz, 1H), 1.73 – 1.66 (m, 2H), 1.51 – 1.44 (m, 1H), 1.43 – 1.36 (m, 1H), 1.07 (s, 3H), 1.07 (s, 3H), 1.05 – 1.01 (m, 1H), 0.99 – 0.92 (m, 2H), 0.91 (d, *J* = 6.6 Hz, 3H), 0.89 (d, *J* = 6.0 Hz, 3H), 0.74 (d, *J* = 7.0 Hz, 3H).

^{13}C NMR (126 MHz, $CDCl_3$) δ 200.0, 175.9, 101.5, 78.5, 50.9, 47.5, 43.6, 39.5, 34.5, 32.7, 31.5, 28.5, 28.4, 26.8, 24.0, 22.2, 20.7, 16.9.

FTIR (neat, cm^{-1}) 3068, 2955, 2868, 1659, 1604, 1454, 1372, 1214.

HRMS (ESI) *m/z* calcd. for $C_{18}H_{30}O_2Na^+$ $[M+Na]^+$: 301.2138, found: 301.2138.

$[\alpha]_D^{25}$ −114.2 (*c* 1.0, EtOH).

Allylation of 7 to form enol ethers 8a and 8b

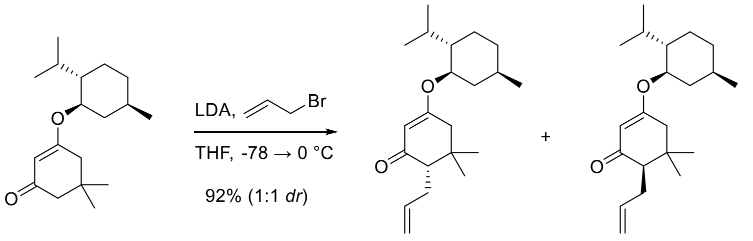

7 **8a** **8b**

To a solution of freshly prepared lithium diisopropylamide (21.6 mmol, 1.2 equiv) in THF (60 mL) was added a solution of enol ether **7** (5.02 g, 18.0 mmol) in THF (10 mL) at −78 °C slowly over a period of 1 h, and the resulting yellow solution was continued to stir at −78 °C for 1 h. Allyl bromide (3.1 mL, 36.0 mmol, 2.0 equiv) was then added and the reaction mixture was warmed to 0 °C. After being stirred at 0 °C for 2 h, the reaction mixture was poured into a biphasic mixture of saturated aq. NH_4Cl (100 mL) and EtOAc (150 mL). The layers were separated and extracted twice with EtOAc (100 mL). The combined organic extracts were dried over anhydrous $MgSO_4$ and concentrated in vacuo. Purification of the residue by flash column chromatography (silica gel, hexanes/EtOAc = 10/1 → 4/1) afforded **8a** and **8b** (5.27 g, 16.6 mmol, 92% yield, 1:1 *dr*) as a colorless liquid. The diastereomers were separated by conducting flash column chromatography (silica gel, petroleum ether/diethyl ether = 40/1 → 10/1) several times.

8b (β-allyl)

R$_f$ 0.45 (hexanes/EtOAc = 3:1, vanillin)

^{1}H NMR (500 MHz, $CDCl_3$) δ 5.89 (ddt, *J* = 17.1, 10.1, 7.0 Hz, 1H), 5.34 (s, 1H), 5.04 – 4.93 (m, 2H), 2.35 – 2.28 (m, 3H), 2.19 (d, *J* = 17.6 Hz, 1H), 2.13 – 2.07 (m, 2H), 1.95 (dtd, *J* = 14.0, 7.0, 2.9 Hz, 1H), 1.69 (ddq, *J* = 13.1, 6.6, 3.2 Hz, 2H), 1.42 (dddt, *J* = 18.6, 16.4, 9.9, 3.3 Hz, 2H), 1.09 (s, 3H), 1.09 – 1.05 (m, 1H), 1.05 – 1.00 (m, 1H), 0.99 (s, 3H), 0.99 – 0.92 (m, 2H), 0.91 (d, *J* = 6.6 Hz, 3H), 0.89 (d, *J* = 7.1 Hz, 3H), 0.74 (d, *J* = 7.0 Hz, 3H).

^{13}C NMR (126 MHz, $CDCl_3$) δ 201.9, 174.1, 138.0, 115.5, 100.8, 78.5, 57.2, 47.6, 42.6, 39.6, 35.3, 34.5, 31.5, 31.4, 29.1, 26.8, 24.5, 24.1, 22.2, 20.6, 16.9.

FTIR (neat, cm^{-1}) 2956, 2929, 1652, 1611, 1459, 1372, 1216, 1147, 989.

HRMS (EI) *m/z* calcd. for $C_{21}H_{34}O_2^+$ $[M]^+$: 318.2664, found: 318.2559.

$[\alpha]_D^{25}$ −116.6 (*c* 1.0, EtOH).

8a (α-allyl)

R*f* 0.44 (hexanes/EtOAc = 3:1, vanillin)

^{1}H NMR (400 MHz, $CDCl_3$) δ 5.91 (ddt, *J* = 17.0, 10.0, 6.9 Hz, 1H), 5.34 (s, 1H), 5.07 – 4.94 (m, 2H), 3.96 (td, *J* = 10.7, 4.1 Hz, 1H), 2.42 – 2.18 (m, 4H), 2.09 (dd, *J* = 8.6, 4.4 Hz, 2H), 1.95 (dtd, *J* = 14.0, 7.0, 2.8 Hz, 1H), 1.75 – 1.62 (m, 3H), 1.51 – 1.35 (m, 2H), 1.09 (s, 3H), 1.05 (dd, *J* = 12.8, 2.9 Hz, 1H), 1.00 (s, 3H), 0.97 – 0.93 (m, 1H), 0.91 (d, *J* = 6.6 Hz, 3H), 0.89 (d, *J* = 7.1 Hz, 3H), 0.74 (d, *J* = 7.0 Hz, 3H).

^{13}C NMR (126 MHz, $CDCl_3$) δ 201.6, 173.8, 137.9, 115.2, 100.6, 78.1, 56.9, 47.3, 42.5, 39.2, 35.3, 34.2, 31.2, 31.0, 28.8, 26.5, 24.2, 23.8, 21.9, 20.4, 16.7.

FTIR (neat, cm^{-1}) 2957, 2871, 1656, 1610, 1458, 1373, 1214, 1149, 989.

HRMS (ESI) *m/z* calcd. for $C_{21}H_{34}O_2Na^+$ $[M+Na]^+$: 341.2453, found: 341.2451.

$[\alpha]_D^{25}$ −144.3 (*c* 1.0, EtOH).

Epimerization of enol ether 8a (recycling to produce 8b)

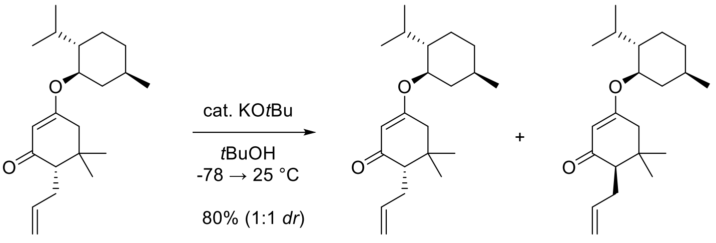

8a **8a** **8b**

To a solution of enol ether **8a** (2.1 g, 6.59 mmol) in *t*-BuOH (30 mL) was added dropwise a solution of KO*t*Bu (0.66 mL, 1.0 M in THF, 0.66 mmol, 0.1 equiv) at 25 ˚C. After being stirred at 25 °C for 12 h, the reaction mixture was poured into a biphasic mixture of saturated aq. NH_4Cl (100 mL) and EtOAc (150 mL). The layers were separated and extracted twice with EtOAc (100 mL). The combined organic extracts were dried over Na_2SO_4 and concentrated in vacuo. Purification of the residue by flash column chromatography (silica gel, hexanes/EtOAc = 10/1 → 4/1) gave **8a** and **8b** (1.68 g, 5.27 mmol, 80% yield, 1:1 *dr*) as a colorless liquid.

Reductive enone transposition of 8b to 6

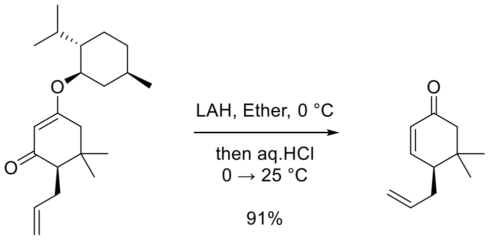

8b **6**

Enantiopure enone **6** was synthesized by the Stork–Danheiser protocol.[2,3] To a solution of enol ether **8b** (940 mg, 2.95 mmol) in 30 mL of diethyl ether was added lithium aluminum hydride (124 mg, 3.01 mmol, 1.05 equiv) at 0 ˚C. After 3 h, an aqueous solution of HCl (15 mL, 1.0 M) was added dropwise, and the resulting mixture was continued to stir

at 25 °C for 12 h and extracted with diethyl ether. The organic phase was washed with a saturated aqueous solution of $NaHCO_3$, dried over $MgSO_4$ and carefully concentrated via rotary evaporation while keeping the bath temperature at 0 °C (caution: volatile). Purification of the residue by flash column chromatography (silica gel, petroleum ether/diethyl ether = 15/1 → 3/1) afforded enone **6** (441 mg, 2.68 mmol, 91% yield) as a colorless liquid.

R*f* 0.42 (Petroleum ether/diethyl ether = 3:1, PMA)
^{1}H NMR (400 MHz, $CDCl_3$) δ 6.78 (dd, *J* = 10.2, 2.7 Hz, 1H), 6.01 (dd, *J* = 10.2, 2.5 Hz, 1H), 5.89 – 5.77 (m, 1H), 5.19 – 5.09 (m, 2H), 2.51 – 2.43 (m, 1H), 2.35 – 2.29 (m, 1H), 2.35 – 2.23 (m, 2H), 1.92 (ddt, *J* = 18.1, 15.4, 7.7 Hz, 1H), 1.11 (s, 3H), 0.91 (s, 3H).
^{13}C NMR (101 MHz, $CDCl_3$) δ 199.7, 151.5, 136.2, 128.3, 117.4, 52.4, 45.9, 37.0, 33.2, 28.8, 21.5.
FTIR (neat, cm^{-1}) 3078, 2963, 2871, 1682, 1640, 1496, 1391, 1259, 994.
HRMS (EI) *m/z* calcd. for $C_{11}H_{16}O^+$ $[M]^+$: 164.1213, found: 164.1201.
$[\alpha]_D^{25}$ +193.8 (*c* 1.0, EtOH).

Iodination of 7 to 7’

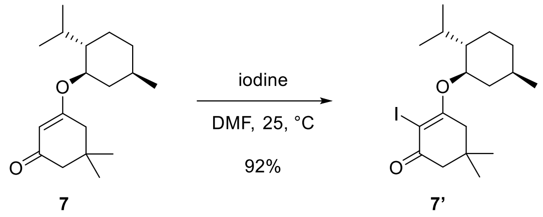

To a solution of enol ether **7** (2.12g, 7.63 mmol, 1.0 equiv) in DMF (8 mL) was added iodine (3.87 g, 15.2 mmol, 2.0 equiv) at 25 °C. The reaction flask was covered with aluminum foil and the resulting suspension was stirred at the same temperature. After 48 h, the reaction mixture was diluted with saturated aq. $Na_2S_2O_3$ (50 mL) and the resulting biphasic mixture was left to stir for 30 minutes. After extraction with diethyl ether (200 mL), the organic layer was washed with water (50 mL), dried with $MgSO_4$, filtered and concentrated under reduced pressure to afford a yellow oil. The crude product was purified by flash column chromatography (silica gel, hexanes/EtOAc = 8/1 → 5/1) to give iodo enol ether **7’** (2.84 g, 7.02 mmol, 92% yield) as a yellow solid.

R*f* 0.23 (hexanes/EtOAc = 4:1, PMA)
^{1}H NMR (500 MHz, $CDCl_3$) δ 4.17 (td, *J* = 10.6, 4.4 Hz, 1H), 2.49 (d, *J* = 4.3 Hz, 2H), 2.46 (d, *J* = 2.8 Hz, 2H), 2.09 (dtd, *J* = 14.0, 7.0, 2.7 Hz, 1H), 1.89 – 1.82 (m, 1H), 1.77 – 1.71 (m, 2H), 1.66 – 1.58 (m, 1H), 1.53 – 1.41 (m, 1H), 1.27 (ddd, *J* = 15.3, 12.5, 8.3 Hz, 2H), 1.12 (s, 6H), 1.08 – 1.02 (m, 1H), 0.95 (dd, *J* = 8.6, 4.9 Hz, 6H), 0.77 (d, *J* = 7.0 Hz, 3H).
^{13}C NMR (101 MHz, $CDCl_3$) δ 192.4, 174.5, 82.1, 79.7, 49.9, 47.6, 42.3, 41.2, 33.9, 32.9, 31.5, 28.3, 27.9, 26.1, 23.3, 21.9, 20.7, 16.5.
FTIR (neat, cm^{-1}); 2956, 2928, 1656, 1569, 1293, 1249, 1037, 900.
HRMS (EI) *m/z* calcd. for $C_{18}H_{29}IO_2Na^+$, $[M+Na]^+$: 427.1110, found: 427.1104.
$[\alpha]_D^{25}$ −37.9 (*c* 1.0, EtOH).

Allylation of iodoketone 7' to form 8c and 8d

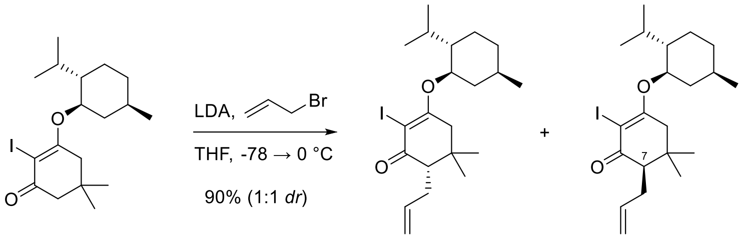

7' 8d 8c

To a solution of freshly prepared lithium diisopropylamide (14.8 mmol, 1.2 equiv) in THF (50 mL) was added a solution of iodo enol ether **7'** (4.97 g, 12.3 mmol) in THF (10 mL) at −78 °C slowly over a period of 1 h, and the resulting yellow solution was continued to stir at −78 °C for 1 h. Allyl bromide (2.13 ml, 24.6mmol, 2.0 equiv) was added and the reaction mixture was warmed to 0 °C. After being stirred at 0 °C for 2 h, the reaction mixture was diluted with EtOAc (150 mL) and saturated aq. NH_4Cl (100 mL). The layers were separated and extracted twice with EtOAc (100 mL). The combined organic extracts were dried over anhydrous $MgSO_4$ and concentrated in vacuo. Purification of the residue by flash column chromatography (silica gel, hexanes/ EtOAc = 10/1 → 5/1) afforded **8d** and **8c** (4.92 g, 11.07 mmol, 90% yield, 1:1 *dr*) as a pale-yellow solid. The diastereomers were separated by performing flash chromatography on a silica gel column (petroleum ether/diethyl ether = 50/1 → 10/1) several times.

The allylated iodoenone **8c** was further recrystallized from DCM/Hexane as a colorless crystal of a tetragonal shape. The crystallized **8c** was analyzed by single-crystal X-ray diffraction (see page **S125**). From the X-ray structure, the C7 absolute stereochemistry of **8c** was confirmed to be of (*R*) configuration.

8c (β-allyl)

$\mathbf{R}_f$ 0.48 (hexanes/EtOAc = 3:1, vanillin)

^{1}H NMR (500 MHz, $CDCl_3$) δ 5.94 – 5.83 (m, 1H), 5.06 – 4.94 (m, 2H), 4.13 (tt, *J* = 13.2, 6.6 Hz, 1H), 2.48 (dd, *J* = 47.1, 17.2 Hz, 2H), 2.37 (ddd, *J* = 11.9, 8.2, 2.4 Hz, 2H), 2.31 – 2.24 (m, 1H), 2.12 – 2.04 (m, 1H), 1.88 – 1.81 (m, 1H), 1.77 – 1.69 (m, 2H), 1.62 (ddt, *J* = 10.3, 6.5, 2.2 Hz, 1H), 1.51 – 1.43 (m, 1H), 1.27 (dd, *J* = 23.3, 12.4 Hz, 1H), 1.14 (s, 3H), 1.10 – 1.05 (m, 1H), 1.03 (s, 3H), 1.02 – 0.98 (m, 1H), 0.96 (d, *J* = 6.5 Hz, 3H), 0.94 (d, *J* = 7.0 Hz, 3H), 0.77 (d, *J* = 7.0 Hz, 3H).

^{13}C NMR (126 MHz, $CDCl_3$) δ 194.8, 173.1, 137.7, 116.0, 81.9, 79.7, 57.1, 47.9, 42.5, 41.3, 36.1, 34.2, 31.8, 31.6, 29.1, 26.3, 24.1, 23.6, 22.2, 21.0, 16.8.

FTIR (neat, cm^{-1}) 2956, 2869, 1657, 1577, 1454, 1349, 1255, 1025, 910.

HRMS (EI) *m/z* calcd. for $C_{21}H_{33}IO_2^+$ $[M]^+$: 444.1521, found: 444.1534.

$[\boldsymbol{\alpha}]_D^{25}$ −15.3 (*c* 1.0, EtOH).

8d (α-allyl)

$\mathbf{R}_f$ 0.47 (hexanes/EtOAc = 3:1, vanillin)

^{1}H NMR (400 MHz, $CDCl_3$) δ 5.85 (ddt, *J* = 16.8, 10.1, 6.9 Hz, 1H), 5.05 – 4.93 (m, 2H), 4.13 (td, *J* = 10.6, 4.4 Hz, 1H), 2.47 (dd, *J* = 61.1, 17.4 Hz, 2H), 2.41 – 2.26 (m, 3H), 2.09 (dtd, *J* = 13.9, 6.9, 2.6 Hz, 1H), 1.85 (dt, *J* = 12.2, 4.6 Hz, 1H), 1.74 (ddd, *J* = 12.1, 7.8, 2.9 Hz, 2H), 1.61 (ddd, *J* = 13.4, 6.3, 3.3 Hz, 1H), 1.46 (dtd, *J* = 14.9, 6.5, 3.4 Hz,

1H), 1.27 (dd, J = 23.3, 12.3 Hz, 1H), 1.13 (s, 3H), 1.10 – 1.05 (m, 1H), 1.05 (s, 3H), 1.01 (dd, J = 14.5, 2.3 Hz, 1H), 0.96 (d, J = 2.8 Hz, 3H), 0.94 (d, J = 3.4 Hz, 3H), 0.78 (d, J = 7.0 Hz, 3H).

^{13}C NMR (126 MHz, $CDCl_3$) δ 194.8, 172.9, 136.9, 115.9, 81.5, 79.5, 56.9, 47.7, 42.2, 40.5, 35.6, 33.9, 31.8, 31.5, 28.8, 26.1, 24.5, 23.4, 22.0, 20.7, 16.5.

FTIR (neat, cm^{-1}) 2957, 2871, 1656, 1575, 1455, 1350, 1254, 1024, 911.

HRMS (ESI) *m/z* calcd. for $C_{21}H_{33}IO_2Na^+$ $[M+Na]^+$: 467.1419, found: 467.1417.

$[\alpha]_D^{25}$ −52.6 (*c* 1.0, EtOH).

Correlation of 8c with 8b via deiodination

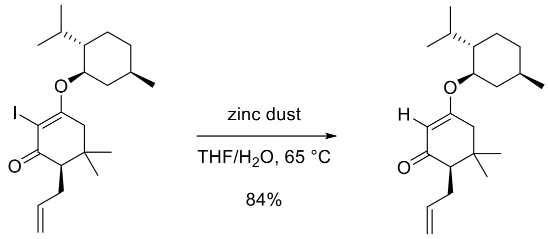

8c **8b**

To a solution of iodide **8c** (2.01 g, 4.53 mmol) in THF/H_2O (20/1, 45 mL) was added zinc dust (2.07 g, 31.7 mmol, 7.0 equiv). The reaction mixture was stirred at 65 °C for 12 h and cooled to 25 °C. The resulting suspension was diluted with EtOAc, washed with an aqueous solution of HCl (100 mL, 1.0 M), water and brine. The combined organic extracts were dried over Na_2SO_4, filtered and concentrated in vacuo. Purification of the residue by flash column chromatography (silica gel, hexanes/EtOAc = 10/1 → 5/1) gave enol ether **8b** (1.21 g, 3.80 mmol, 84% yield) as a colorless liquid.

3.2. Synthetic procedures in the palladium-catalyzed alkoxycarbonylation approach

The Mukaiyama aldol reaction of 6

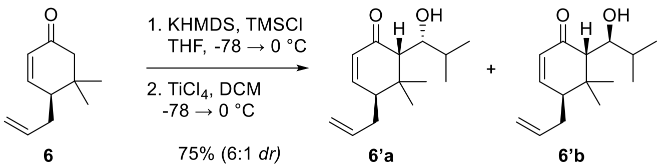

To a solution of potassium bis(trimethylsilyl)amide (KHMDS, 61 mL, 0.5 M in toluene, 30.5 mmol, 1.1 equiv) in THF (50 mL) was added dropwise a solution of enone **6** (4.55 g, 27.7 mmol) in THF (10 mL) at –78 ˚C, and the resulting yellow solution was continued to stir at the same temperature for 1 h. Trimethylsilyl chloride (TMSCl, 5.25 mL, 45.7 mmol, 1.5 equiv) was added to a solution and the reaction mixture was warmed up to 0 ˚C. After being stirred at 0 ˚C for 1.5 h, the reaction mixture was poured into a biphasic mixture of saturated aq. $NaHCO_3$ (150 mL) and diethyl ether (200 mL). The layers were separated and extracted twice with diethyl ether (200 mL). The combined organic extracts were dried over Na_2SO_4 and concentrated in vacuo. The obtained silyl enol ether was used in the next step without purification.

To a mixture of the crude silyl enol ether and isobutyraldehyde (1.37 mL, 30.5 mmol, 1.1 equiv) in DCM (135 mL) was added dropwise a solution of $TiCl_4$ (30.5 mL, 1.0 M in DCM, 30.5 mmol, 1.1 equiv) at –78 ˚C. The resulting light brown solution was allowed to warm to 0 ˚C over a period of 15 min. After being stirred at 0 °C for 1 h, the reaction mixture was diluted with diethyl ether (150 mL) and saturated aq. NH_4Cl (150 mL). The layers were separated and extracted twice with diethyl ether (200 mL). The combined organic extracts were dried over $MgSO_4$ and concentrated in vacuo. Purification of the residue by flash column chromatography (silica gel, hexanes/diethyl ether = 20/1 → 3/1) gave alcohols **6'a** and **6'b** (160 mg, 0.55 mmol, 75% yield, inseparable diastereomeric mixture, 6:1 *dr*) as a white solid. The diastereomeric aldol products were directly used for the next step. Characterization was performed on the analytically pure samples obtained from the partial separation via silica gel column chromatography.

Major isomer 6'a (rear fraction)

$\mathbf{R}_f$ 0.25 (hexanes/EtOAc = 4:1, vanillin)

^{1}H NMR (400 MHz, $CDCl_3$) δ 6.68 (dd, *J* = 10.2, 2.2 Hz, 1H), 5.95 (dd, *J* = 10.2, 2.7 Hz, 1H), 5.84 (dddd, *J* = 16.7, 10.3, 8.9, 5.1 Hz, 1H), 5.19 – 5.09 (m, 2H), 3.82 (t, *J* = 6.3 Hz, 1H), 2.60 (ddd, *J* = 11.5, 5.3, 2.7 Hz, 1H), 2.53 – 2.43 (m, 1H), 2.21 (d, *J* = 8.2 Hz, 1H), 1.86 (ddd, *J* = 13.6, 11.7, 8.9 Hz, 1H), 1.60 (d, *J* = 6.8 Hz, 1H), 1.55 – 1.44 (m, 1H), 1.30 (s, 3H), 0.95 (d, *J* = 6.8 Hz, 3H), 0.89 (d, *J* = 6.6 Hz, 3H), 0.89 (s, 3H).

^{13}C NMR (101 MHz, $CDCl_3$) δ 202.2, 149.9, 136.5, 128.1, 117.6, 73.6, 64.5, 43.5, 39.6, 32.5, 32.2, 27.0, 23.8, 20.8, 14.0.

FTIR (neat, cm^{-1}) 3470, 2965, 2934, 1672, 1469, 1394, 994, 916.

HRMS (CI) *m/z* calcd. for $C_{15}H_{25}O_2^+$ $[M+H]^+$: 237.1855, found:237.1849.

Minor isomer 6'b (front fraction)

^{1}H NMR (400 MHz, $CDCl_3$) δ 6.80 (dd, *J* = 10.2, 3.6 Hz, 1H), 5.99 (d, *J* = 10.2 Hz, 1H), 5.89 – 5.76 (m, 1H), 5.17 – 5.07 (m, 2H), 3.51 (t, *J* = 7.1 Hz, 1H), 2.57 – 2.41 (m, 3H), 2.28 – 2.16 (m, 1H), 2.11 – 1.97 (m, 1H), 1.88 (td, *J* = 13.8, 6.8 Hz, 1H), 1.17 (s, 3H), 1.03 (s, 3H), 0.99 (d, *J* = 6.7 Hz, 3H), 0.92 (d, *J* = 6.6 Hz, 3H).

^{13}C NMR (101 MHz, $CDCl_3$) δ 202.1, 151.9, 136.2, 129.0, 117.3, 75.4, 56.9, 45.7, 38.9, 33.8, 33.6, 25.4, 25.0, 20.1, 18.8.

FTIR (neat, cm^{-1}) 3467, 2963, 2933, 1665, 1472, 1393, 1055, 913.

HRMS (CI) *m/z* calcd. for $C_{15}H_{25}O_2^+$ [M+H]$^+$: 237.1862, found: 237.1849.

Table S1. Dehydration of alcohol **6'**

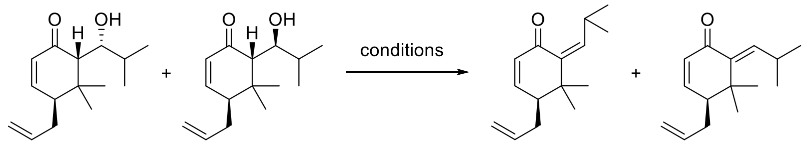

6'a **6'b** (6:1 *dr*) **(Z)-9** **(E)-9**

entry	conditions[a]	results[b,c]
1	5 mol% *p*-TsOH, toluene, 65 °C, 30 min	**(Z)** (51%) + **(E)** (10%)
2	5 mol% *p*-TsOH, benzene, 25 °C, 24 h	**(Z)** (57%) + **(E)** (11%)
3	3 equiv MsCl, 6 equiv TEA, DCM, 25 °C, then basic alumina	complex mixture
4	2.0 equiv Burgess reagent, benzene, 65 °C, 3 h	**(Z)** (52%) + **(E)** (16%)
5	0.5 equiv Triphosgene, 2.0 equiv DMAP, DCM, 25 °C, 12 h	**(Z)** (78%) + **(E)** (4%)

[a] All entry reactions were carried out using a 6:1 diastereomeric mixture of the aldol adducts. [b] Isolated yield. [c] The E/Z information was determined by NOESY experiments.

Dehydration of 6' to dienone 9

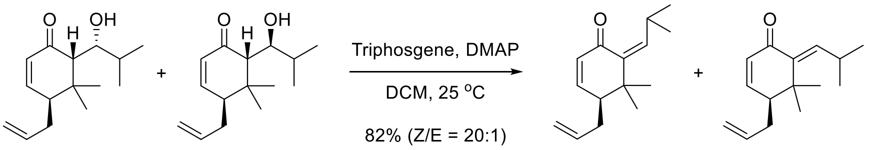

6'a **6'b** (6:1 *dr*) **(Z)-9** **(E)-9**

The reaction was carried out following a literature procedure.[4] To a mixture of alcohol **6'** (717 mg, 3.03 mmol, 1.0 equiv, diastereomeric mixture, 6:1) and DMAP (740 mg, 6.06 mmol, 2.0 equiv) dissolved in DCM (10 mL) was added dropwise a freshly prepared solution of triphosgene (450 mg, 1.52 mmol, 0.5 equiv) in DCM (5 mL). After being stirred at 25 °C for 12 h, the reaction mixture was diluted with DCM (20 mL) and 2 *N* HCl (10 mL). The layers were separated and extracted twice with DCM (20 mL). The combined organic layers were dried over Na_2SO_4, filtered, and concentrated under reduced pressure. The resulting crude oil was then purified by column chromatography (silica gel, hexanes/diethyl ether = 40/1 → 5/1) to afford dienones **(Z)-9** (516 mg, 2.36 mmol, 78% yield) and **(E)-9** (26 mg, 0.12 mmol, 4% yield) both as colorless liquids.

(Z)-9

R*f* 0.48 (hexanes/diethyl ether = 5:1, vanillin)

¹H NMR (500 MHz, $CDCl_3$) δ 6.73 (dd, *J* = 10.1, 4.3 Hz, 1H), 6.03 (dd, *J* = 10.1, 1.2 Hz, 1H), 5.83 – 5.73 (m, 1H), 5.50 (d, *J* = 9.6 Hz, 1H), 5.10 – 5.03 (m, 2H), 3.21 – 3.12 (m, 1H), 2.48 – 2.41 (m, 1H), 2.29 – 2.23 (m, 1H), 2.02 – 1.92 (m, 1H), 1.18 (s, 3H), 1.08 (s, 3H), 0.99 (d, *J* = 5.0 Hz, 3H), 0.98 (d, *J* = 5.0 Hz, 3H).

¹³C NMR (126 MHz, $CDCl_3$) δ 192.7, 150.7, 143.7, 140.5, 136.3, 130.2, 117.3, 48.2, 41.9, 34.9, 28.0, 27.8, 23.6, 23.5, 23.3.

FTIR (neat, cm^{-1}) 2967, 2866, 1667, 1628, 1468, 1392, 915, 839.

HRMS (EI) *m/z* calcd. for $C_{15}H_{22}O^+$ $[M]^+$: 218.1668, found: 218.1671.

$[\alpha]_D^{27}$ +222.6 (*c* 1.0, EtOH).

(E)-9

R*f* 0.36 (hexanes/diethyl ether = 5:1, vanillin)

¹H NMR (400 MHz, $CDCl_3$) δ 6.84 (dd, *J* = 10.0, 5.3 Hz, 1H), 6.43 (d, *J* = 11.0 Hz, 1H), 6.11 (d, *J* = 10.1 Hz, 1H), 5.84 – 5.71 (m, 1H), 5.10 – 5.01 (m, 2H), 3.10 – 2.97 (m, 1H), 2.58 – 2.48 (m, 1H), 2.18 (dt, *J* = 9.6, 4.6 Hz, 1H), 2.09 – 1.96 (m, 1H), 1.39 (s, 3H), 1.28 (s, 3H), 1.04 (d, *J* = 6.5 Hz, 6H).

¹³C NMR (101 MHz, $CDCl_3$) δ 190.7, 151.5, 146.6, 138.4, 135.6, 128.6, 117.1, 48.6, 41.3, 36.1, 30.1, 27.4, 26.1, 23.03, 23.00.

FTIR (neat, cm^{-1}) 2964, 2934, 1733, 1671, 1607, 1467, 1391, 1253, 915, 825.

HRMS (EI) *m/z* calcd. for $C_{15}H_{22}O^+$ $[M]^+$: 218.1673, found: 218.1671.

1D nOe analysis for the determination of the geometry of alkene **9**

(Z) isomer

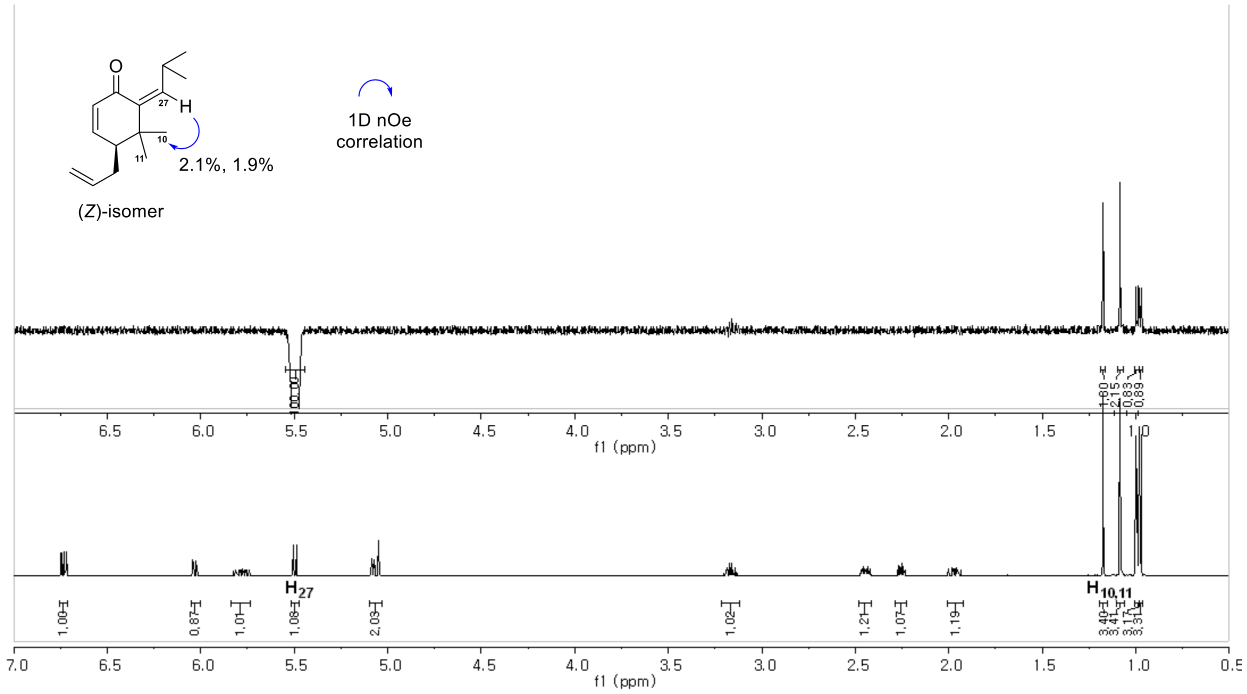

(E) isomer

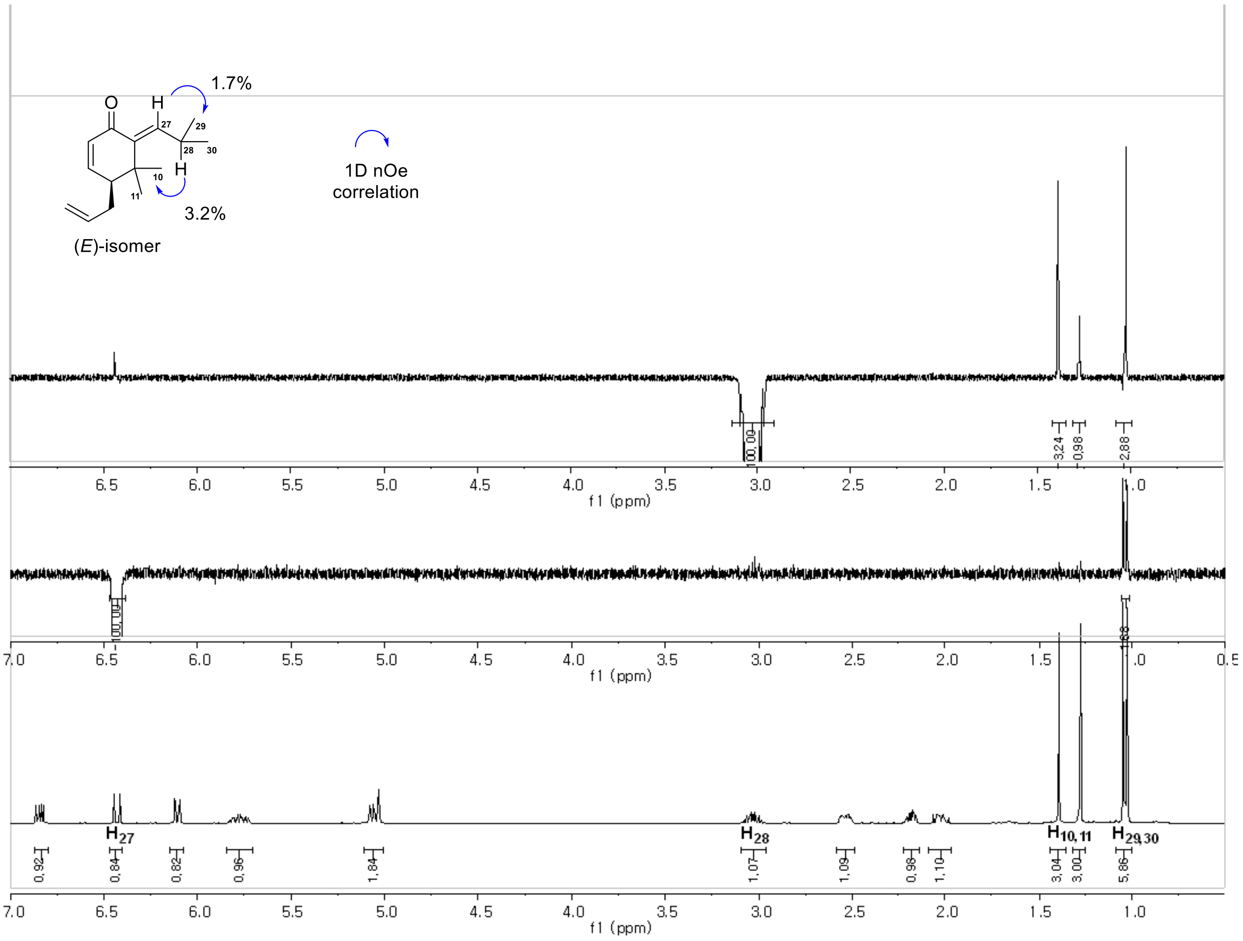

Prenylated enone 10

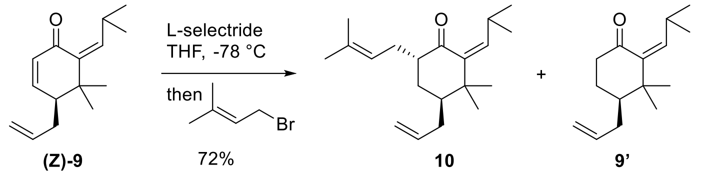

To a solution of dienone **9** (1.36 g, 6.24 mmol) in THF (9 mL) cooled to −78 ˚C was added dropwise a solution of lithium tri-*sec*-butylborohydride (L-selectride, 6.55 mL, 1.0 M in THF, 6.55 mmol, 1.05 equiv). After the addition, the reaction was left to stir at that temperature for 1 h, after which prenyl bromide (1.21 mL, 9.36 mmol, 1.5 equiv) was added. The reaction was then allowed to warm to 0 ˚C. After being stirred at 0 °C for 12 h, the reaction mixture was poured into a biphasic mixture of saturated aq. NH_4Cl (50 mL) and EtOAc (100 mL). The layers were separated and extracted twice with EtOAc (100 mL). The combined organic extracts were dried over Na_2SO_4 and concentrated in vacuo. Purification of the residue by flash column chromatography (silica gel, hexanes/diethyl ether = 100/1 → 20/1) afford the prenylated enone **10** (1.30 g, 4.49 mmol, 72% yield) as a pale-yellow liquid along with a small amount of the simple reduction product *exo*-enone **9’** (26 mg, 0.12 mmol, 2% yield).

Prenylated enone 10

R$_f$ 0.31 (hexanes/EtOAc = 20:1, vanillin)

^{1}H NMR (500 MHz, $CDCl_3$) δ 5.81 – 5.71 (m, 1H), 5.20 (d, *J* = 9.8 Hz, 1H), 5.09 – 4.99 (m, 3H), 2.66 – 2.56 (m, 1H), 2.41 – 2.29 (m, 3H), 2.11 – 2.00 (m, 1H), 1.94 – 1.86 (m, 1H), 1.83 (dt, *J* = 13.9, 5.9 Hz, 1H), 1.77 – 1.71 (m, 1H), 1.68 (s, *J* = 0.9 Hz, 3H), 1.66 – 1.60 (m, 1H), 1.60 (s, *J* = 6.6 Hz, 3H), 1.06 (s, 3H), 1.01 (s, *J* = 5.8 Hz, 3H), 0.98 (d, *J* = 6.6 Hz, 3H), 0.94 (d, *J* = 6.6 Hz, 3H).

^{13}C NMR (126 MHz, $CDCl_3$) δ 209.1, 144.4, 138.0, 137.2, 133.4, 121.9, 116.1, 48.2, 43.4, 42.9, 33.8, 30.0, 28.4, 28.3, 28.1, 26.0, 23.9, 23.8, 23.5, 18.2.

FTIR (neat, cm^{-1}) 3075, 2965, 2928, 1694, 1640, 1466, 1388, 1370, 909.

HRMS (CI) *m/z* calcd. for $C_{20}H_{33}O^+$ $[M+H]^+$: 289.2536, found: 289.2526.

$[\alpha]_D^{25}$ +58.0 (*c* 0.4, EtOH).

***exo*-Enone 9'**

R$_f$ 0.29 (hexanes/EtOAc = 20:1, vanillin)

^{1}H NMR (400 MHz, $CDCl_3$) δ 5.84 – 5.70 (m, 1H), 5.24 (d, *J* = 9.7 Hz, 1H), 5.08 – 4.96 (m, 2H), 2.78 – 2.65 (m, 1H), 2.47 – 2.36 (m, 2H), 2.33 – 2.25 (m, 1H), 2.04 (dtd, *J* = 10.1, 6.7, 3.7 Hz, 1H), 1.90 – 1.80 (m, 1H), 1.72 – 1.64 (m, 1H), 1.64 – 1.55 (m, 1H), 1.11 (s, 3H), 0.96 (d, *J* = 6.7 Hz, 3H), 0.96 (s, 3H), 0.95 (d, *J* = 6.7 Hz, 3H).

^{13}C NMR (101 MHz, $CDCl_3$) δ 207.1, 144.9, 137.7, 137.1, 116.0, 45.0, 42.1, 40.7, 33.8, 27.7, 27.2, 25.0, 23.4, 23.2, 22.8.

FTIR (neat, cm^{-1}) 3077, 2963, 2869, 1695, 1640, 1466, 1388, 1371, 1208, 996, 912.

HRMS (EI) *m/z* calcd. for $C_{15}H_{24}O^+$ $[M]^+$: 220.1827, found: 220.1827.

$[\alpha]_D^{26}$ +17.7 (*c* 1.0, EtOH).

Table S2. Alkynylation of enone **10**

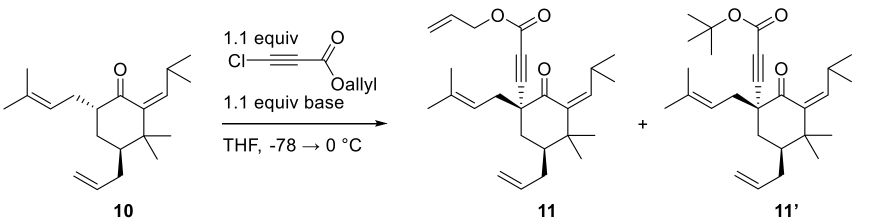

entry	base	results[a,b]
1	LDA	**10** recovered (81%)[c]
2	LiHMDS	**10** recovered (75%)[c]
3	KO*t*Bu	**11** (51%) + **11'** (21%)[d]
4	KHMDS	**11** (87%)

[a]Allyl 3-chloropropiolate was synthesized using the known method by Jørgensen and cowokers (ref 5). [b] Isolated yield. [c] The starting material (**10**) was recovered as an epimeric mixture. [d] *tert*-Butyl ester **11'** may be formed from transesterification induced by KO*t*Bu (ref 6).

Synthesis of allyl 3-chloropropiolate[5]

To a solution of allyl propiolate (6.5 g, 59 mmol, 1.0 equiv) and freshly prepared *tert*-butyl hypochlorite (6.7 ml, 59 mmol, 1.0 equiv) in *t*-BuOH (59 mL) was added *t*-BuOK (697 mg, 5.9 mmol, 0.1 equiv) in two equal portions. The mixture was stirred for 3 h, and then filtered through a pad of silica gel. The residue was washed with 300 ml of diethyl ether and the solvent was carefully removed under reduced pressure (keeping the bath temperature at 0 ˚C). The residue was purified by flash column chromatography (silica gel, petroleum ether/diethyl ether = 100/1 → 40/1) to furnish allyl 3-chloropropiolate (3.7 g, 25.4 mmol, 43% yield) as a pale-yellow liquid (can be stored in the refrigerator).

R*f* 0.49 (hexanes/EtOAc = 10:1, $KMnO_4$)
^{1}H NMR (500 MHz, $CDCl_3$) δ 5.92 (ddt, *J* = 17.1, 10.4, 5.9 Hz, 1H), 5.38 (ddd, *J* = 17.2, 2.8, 1.4 Hz, 1H), 5.33 – 5.28 (m, 1H), 4.69 – 4.66 (m, 2H).

Alkynylation of 10 to form alkynoate 11

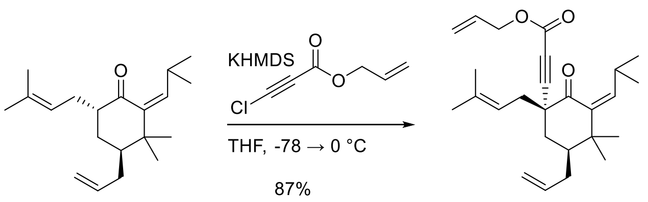

10 **11**

To a solution of enone **10** (865 mg, 3.0 mmol) in THF (60 mL) was added dropwise a solution of KHMDS (6.6 mL, 0.5 M in toluene, 3.3 mmol, 1.1 equiv) at –78 ˚C. The resulting mixture was stirred at that temperature for 1 h, after which allyl 3-chloropropiolate (477 mg, 3.3 mmol, 1.1 equiv) was added. After being stirred at 0 ˚C for 1 h, the reaction mixture was diluted with EtOAc (100 mL) and a saturated aq. NH_4Cl (100 mL). The layers were separated and extracted twice with EtOAc (100 mL). The combined organic phases were washed with a saturated aq. NaCl (150 mL), dried over Na_2SO_4 and concentrated in vacuo. Purification of the residue by flash column chromatography (silica gel, hexanes/EtOAc = 40/1 → 10/1) gave alkynyl enone **11** (1.02 g, 2.6 mmol, 87% yield) as a pale-yellow liquid.

Alkynoate 11
R*f* 0.52 (hexanes/diethyl ether = 10:1, $KMnO_4$)
^{1}H NMR (499 MHz, $CDCl_3$) δ 5.94 – 5.84 (m, 1H), 5.76 (dddd, *J* = 15.8, 10.4, 8.3, 5.2 Hz, 1H), 5.35 (d, *J* = 10.0 Hz, 1H), 5.35 – 5.24 (m, 2H), 5.23 – 5.17 (m, 1H), 5.03 (ddd, *J* = 7.6, 6.2, 5.6 Hz, 2H), 4.62 (dt, *J* = 5.8, 1.3 Hz, 2H), 2.57 (dd, *J* = 14.5, 7.3 Hz, 1H), 2.48 – 2.40 (m, 2H), 2.37 (dd, *J* = 14.6, 7.6 Hz, 1H), 2.15 (dd, *J* = 14.0, 3.6 Hz, 1H), 1.98 (ddt, *J* = 13.8, 10.5, 3.4 Hz, 1H), 1.73 (s, 3H), 1.70 (dd, *J* = 14.3, 4.0 Hz, 1H), 1.64 (s, 3H), 1.50 – 1.41 (m, 1H), 1.16 (s, 3H), 1.01 (d, *J* = 6.6 Hz, 3H), 0.95 (d, *J* = 6.5 Hz, 3H), 0.79 (s, 3H).
^{13}C NMR (126 MHz, $CDCl_3$) δ 201.4, 153.0, 144.2, 137.5, 137.2, 135.6, 131.2, 119.0, 118.4, 116.4, 88.5, 76.8, 66.3, 50.7, 42.3, 42.3, 37.8, 34.5, 33.9, 28.6, 26.0, 24.9, 23.4, 23.0, 21.0, 18.1.
FTIR (neat, cm^{-1}) 3078, 2968, 2932, 2226, 1714, 1639, 1448, 1371, 1236, 918.
HRMS (CI) *m/z* calcd. for $C_{26}H_{37}O_3^+$ $[M+H]^+$: 397.2746, found: 397.2737.

$[\alpha]_D^{24}$ +269.2 (*c* 1.1, EtOH).

In Table S2 (entry 3), when KO*t*Bu was employed as a base, along with the desired alkynoate **11** (51%), a significant amount (21%) of *tert*-butyl ester **11'** was generated presumably via transesterification induced by KO*t*Bu.[6]

***tert*-Butyl alkynoate 11'**

R*f* 0.50 (hexanes/diethyl ether = 10:1, $KMnO_4$)

^{1}H NMR (400 MHz, $CDCl_3$) δ 5.84 – 5.73 (m, 1H), 5.33 (d, *J* = 10.1 Hz, 1H), 5.20 (t, *J* = 7.5 Hz, 1H), 5.09 – 4.99 (m, 2H), 2.55 (dd, *J* = 14.5, 7.4 Hz, 1H), 2.50 – 2.39 (m, 2H), 2.36 (dd, *J* = 14.7, 7.8 Hz, 1H), 2.18 – 2.09 (m, 1H), 2.06 – 1.93 (m, 1H), 1.73 (s, 3H), 1.72 – 1.66 (m, 1H), 1.64 (s, 3H), 1.51 – 1.41 (m, 10H), 1.16 (s, 3H), 1.01 (d, *J* = 6.6 Hz, 3H), 0.96 (d, *J* = 6.5 Hz, 3H), 0.79 (s, 3H).

^{13}C NMR (101 MHz, $CDCl_3$) δ 201.9, 152.5, 144.3, 137.3, 137.2, 135.4, 118.5, 116.3, 85.3, 83.0, 78.4, 50.5, 42.3, 42.1, 37.7, 34.6, 33.9, 28.6, 27.9, 26.0, 24.8, 23.4, 23.0, 21.0, 18.1.

FTIR (neat, cm^{-1}) 2973, 2935, 2360, 2341, 1708, 1370, 1282, 1254, 1155, 668.

HRMS (ESI) *m/z* calcd. for $C_{27}H_{40}O_3Na^+$ $[M+Na]^+$ 435.2872, found 435.2870.

Decarboxylative allylation of 11 to form 12a

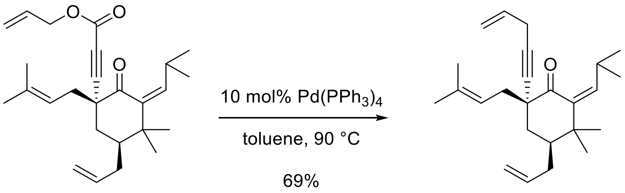

11 12a

The reaction was carried out following a literature procedure.[7] To a solution of allyl alkynoate **11** (158 mg, 0.40 mmol) in toluene (6.6 mL) was added tetrakis(triphenylphosphine)palladium(0) (46 mg, 0.04 mmol, 0.1 equiv), and the resulting suspension was heated at 90 ˚C for 24 h. Then the reaction mixture was cooled to 25 °C and concentrated in vacuo. Purification of the residue by flash column chromatography (silica gel, hexanes/EtOAc = 100/1 → 10/1) afforded enyne **12a** (98 mg, 0.28 mmol, 69% yield) as a pale-yellow liquid.

R*f* 0.67 (hexanes/diethyl ether = 10:1, vanillin)

^{1}H NMR (500 MHz, $CDCl_3$) δ 5.83 – 5.70 (m, 2H), 5.29 (d, *J* = 9.9 Hz, 1H), 5.32 – 5.21 (m, 2H), 5.11 – 4.99 (m, 3H), 2.92 (dt, *J* = 3.9, 1.7 Hz, 2H), 2.50 (dd, *J* = 14.5, 7.1 Hz, 1H), 2.48 – 2.43 (m, 1H), 2.43 – 2.37 (m, 1H), 2.31 (dd, *J* = 14.5, 7.4 Hz, 1H), 2.07 – 1.99 (m, 2H), 1.77 – 1.73 (m, 1H), 1.72 (s, 3H), 1.62 (s, 3H), 1.39 (t, *J* = 13.9 Hz, 1H), 1.15 (s, 3H), 1.01 (d, *J* = 6.6 Hz, 3H), 0.89 (d, *J* = 6.5 Hz, 3H), 0.78 (s, 3H).

^{13}C NMR (126 MHz, $CDCl_3$) δ 204.1, 145.0, 137.9, 136.8, 134.3, 132.8, 120.1, 116.2, 116.0, 83.2, 81.8, 73.2, 50.5, 42.4, 42.4, 38.7, 35.0, 34.8, 28.7, 26.2, 25.2, 23.7, 23.4, 21.3, 18.4.

FTIR (neat, cm^{-1}) 3079, 2967, 2931, 1704, 1641, 1468, 1387, 990, 912.

HRMS (CI) *m/z* calcd. for $C_{25}H_{37}O^+$ $[M+H]^+$: 353.2847, found: 353.2849.

$[\alpha]_D^{20}$ +298.0 (*c* 1.0, EtOH).

Oxidation of enyne 12a to epoxide 12'a

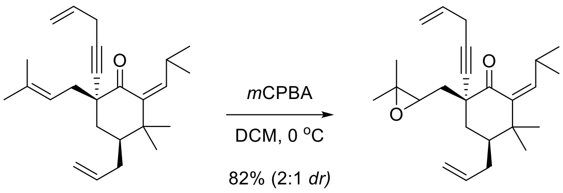

12a **12'a**

To a solution of enyne **12a** (245 mg, 0.69 mmol) in DCM (14 mL) was added *m*-CPBA (181 mg, 0.76 mmol, 1.1 equiv) at 0 ˚C. The reaction mixture was then stirred at that temperature for 1 h, at which time the reactant was completely consumed as judged by TLC. After addition of a saturated aqueous solution of $NaHCO_3$ and warming to 25 °C, the reaction mixture was extracted with DCM. The combined organic extracts were dried over Na_2SO_4 and concentrated in vacuo. Purification by flash column chromatography (silica gel, hexanes/EtOAc = 20:1 → 4:1) afforded epoxide **12'a** (210 mg, 0.57 mmol, 82% yield, inseparable isomeric mixture, 2:1 *dr*) as a colorless liquid.

Characterization data for the signals from the major isomer of **12'a**

R*f* 0.44 (hexanes/EtOAc = 8:1, vanillin)

^{1}H (400 MHz, $CDCl_3$) δ 5.86 – 5.70 (m, 1H), 5.32 (d, *J* = 10.2 Hz, 1H), 5.28 – 5.23 (m, 1H), 5.14 – 5.00 (m, 2H), 3.01 (dd, *J* = 7.1, 3.8 Hz, 1H), 2.98 – 2.91 (m, 2H), 2.49 – 2.36 (m, 2H), 2.35 – 2.23 (m, 1H), 2.14 – 2.04 (m, 1H), 2.04 – 1.87 (m, 1H), 1.81 – 1.70 (m, 1H), 1.62 – 1.43 (m, 2H), 1.33 (s, 3H), 1.30 (s, 3H), 1.29 – 1.24 (m, 1H), 1.17 (s, 3H), 1.01 (d, *J* = 6.6 Hz, 3H), 0.96 – 0.92 (m, 1H), 0.91 (d, *J* = 6.5 Hz, 3H), 0.79 (s, 3H).

^{13}C NMR (101 MHz, $CDCl_3$) δ 203.4, 144.5, 137.4, 136.7, 132.3, 116.2, 116.0, 82.8, 81.8, 61.3, 58.0, 57.4, 49.5, 48.8, 42.4, 42.3, 42.0, 39.5, 35.5, 34.4, 28.5, 24.8, 23.4, 23.1, 21.1, 19.1.

FTIR (neat, cm^{-1}) 3270, 2966, 2867, 1699, 1641, 1467, 1381, 991, 912.

HRMS (CI) *m/z* calcd. for $C_{25}H_{37}O_2^+$ $[M+H]^+$: 369.2791, found: 369.2788.

$[\alpha]_D^{26}$ +205.5 (*c* 0.4, EtOH).

Hydrolysis of epoxide 12'a to diol 13a

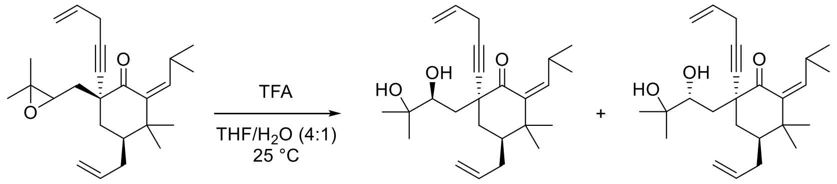

12'a(2:1 *dr*) **13a** 57% **13a'** 38%

To a solution of epoxide **12'a** (442 mg, 1.20 mmol) in THF (12.8 mL) was added TFA (3.2 mL, 2 *N* in H_2O). After stirring at 25 °C for 12 h, a saturated aqueous solution of $NaHCO_3$ was added, and the reaction mixture was extracted twice with EtOAc. The combined organic extracts were washed with brine, dried over Na_2SO_4, filtered, and concentrated in vacuo. The crude oil was purified by flash column chromatography (silica gel, hexanes/EtOAc = 20/1

→ 3/1) to give diol **13a** (263 mg, 0.68 mmol, 57% yield) and **13a'** (147 mg, 0.46 mmol, 38% yield) both as colorless liquids.

13a (major isomer)

R*f* 0.15 (hexanes/EtOAc = 4:1, vanillin)

^{1}H NMR (400 MHz, $CDCl_3$) δ 5.77 (tdd, *J* = 15.4, 9.8, 5.4 Hz, 2H), 5.31 (d, *J* = 10.0 Hz, 1H), 5.35 – 5.21 (m, 1H), 5.14 – 5.00 (m, 3H), 3.70 (dd, *J* = 9.0, 3.2 Hz, 1H), 2.95 (d, *J* = 4.6 Hz, 2H), 2.53 – 2.31 (m, 3H), 2.24 – 2.01 (m, 3H), 1.83 – 1.71 (m, 1H), 1.67 (dd, *J* = 14.5, 5.0 Hz, 1H), 1.63 – 1.54 (m, 1H), 1.45 (s, 1H), 1.25 (s, *J* = 5.2 Hz, 3H), 1.21 (s, 3H), 1.16 (s, 3H), 1.01 (d, *J* = 6.6 Hz, 3H), 0.90 (d, *J* = 6.5 Hz, 3H), 0.81 (s, 3H).

^{13}C NMR (101 MHz, $CDCl_3$) δ 205.1, 144.6, 137.4, 136.6, 132.0, 116.3, 116.2, 83.5, 82.8, 75.6, 73.0, 49.3, 42.5, 42.0, 39.6, 38.4, 34.3, 28.5, 27.9, 25.6, 24.8, 23.6, 23.4, 23.1, 21.1.

FTIR (neat, cm^{-1}) 3453, 2971, 2936, 1698, 1641, 1387, 1370, 1162, 993, 913.

HRMS (FAB) *m/z* calcd. for $C_{25}H_{39}O_3^+$ $[M+H]^+$: 387.2895, found: 387.2894.

$[\alpha]_D^{24}$ +236.8 (*c* 0.5, EtOH).

13a' (minor isomer)

R*f* 0.16 (hexanes/EtOAc = 4:1, vanillin)

^{1}H NMR (400 MHz, $CDCl_3$) δ 5.83 – 5.69 (m, 2H), 5.34 (d, *J* = 10.0 Hz, 1H), 5.31 – 5.22 (m, 1H), 5.13 – 5.01 (m, 3H), 4.56 (s, 1H), 3.74 (d, *J* = 8.8 Hz, 1H), 2.99 – 2.91 (m, 2H), 2.57 (bs, 1H), 2.47 – 2.33 (m, 2H), 2.21 – 2.03 (m, 2H), 1.89 (dd, *J* = 14.7, 9.0 Hz, 1H), 1.70 (ddd, *J* = 38.0, 21.1, 12.2 Hz, 2H), 1.49 (t, *J* = 13.0 Hz, 1H), 1.21 (s, 3H), 1.20 (s, 3H), 1.17 (s, 3H), 1.00 (d, *J* = 6.6 Hz, 3H), 0.90 (d, *J* = 6.5 Hz, 3H), 0.79 (s, 3H).

^{13}C NMR (101 MHz, $CDCl_3$) δ 207.9, 144.3, 137.3, 137.3, 132.1, 116.4, 116.2, 84.4, 80.9, 75.1, 72.7, 50.9, 42.6, 42.1, 42.1, 39.5, 34.3, 28.7, 25.5, 24.7, 24.0, 23.3, 23.1, 23.1, 21.1.

FTIR (neat, cm^{-1}) 3453, 2971, 2936, 1688, 1641, 1467, 1387, 1371, 1167, 1064, 993, 914.

HRMS (FAB) *m/z* calcd. for $C_{25}H_{39}O_3^+$ $[M+H]^+$: 387.2894, found: 387.2894.

$[\alpha]_D^{24}$ +262.8 (*c* 1.0, EtOH).

Table S3. Palladium-catalyzed oxycarbonylation of enyne diol **13a**

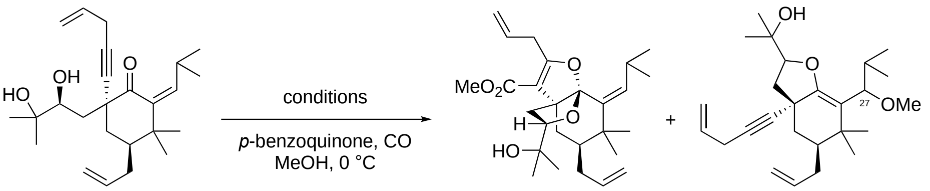

13a **16** **15’**

Entry	conditions[a]	results[b,c]
1	$Pd(CH_3CN)_2Cl_2$	**16** (trace) + **15’** (41%)
2	$Pd(CH_3CN)_4(BF_4)_2$	**16** (7%) + **15’** (31%)
3	$Pd(TFA)_2$	**16** (8%) + **15’** (30%)
4	$Pd_2(dba)_3{\cdot}CHCl_3$	**13a** recovered
5	$Pd(OAc)_2$	**13a** recovered
6	$Pd(TFA)_2$, 15 mol % pyridine	**13a** recovered
7	$Pd(TFA)_2$, 15 mol % (+)-PyBOX	**16** (5%) + **15’** (35%)
8	$Pd(TFA)_2$, 15 mol % (+)-PhBOX, 0 to 25 °C, 24 h	complex mixture
9	$Pd(CH_3CN)_2Cl_2$, 20 mol % DtBpy	**16** (49%) + **15’** (trace)

[a]10 mol % of a palladium catalyst was used in all reactions. [b]Isolated yield. [c]Methyl ether **15’** was obtained as a single isomer with undetermined C27 stereochemistry.

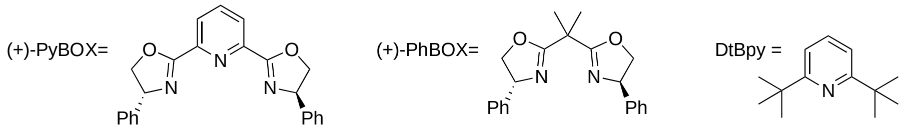

Palladium-catalyzed alkoxycarbonylation of 13a to tricyclic ketal 16

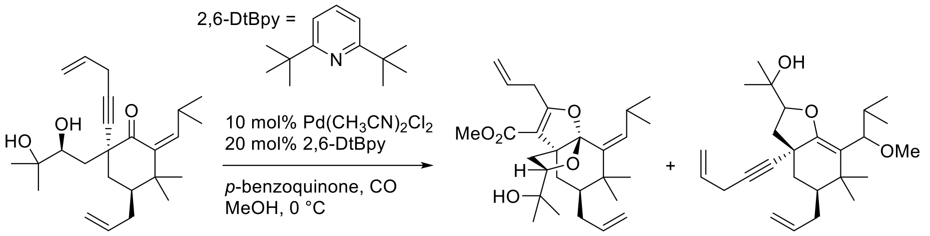

13a 49% **16** **15’**

The reaction was carried out following a modified literature procedure.[8] A round bottom flask, containing a Teflon-coated magnetic bar, bis(acetonitrile)dichloropalladium(II) (3.4 mg, 0.013 mmol, 0.1 equiv), 1,4-benzoquinone (28 mg, 0.29 mmol, 2.0 equiv), 2,6-di-*tert*-butylpyridine (0.052 ml, 0.5 M solution in MeOH, 0.026 mmol, 0.2 equiv) and 4.3 mL of MeOH, was fitted with a rubber septum. The apparatus was purged with carbon monoxide using a balloon several times. When the suspension was stirred for 30 min, it became a clear yellow solution. At this time, a solution of diol **13a** (50 mg, 0.13 mmol) in MeOH (0.5 mL) was added dropwise to the stirred mixture via a syringe at 0 ˚C. After stirring for 12 h, the mixture was diluted with DCM, washed with aqueous 5% NaOH solution, dried over $MgSO_4$, and concentrated. The crude material was purified by column chromatography (silica gel, hexanes/EtOAc = 20/1 →

4/1) to give tricyclic ketal **16** (28 mg, 0.06 mmol, 49% yield) and methyl ether **15'** (3 mg, 0.003 mmol, 2% yield) both as colorless liquids.

Tricyclic ketal 16

R*f* 0.45 (hexanes/EtOAc = 4:1, vanillin)

^{1}H NMR (400 MHz, $CDCl_3$) δ 5.77 (ddt, *J* = 16.8, 10.1, 6.6 Hz, 1H), 5.69 – 5.56 (m, 1H), 5.42 (d, *J* = 10.3 Hz, 1H), 5.19 – 5.03 (m, 2H), 5.02 – 4.91 (m, 2H), 3.94 (dd, *J* = 9.1, 7.4 Hz, 1H), 3.70 (s, 3H), 3.44 – 3.35 (m, 1H), 3.33 – 3.20 (m, 2H), 2.40 – 2.30 (m, 1H), 2.28 – 2.22 (m, 1H), 2.21 (dd, *J* = 12.8, 9.3 Hz, 1H), 2.06 – 1.97 (m, 2H), 1.69 – 1.57 (m, 1H), 1.27 (s, *J* = 8.1 Hz, 3H), 1.23 – 1.14 (m, 2H), 1.07 (s, 6H), 0.96 (d, *J* = 2.7 Hz, 3H), 0.94 (d, *J* = 3.6 Hz, 3H), 0.94 (s, 3H).

^{13}C NMR (101 MHz, $CDCl_3$) δ 165.5, 165.3, 141.5, 140.0, 138.1, 131.9, 119.5, 117.6, 115.6, 109.2, 85.1, 70.5, 57.6, 50.6, 40.5, 40.3, 37.6, 34.6, 32.7, 31.3, 29.9, 28.3, 27.8, 25.0, 24.1, 23.1, 23.0.

FTIR (neat, cm^{-1}) 3499, 2971, 2867, 1702, 1639, 1437, 1365, 1203, 1110, 993, 912.

HRMS (FAB) *m/z* calcd. for $C_{27}H_{41}O_5^+$ $[M+H]^+$: 445.2948, found: 445.2949.

$[\alpha]_D^{25}$ +80.8 (*c* 1.0, EtOH).

Methyl ether 15'

R*f* 0.61 (hexanes/EtOAc = 4:1, vanillin)

^{1}H NMR (500 MHz, $CDCl_3$) δ 5.86 – 5.75 (m, 2H), 5.33 (dd, *J* = 16.9, 1.7 Hz, 1H), 5.14 – 5.02 (m, 3H), 4.27 (dd, *J* = 9.6, 5.8 Hz, 1H), 4.07 (s, 1H), 3.36 (s, *J* = 13.1 Hz, 3H), 3.09 (d, *J* = 9.3 Hz, 1H), 2.98 – 2.92 (m, 2H), 2.53 (tt, *J* = 13.6, 6.8 Hz, 1H), 2.36 (dd, *J* = 14.0, 2.8 Hz, 1H), 2.15 (dd, *J* = 11.6, 5.9 Hz, 1H), 2.06 (dd, *J* = 12.3, 1.8 Hz, 1H), 1.90 – 1.77 (m, 2H), 1.76 – 1.65 (m, 1H), 1.36 (s, 3H), 1.30 – 1.19 (m, 1H), 1.06 (d, *J* = 6.5 Hz, 3H), 1.02 (s, 3H), 1.01 (s, 3H), 0.90 (s, 3H), 0.86 (d, *J* = 7.1 Hz, 3H).

^{13}C NMR (126 MHz, $CDCl_3$) δ 154.1, 138.3, 133.0, 115.9, 115.7, 115.5, 86.6, 86.3, 85.3, 77.1, 70.7, 59.0, 42.1, 41.7, 39.5, 36.8, 35.5, 34.4, 34.1, 28.9, 26.8, 23.1, 23.0, 22.1, 21.0, 20.5.

Alloc removal of allyl alkynoate 11 to terminal alkyne 12b

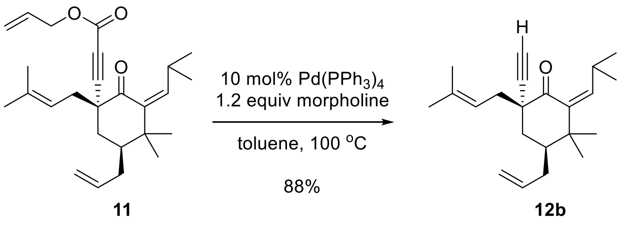

To a solution of alkynoate **11** (265 mg, 0.67 mmol) in toluene (13.4 mL) were added tetrakis(triphenylphosphine)-palladium(0) (77 mg, 0.07 mmol, 0.1 equiv) and morpholine (0.06 ml, 0.80 mmol, 1.2 equiv) at 25 °C, and the resulting suspension was stirred at 100 ˚C for 1 h. Then the reaction mixture was cooled to 25 °C and concentrated in vacuo. Purification of the residue by flash column chromatography (silica gel, hexanes/EtOAc = 100/1 → 10/1) afforded terminal alkyne **12b** (185 mg, 0.59 mmol, 88% yield) as a pale-yellow liquid.

R$_f$ 0.61 (hexanes/EtOAc = 8:1, vanillin)

^{1}H NMR (400 MHz, $CDCl_3$) δ 5.84 – 5.72 (m, 1H), 5.31 (d, *J* = 10.0 Hz, 1H), 5.24 (dq, *J* = 5.9, 4.3 Hz, 1H), 5.04 (ddd, *J* = 6.6, 1.8, 1.1 Hz, 2H), 2.52 (dd, *J* = 14.7, 7.0 Hz, 1H), 2.48 – 2.37 (m, 2H), 2.33 (dd, *J* = 14.7, 7.6 Hz, 1H), 2.22 (s, 1H), 2.08 (dd, *J* = 13.5, 3.6 Hz, 1H), 2.06 – 1.98 (m, 1H), 1.73 (s, 3H), 1.63 (s, 3H), 1.59 (dd, *J* = 11.6, 5.6 Hz, 1H), 1.39 (dd, *J* = 13.3, 12.5 Hz, 1H), 1.16 (s, 3H), 1.01 (d, *J* = 6.6 Hz, 3H), 0.93 (d, *J* = 6.5 Hz, 3H), 0.79 (s, 3H).

^{13}C NMR (101 MHz, $CDCl_3$) δ 203.3, 144.3, 137.5, 136.8, 134.6, 119.3, 116.1, 84.5, 73.0, 50.2, 42.2, 42.1, 38.1, 34.5, 34.3, 28.5, 25.9, 24.9, 23.4, 23.1, 21.0, 18.1.

FTIR (neat, cm^{-1}) 3309, 2967, 2934, 1703, 1640, 1466, 1371, 993, 913, 636.

HRMS (CI) *m/z* calcd. for $C_{22}H_{33}O^+$ $[M+H]^+$: 313.2525, found: 313.2526.

$[\alpha]_D^{24}$ +216.4 (*c* 0.7, EtOH).

Oxidation of 12b to epoxide 12'b

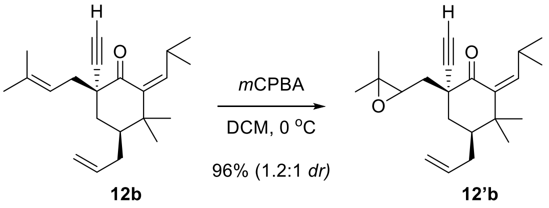

To a solution of alkyne **12b** (57 mg, 0.18 mmol) in DCM (4 mL) was added *m*-CPBA (36 mg, 0.20 mmol, 1.1 equiv) at 0 ˚C. The reaction mixture was then stirred at that temperature for 1 h, at which time the TLC analysis indicated complete consumption of the starting material. After addition of a saturated aqueous solution of $NaHCO_3$ and warming to 25 °C, the reaction mixture was extracted with DCM. The combined organic extracts were dried over Na_2SO_4 and concentrated in vacuo. Purification by flash column chromatography (silica gel, hexanes/EtOAc = 20:1 → 4:1) afforded epoxide **12'b** (57 mg, 0.17 mmol, 96% yield, inseparable isomeric mixture, 1.2:1 *dr*) as a colorless liquid.

Characterization data for the signals from the major isomer of **12'b**

R$_f$ 0.36 (hexanes/EtOAc = 8:1, vanillin)

^{1}H NMR (400 MHz, $CDCl_3$) δ 5.86 – 5.72 (m, 1H), 5.33 (d, *J* = 10.0 Hz, 1H), 5.11 – 5.01 (m, 2H), 3.01 (dd, *J* = 7.5, 3.5 Hz, 1H), 2.49 – 2.36 (m, 2H), 2.36 – 2.27 (m, 2H), 2.18 – 2.06 (m, 1H), 2.06 – 1.88 (m, 1H), 1.81 – 1.70 (m, 1H), 1.62 – 1.44 (m, 2H), 1.34 (s, 3H), 1.30 (s, 3H), 1.18 (s, 3H), 1.01 (d, *J* = 6.6 Hz, 3H), 0.94 (d, *J* = 6.5 Hz, 3H), 0.80 (s, 3H).

^{13}C NMR (101 MHz, $CDCl_3$) δ 203.0, 144.0, 137.3, 136.7, 116.4, 83.4, 74.1, 61.1, 57.4, 49.4, 42.4, 42.2, 39.2, 35.3, 34.3, 28.6, 24.8, 24.7, 23.3, 23.1, 21.0, 19.1.

FTIR (neat, cm^{-1}) 3277, 2965, 2934, 1704, 1640, 1465, 1377, 1121, 995, 913.

HRMS (FAB) *m/z* calcd. for $C_{22}H_{33}O_2^+$ $[M+H]^+$: 329.2487, found: 329.2475.

$[\alpha]_D^{24}$ +154.8 (*c* 0.6, EtOH).

Hydrolysis of epoxide 12’b to diol 13b

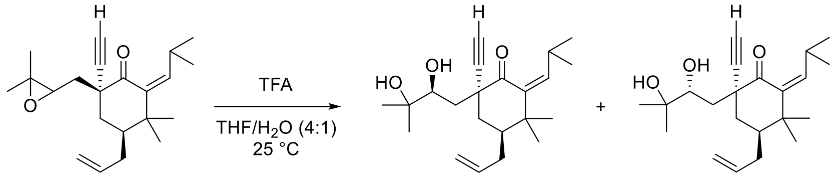

12’b (1.2:1 *dr*) **13b** 42% **13b’** 32%

To a solution of epoxide **12’b** (252 mg, 0.77 mmol) in THF (6.2 mL) was added TFA (1.5 mL, 2.0 M in H_2O). After 12 h, a saturated aqueous $NaHCO_3$ was added, and the aqueous layer was extracted twice with EtOAc. The combined organic extracts were washed with brine, dried over Na_2SO_4, filtered, and concentrated in vacuo. The crude oil was purified by flash column chromatography (silica gel, hexanes/EtOAc = 20/1 → 3/1) to give diol **13b** (112 mg, 0.32 mmol, 42% yield) and diol **13b'** (84 mg, 0.24 mmol, 32% yield) both as colorless liquids.

13b (major isomer)

R*f* 0.28 (hexanes/diethyl ether = 10:1, vanillin)

^{1}H NMR (400 MHz, $CDCl_3$) δ 5.85 – 5.72 (m, 1H), 5.33 (d, *J* = 10.1 Hz, 1H), 5.11 – 5.02 (m, 2H), 3.70 (dd, *J* = 9.4, 4.1 Hz, 1H), 2.84 (d, *J* = 4.2 Hz, 1H), 2.47 – 2.39 (m, 1H), 2.38 (s, 1H), 2.31 (s, 1H), 2.21 (dd, *J* = 13.8, 3.6 Hz, 1H), 2.14 – 2.03 (m, 2H), 1.81 – 1.70 (m, 2H), 1.70 – 1.57 (m, 2H), 1.26 (s, 3H), 1.22 (s, 3H), 1.17 (s, 3H), 1.01 (d, *J* = 6.6 Hz, 3H), 0.94 (d, *J* = 6.5 Hz, 3H), 0.82 (s, 3H).

^{13}C NMR (101 MHz, $CDCl_3$) δ 204.6, 144.1, 137.3, 136.8, 116.3, 84.5, 75.5, 74.5, 73.1, 49.4, 42.6, 42.0, 39.2, 37.9, 34.3, 28.6, 25.6, 24.8, 23.6, 23.3, 23.0, 21.0.

FTIR (neat, cm^{-1}) 3447, 3305, 2970, 2936, 1766, 1699, 1472, 1370, 1389, 1240, 1062, 992, 918.

HRMS (FAB) *m/z* calcd. for $C_{22}H_{35}O_3^+$ $[M+H]^+$: 347.2589, found: 347.2581.

$[\alpha]_D^{23}$ +210.0 (*c* 1.0, EtOH).

13b' (minor isomer)

R*f* 0.33 (hexanes/EtOAc = 3:1, vanillin)

^{1}H NMR (400 MHz, $CDCl_3$) δ 5.85 – 5.71 (m, 1H), 5.36 (d, *J* = 10.1 Hz, 1H), 5.12 – 5.01 (m, 2H), 4.42 (d, *J* = 2.9 Hz, 1H), 3.74 (ddd, *J* = 9.1, 2.7, 1.6 Hz, 1H), 2.51 (s, 1H), 2.47 – 2.41 (m, 1H), 2.41 – 2.39 (m, 1H), 2.39 – 2.32 (m, 1H), 2.20 – 2.07 (m, 2H), 1.93 (dd, *J* = 14.8, 9.1 Hz, 1H), 1.79 – 1.70 (m, 1H), 1.73 – 1.63 (m, 1H), 1.51 (t, *J* = 13.7 Hz, 1H), 1.23 (s, 3H), 1.21 (s, 3H), 1.19 (s, 3H), 1.00 (d, *J* = 6.6 Hz, 3H), 0.94 (d, *J* = 6.5 Hz, 3H), 0.80 (s, 3H).

^{13}C NMR (101 MHz, $CDCl_3$) δ 207.5, 143.9, 137.5, 137.1, 116.6, 82.6, 75.4, 75.0, 72.7, 50.8, 42.7, 42.0, 41.8, 38.9, 34.2, 28.8, 25.5, 24.7, 24.1, 23.3, 23.1, 21.0.

FTIR (neat, cm^{-1}) 3460, 3310, 2969, 2936, 1690, 1641, 1467, 1371, 1062, 993, 918.

HRMS (FAB) *m/z* calcd. for $C_{22}H_{35}O_3^+$ $[M+H]^+$: 347.2589, found: 347.2581.

$[\boldsymbol{\alpha}]_{\boldsymbol{D}}^{\mathbf{24}}$ +174.1 (*c* 0.6, EtOH).

Palladium-catalyzed alkoxycarbonylation of 13b to methyl ester 17

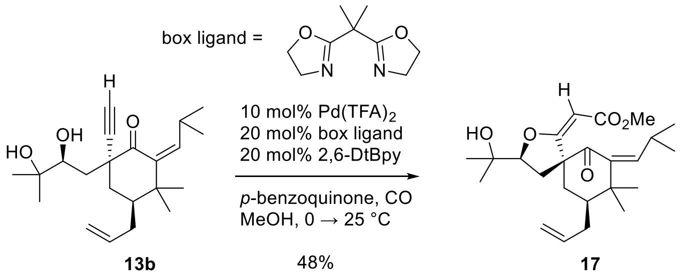

The box ligand was synthesized following a literature procedure.[9,10] A round bottom flask, containing a Teflon-coated magnetic bar, palladium(II) trifluoroacetate (2 mg, 0.006 mmol, 0.1 equiv), 1,4-benzoquinone (13 mg, 0.12 mmol, 2.0 equiv), 2,6-di-*tert*-butylpyridine (0.024 ml, 0.5 M solution in MeOH, 0.012 mmol, 0.2 equiv), box ligand (2.2 mg, 0.012 mmol, 0.2 equiv) and MeOH (2 mL), was fitted with a rubber septum. The apparatus was purged with carbon monoxide using a balloon several times. When the suspension was stirred for 30 min, it became a clear yellow solution. At this time, a solution of diol **13b** (21 mg, 0.06 mmol) in MeOH (0.5 mL) was added dropwise to the stirred mixture via a syringe at 0 ˚C. Raised the reaction temperature to 25 ˚C, the color of the solution turns dark yellow. After stirring for 12 h, the mixture was diluted with DCM, washed with aqueous 5% NaOH solution and dried over $MgSO_4$. The crude material was purified by column chromatography (silica gel, hexanes/EtOAc = 20/1 → 4/1) to give the spiro cyclic methyl ester **17** (12 mg, 0.029 mmol, 48% yield) as a colorless liquid.

$\mathbf{R}_f$ 0.45 (hexanes/EtOAc = 3:1, vanillin)

^{1}H NMR (400 MHz, $CDCl_3$) δ 5.78 – 5.67 (m, 1H), 5.67 (d, *J* = 9.8 Hz, 1H), 5.45 (s, 1H), 5.09 – 4.95 (m, 2H), 4.16 (dd, *J* = 9.1, 7.1 Hz, 1H), 3.59 (s, 3H), 2.88 (ddt, *J* = 13.1, 9.9, 6.6 Hz, 1H), 2.48 – 2.41 (m, 1H), 2.41 (dd, *J* = 14.3, 4.7 Hz, 1H), 2.25 (ddd, *J* = 15.8, 8.6, 4.3 Hz, 1H), 2.15 – 2.08 (m, 2H), 1.87 – 1.71 (m, 3H), 1.37 (s, 3H), 1.29 (s, 3H), 1.12 (s, 3H), 1.11 (s, 3H), 1.05 (d, *J* = 6.6 Hz, 3H), 0.93 (d, *J* = 6.5 Hz, 3H).

^{13}C NMR (101 MHz, $CDCl_3$) δ 198.8, 175.3, 166.7, 146.2, 142.4, 137.9, 116.2, 91.3, 85.7, 70.4, 59.4, 50.8, 41.1, 40.7, 40.3, 35.0, 34.8, 29.6, 28.2, 27.0, 25.1, 24.2, 23.5, 22.7.

FTIR (neat, cm^{-1}) 3455, 2972, 3868, 1713, 1640, 1466, 1436, 1121, 1107, 1052, 990, 912.

HRMS (FAB) *m/z* calcd. for $C_{24}H_{37}O_5^+$ $[M+H]^+$: 405.2642, found: 405.2636.

$[\alpha]_D^{25}$ +55.0 (*c* 0.2, EtOH).

3.3. Synthetic procedures in the cascade dithiol addition/cyclization approach to (+)-garsubellin A

Preparation of 3-methyl-2-oxo-butyronitrile[11]

A round-bottomed flask was charged with isobutyryl chloride (6.3 ml, 60 mmol), TMSCN (7.5 ml, 75 mmol, 1.25 equiv) and ZnI_2 (114 mg, 0.36 mmol, 0.006 equiv). After heating at 120 ˚C for 3 h, the resulting deep red solution was cooled to 25 °C and concentrated in vacuo. After distillation (50 ˚C, 2.5 mbar), 3-methyl-2-oxo-butyronitrile (isobutyryl cyanide, 2.5 g, 25.8 mmol, 43% yield) was obtained as an orange liquid.

Acylation of ketone 6 to diketone 20

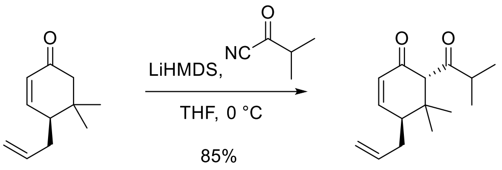

6 **20**

To a solution of enone **6** (1.00 g, 6.09 mmol) in THF (25 mL) was added dropwise a solution of lithium bis(trimethylsilyl)amide (LiHMDS, 6.7 mL, 1.0 M in THF, 6.7 mmol, 1.1 equiv) at −78 ˚C. After stirring the reaction mixture at that temperature for 1 h, a freshly prepared 3-methyl-2-oxo-butyronitrile (1.18 g, 12.18 mmol, 2.0 equiv) was added. The stirring was continued for an additional period of 1 h, after which the reaction mixture was diluted with EtOAc (50 mL) and a saturated aq. NH_4Cl (50 mL), allowed to warm to 25 °C, and extracted twice with EtOAc (50 mL). The combined organic extracts were dried over Na_2SO_4 and concentrated in vacuo. Purification of the residue by flash column chromatography (silica gel, hexanes/EtOAc = 20/1 → 8/1) afforded diketone **20** (1.21 g, 5.17 mmol, 85% yield) as a pale-yellow liquid.

R*f* 0.61 (hexanes/EtOAc = 4:1, vanillin)

^{1}H NMR (400 MHz, $CDCl_3$) δ 6.84 (dd, *J* = 10.3, 2.1 Hz, 1H), 5.94 (dd, *J* = 10.3, 2.7 Hz, 1H), 5.90 – 5.75 (m, 1H), 5.21 – 5.10 (m, 2H), 3.57 (s, 1H), 3.06 (dd, *J* = 11.7, 2.4 Hz, 1H), 2.79 (dp, *J* = 13.9, 6.9 Hz, 1H), 2.50-2.35 (m, 1H), 1.87 (dd, *J* = 21.9, 12.7 Hz, 1H), 1.11 (s, 3H), 1.09 (d, *J* = 7.0 Hz, 3H), 1.06 (d, *J* = 6.8 Hz, 3H), 0.94 (s, 3H).

^{13}C NMR (101 MHz, $CDCl_3$) δ 209.8, 195.1, 153.2, 136.3, 125.8, 117.6, 71.2, 42.8, 41.9, 39.6, 32.5, 25.1, 23.0, 17.5, 17.3.

FTIR (neat, cm^{-1}) 2974, 2936, 1701, 1671, 1570.

HRMS (ESI) *m/z* calcd. for $C_{15}H_{22}O_2Na^+$ $[M+Na]^+$: 257.1513, found: 257.1512.

$[\alpha]_D^{28}$ +156.5 (*c* 1.0, EtOH).

Reductive prenylation of 20 to 21

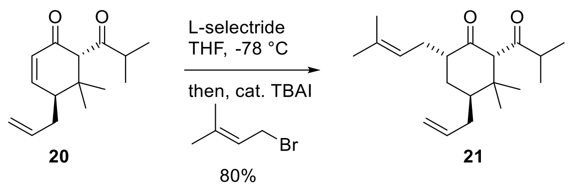

To a solution of enone **20** (313 mg, 1.34 mmol) in THF (2 mL) was added dropwise a solution of L-selectride (1.47 mL, 1.0 M in THF, 1.41 mmol, 1.05 equiv) at −78 ˚C. After stirring the reaction mixture at that temperature for 1 h, tetrabutylammonium iodide (TBAI, 25 mg, 0.067 mmol, 0.05 equiv) and 1-bromo-3-methyl-2-butene (prenyl bromide, 0.23 mL, 2.01 mmol, 1.5 equiv) were added. Then, the reaction mixture was allowed to warm to 0 ˚C over 4 h and diluted with EtOAc (50 mL) and a saturated aq. NH_4Cl (50 mL). The layers were separated and extracted twice with EtOAc (50 mL). The combined organic extracts were dried over Na_2SO_4 and concentrated in vacuo. Purification of the residue by flash column chromatography (silica gel, hexanes/diethyl ether = 40/1 → 10/1) afforded prenylated diketone **21** (326 mg, 1.07 mmol, 80% yield) as a colorless liquid.

$\mathbf{R}_f$ 0.58 (hexanes/EtOAc = 8:1, vanillin)

^{1}H NMR (400 MHz, $CDCl_3$) δ 5.85 – 5.72 (m, 1H), 5.14 – 5.05 (m, 2H), 5.05 – 4.97 (m, 1H), 3.74 (s, 1H), 2.54 (td, J = 13.6, 6.6 Hz, 1H), 2.56 – 2.47 (m, 1H), 2.39 (ddd, J = 10.5, 6.9, 5.6 Hz, 1H), 2.34 – 2.25 (m, 1H), 2.19 – 2.07 (m, 1H), 2.00 (dd, J = 15.0, 7.6 Hz, 1H), 1.91 (ddd, J = 11.6, 10.9, 7.6 Hz, 1H), 1.74 – 1.68 (m, 2H), 1.67 (s, 3H), 1.59 (s, 3H), 1.18 (s, 3H), 1.04 (t, J = 6.7 Hz, 6H), 0.99 (s, 3H).

^{13}C NMR (101 MHz, $CDCl_3$) δ 210.5, 209.3, 137.6, 133.5, 121.4, 116.3, 67.1, 46.6, 43.5, 42.6, 41.9, 32.6, 30.6, 28.0, 25.7, 25.4, 25.2, 18.2, 17.9, 17.1.

FTIR (neat, cm^{-1}) 2970, 2931, 2874, 1726, 1699, 1462, 1388, 912.

HRMS (ESI) *m/z* calcd. for $C_{20}H_{32}O_2Na^+$ $[M+Na]^+$: 327.2296, found: 327.2295.

$[\alpha]_D^{25}$ −61.6 (*c* 1.0, EtOH).

1D nOe analysis to determine the stereochemistry of the C1, C5, and C7 centers

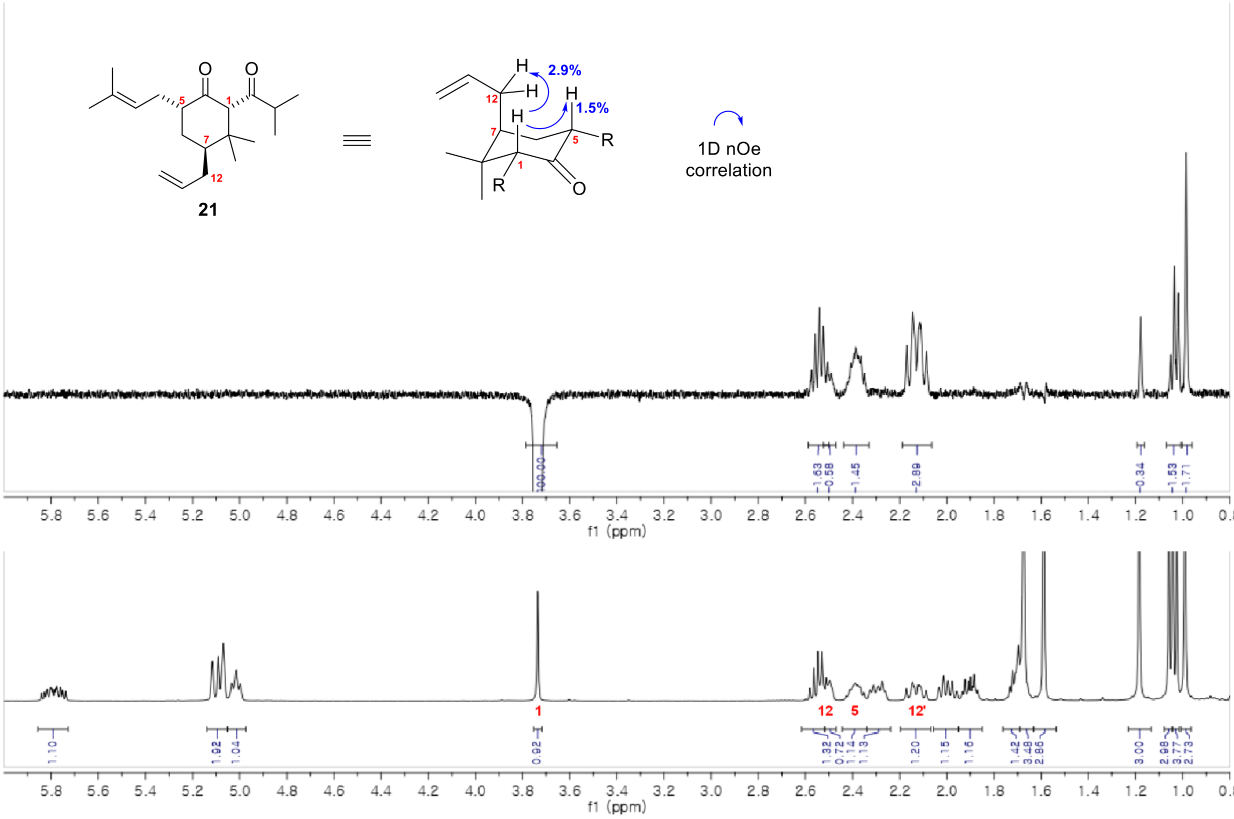

Table S4. Alkynylation of diketone **21**

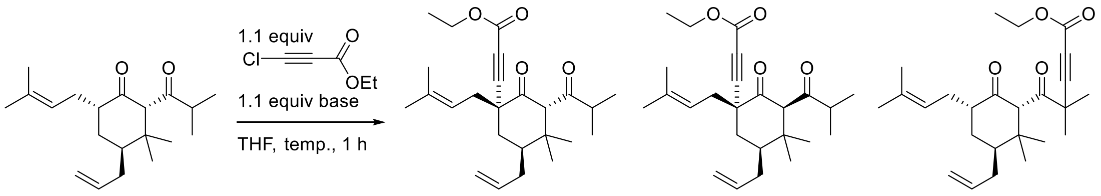

entry	base	reaction temp. (°C)	results[a,b]
1	LDA	–78	**22’** (31%) + **21** recovered (21%)[c]
2	LiTMP	–78	**22’** (63%)
3	KHMDS	–78	**22a** (38%) + **21** recovered (19%)[b]
4	KO*t*Bu	–78	**22a** (59%)[d]
5[e]	KO*t*Bu	–78 → 0	**22b** (58%)

[a]Ethyl 3-chloropropiolate was synthesized using the known method by Jørgensen (ref 5). [b]The structural determination of isomers **22a**, **22b** and **22’** was carried out based on 1D NOESY and HMBC experiments. [c]The starting material (**21**) was recovered as a 1:1 diastereomeric mixture. [d]The α-isomer **22a** was converted to the β-isomer **22b** under basic conditions (K_2CO_3/EtOH). [e]The reaction was performed at –78 °C for 1 h and at 0 °C for additional 6 h.

Preparation of ethyl 3-chloropropiolate[5]

Following the procedure for the preparation of allyl chloropropiolate, the reaction of ethyl propiolate (1.0 ml, 10 mmol) with a freshly prepared *tert*-butyl hypochlorite (1.1 ml, 10 mmol, 1.0 equiv) and *t*-BuOK (118 mg, 1.0 mmol, 0.1 equiv) was carried out to produce ethyl 3-chloropropiolate (543 mg, 4.1 mmol, 41% yield) as a pale-yellow liquid.

R*f* 0.46 (hexanes/EtOAc = 10:1, $KMnO_4$)
^{1}H NMR (499 MHz, $CDCl_3$) δ 4.25 (q, *J* = 7.1 Hz, 2H), 1.32 (t, *J* = 7.1 Hz, 3H).
^{13}C NMR (126 MHz, $CDCl_3$) δ 152.3, 68.0, 62.4, 61.9, 13.9.

Alkynylation of 21 to form 22b

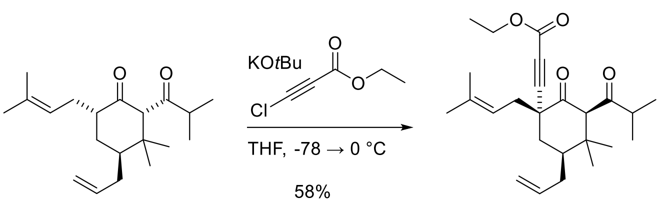

To a solution of the prenylated diketone **21** (100 mg, 0.33 mmol) in THF (6.4 mL) was added dropwise a solution of KO*t*Bu (0.34 mL, 0.34 mmol, 1.0 M solution in THF, 1.05 equiv) at −78 ˚C, and the resulting light-yellow solution was continued to stir at that temperature. Ethyl 3-chloropropiolate (48 mg, 0.36 mmol, 1.1 equiv) was added and the reaction mixture was warmed to 0 ˚C. After being stirred for 6 h, the reaction mixture was diluted with EtOAc (20 mL) and a saturated aq. NH_4Cl (20 mL). The layers were separated and extracted twice with EtOAc (20 mL). The combined organic phases were washed with a saturated aq. NaCl (20 mL), dried over Na_2SO_4 and concentrated in vacuo. Purification of the residue by flash column chromatography (silica gel, hexanes/EtOAc = 40/1 → 8/1) afforded alkynyl diketone **22b** (75 mg, 0.19 mmol, 58% yield) as a pale-yellow liquid.

R*f* 0.46 (hexanes/EtOAc = 8:1, vanillin)
^{1}H NMR (400 MHz, $CDCl_3$) δ 5.83 – 5.71 (m, 1H), 5.17 (t, *J* = 7.4 Hz, 1H), 5.06 (dd, *J* = 13.5, 7.8 Hz, 2H), 4.42 (s, 1H), 4.26 (q, *J* = 7.1 Hz, 2H), 2.51 (dd, *J* = 14.5, 7.2 Hz, 1H), 2.51 – 2.44 (m, 1H), 2.44 – 2.36 (m, 1H), 2.29 (dd, *J* = 14.6, 7.8 Hz, 1H), 2.22 (dd, *J* = 14.1, 3.6 Hz, 1H), 1.99 (ddd, *J* = 13.5, 6.2, 3.1 Hz, 1H), 1.73 (s, 3H), 1.72 – 1.64 (m, 1H), 1.63 (s, 3H), 1.44 – 1.36 (m, 1H), 1.33 (t, *J* = 7.1 Hz, 3H), 1.09 (s, 3H), 1.09 – 1.05 (m, 6H), 0.99 (s, 3H).
^{13}C NMR (101 MHz, $CDCl_3$) δ 209.7, 202.4, 153.2, 136.9, 136.0, 117.8, 116.7, 88.0, 77.9, 66.3, 62.1, 50.4, 44.0, 43.8, 43.1, 39.6, 33.6, 33.0, 26.3, 25.9, 18.1, 17.9, 17.2, 15.4, 14.0.
FTIR (neat, cm^{-1}); 2972, 2933, 2231, 1715, 1368, 1244.
HRMS (ESI) *m/z* calcd. for $C_{25}H_{36}O_4Na^+$ $[M+Na]^+$: 423.2505, found: 423.2506.
$[\boldsymbol{\alpha}]_D^{25}$ +202.6 (*c* 0.7, EtOH).

Alkynylation of 21 to form 22a (kinetic product)

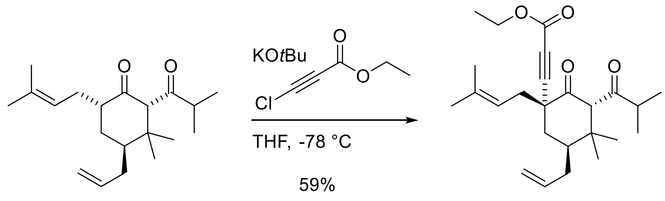

21 **22a**

Following the procedure for diketone alkynoate **22b**, prenylated diketone **21** (120 mg, 0.39 mmol) was reacted with KO*t*Bu (0.41 mL, 1.0 M solution in THF, 0.41 mmol, 1.05 equiv) and ethyl 3-chloropropiolate (57 mg, 0.43 mmol, 1.1 equiv) at −78 ˚C for 1 h and quenched at −78 ˚C to furnish the alkynylated diketone **22a** (92 mg, 0.23 mmol, 59% yield) as a pale-yellow oil.

R*f* 0.47 (hexanes/EtOAc = 8:1, vanillin)

^{1}H NMR (400 MHz, $CDCl_3$) δ 5.83 (dddd, *J* = 18.5, 15.7, 10.4, 6.0 Hz, 1H), 5.16 – 5.09 (m, 1H), 5.04 (dd, *J* = 8.6, 7.4 Hz, 2H), 4.19 (q, *J* = 7.1 Hz, 2H), 3.57 (s, 1H), 3.03 (ddt, *J* = 14.2, 10.7, 3.5 Hz, 1H), 2.81 (dt, *J* = 13.7, 6.8 Hz, 1H), 2.53 (dd, *J* = 14.4, 7.7 Hz, 1H), 2.48 – 2.36 (m, 2H), 2.15 (dd, *J* = 14.0, 3.8 Hz, 1H), 1.72 (s, 3H), 1.71 – 1.63 (m, 1H), 1.62 (s, 3H), 1.44 (t, *J* = 14.8 Hz, 1H), 1.28 (t, *J* = 7.1 Hz, 3H), 1.12 (d, *J* = 7.1 Hz, 3H), 1.09 (s, 3H), 1.05 (d, *J* = 6.6 Hz, 3H), 0.72 (s, 3H).

^{13}C NMR (101 MHz, $CDCl_3$) δ 206.9, 201.4, 153.0, 137.4, 136.1, 117.9, 116.3, 87.6, 78.0, 74.5, 61.9, 49.5, 40.1, 39.6, 37.9, 36.2, 35.6, 33.7, 25.9, 25.7, 22.4, 19.5, 18.1, 17.5, 13.9.

FTIR (neat, cm^{-1}); 2972, 2933, 2232, 1719, 1435, 1305, 1247.

HRMS (ESI) *m/z* calcd. for $C_{25}H_{36}O_4Na^+$ $[M+Na]^+$: 423.2507, found: 423.2506.

$[\alpha]_D^{23}$ −62.5 (*c* 0.8, EtOH).

Alkynylation of 21 to form 22’ (regioisomer)

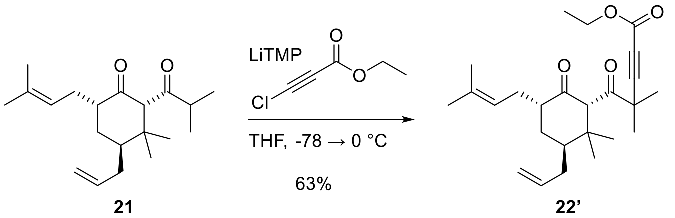

21 **22’**

Following the procedure for diketone alkynoate **22b**, prenylated diketone **21** (96 mg, 0.32 mmol) was reacted with lithium 2,2,6,6-tetramethylpiperidide (LiTMP, 0.34 mL, 1.0 M solution in THF, 0.34 mmol, 1.05 equiv) and ethyl 3-chloropropiolate (47 mg, 0.35 mmol, 1.1 equiv) and at −78 ˚C for 1 h and quenched at −78 ˚C to furnish the alkynyl diketone **22’** (79 mg, 0.20 mmol, 63% yield) as a pale-yellow oil.

R*f* 0.45 (hexanes/EtOAc = 8:1, vanillin)

^{1}H NMR (400 MHz, $CDCl_3$) δ 5.80 (dddd, *J* = 17.0, 9.9, 8.9, 5.3 Hz, 1H), 5.13 (dd, *J* = 23.2, 5.5 Hz, 2H), 5.07 – 4.98 (m, 1H), 4.46 (s, 1H), 4.22 (q, *J* = 7.1 Hz, 2H), 2.59 (td, *J* = 13.4, 6.8 Hz, 2H), 2.43 – 2.25 (m, 2H), 2.04 (ddd, *J* = 13.9,

6.3, 2.2 Hz, 1H), 2.01 – 1.91 (m, 1H), 1.67 (s, 3H), 1.62 (dd, J = 13.2, 4.9 Hz, 1H), 1.59 (s, 3H), 1.47 (dt, J = 11.0, 4.4 Hz, 1H), 1.41 (s, 3H), 1.33 (s, 3H), 1.30 (t, J = 7.1 Hz, 3H), 1.30 – 1.25 (m, 1H), 1.22 (s, 2H), 1.06 (s, 3H).

^{13}C NMR (101 MHz, $CDCl_3$) δ 208.4, 204.1, 153.2, 137.5, 133.2, 121.3, 116.4, 90.5, 64.2, 62.0, 45.6, 45.0, 44.8, 43.2, 32.4, 31.1, 27.3, 25.7, 25.3, 25.0, 24.4, 17.8, 14.0.

FTIR (neat, cm^{-1}); 2975, 2933, 2225, 1736, 1712, 1454, 1338, 1267, 1212, 1034.

HRMS (ESI) m/z calcd. for $C_{25}H_{36}O_4Na^+$ $[M+Na]^+$: 423.2505, found: 423.2506.

Epimerization of diketone 22a to 22b

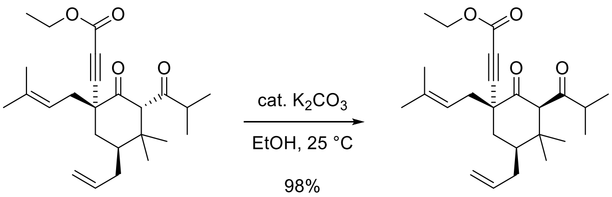

22a **22b**

To a solution of the α-butyrylated diketone **22a** (180 mg, 0.45 mmol) in EtOH (9 mL) was added potassium carbonate (3 mg, 0.023 mmol, 0.05 equiv) at 25 ˚C. The reaction mixture was left to stir at that temperature for 12 h and poured into a saturated aqueous solution of NH_4Cl. After extraction twice with EtOAc, the combined organic phases were washed with a saturated aqueous solution of NaCl, dried over Na_2SO_4 and concentrated in vacuo. Purification of the residue by flash column chromatography (silica gel, hexanes/EtOAc = 40/1 → 8/1) of the β-butyrylated diketone **22b** (176 mg, 0.44 mmol, 98% yield) as a pale-yellow liquid.

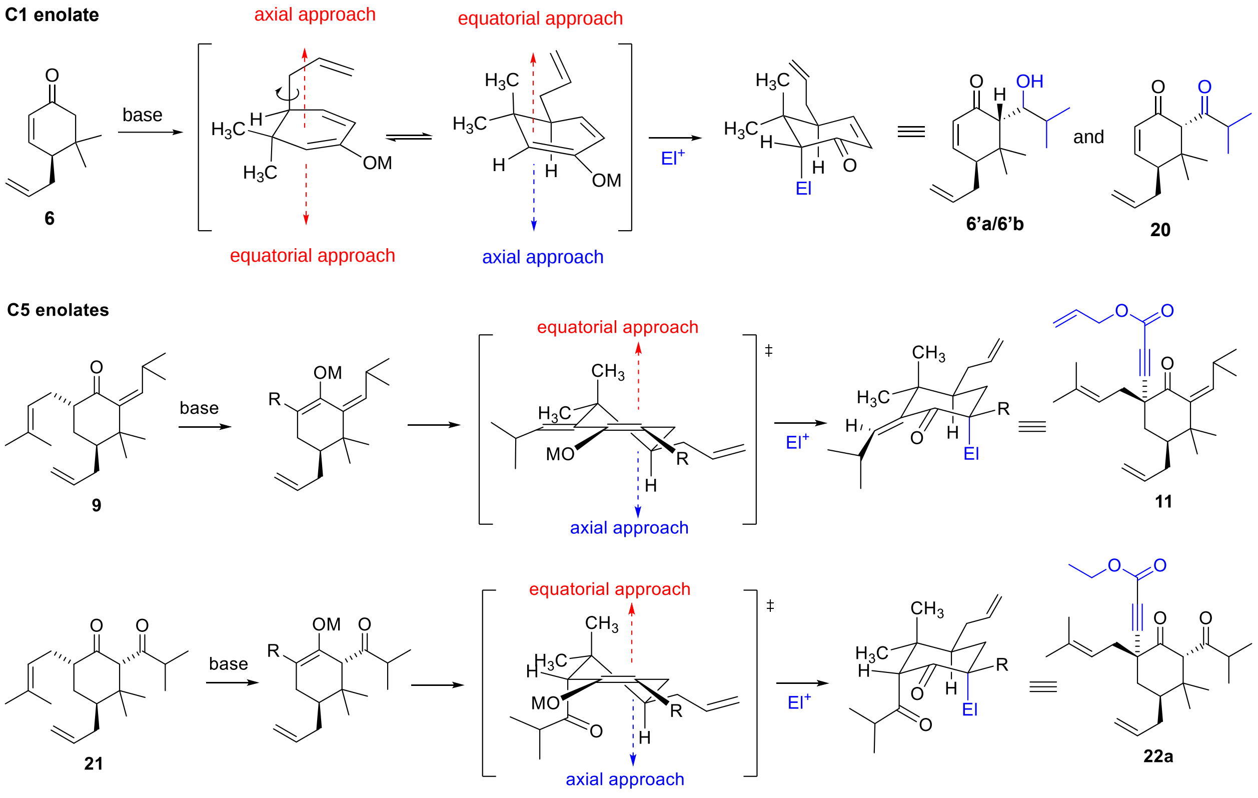

Scheme S4. Rationale for the diastereoselectivity in the reactions of the enolate

1D nOe analysis to determine the stereochemistry of C1

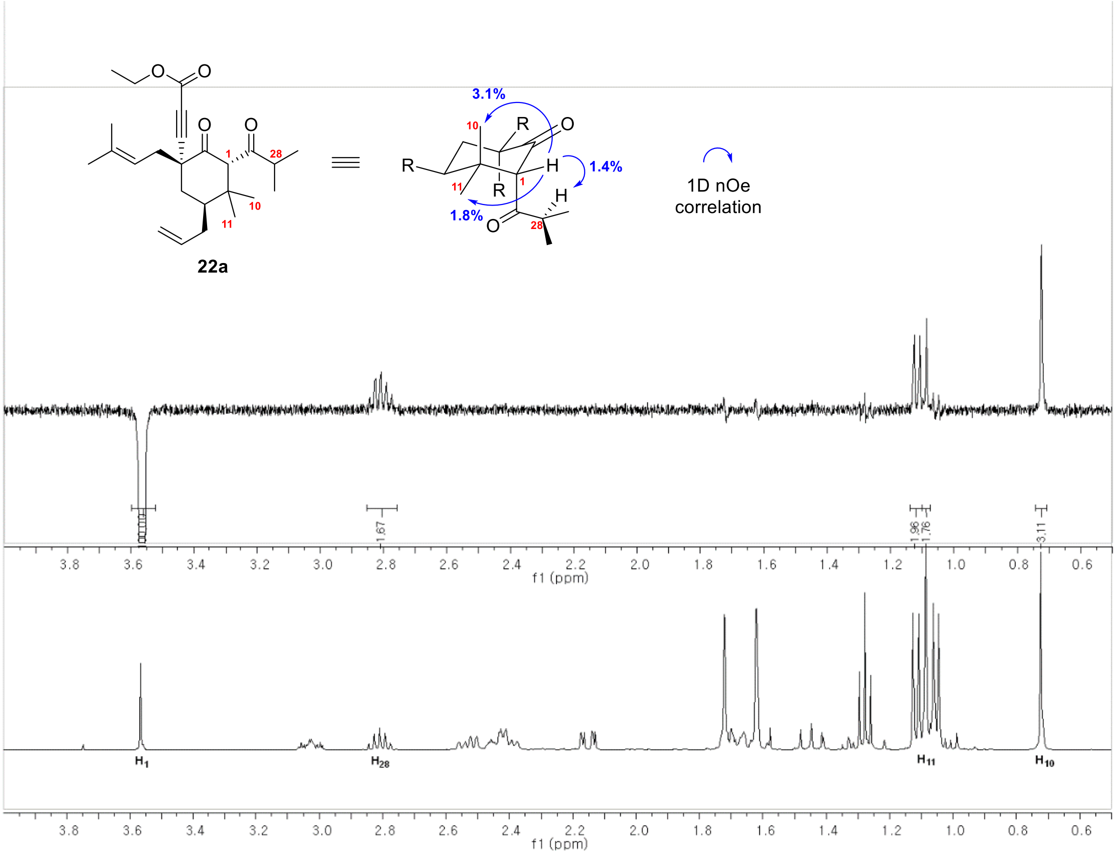

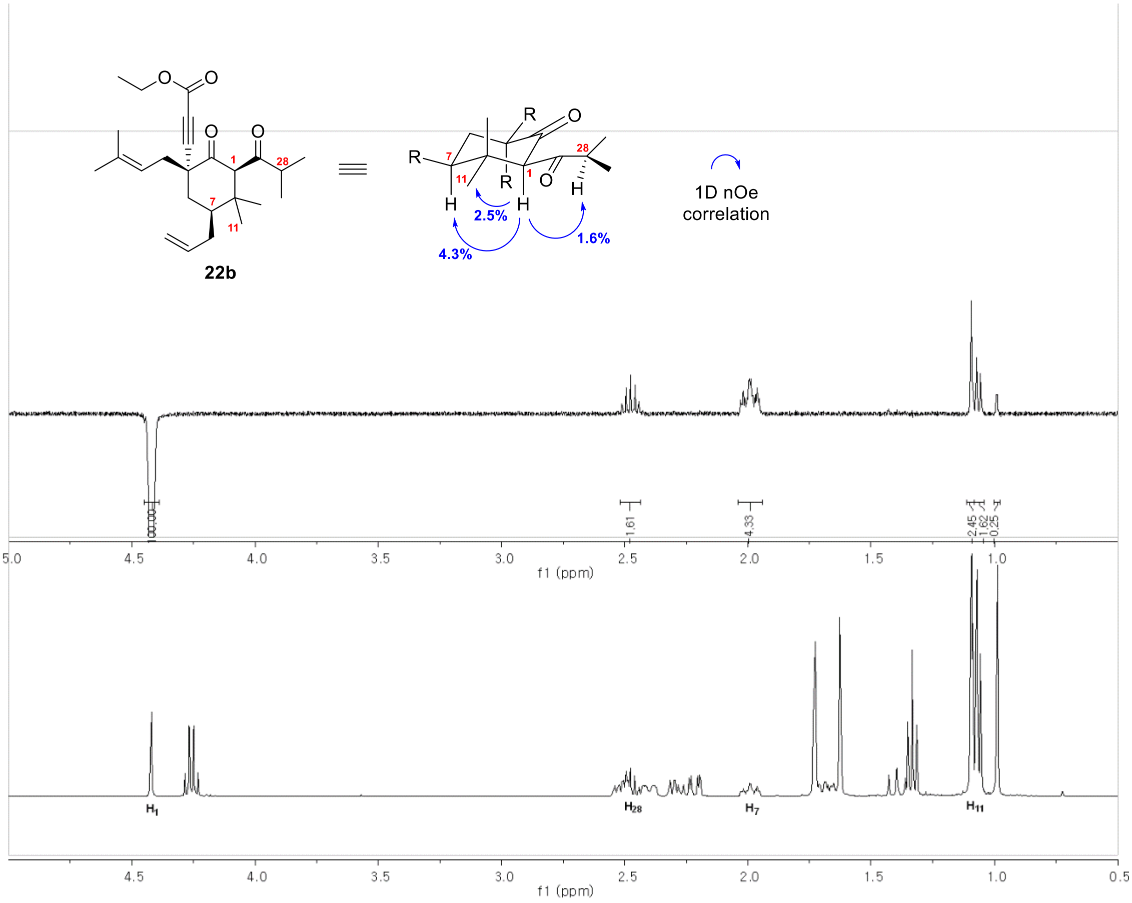

2D NMR analysis was carried out to determine the site of alkynylation

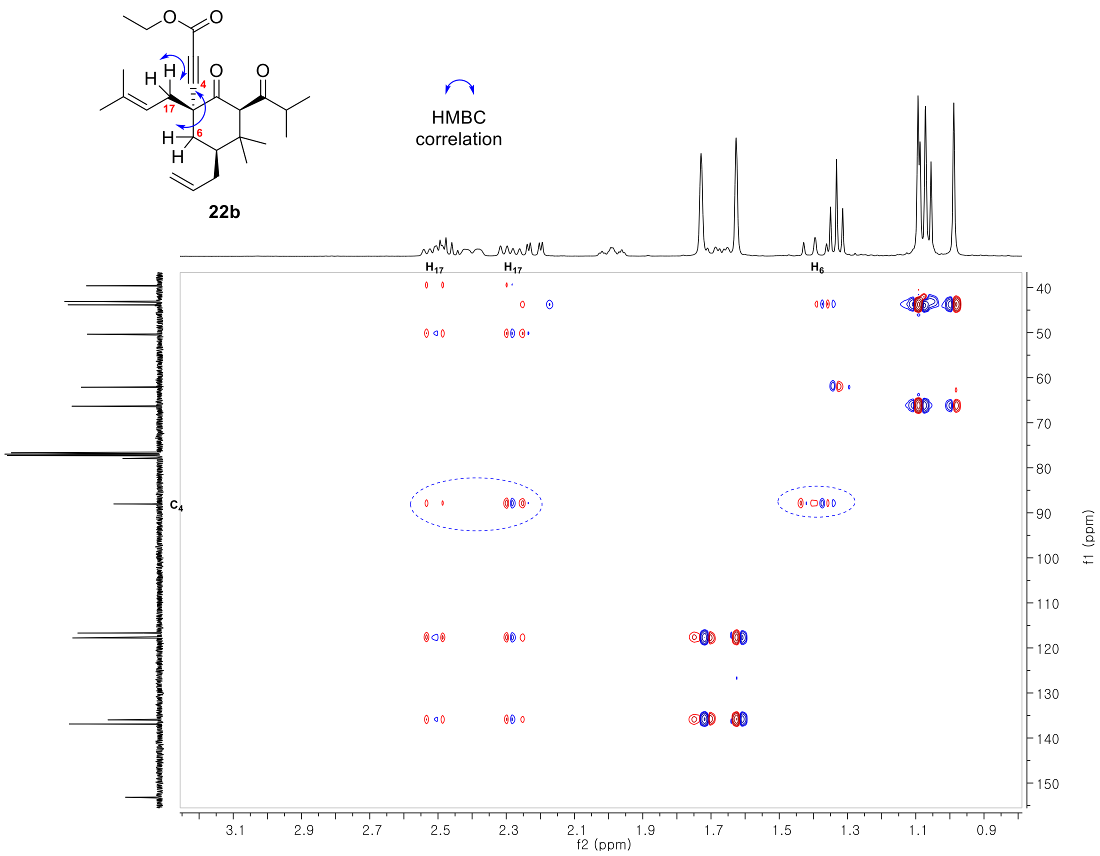

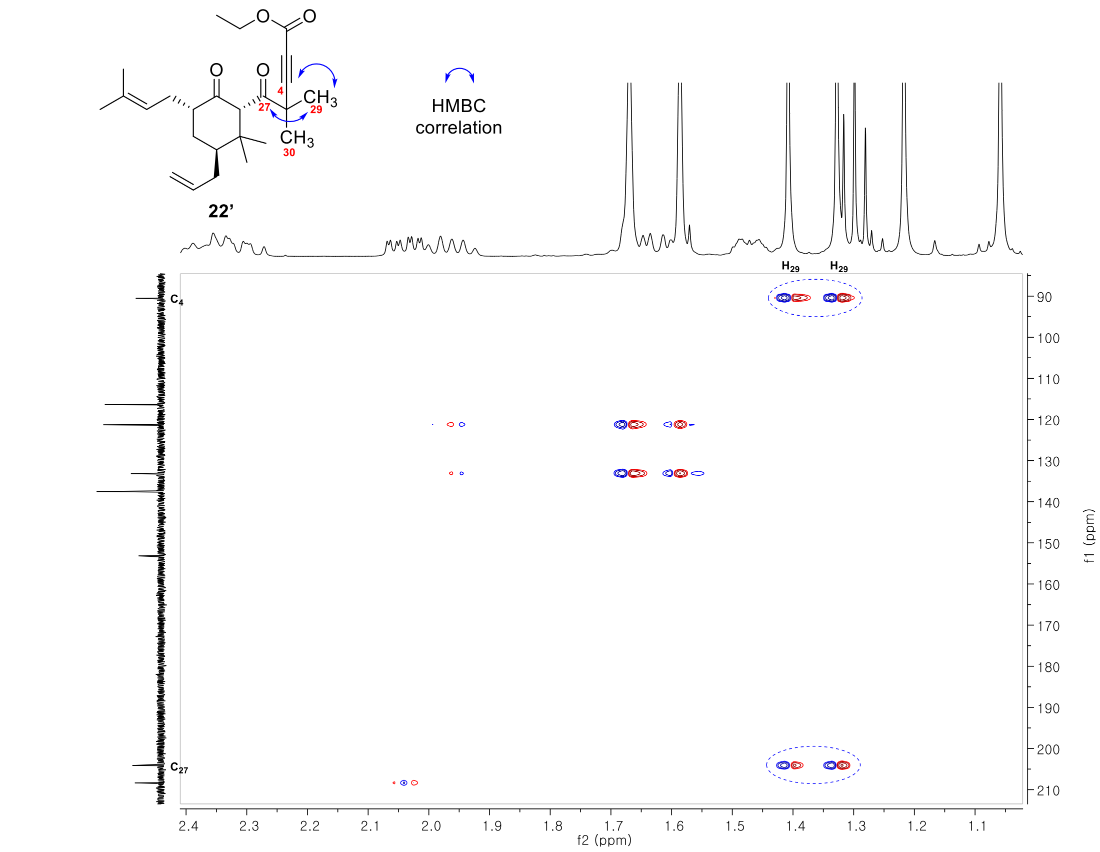

Table S5. Reduction of diketone alkynoate **22b**

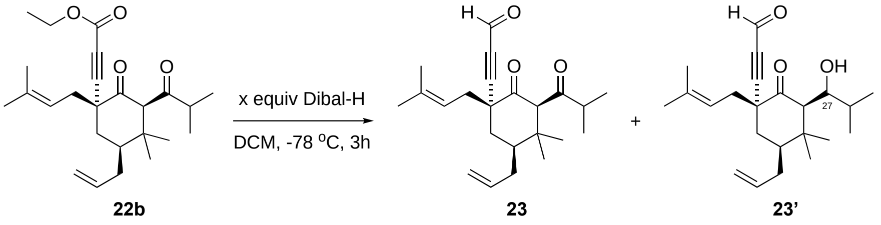

entry	x (equivalent)	results[a,b]
1	1.1	**23** (21%) + **22b** recovered (59%)
2	2.1	**23** (85%) + **23’** (5%)
3	2.5	**23** (33%) + **23’** (48%)

[a]The C27 alcohol **23’** was obtained as a single isomer with undetermined stereochemistry and was recycled to aldehyde **23** through oxidation (DMP, DCM). [b]Under the conditions examined, a propargylic alcohol from over-reduction of the aldehyde was not detected.

Reduction of ethyl ester 22b to aldehyde 23

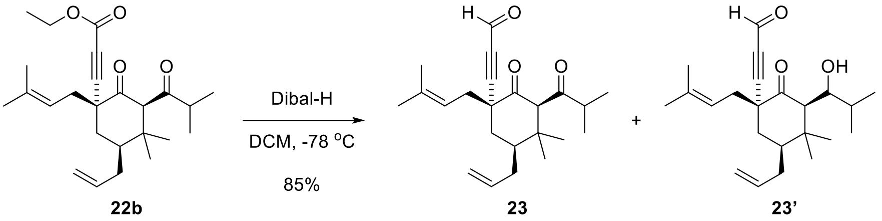

To a solution of ethyl ester **22b** (269 mg, 0.70 mmol) in DCM (14 mL) was added dropwise a solution of diisobutylaluminum hydride (1.50 mL, 1.0 M in DCM, 1.46 mmol, 2.1 equiv) at –78 ˚C. After stirring for 2 h, excess reagents were quenched by adding methanol (1 mL) and saturated aq. sodium potassium tartrate (10 mL). The biphasic mixture was allowed to warm to 25 °C with stirring over a period of 30 min. The mixture was extracted with EtOAc (50 mL), and the combined organic phases were washed with a saturated aq. NaCl (50 mL), dried over Na_2SO_4 and concentrated in vacuo. Purification of the residue by flash column chromatography (silica gel, hexanes/EtOAc = 20/1 → 8/1) gave aldehyde **23** (212 mg, 0.60 mmol, 85% yield) and alcohol **23’** (12 mg, 0.034 mmol, 5% yield) as a colorless liquid.

Diketone ynal 23

R*f* 0.36 (hexanes/EtOAc = 8:1, vanillin)

^{1}H NMR (400 MHz, $CDCl_3$) δ 9.27 (s, 1H), 5.82 – 5.70 (m, 1H), 5.17 (t, *J* = 7.5 Hz, 1H), 5.07 (ddd, *J* = 14.9, 10.3, 3.3 Hz, 2H), 4.33 (s, 1H), 2.55 (dd, *J* = 14.6, 7.2 Hz, 1H), 2.46 (dt, *J* = 13.7, 6.9 Hz, 1H), 2.40 (dd, *J* = 9.2, 2.1 Hz, 1H), 2.32 (dd, *J* = 14.6, 7.8 Hz, 1H), 2.24 (dd, *J* = 14.1, 3.6 Hz, 1H), 2.02 – 1.91 (m, 1H), 1.73 (s, *J* = 6.2 Hz, 3H), 1.68 (dd, *J* = 9.4, 4.5 Hz, 1H), 1.63 (s, 3H), 1.44 (dd, *J* = 13.7, 13.1 Hz, 1H), 1.10 (s, 3H), 1.06 (t, *J* = 7.0 Hz, 6H), 1.00 (s, 3H).

^{13}C NMR (101 MHz, $CDCl_3$) δ 209.5, 201.9, 176.1, 136.8, 136.2, 117.6, 116.9, 97.1, 85.0, 66.7, 50.8, 44.0, 44.0, 43.1, 39.7, 33.8, 33.0, 26.3, 25.9, 18.1, 17.9, 17.3, 15.4.

FTIR (neat, cm^{-1}); 2971, 2931, 2873, 2201, 1733, 1715, 1669, 1466, 1388, 1113, 915.

HRMS (ESI) *m/z* calcd. for $C_{23}H_{32}O_3Na^+$ $[M+Na]^+$: 379.2253, found: 379.2244.

$[\boldsymbol{a}]_D^{22}$ +186.2 (*c* 1.0, EtOH).

Alcohol 23'

R*f* 0.33 (hexanes/EtOAc = 8:1, vanillin)

^{1}H NMR (400 MHz, $CDCl_3$) δ 9.23 (s, 1H), 5.83 – 5.69 (m, 2H), 5.16 (ddd, *J* = 8.1, 2.7, 1.3 Hz, 1H), 5.12 – 4.98 (m, 3H), 3.66 (d, *J* = 11.5 Hz, 1H), 3.47 (dd, *J* = 11.0, 9.0 Hz, 1H), 3.12 (s, 1H), 2.50 (dd, *J* = 14.7, 7.0 Hz, 1H), 2.46 – 2.38 (m, 1H), 2.32 – 2.22 (m, 2H), 2.01 – 1.90 (m, 1H), 1.74 (s, 3H), 1.64 (s, 3H), 1.46 – 1.36 (m, 1H), 1.20 (s, 3H), 1.00 (d, *J* = 6.6 Hz, 3H), 0.95 (s, 3H), 0.85 (d, *J* = 6.7 Hz, 3H).

^{13}C NMR (101 MHz, $CDCl_3$) δ 211.0, 176.0, 137.1, 135.9, 117.8, 116.7, 97.6, 85.0, 76.1, 57.0, 51.8, 45.3, 44.0, 41.1, 34.2, 33.8, 33.5, 26.4, 25.9, 19.9, 19.8, 18.2, 16.6.

Table S6. Dithiol double conjugated addition-intramolecular aldol cyclization

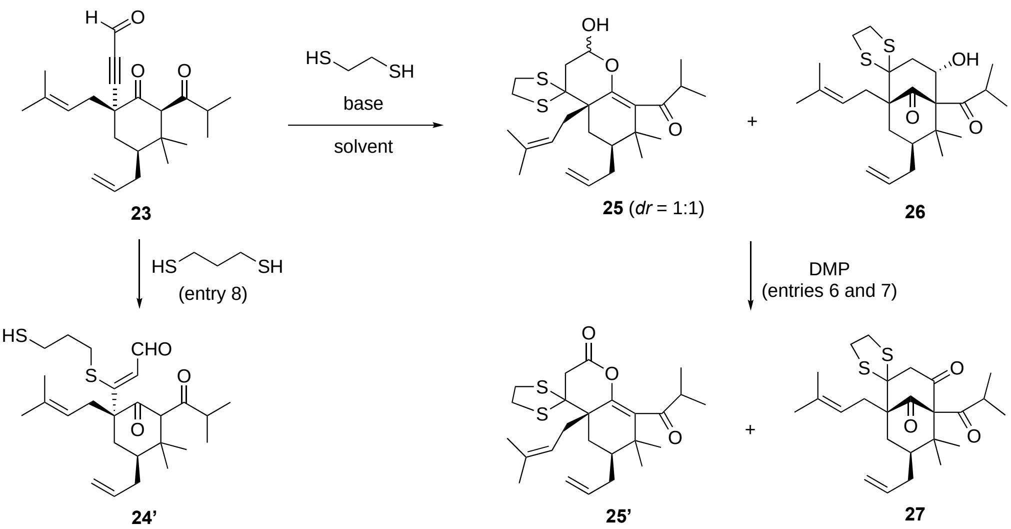

entry	1,2-ethanedithiol (equiv)	conditions	results[a]
1	1.1	NaOMe (1.1 equiv), DCM/MeOH, –10 → 0 °C, 12 h	**26** (19%) + **25** (23%)
2	2.1	NaOMe (1.1 equiv), DCM/MeOH, –10 → 0 °C, 12 h	**26** (36%) + **25** (41%)
3	2.1	NaOMe (1.1 equiv), THF, –10 → 25 °C, 6 h	complex mixture
4	2.1	DIPEA (1.1 equiv), DCM, –10 → 25 °C, 24 h	**26** (10%) + **25** (4%)
5	2.1	TMG (1.1 equiv), DCM, –10 → 0 °C, 12 h	**26** (53%) + **25** (26%)
6	2.1	TMG (1.1 equiv), DCM, –10 → 0 °C, 12 h, then 2.5 equiv DMP	**27** (45%) + **25'** (17%)
7	2.1	TMG (2.1 equiv), DCM, –10 → 0 °C, 12 h, then 2.5 equiv DMP	**27** (29%) + **25'** (10%)
8[b]	1,3-propanedithiol (1.1 equiv)	NaOMe (1.1 equiv), DCM/MeOH, –10 → 0 °C, 24 h	**24'**

[a] Isolated yield. [b] Aldehyde **24'** (^{1}H NMR δ 9.5 ppm) has not been fully characterized. DMP = Dess–Martin periodinane.

Double conjugate addition of 1,2-ethanedithiol to ynal 23 to form alcohol 26 and lactol 25

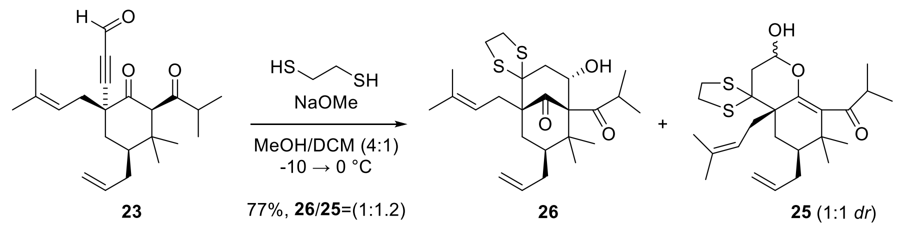

The reaction was carried out using a modified literature procedure.[12] To a solution of aldehyde **23** (48 mg, 0.135 mmol) and 1,2-ethanedithiol (0.017 mL, 0.284 mmol, 2.1 equiv) in MeOH-DCM (4:1, 6.8 mL, 0.02 M) stirred at −10 ˚C was added a solution of sodium methoxide (0.3 mL, 0.5 M in MeOH, 0.149 mmol, 1.1 equiv). The reaction was allowed to warm to 0 ˚C overnight, poured into a biphasic mixture of saturated aq. NH_4Cl (20 mL) and EtOAc (20 mL). The layers were separated and extracted twice with EtOAc (20 mL). The combined organic phases were dried over Na_2SO_4 and concentrated in vacuo. Purification of the residue by flash column chromatography (silica gel, hexanes/EtOAc = 20/1

→ 4/1) afforded dithiolane alcohol **26** (22 mg, 0.049 mmol, 36% yield) and lactol **25** (25 mg, 0.055 mmol, 41% yield, *dr* 1:1) both as pale-yellow liquids.

Dithiolane alcohol 26

R*f* 0.46 (hexanes/EtOAc = 4:1, vanillin)

^{1}H NMR (400 MHz, $CDCl_3$) δ 5.78 (dddd, *J* = 16.2, 10.4, 8.4, 5.6 Hz, 1H), 5.29 – 5.21 (m, 1H), 5.08 – 5.00 (m, 2H), 4.29 (ddd, *J* = 11.1, 6.7, 1.9 Hz, 1H), 4.03 (d, *J* = 2.5 Hz, 1H), 3.32 (ddt, *J* = 17.9, 10.6, 3.9 Hz, 2H), 3.20 (ddd, *J* = 13.1, 6.9, 3.3 Hz, 2H), 3.14 – 3.06 (m, 1H), 2.97 (dd, *J* = 14.9, 11.2 Hz, 1H), 2.82 (dd, *J* = 14.1, 4.3 Hz, 1H), 2.64 (dd, *J* = 15.0, 6.8 Hz, 1H), 2.60 – 2.49 (m, 1H), 2.44 – 2.41 (m, 1H), 2.37 (dd, *J* = 14.0, 10.5 Hz, 1H), 2.14 (dd, *J* = 15.6, 5.9 Hz, 1H), 1.83 (dd, *J* = 15.6, 12.9 Hz, 1H), 1.74 (s, 3H), 1.63 (s, 3H), 1.62 – 1.55 (m, 1H), 1.24 (d, *J* = 6.4 Hz, 3H), 1.18 (s, 3H), 1.12 (d, *J* = 6.5 Hz, 3H), 0.88 (s, 3H).

^{13}C NMR (101 MHz, $CDCl_3$) δ 223.1, 210.1, 137.1, 135.1, 120.6, 116.3, 76.0, 75.6, 74.7, 59.0, 50.8, 46.1, 42.0, 41.0, 40.2, 39.3, 38.0, 35.8, 31.3, 26.0, 25.7, 21.7, 20.6, 19.0, 18.2.

FTIR (neat, cm^{-1}); 3513, 2971, 2927, 1705, 1676, 1448, 1376, 1080, 913.

HRMS (ESI) *m/z* calcd. for $C_{25}H_{38}O_3S_2Na^+$ $[M+Na]^+$: 473.2157, found: 473.2155.

$[\boldsymbol{a}]_D^{22}$ +52.6 (*c* 0.7, EtOH).

Dithiolane lactol 25

R*f* 0.34 (hexanes/EtOAc = 4:1, vanillin)

Partial characterization of major peak

^{1}H NMR (400 MHz, $CDCl_3$) δ 5.83 – 5.70 (m, 1H), 5.31 – 5.25 (m, 1H), 5.04 (dt, *J* = 22.9, 4.8 Hz, 2H), 5.01 – 4.90 (m, 1H), 3.37 – 3.26 (m, 2H), 3.26 – 3.15 (m, 2H), 2.78 (dt, *J* = 13.9, 6.9 Hz, 1H), 2.62 (dd, *J* = 14.0, 9.4 Hz, 1H), 2.38 (dd, *J* = 14.1, 2.7 Hz, 1H), 2.30 (ddd, *J* = 15.8, 8.9, 2.0 Hz, 3H), 1.71 (s, 3H), 1.64 (s, 3H), 1.58 – 1.48 (m, 2H), 1.23 – 1.19 (m, 2H), 1.17 (d, *J* = 7.2 Hz, 3H), 1.11 (d, *J* = 6.5 Hz, 3H), 1.08 (s, 3H), 0.98 (s, 3H).

^{13}C NMR (126 MHz, $CDCl_3$) δ 213.3, 148.0, 138.2, 137.6, 134.6, 121.1, 115.8, 94.0, 74.0, 48.4, 45.3, 42.5, 41.5, 39.8, 38.9, 37.4, 34.9, 31.0, 29.7, 26.0, 26.0, 21.6, 18.6, 18.2, 16.9.

FTIR (neat, cm^{-1}); 3425, 2970, 2928, 1677, 1642, 1441, 1379, 1086, 1046 909.

HRMS (ESI) *m/z* calcd. for $C_{25}H_{38}O_3S_2Na^+$ $[M+Na]^+$: 473.2158, found: 473.2155.

$[\boldsymbol{a}]_D^{22}$ +35.1 (*c* 1.0, EtOH).

1D nOe analysis to determine the stereochemistry of C2.

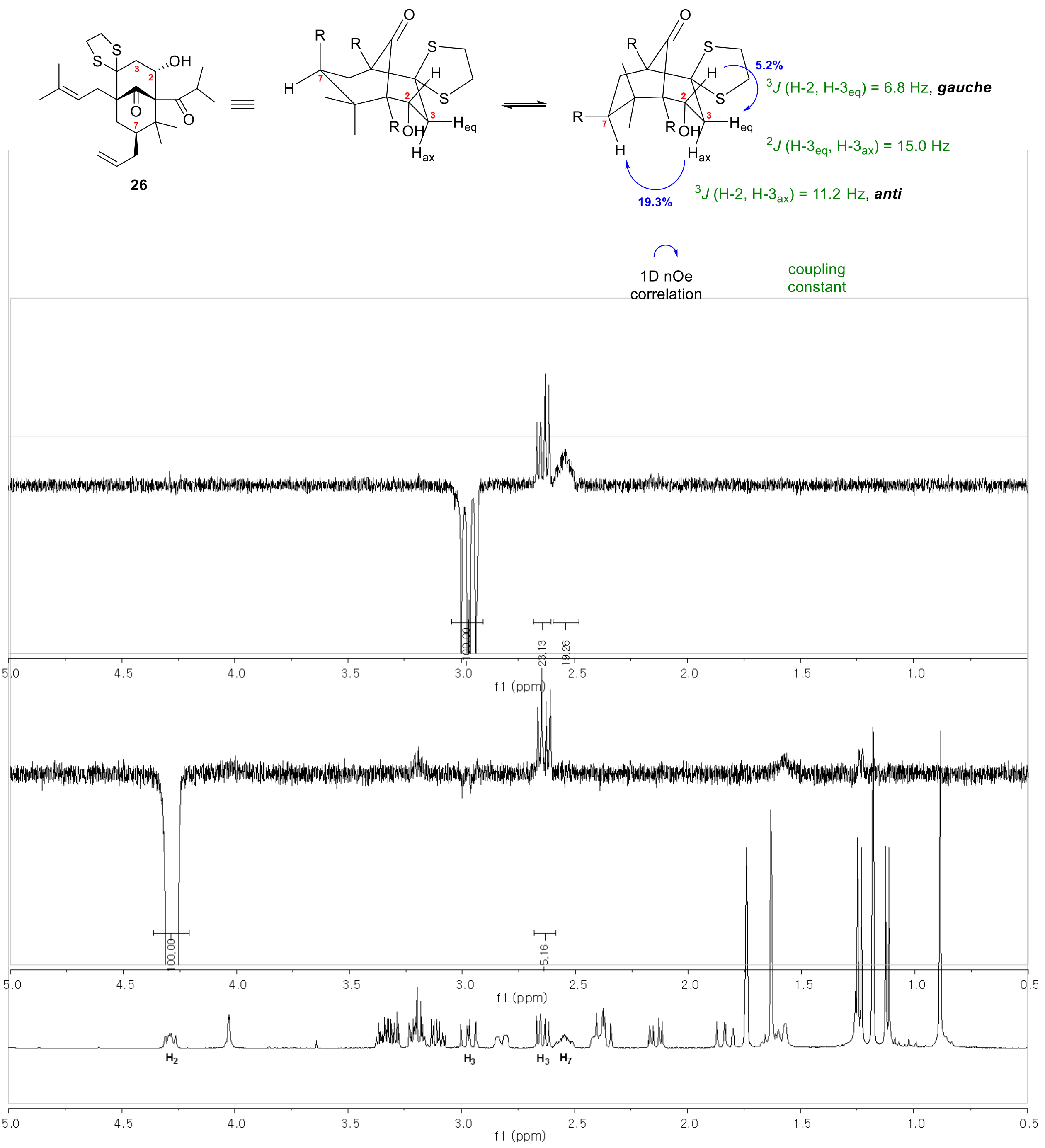

Oxidation of lactol 25 to lactone 25'

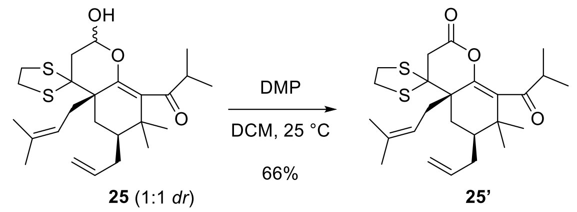

To a solution of dithiolane lactol **25** (30 mg, 0.067 mmol) in DCM (2.2 mL) was added Dess–Martin periodinane (DMP, 37 mg, 0.087 mmol, 1.3 equiv) in one portion. The reaction mixture was stirred at 25 °C for 1 h until complete consumption of starting material as observed by TLC. Slightly excess reagents were quenched with saturated aq. $NaHCO_3$ (10 mL) and saturated aq. $Na_2S_2O_3$ (10 mL). The mixture was extracted with EtOAc (20 mL), and the combined organic phases were dried over Na_2SO_4 and concentrated in vacuo. Purification of the residue by flash column chromatography (silica gel, hexanes/EtOAc = 20/1 → 8/1) afforded lactone **25'** (20 mg, 0.044 mmol, 66% yield) as a pale-yellow liquid.

R*f* 0.47 (hexanes/EtOAc = 4:1, vanillin)

^{1}H NMR (400 MHz, $CDCl_3$) δ 5.83 – 5.70 (m, 1H), 5.13 – 4.98 (m, 3H), 3.49 (d, *J* = 19.5 Hz, 1H), 3.52 – 3.47 (m, 1H), 3.39 – 3.34 (m, 1H), 3.35 (d, *J* = 19.5 Hz, 1H), 3.23 (td, *J* = 10.5, 3.9 Hz, 1H), 3.19 – 3.11 (m, 1H), 2.88 (dp, *J* = 13.9, 6.9 Hz, 1H), 2.40 (dd, *J* = 15.1, 4.5 Hz, 1H), 2.33 (dd, *J* = 13.7, 10.1 Hz, 2H), 2.29 – 2.22 (m, 1H), 2.22 – 2.15 (m, 1H), 1.72 (s, 3H), 1.61 (s, 3H), 1.56 (dd, *J* = 11.5, 7.7 Hz, 1H), 1.36 (dd, *J* = 14.8, 13.4 Hz, 1H), 1.19 (d, *J* = 6.8 Hz, 3H), 1.17 (d, *J* = 7.2 Hz, 3H), 1.09 (s, 3H), 1.00 (s, 3H).

^{13}C NMR (101 MHz, $CDCl_3$) δ 211.0, 164.2, 147.6, 137.7, 136.2, 133.5, 119.3, 115.9, 72.3, 49.6, 44.2, 42.4, 41.9, 41.6, 38.4, 37.7, 36.1, 34.6, 31.2, 25.9, 25.8, 21.8, 18.9, 18.1, 17.7.

FTIR (neat, cm^{-1}); 2973, 2928, 1762, 1684, 1456, 1194, 1049.

HRMS (ESI) *m/z* calcd. for $C_{25}H_{36}O_3S_2Na^+$ $[M+Na]^+$: 471.1997, found: 471.1998.

$[\alpha]_D^{24}$ −12.6 (*c* 1.0, EtOH).

Oxidation of alcohol 26 to triketone 27

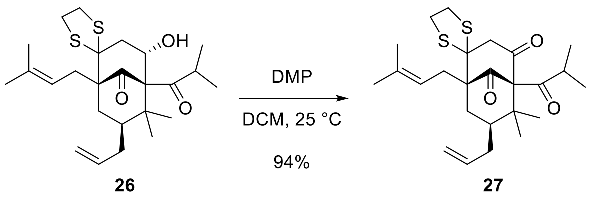

Following the procedure for lactone **25'**, alcohol **26** (18 mg, 0.040 mmol) was reacted with Dess–Martin periodinane (DMP, 20 mg, 0.052 mmol, 1.3 equiv) in DCM (2 mL) to obtain triketone **27** (17 mg, 0.038 mmol, 94% yield) as pale-yellow oil.

R*f* 0.52 (hexanes/EtOAc = 4:1, vanillin)

^{1}H NMR (400 MHz, $CDCl_3$) δ 5.68 (dddd, J = 16.1, 10.1, 8.2, 5.8 Hz, 1H), 5.40 – 5.33 (m, 1H), 5.02 (t, J = 12.5 Hz, 2H), 3.56 – 3.45 (m, 2H), 3.48 – 3.42 (m, 2H), 3.33 – 3.15 (m, 2H), 2.87 (dd, J = 14.0, 4.2 Hz, 1H), 2.60 (dd, J = 14.1, 10.3 Hz, 1H), 2.41 – 2.33 (m, 1H), 2.36 – 2.29 (m, 1H), 2.14 (dd, J = 15.7, 6.3 Hz, 1H), 1.97 (dd, J = 15.6, 12.1 Hz, 1H), 1.74 (s, 3H), 1.71 – 1.64 (m, 1H), 1.63 (s, 3H), 1.61 – 1.52 (m, 1H), 1.17 (d, J = 7.4 Hz, 3H), 1.16 (s, 3H), 1.05 (d, J = 6.5 Hz, 3H), 0.95 (s, 3H).

^{13}C NMR (101 MHz, $CDCl_3$) δ 208.7, 207.2, 204.5, 136.5, 135.5, 120.3, 116.8, 86.9, 72.1, 60.5, 58.6, 50.2, 44.1, 43.2, 40.8, 39.0, 35.5, 30.6, 25.9, 22.3, 21.2, 20.7, 18.1, 17.0.

FTIR (neat, cm^{-1}) 2972, 2928, 1731, 1687, 1641, 1444, 1375, 1243, 1143, 915.

HRMS (ESI) *m/z* calcd. for $C_{25}H_{36}O_3S_2Na^+$ $[M+Na]^+$: 471.1995, found: 471.1998.

$[\boldsymbol{\alpha}]_D^{25}$ −6.2 (*c* 0.9, EtOH).

One-pot conjugate addition, aldol cyclization and oxidation to form 27

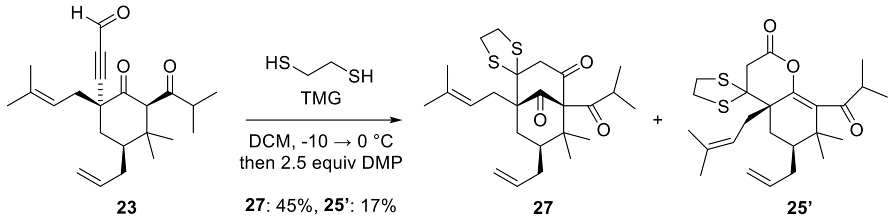

To a stirred solution of aldehyde **23** (44 mg, 0.123 mmol) and 1,2-ethanedithiol (0.022mL, 0.258 mmol, 2.1 equiv) in DCM (6.8 mL) at −10 ˚C was added 1,1,3,3-tetramethylguanidine (TMG, 0.016 mL, 0.129 mmol, 1.05 equiv) via a microsyringe. The reaction was allowed to warm to 0 ˚C and stirred for 12 h, at which time monitoring with TLC indicated formation of aldol **26** and lactol **25** adducts. After Dess–Martin periodinane (132 mg, 0.31 mmol, 2.5 equiv) was added in one portion, the reaction mixture was allowed to warm to 25 ˚C and further stirred for 1 h. Excess reagents were quenched by addition of saturated aq. $NaHCO_3$(10 mL) and $Na_2S_2O_3$(10 mL). The mixture was extracted twice with EtOAc (20 mL). The combined organic phases were dried over Na_2SO_4 and concentrated in vacuo. Purification of the residue by flash column chromatography (silica gel, hexanes/EtOAc = 20/1 → 4/1) to afford triketone (25 mg, 0.055 mmol, 45% yield) and lactone (9.4 mg, 0.021 mmol, 17% yield) both as pale-yellow liquids.

Table S7. Isomerization of dithiolane lactol **25**

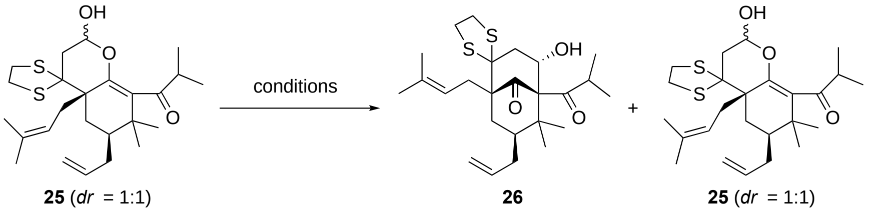

entry	conditions[a]	pK_a	results (**26**:**25**)[a]
1	cat. K_2CO_3, MeOH, 0 to 25 °C, 48 h		92% (1:1.2)
2	KHMDS, THF, –78 to 0 °C, 1 h		72% (1:1.1)
3	2.0 equiv KHMDS, THF, –78 to 0 °C, 1 h		complex mixture
4	LiHMDS, THF, –78 to 0 °C, 1 h		82% (1.1:1)
5	$AlMe_3$, toluene, 25 °C		SM recovered
6	Et_2AlOEt, DCM, 25 °C		SM recovered
7	2,6-lutidine, DCM, 40 °C, 72 h	6.8	SM recovered
8	DABCO, DCM, 40 °C, 72 h	8.8	89% (1:1.9)
9	TMEDA, DCM, 40 °C, 72 h	8.9	83% (1:1.4)
10	1-methylpiperazine, DCM, 40 °C, 72 h	9.4	45% (1:8)
11	DMAP, DCM, 40 °C, 72 h	9.7	89% (1:1.4)
12	(+)-cinchonine, DCM, 40 °C, 72 h	9.92	95% (3.2:1)
13[b]	DIPEA, DCM, 40 °C, 72 h	10.8	90% (2.8:1)
14	quinuclidine, DCM, 40 °C, 72 h	11.0	75% (1.5:1)
15	DBU, DCM, 40 °C, 72 h	12.5	50% (1:2)
16[b]	TMG, DCM, 25 °C, 1 h	13.0	93% (2.1:1)
17	Barton's base, DCM, 0 °C, 1 h	13.8	71% (2.8:1)

[a] Isolated yield. [b] In situ oxidation was possible with 2.0 equiv of DMP upon completion of equilibration. The 2.8:1 isomeric ratio was maintained even with the use of 5 equivalents of DIPEA. TMG = 1,1,3,3-tetramethylguanidine. Barton's base = 2-*tert*-butyl-1,1,3,3-tetramethyguanidine.

Dithiolane alcohol 26 and lactol 25 (Table S7, entry 13)

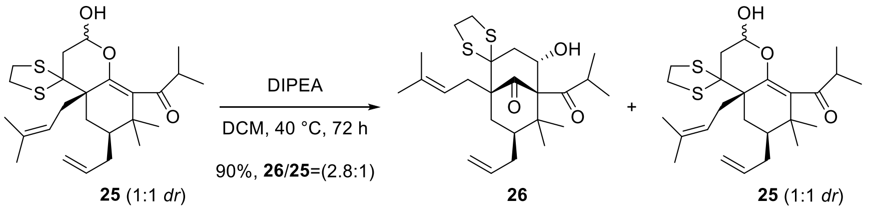

To a solution of lactol **25** (32 mg, 0.071 mmol) in DCM (2.4 mL) was added a solution of *N,N*-diisopropylethylamine (DIPEA, 0.071 mL, 1.0 M in DCM, 0.071 mmol, 1.0 equiv) at 25 ˚C. The reaction mixture was heated at 40 ˚C for 72

h. The reaction was cooled to 25 °C, diluted with a saturated aq. NH_4Cl (10 mL) and extracted twice with EtOAc (10 mL). The combined organic phases were dried over Na_2SO_4 and concentrated in vacuo. Purification of the residue by flash column chromatography (silica gel, hexanes/EtOAc = 20/1 → 4/1) afforded alcohol **26** (21 mg, 0.046 mmol, 74% yield) and lactol **25** (8 mg, 0.018 mmol, 16% yield).

Table S8. Oxidation of dithiolane triketone **27**

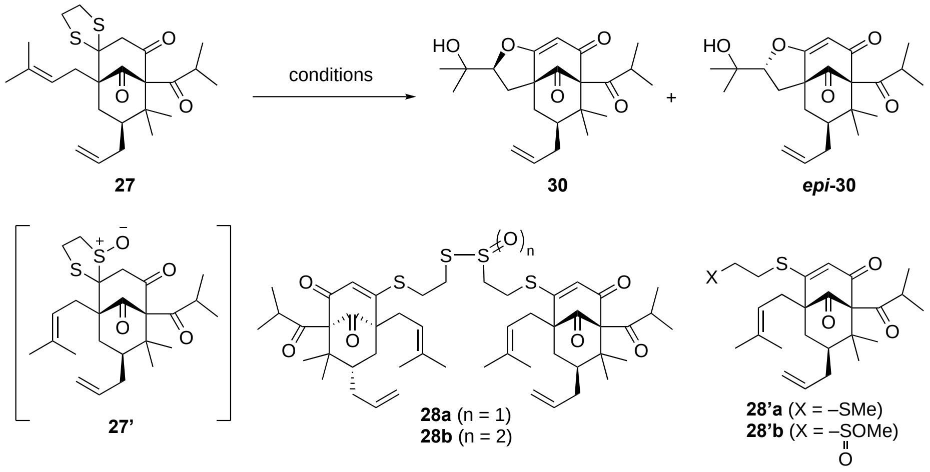

entry	conditions	results
1	4.0 equiv PIFA, MeOH, 25 °C, 1 h	**28'b** (13%) + **27** recovered (65%)
2	1.1 equiv Me_3OBF_4, DCM, 25 °C, 12 h	**27** recovered
3	excess MeI, 5 equiv $CaCO_3$, CH_3CN/H_2O, 25 → 80 °C, 12 h	**28'a** (51%)
4[a]	1.0 equiv *m*CPBA, $CDCl_3$, 0 → 25 °C, 5 min	**27'**
5	1.0 equiv *m*CPBA, DCM, 0 → 25 °C, 6 h	**28a** (50%) + **28b** (15%)
6	2.0 equiv *m*CPBA, DCM, 0 → 25 °C, 6 h	**28a** (21%) + **28b** (11%)
7[b]	4.5 equiv *m*CPBA, DCM, −10 → 0 °C, 48 h	**30** (62%) + ***epi*-30** (15%)
8	4.5 equiv *m*CPBA, Hexane, −10 → 0 °C, 36 h	**30** (36%) + ***epi*-30** (9%)
9	4.5 equiv *m*CPBA, trifluorotoluene, −10 → 0 °C, 36 h	**30** (69%) + ***epi*-30** (20%)
10	5.0 equiv DMDO, acetone/DCM, −40 → 0 °C, 36 h	**30** (22%) + ***epi*-30** (16%)
11[c]	4.5 equiv Triazox, DCM, −20 → 25 °C, 36 h	**30** (47%) + ***epi*-30** (39%)

[a] The structure of sulfoxide **27'** was inferred from the ^{1}H NMR spectrum. Upon standing at 25 °C, **27'** was converted to **28a** in two hours. [b] When unpurified commercial *m*CPBA (ca. 70–75%) was used, the diastereomeric ratio was reduced to 2:1. [c] Triazox (2-hydroperoxy-4,6-diphenyl-1,3,5-triazine) was synthesized according to the known method (ref 13).

Purification of *m*CPBA[14,15]

Commercial *m*CPBA (5.0 g, ca. 70-75%) was dissolved in 50 mL of diethyl ether and washed thrice with 20 ml buffer solution (410 ml 0.1 M NaOH, 250 ml 0.2 M KH_2PO_4 made up to 1 L, pH 7.5). The organic layer was dried over $MgSO_4$ and carefully evaporated under reduced pressure to give pure *m*CPBA (2.6 g) as dry white solid (caution: potential explosive).

Tricyclic ketone 30

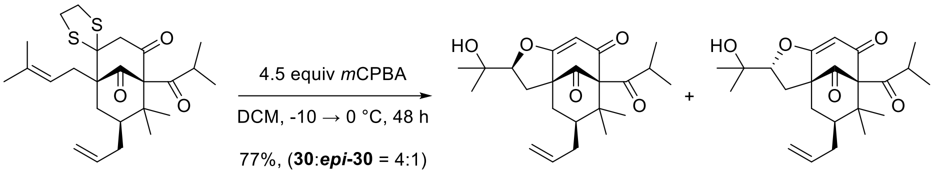

27 **30** ***epi*-30**

To a solution of dithiolane **27** (14 mg, 0.031 mmol) in DCM (3.1 mL) cooled to −10 ˚C was added purified *m*CPBA (24 mg, 0.14 mmol, 4.5 equiv). The resulting suspension was stirred for 30 minutes before it was allowed to warm to 0 ˚C and stirred at that temperature for 48 h. After addition of a saturated aqueous solution of $NaHCO_3$, the reaction mixture was extracted twice with EtOAc. The combined organic phases were dried over Na_2SO_4 and concentrated in vacuo. Purification of the residue by flash column chromatography (silica gel, hexanes/EtOAc = 10/1 → 2/1) afforded tricyclic ketone **30** (7.5 mg, 0.019 mmol, 62% yield) and ***epi*-30** (1.8 mg, 0.005 mmol, 15% yield) as both colorless liquids.

Tricyclic ketone 30 (major isomer)

$\mathbf{R}_f$ 0.22 (Hexanes/EtOAc = 2:1, vanillin)

^{1}H NMR (400 MHz, $CDCl_3$) δ 5.95 (s, 1H), 5.64 (dddd, *J* = 15.6, 10.5, 8.5, 5.3 Hz, 1H), 5.07 – 4.97 (m, 2H), 4.57 (dd, *J* = 11.0, 5.5 Hz, 1H), 2.68 (dd, *J* = 13.0, 11.1 Hz, 1H), 2.38 (dd, *J* = 12.1, 4.3 Hz, 1H), 2.15 – 2.04 (m, 2H), 1.80 (dd, *J* = 13.2, 5.5 Hz, 1H), 1.72 (dd, *J* = 10.6, 4.4 Hz, 1H), 1.65 (d, *J* = 15.0 Hz, 1H), 1.55 (dd, *J* = 15.6, 9.7 Hz, 2H), 1.39 (s, 3H), 1.28 (s, 3H), 1.23 (s, 3H), 1.11 (d, *J* = 6.5 Hz, 3H), 1.06 (s, 3H), 1.01 (d, *J* = 6.5 Hz, 3H).

^{13}C NMR (101 MHz, $CDCl_3$) δ 208.6, 204.3, 193.6, 178.2, 136.6, 117.1, 104.5, 90.9, 82.3, 70.7, 59.7, 46.4, 42.0, 41.0, 38.1, 32.7, 29.7, 26.9, 24.3, 22.7, 21.5, 20.4, 16.0.

FTIR (neat, cm^{-1}); 3458, 2975, 1728, 1624, 1468, 1373, 1178.

HRMS (ESI) *m/z* calcd. for $C_{23}H_{32}O_5Na^+$ $[M+Na]^+$: 411.2145, found: 411.2142.

$[\alpha]_D^{22}$ +38.3 (*c* 0.5, EtOH).

Tricyclic ketone *epi*-30 (minor isomer)

$\mathbf{R}_f$ 0.31 (Hexanes/EtOAc = 2:1, vanillin)

^{1}H NMR (400 MHz, $CDCl_3$) δ 5.96 (s, 1H), 5.63 (td, *J* = 17.5, 6.7 Hz, 1H), 5.03 – 4.95 (m, 2H), 4.43 (dd, *J* = 10.3, 5.0 Hz, 1H), 3.01 (dd, *J* = 13.6, 10.3 Hz, 1H), 2.48 (dd, *J* = 14.0, 3.7 Hz, 1H), 2.39 – 2.29 (m, 1H), 2.11 (dt, *J* = 13.0, 6.5 Hz, 1H), 1.85 (dd, *J* = 13.6, 4.9 Hz, 1H), 1.67 (dd, *J* = 17.7, 9.5 Hz, 2H), 1.60 – 1.49 (m, 2H), 1.43 (s, 3H), 1.27 (s, 3H), 1.24 (s, 3H), 1.10 (d, *J* = 6.5 Hz, 3H), 1.04 (s, 3H), 1.02 (d, *J* = 6.5 Hz, 3H).

^{13}C NMR (101 MHz, $CDCl_3$) δ 208.6, 204.1, 193.7, 178.7, 136.6, 116.7, 105.3, 92.2, 82.0, 70.9, 59.7, 45.8, 42.0, 41.7, 40.7, 32.6, 27.9, 27.2, 25.6, 22.5, 21.5, 20.5, 16.1.

FTIR (neat, cm^{-1}) 3438, 2974, 1731, 1623, 1376, 1199, 1179, 948.

HRMS (ESI) *m/z* calcd. for $C_{23}H_{32}O_5Na^+$ $[M+Na]^+$: 411.2143, found: 411.2142.

$[\alpha]_D^{26}$ −17.9 (*c* 0.2, EtOH).

***S*-Methylation of dithioketal 27 to 28'a**

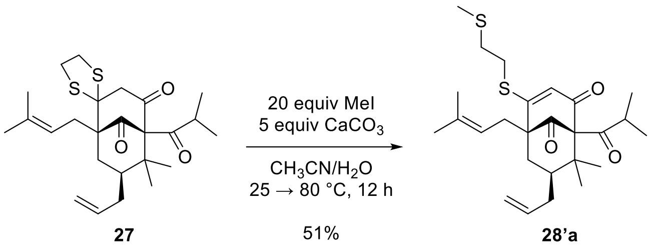

To a solution of dithiolane **27** (12 mg, 0.027 mmol) in CH_3CN/H_2O (10:1, 1 mL) were added calcium carbonate (14 mg, 0.14 mmol, 5 equiv) and methyl iodide (0.033 mL, 0.54 mmol, 20 equiv) at 25 °C. The resulting suspension was heated up to 80 ˚C. After 12 h, excess reagents were quenched with a saturated aq. NH_4Cl (5 mL). The resulting biphasic mixture was extracted several times with EtOAc (10 mL). The organic layers were dried over anhydrous Na_2SO_4 and filtered. The filtrate was concentrated under reduced pressure, and the residue was purified by flash column chromatography (silica gel, hexanes/EtOAc = 20/1 → 4/1) to afford methyl sulfide **29'a** (6.4 mg, 0.014 mmol, 51% yield) as a colorless liquid.

R*f* 0.49 (hexanes/EtOAc = 4:1, vanillin)

^{1}H NMR (400 MHz, $CDCl_3$) δ 6.37 (s, 1H), 5.68 – 5.54 (m, 1H), 5.07 – 4.92 (m, 3H), 3.14 – 3.06 (m, 2H), 2.79 (t, *J* = 7.6 Hz, 2H), 2.63 (dd, *J* = 15.6, 5.6 Hz, 1H), 2.44 (dd, *J* = 15.6, 6.7 Hz, 1H), 2.31 (dd, *J* = 13.5, 3.7 Hz, 1H), 2.20 (s, 3H), 2.15 – 2.03 (m, 1H), 1.94 (d, *J* = 10.0 Hz, 1H), 1.69 (s, 3H), 1.66 (s, 3H), 1.61 – 1.47 (m, 3H), 1.22 (s, 3H), 1.13 (d, *J* = 6.5 Hz, 3H), 1.03 (d, *J* = 6.5 Hz, 3H), 1.01 (s, 3H).

^{13}C NMR (101 MHz, $CDCl_3$) δ 208.5, 206.0, 190.6, 168.3, 136.7, 134.7, 123.7, 118.9, 116.8, 83.7, 57.2, 46.6, 42.7, 41.4, 40.2, 32.5, 31.7, 31.5, 31.0, 25.9, 22.6, 21.4, 20.4, 18.2, 15.8, 15.6.

FTIR (neat, cm^{-1}) 2973, 2923, 1730, 1641, 1556, 1440, 1375, 1266, 1067, 917.

HRMS (ESI) *m/z* calcd. for $C_{26}H_{38}O_3S_2Na^+$ $[M+Na]^+$: 485.2156, found: 485.2155.

PIFA oxidation of 27 to sulfinate ester 28'b

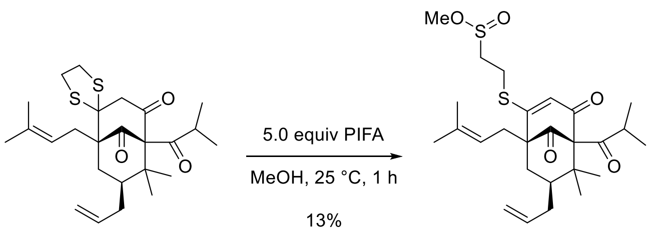

To a solution of dithiolane **27** (17 mg, 0.038 mmol) in MeOH (1.5 mL) was added [bis(trifluoroacetoxy)iodo]benzene (PIFA, 82 mg, 0.19 mmol, 5 equiv) at 25 °C. After being stirred for 1 h, excess reagents were quenched with a saturated aq. $NaHCO_3$ (5 mL) and $Na_2S_2O_3$ (5 mL). The resulting biphasic mixture was extracted several times with EtOAc (10 mL). The organic layers were dried over anhydrous Na_2SO_4, filtered and concentrated under reduced pressure. Purification of the residue by flash column chromatography (silica gel, hexanes/EtOAc = 5/1 → 1/1) afforded sulfinate ester **28'b** (2.4 mg, 0.005 mmol, 13% yield) as a colorless liquid.

Characterization data for the 1:1 diastereomeric (*S*-stereocenter) mixture of **28'b**

R*f* 0.13 (hexanes/EtOAc = 2:1, vanillin)

^{1}H NMR (400 MHz, $CDCl_3$) δ 6.40 (s, 1H), 5.68 – 5.52 (m, 1H), 5.06 – 4.93 (m, 2H), 3.84 (s, 3H), 3.32 – 3.16 (m, 2H), 3.09 – 2.99 (m, 2H), 2.63 (dd, *J* = 15.6, 5.3 Hz, 1H), 2.41 (dd, *J* = 15.5, 6.9 Hz, 1H), 2.31 (dd, *J* = 13.4, 4.0 Hz, 1H), 2.09 (dt, *J* = 12.8, 6.2 Hz, 1H), 1.92 (dd, *J* = 9.5, 1.7 Hz, 1H), 1.69 (s, 3H), 1.65 (s, 3H), 1.56 (s, 3H), 1.55 – 1.47 (m, 1H), 1.23 (s, 3H), 1.14 (d, *J* = 6.5 Hz, 3H), 1.03 (d, *J* = 6.6 Hz, 3H), 1.01 (s, 3H).

^{13}C NMR (126 MHz, $CDCl_3$) δ 208.4, 205.8, 190.6, 167.2, 136.6, 134.9, 124.0, 118.7, 116.9, 83.8, 57.1, 55.4, 53.6, 46.8, 42.8, 41.5, 40.1, 32.5, 31.1, 25.9, 22.7, 22.6, 21.4, 20.4, 18.2, 15.8.

FTIR (neat, cm^{-1}) 2927, 1731, 1645, 1600, 1449, 1371, 1235, 1064, 915, 830.

HRMS (ESI) *m/z* calcd. for $C_{26}H_{38}O_5S_2Na^+$ $[M+Na]^+$: 517.2054, found: 517.2053.

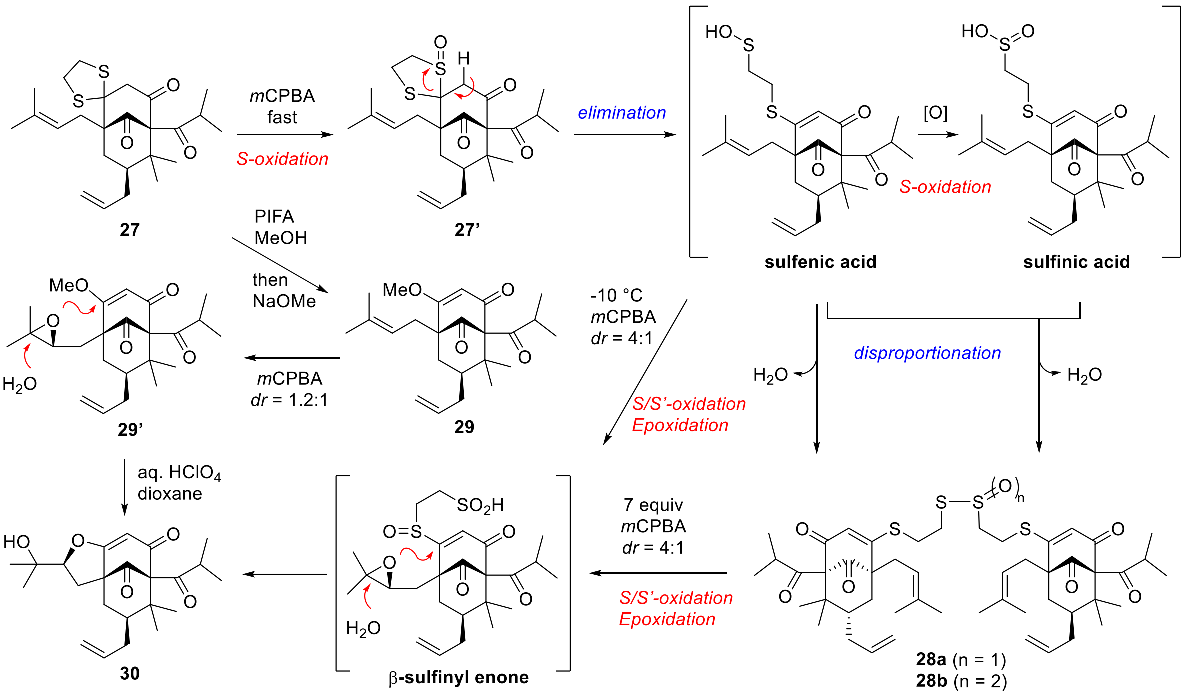

Scheme S5. Possible reaction pathways in the dithiolane oxidative removal step

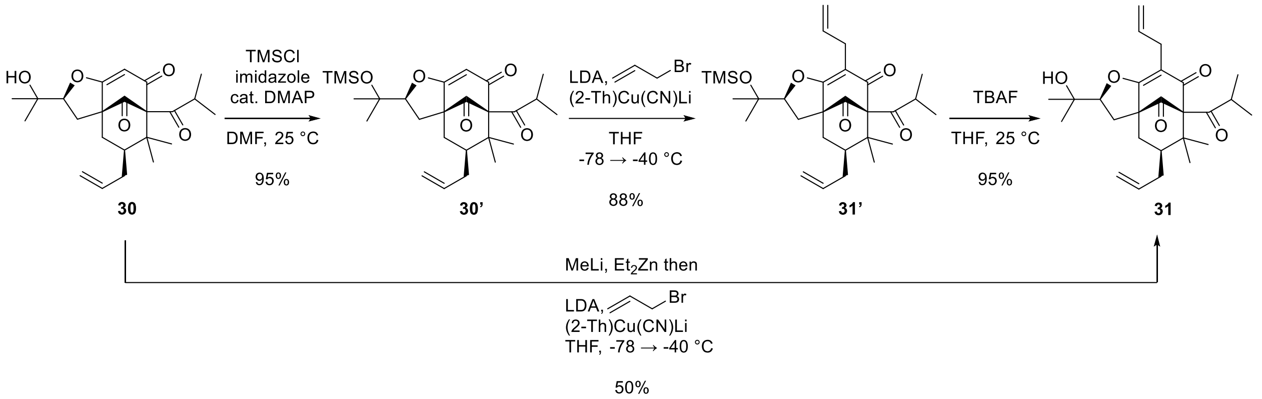

Scheme S6. Alkenyl C-H allylation

TMS protection of 30 to 30'

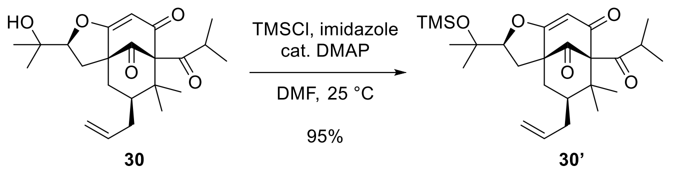

To a mixture of alcohol **30** (8 mg, 0.021 mmol), imidazole (14 mg, 0.21 mmol, 10 equiv) and 4-(dimethylamino)-pyridine (DMAP, 0.3 mg, 0.0021 mmol, 0.1 equiv) in DMF (1 mL) was added trimethylsilyl chloride (TMSCl, 0.013 mL, 0.11 mmol, 5.0 equiv) at 25 °C. After 1 h, the reaction mixture was poured into a saturated aqueous solution of NH_4Cl and extracted with diethyl ether several times. The combined organic layers were dried over anhydrous $MgSO_4$, filtered and concentrated under reduced pressure. Purification of the residue by flash column chromatography (silica gel, hexanes/EtOAc = 20/1 → 4/1) afforded TMS ether **30'** (9 mg, 0.02 mmol, 95% yield) as a colorless liquid.

$\mathbf{R}_f$ 0.47 (hexanes/EtOAc = 4:1, vanillin)

^{1}H NMR (400 MHz, $CDCl_3$) δ 5.92 (s, 1H), 5.63 (dddd, *J* = 15.9, 10.7, 8.5, 5.2 Hz, 1H), 5.06 – 4.96 (m, 2H), 4.50 (dd, *J* = 10.1, 6.1 Hz, 1H), 2.77 (dd, *J* = 13.1, 10.1 Hz, 1H), 2.37 (ddd, *J* = 13.0, 5.0, 1.7 Hz, 1H), 2.10 (dt, *J* = 13.0, 6.5 Hz, 1H), 2.07 (dd, *J* = 17.2, 5.4 Hz, 1H), 1.79 – 1.63 (m, 3H), 1.53 (dd, *J* = 13.5, 12.0 Hz, 1H), 1.36 (s, 3H), 1.28 (s, 3H), 1.22 (s, 3H), 1.10 (d, *J* = 6.5 Hz, 3H), 1.05 (s, 3H), 1.02 (d, *J* = 6.5 Hz, 3H), 0.08 (s, 9H).

^{13}C NMR (101 MHz, $CDCl_3$) δ 208.8, 204.5, 193.8, 179.0, 136.7, 117.0, 104.0, 91.6, 82.2, 73.6, 59.8, 46.2, 41.9, 41.2, 38.9, 32.7, 29.2, 26.7, 26.1, 22.7, 21.5, 20.3, 16.1, 2.2.

FTIR (neat, cm^{-1}) 2974, 1734, 1629, 1373, 1252, 1177, 1055, 899, 841.

HRMS (ESI) *m/z* calcd. for $C_{26}H_{40}O_5SiNa^+$ $[M+Na]^+$: 483.2538, found: 483.2537.

$[\alpha]_D^{26}$ +49.6 (*c* 0.45, EtOH).

Allylation of TMS ether 30' to prepare bis-allyl tricycle 31'

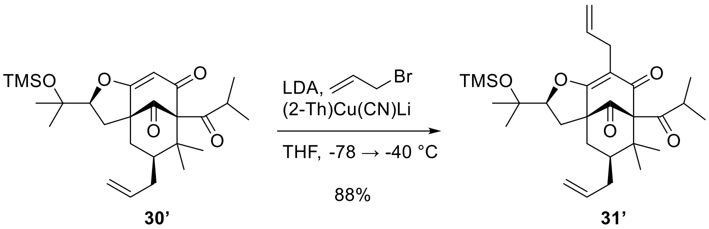

30' **31'**

To a solution of TMS ether **30'** (8 mg, 0.017 mmol) in THF (0.85 mL) was added dropwise a freshly prepared THF solution of lithium diisopropylamide (LDA, 0.05 mL, 1.0 M in THF, 0.051 mmol, 3.0 equiv) at −78 ˚C. After stirring the resulting light-yellow solution at −78 ˚C for 30 min, a freshly prepared THF solution of 2-thienyl(cyano)copper lithium (0.51 mL, 0.1 M in THF, 0.051 mmol, 3.0 equiv) was added dropwise. The resulting dark yellow solution was warmed to –40 ˚C and stirred at for additional 30 min, after which allyl bromide (0.022 mL, 0.26 mmol, 15 equiv) was added. After being stirred at –40 °C for 1 h, the reaction mixture was diluted with EtOAc (10 mL) and saturated aq. NH_4Cl (10 mL). After allowed to warm to 25 °C, the layers were separated and extracted twice with EtOAc (10 mL). The combined organic extracts were dried over Na_2SO_4 and concentrated in vacuo. Purification of the residue by flash column chromatography (silica gel, hexanes/EtOAc = 40/1 → 8/1) afforded the bis-allyl tricycle **31'** (7.7 mg, 0.015 mmol, 88% yield) as a colorless liquid.

$\mathbf{R}_f$ 0.59 (hexanes/EtOAc = 8:1, vanillin)

^{1}H NMR (400 MHz, $CDCl_3$) δ 5.80 (ddt, *J* = 16.6, 10.0, 6.5 Hz, 1H), 5.69 – 5.55 (m, 1H), 5.00 (dt, *J* = 16.2, 15.4 Hz, 4H), 4.48 (dd, *J* = 10.0, 6.2 Hz, 1H), 3.13 (ddd, *J* = 30.3, 14.5, 6.5 Hz, 2H), 2.78 (dd, *J* = 13.0, 10.1 Hz, 1H), 2.41 – 2.30 (m, 1H), 2.07 (dd, *J* = 13.3, 3.6 Hz, 1H), 1.98 (dt, *J* = 13.0, 6.5 Hz, 1H), 1.74 (dd, *J* = 13.1, 6.1 Hz, 1H), 1.64 (ddd, *J* = 14.8, 12.6, 6.1 Hz, 2H), 1.51 (dd, *J* = 21.2, 7.9 Hz, 1H), 1.34 (s, 3H), 1.27 (s, 3H), 1.24 (s, 3H), 1.08 (d, *J* = 6.5 Hz, 3H), 1.04 (s, 3H), 0.98 (d, *J* = 6.5 Hz, 3H), 0.09 (s, 9H).

^{13}C NMR (101 MHz, $CDCl_3$) δ 208.9, 204.5, 192.5, 174.4, 136.8, 134.8, 116.8, 115.2, 114.3, 91.1, 82.0, 73.8, 59.7, 46.1, 42.0, 41.3, 39.1, 32.7, 29.8, 27.3, 26.4, 26.1, 22.7, 21.3, 20.3, 16.1, 2.2.

FTIR (neat, cm^{-1}) 2974, 1733, 1625, 1372, 1251, 1229, 1051, 915, 842.

HRMS (ESI) *m/z* calcd. for $C_{29}H_{44}O_5SiNa^+$ $[M+Na]^+$: 523.2852, found: 523.2850.

$[\alpha]_D^{24}$ +37.1 (*c* 0.3, EtOH).

Desilylation of TMS ether 31' to alcohol 31

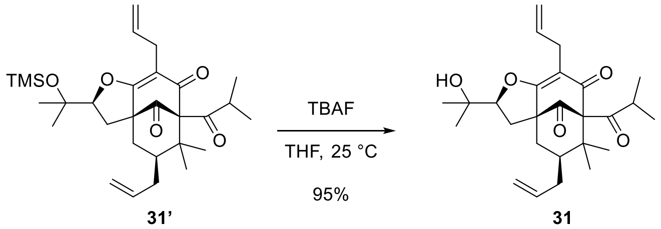

31' **31**

To a solution of TMS ether **31'** (8 mg, 0.016 mmol) in 0.8 mL of THF was added tetrabutylammonium fluoride (TBAF, 0.08 mL, 1.0 M in THF, 0.08 mmol, 5.0 equiv). The reaction mixture was stirred at 25 °C for 30 min and poured into

a saturated aqueous solution of $NaHCO_3$. After extraction twice with EtOAc, the combined organic extracts were dried over Na_2SO_4 and concentrated in vacuo. Purification of the residue by flash column chromatography (silica gel, hexanes/EtOAc = 10/1 → 3/1) afforded alcohol **31** (6.5 mg, 0.015 mmol, 95% yield) as a colorless liquid.

R*f* 0.35 (hexanes/EtOAc = 2:1, vanillin)

^{1}H NMR (400 MHz, $CDCl_3$) δ 5.85 – 5.71 (m, 1H), 5.60 (dd, *J* = 15.4, 8.9 Hz, 1H), 5.10 – 4.92 (m, 4H), 4.57 (dd, *J* = 10.7, 5.8 Hz, 1H), 3.25 (dd, *J* = 14.6, 4.7 Hz, 1H), 3.07 (dd, *J* = 14.5, 6.9 Hz, 1H), 2.75 – 2.65 (m, 1H), 2.37 (dd, *J* = 12.0, 4.4 Hz, 1H), 2.18 (s, 1H), 2.09 (dd, *J* = 13.3, 3.2 Hz, 1H), 2.04 – 1.94 (m, 1H), 1.81 (dd, *J* = 13.2, 5.5 Hz, 1H), 1.74 – 1.60 (m, 2H), 1.55 – 1.48 (m, 1H), 1.38 (s, 3H), 1.27 (s, 3H), 1.21 (s, 3H), 1.10 (d, *J* = 6.4 Hz, 3H), 1.05 (s, 3H), 0.99 (d, *J* = 6.5 Hz, 3H).

^{13}C NMR (101 MHz, $CDCl_3$) δ 208.7, 204.3, 192.3, 173.8, 136.7, 135.1, 117.0, 115.0, 114.6, 90.3, 82.2, 70.8, 59.7, 46.3, 42.1, 41.3, 38.2, 32.7, 30.1, 27.1, 27.0, 24.0, 22.7, 21.4, 20.5, 16.0.

FTIR (neat, cm^{-1}) 3499, 2975, 2928, 1732, 1625, 1472, 1368, 1231, 993, 918.

HRMS (ESI)*m/z* calcd. for $C_{26}H_{36}O_5Na^+$ $[M+Na]^+$: 451.2458, found: 451.2455.

$[\alpha]_D^{25}$ +24.9 (*c* 0.07, EtOH)

Direct alkenyl C-H allylation of alcohol 30 to form bis-allyl tricycle 31

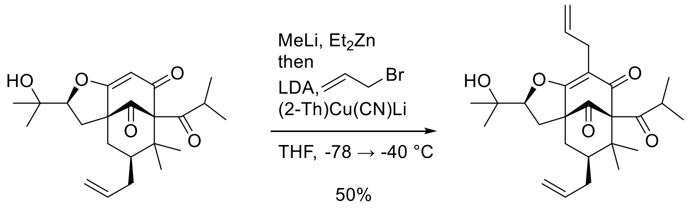

30 **31**

To a solution of alcohol **30** (9 mg, 0.023 mmol) in THF (1 mL) was added dropwise MeLi (0.022 mL, 1.6 M in Et_2O, 0.035 mmol, 1.5 equiv) at 0 °C. After stirring 1 h, diethyl zinc (0.035 mL, 1.0 M in hexane, 0.035 mmol, 1.5 equiv) was slowly added and stirring was continued for 30 min. After cooling to −78 °C, a freshly prepared THF solution of lithium diisopropylamide (LDA, 0.14 mL, 0.5 M in THF, 0.069 mmol, 3.0 equiv) was added dropwise. After stirring the resulting yellow solution at −40 °C for 1 h, 2-thienyl(cyano)copper lithium (0.28 mL, 0.25 M in THF, 0.069 mmol, 3.0 equiv) was added dropwise. The resulting dark yellow solution was stirred for additional 30 min, after which allyl bromide (0.01 mL, 0.12 mmol, 5.0 equiv) was added. After being stirred at −40 °C for 1 h, the reaction mixture was diluted with EtOAc (10 mL) and saturated aq. NH_4Cl (10 mL). After allowed to warm to 25 °C, the biphasic solution was separated and extracted twice with EtOAc (10 mL). The combined organic extracts were dried over Na_2SO_4 and concentrated in vacuo. Purification of the residue by flash column chromatography (silica gel, hexanes/EtOAc = 10/1 → 2/1) afforded bis-allyl tricycle **31** (4.9 mg, 0.012 mmol, 50% yield) as a colorless liquid and unreacted starting material **30** (1.3 mg, 0.0035 mmol, 15% yield).

Ruthenium-catalyzed cross-metathesis of 31 to form garsubellin A (1)

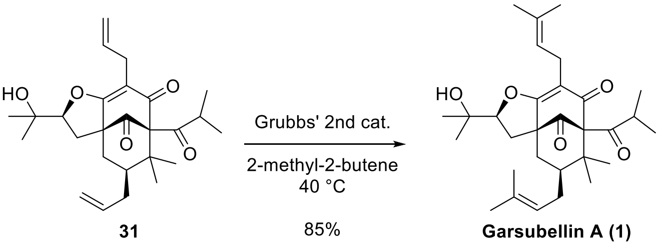

To a solution of bis-allyl tricycle **31** (5 mg, 0.012 mmol) in 2-methyl-but-2-ene (2 mL) was added the 2nd generation Grubbs catalyst® (1 mg, 0.0012 mmol, 0.1 equiv) at 25 °C. The resulting suspension was heated at 40 ˚C for 12 h, cooled to 25 °C and concentrated. Purification of the residue by flash column chromatography (silica gel, hexanes/EtOAc = 50/1 → 10/1) afforded garsubellin A (**1**, 5 mg, 0.01 mmol, 85% yield) as a colorless liquid.

$\mathbf{R}_f$ 0.48 (hexanes/EtOAc = 2:1, vanillin)

^{1}H NMR (400 MHz, C_6D_6) δ 5.39 (t, J = 7.1 Hz, 1H), 5.00 – 4.92 (m, 1H), 3.91 (dd, J = 10.6, 5.8 Hz, 1H), 3.38 (dd, J = 14.0, 7.0 Hz, 1H), 3.20 (dd, J = 14.0, 7.5 Hz, 1H), 2.72 (dd, J = 13.0, 10.8 Hz, 1H), 2.25 (dt, J = 12.9, 6.5 Hz, 1H), 2.13 – 2.04 (m, 1H), 1.92 (dd, J = 13.5, 4.4 Hz, 1H), 1.80 – 1.72 (m, 1H), 1.69 (s, 3H), 1.60 (s, 6H), 1.57 (s, 3H), 1.56 – 1.50 (m, 1H), 1.44 (s, 3H), 1.36 (d, J = 6.5 Hz, 3H), 1.31 – 1.29 (m, 1H), 1.29 (d, J = 6.5 Hz, 3H), 1.28 – 1.26 (m, 1H), 1.24 (s, 3H), 0.93 (s, 3H), 0.76 (s, 3H).

^{13}C NMR (101 MHz, C_6D_6) δ 208.5, 204.7, 192.9, 173.2, 133.2, 132.5, 123.3, 122.1, 116.8, 90.2, 82.7, 70.3, 59.9, 46.7, 43.1, 42.8, 39.1, 30.3, 27.1, 26.4, 26.0, 25.8, 24.5, 23.2, 22.7, 22.0, 20.9, 17.93, 17.91, 16.6.

FTIR (neat, cm^{-1}) 3474, 2973, 2932, 1731, 1625, 1447, 1366, 1212.

HRMS (ESI) *m/z* calcd. for $C_{30}H_{44}O_5Na^+$ [M+Na]$^+$:507.3085, found: 507.3081.

$[\boldsymbol{\alpha}]_{\boldsymbol{D}}^{\mathbf{25}}$ +23.9 (*c* 0.4, EtOH).

3.4. Alternative approach toward tricyclic ketone **30**

Oxidation of 27 to dimeric thiosulfinate 28a and thiosulfonate 28b

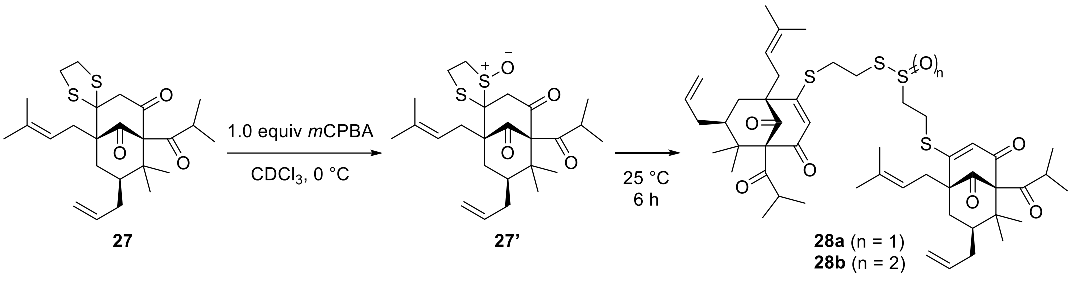

To a solution of dithiolane **27** (14 mg, 0.031 mmol) in $CDCl_3$ (3.1 mL) was added purified *m*CPBA (24 mg, 0.14 mmol, 1.0 equiv) at 0 ˚C. After being stirred for 30 min, the reaction mixture was directly transferred into an NMR tube. After obtaining the ^{1}H NMR spectrum of the crude reaction mixture which indicated complete consumption of **27**, the NMR tube was left at 25 °C for 6 h. The reaction mixture was then poured into a saturated aqueous solution of $NaHCO_3$ and extracted twice with EtOAc. The combined organic phases were dried over Na_2SO_4 and concentrated in vacuo. Purification of the residue by flash column chromatography (silica gel, hexanes/EtOAc = 8/1 → 2/1) afforded dimeric thiosulfinate **28a** (7 mg, 0.0077 mmol, 50% yield) and dimeric thiosulfonate **28b** (2.2 mg, 0.0024 mmol, 15% yield) both as yellow liquids.

Thiosulfinate 28a

R*f* 0.39 (hexanes/EtOAc = 2:1, vanillin)

Partial characterization of major peak

^{1}H NMR (400 MHz, $CDCl_3$) δ 6.42 (s, 1H), 6.41 (s, 1H), 5.69 – 5.54 (m, 2H), 5.07 – 4.94 (m, 6H), 3.43 (dt, *J* = 13.4, 8.5 Hz, 6H), 3.37 – 3.21 (m, 4H), 2.65 (d, *J* = 15.7 Hz, 2H), 2.43 (dd, *J* = 15.7, 6.8 Hz, 2H), 2.36 – 2.26 (m, 2H), 2.15 – 2.03 (m, 2H), 1.99 – 1.88 (m, 2H), 1.70 (s, 6H), 1.66 (s, 6H), 1.53 (d, *J* = 7.6 Hz, 4H), 1.23 (s, 6H), 1.14 (d, *J* = 6.5 Hz, 6H), 1.03 (d, *J* = 6.6 Hz, 6H), 1.01 (s, 6H).

^{13}C NMR (101 MHz, $CDCl_3$) δ 208.2, 205.7, 190.6, 166.9, 136.7, 134.9, 124.0, 118.8, 116.9, 83.8, 57.2, 52.8, 46.9, 42.8, 41.5, 40.1, 32.4, 31.1, 25.9, 24.6, 22.5, 21.4, 20.4, 18.3, 15.8.

FTIR (neat, cm^{-1}) 2974, 2928, 1730, 1642, 1559, 1442, 1266, 1075, 917, 833.

HRMS (FAB) *m/z* calcd. for $C_{50}H_{71}O_7S_4^+$ $[M+H]^+$: 911.4080, found: 911.4077.

Thiosulfonate 28b

R*f* 0.48 (hexanes/EtOAc =2:1 , vanillin)

Partial characterization of major peak

^{1}H NMR (400 MHz, $CDCl_3$) δ 6.43 (s, 1H), 6.40 (d, *J* = 2.9 Hz, 1H), 5.62 (ddd, *J* = 15.9, 9.2, 5.6 Hz, 2H), 5.02 (dd, *J* = 17.1, 10.9 Hz, 6H), 3.61 (dt, *J* = 16.7, 6.3 Hz, 2H), 3.46 – 3.27 (m, 8H), 2.64 (dd, *J* = 16.1, 4.8 Hz, 2H), 2.47 – 2.37 (m, 2H), 2.36 – 2.27 (m, 2H), 2.14 – 2.00 (m, 2H), 1.92 (dd, *J* = 9.8, 6.7 Hz, 2H), 1.70 (s, 6H), 1.66 (s, 6H), 1.60 – 1.46 (m, 4H), 1.22 (s, 6H), 1.14 (d, *J* = 6.5 Hz, 6H), 1.03 (d, *J* = 6.6 Hz, 6H), 1.01 (s, 6H).

^{13}C NMR (101 MHz, $CDCl_3$) δ 208.2, 207.9, 205.6, 205.3, 190.6, 166.4, 166.0, 166.0, 136.7, 136.6, 135.1, 135.0, 124.1, 124.0, 118.7, 118.5, 117.1, 117.0, 83.8, 59.6, 57.2, 57.1, 46.9, 46.8, 42.9, 42.8, 41.8, 41.6, 40.1, 40.0, 34.1, 32.4, 32.4, 31.7, 31.0, 25.9, 24.7, 22.5, 21.4, 20.4, 18.2, 15.8.

FTIR (neat, cm^{-1}) 2974, 1730, 1643, 1560, 1442, 1330, 1267, 1134, 918., 835.

HRMS (ESI) *m/z* calcd. for $C_{50}H_{70}O_8S_4Na^+$ $[M+Na]^+$: 949.3851, found: 949.3846.

Oxidation and desulfurization of thiosulfinate 28a to form tricyclic ketone 30 and *epi*-30

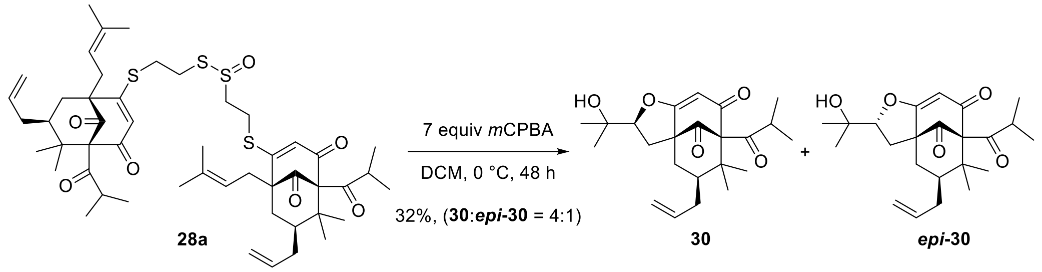

To a solution of thiosulfinate **28a** (8 mg, 0.009 mmol) in DCM (0.9 mL) of DCM cooled to −10 ˚C was added purified *m*CPBA (11 mg, 0.063 mmol, 7.0 equiv). The resulting solution was stirred at −10 ˚C for 30 minutes before it was allowed to warm to 0 ˚C. After being stirred at 0 ˚C for 48 h, the reaction mixture was poured into a saturated aqueous solution of $NaHCO_3$ and extracted twice with EtOAc. The combined organic phases were dried over Na_2SO_4 and concentrated in vacuo. Purification of the residue by flash column chromatography (silica gel, hexanes/EtOAc = 10/1 → 2/1) gave tricyclic ketone **30** (1.7 mg, 0.004 mmol, 26% yield) and ***epi*-30** (0.5 mg, 0.001 mmol, 6% yield) both as colorless liquids.

Methanolysis of 28a to vinylogous methyl ester 29

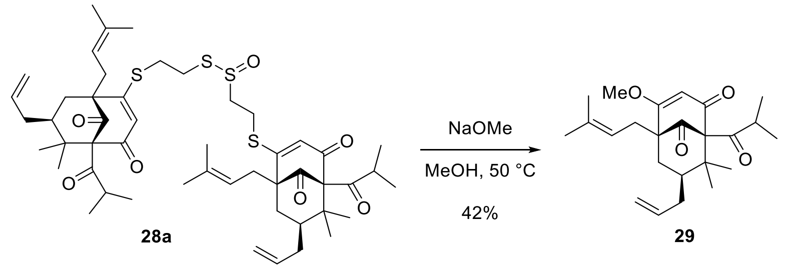

To a solution of thiosulfinate **28a** (8 mg, 0.009 mmol) in MeOH (0.9 mL) was added dropwise a solution of sodium methoxide (0.09 mL, 0.5 M in MeOH, 0.045 mmol, 5.0 equiv) at 25 °C. After heated at 50 ˚C for 6 h, the reaction mixture was poured into a saturated aqueous solution of NH_4Cl and extracted with EtOAc. The combined organic phases were dried over Na_2SO_4 and concentrated in vacuo. Purification of the residue by flash column chromatography (silica gel, hexanes/EtOAc = 20/1 → 4/1) afforded the vinylogous methyl ester **29** (2.8 mg, 0.008 mmol, 42% yield) as a colorless liquid.

$\mathbf{R}_f$ 0.43 (hexanes/EtOAc = 4:1, vanillin)

^{1}H NMR (400 MHz, $CDCl_3$) δ 5.91 (s, 1H), 5.61 (ddd, *J* = 17.2, 12.0, 7.0 Hz, 1H), 5.03 – 4.93 (m, 3H), 3.80 (s, 3H), 2.47 (ddd, *J* = 31.5, 14.8, 7.2 Hz, 2H), 2.30 (dd, *J* = 11.4, 4.9 Hz, 1H), 2.11 (dt, *J* = 13.1, 6.5 Hz, 1H), 1.90 (dd, *J* =

13.7, 3.5 Hz, 1H), 1.67 (s, 6H), 1.65 – 1.53 (m, 2H), 1.48 – 1.37 (m, 1H), 1.24 (s, 3H), 1.13 (d, *J* = 6.5 Hz, 3H), 1.03 (d, *J* = 6.5 Hz, 3H), 0.99 (s, 3H).

^{13}C NMR (101 MHz, $CDCl_3$) δ 208.6, 206.7, 192.5, 177.2, 136.8, 134.3, 118.9, 116.7, 106.7, 83.2, 57.1, 56.9, 46.6, 42.5, 41.7, 39.3, 32.8, 29.4, 25.9, 22.9, 21.5, 20.5, 18.0, 15.6.

FTIR (neat, cm^{-1}) 2973, 2925, 1729, 1642, 1559, 1443, 1381, 1322, 1267, 1136, 992.

HRMS (ESI) *m/z* calcd. for $C_{24}H_{34}O_4Na^+$ $[M+Na]^+$: 409.2354, found: 409.2349.

Methanolysis of 28'b to vinylogous methyl ester 29

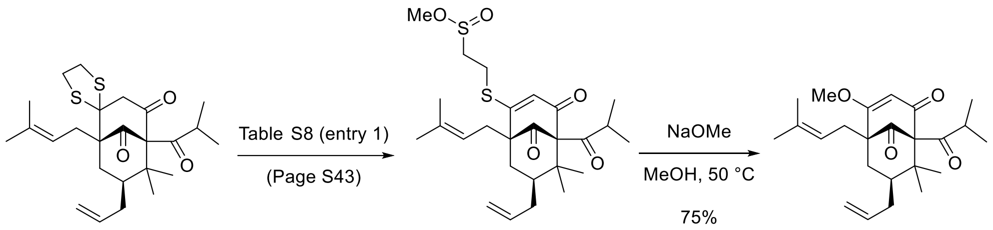

27 28'b 29

Following the procedure for vinylogous methyl ester **29**, sulfinate ester **28'b** (10 mg, 0.020 mmol) was reacted with a solution of sodium methoxide (0.20 mL, 0.5 M in MeOH, 0.10 mmol, 5.0 equiv) in MeOH (2 mL) to obtain vinylogous methyl ester **29** (5.8 mg, 0.015 mmol, 75% yield) as a colorless liquid.

Epoxidation of methyl ether 29 to epoxide 29'

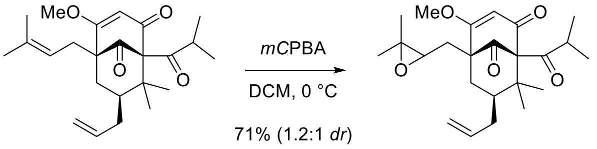

29 29'

To a solution of vinylogous methyl ester **29** (20 mg, 0.054 mmol) in DCM (1.8 mL) was added purified *m*CPBA (10.3 mg, 0.059 mmol, 1.1 equiv) at 0 ˚C. The reaction mixture was left to stir at that temperature for 1 h, at which time the starting material was consumed as judged by TLC and a saturated aqueous solution of $NaHCO_3$ was added. After extraction with DCM, the combined organic extracts were dried over Na_2SO_4 and concentrated in vacuo. Purification of the residue by flash column chromatography (silica gel, hexanes/EtOAc = 20:1 → 3:1) afforded epoxide **29'** (14.9 mg, 0.038 mmol, 71% yield, inseparable mixture, 1.2:1 *dr*) as a colorless liquid.

Characterization data for the signals from the major isomer of **29'**

R*f* 0.10 (hexanes/EtOAc = 4:1, vanillin)

^{1}H (400 MHz, $CDCl_3$) δ 5.98 (s, 1H), 5.67 – 5.55 (m, 1H), 5.05 – 4.94 (m, 2H), 3.87 (s, 3H), 2.60 (t, *J* = 5.0 Hz, 1H), 2.37 – 2.27 (m, 1H), 2.15 – 2.09 (m, 1H), 2.09 – 2.00 (m, 1H), 2.00 – 1.92 (m, 1H), 1.67 (t, *J* = 8.4 Hz, 1H), 1.48 – 1.39 (m, 1H), 1.35 (s, 3H), 1.28 (s, 3H), 1.26 (s, 3H), 1.12 (d, *J* = 6.9 Hz, 3H), 1.04 (d, *J* = 6.4 Hz, 3H), 1.01 (s, 3H).

^{13}C NMR (101 MHz, $CDCl_3$) δ 208.3, 207.0, 192.2, 176.1, 136.6, 116.9, 106.8, 83.2, 61.0, 57.2, 56.2, 46.8, 42.6, 41.5, 39.7, 32.7, 30.2, 24.5, 22.8, 21.5, 20.4, 19.0, 15.6.

FTIR (neat, cm^{-1}) 2933, 1731, 1647, 1601, 1457, 1372, 1234, 1107, 916, 831.

HRMS (ESI) *m/z* calcd. for $C_{24}H_{34}O_5Na^+$ $[M+Na]^+$: 425.2300, found: 425.2299.

Hydrolysis of epoxide 29' to 30 and *epi*-30

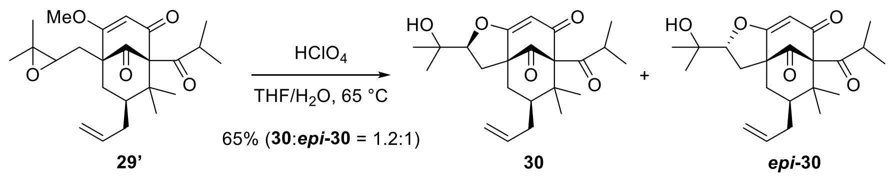

To a solution of epoxide **29'** (14 mg, 0.035 mmol, *dr* = 1.2:1) in THF/H_2O (4:1, 1.8 mL) was added perchloric acid (0.18 mL, 2 *N* in H_2O). After heated at 65 ˚C for 12 h, the reaction mixture was poured into a saturated aqueous solution of $NaHCO_3$ and extracted with EtOAc. The combined organic extracts were washed with brine, dried over Na_2SO_4, filtered, and concentrated. The crude oil was purified by flash column chromatography (silica gel, hexanes/EtOAc = 10/1 → 2/1) to give tricyclic ketone **30** (4.7 mg, 0.012 mmol, 35% yield) and ***epi*-30** (4.1 mg, 0.011 mmol, 30% yield) both as colorless liquids.

3.5. Experimental results for the synthesis of (−)-garsubellin A and comparison of optical rotation values

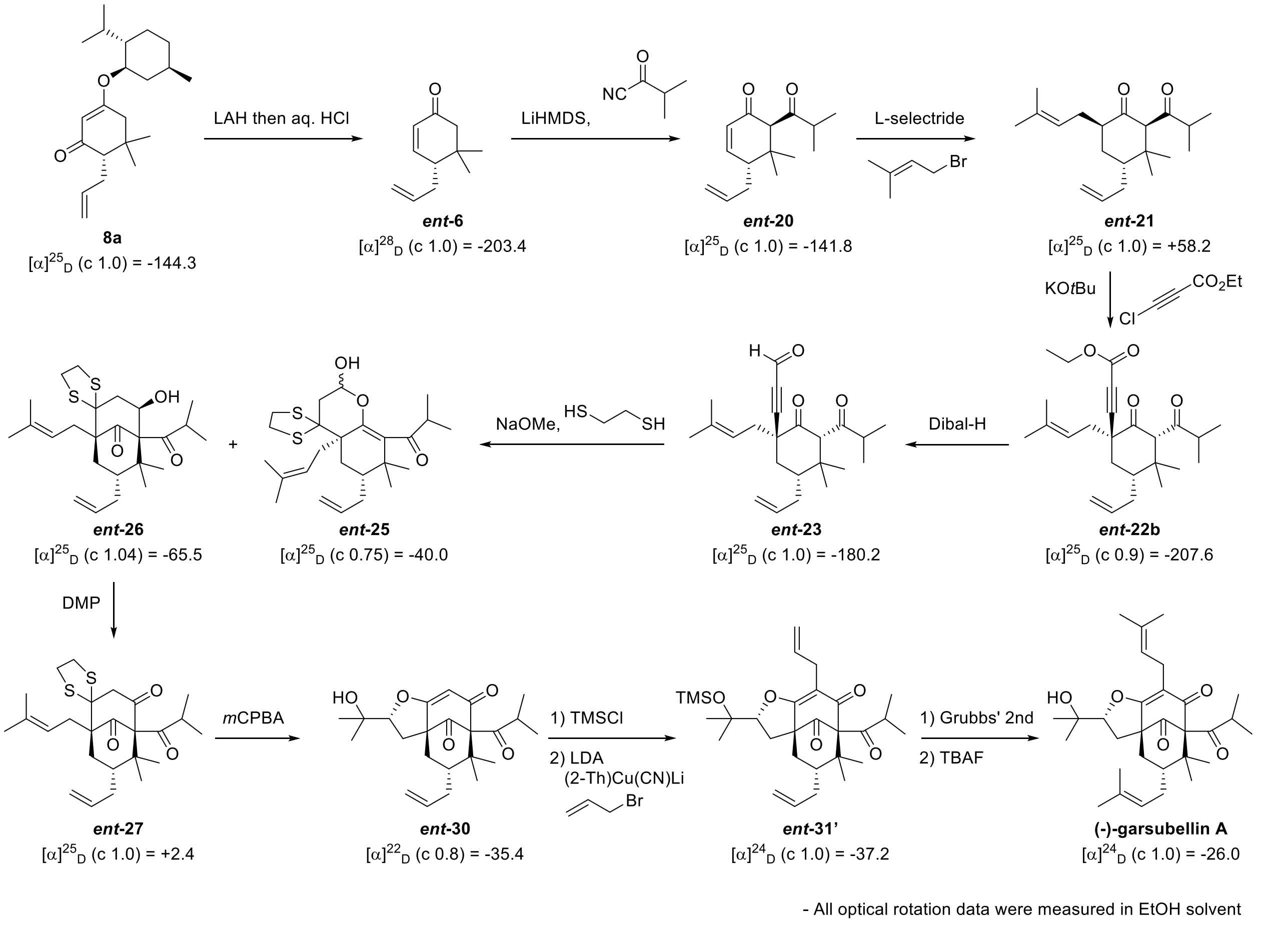

Scheme S7. Total synthesis of (−)-garsubellin A from isomer **8a**

4. Comparison with the reported spectral data of garsubellin A

<Numbering of garsubellin A>

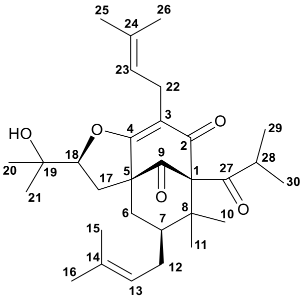

Comparison of the NMR spectral data in benzene-d6

Atom number	This work (400 MHz, 100 MHz)		Maimone's work (700 MHz, 175 MHz)		Natural (isolation) (400 MHz, 100 MHz)	
	^{13}C	^{1}H	^{13}C	^{1}H	^{13}C	^{1}H
**1**	82.7		82.7		82.6	
**2**	192.9		193.0		192.9	
**3**	116.8		116.7		116.7	
**4**	173.2		173,2		173.2	
**5**	59.9		59.9		59.9	
**6**	39.1	1.28 – 1.26 (m, 1H)<br>1.31 – 1.29 (m, 1H)	39.1	1.26 (m, 1H)<br>1.29 – 1.27 (m, 1H)	39.0	1.30 (dd, J = 11.3, 13.6 Hz, 1H)<br>1.32 (dd, J = 5.9, 12.9 Hz, 1H)
**7**	43.1	1.80 – 1.72 (m, 1H)	43.1	1.75 (dddd, J = 12.5, 10.5, 4.6, 2.8 Hz, 1H)	43.0	1.74 (dddd, J = 3.6, 4.5, 7.1, 11.3 Hz, 1H)
**8**	46.7		46.7		46.6	
**9**	204.7		204.7		204.7	
**10**	16.6	1.24 (s, 3H)	16.5	1.25 (s, 3H)	16.5	1.24 (s, 3H)
**11**	23.2	1.60 (s, 6H)	23.2	1.61 (s, 6H)	23.1	1.60 (s, 3H)
**12**	27.1	1.56 – 1.50 (m, 1H)<br>2.13 – 2.04 (m, 1H)	27.1	1.57 – 1.53 (m, 1H)<br>2.15 – 2.02 (m, 1H)	27.0	1.58 (m, 1H)<br>2.09 (ddd, J = 3.6, 7.1, 13.4 Hz, 1H)
**13**	123.3	5.00 – 4.92 (m, 1H)	123.3	5.07 – 4.89 (m, 1H)	123.2	4.96 (dd, J = 7.1, 7.1 Hz, 1H)
**14**	133.2		133.2		133.2	
**15**	17.93	1.44 (s, 3H)	17.93	1.45 (s, 3H)	17.8	1.45 (s, 3H)
**16**	26.0	1.57 (s, 3H)	26.0	1.58 (s, 3H)	25.9	1.58 (s, 3H)
**17**	30.3	1.92 (dd, J = 13.5, 4.4 Hz, 1H)<br>2.72 (dd, J = 13.0, 10.8 Hz, 1H)	30.3	1.93 (dd, J = 13.6, 4.5 Hz, 1H)<br>2.73 (dd, J = 13.0, 10.7 Hz, 1H)	30.3	1.93 (dd, J = 4.5, 13.6 Hz, 1H)<br>2.73 (dd, J = 10.7, 12.9 Hz, 1H)
**18**	90.2	3.91 (dd, J = 10.6, 5.8 Hz, 1H)	90.2	3.91 (dd, J = 10.7, 5.8 Hz, 1H)	90.1	3.92 (dd, J = 5.9, 10.7 Hz, 1H)
**19**	70.3		70.3		70.2	
**20**	26.4	0.93 (s, 3H)	26.4	0.93 (s, 3H)	26.3	0.94 (s, 3H)
**21**	24.5	0.76 (s, 3H)	24.5	0.77 (s, 3H)	24.4	0.77 (s, 3H)
**22**	22.7	3.20 (dd, J = 14.0, 7.5 Hz, 1H)<br>3.38 (dd, J = 14.0, 7.0 Hz, 1H)	22.7	3.21 (dd, J = 14.2, 7.6 Hz, 1H)<br>3.39 (dd, J = 14.2, 7.2 Hz, 1H)	22.6	3.21 (dd, J = 7.3, 14.2 Hz, 1H)<br>3.39 (dd, J = 7.1, 14.2 Hz, 1H)
**23**	122.1	5.39 (t, J = 7.1 Hz, 1H)	122.1	5.40 (ddt, J = 7.4, 5.9, 1.4 Hz, 1H)	122.0	5.40 (dd, J = 7.1, 7.3 Hz, 1H)
**24**	132.5		132.5		132.4	
**25**	17.91	1.69 (s, 3H)	17.90	1.70 (d, J = 1.3 Hz, 3H)	17.9	1.70 (s, 3H)
**26**	25.8	1.60 (s, 6H)	25.8	1.61 (s, 6H)	25.7	1.61 (s, 3H)
**27**	208.5		208.5		208.5	
**28**	42.8	2.25 (dt, J = 12.9, 6.5 Hz, 1H)	42.8	2.26 (hept, J = 6.5 Hz, 1H)	42.7	2.26 (dq, J = 6.6 Hz, 1H)
**29**	22.0	1.29 (d, J = 6.5 Hz, 3H)	22.0	1.31 (d, J = 6.5 Hz, 3H)	21.9	1.30 (d, J = 6.6 Hz, 3H)
**30**	20.9	1.36 (d, J = 6.5 Hz, 3H)	20.9	1.37 (d, J = 6.5 Hz, 3H)	20.9	1.37 (d, J = 6.6 Hz, 3H)

Comparison of the ^{1}H NMR spectral data in benzene-d6

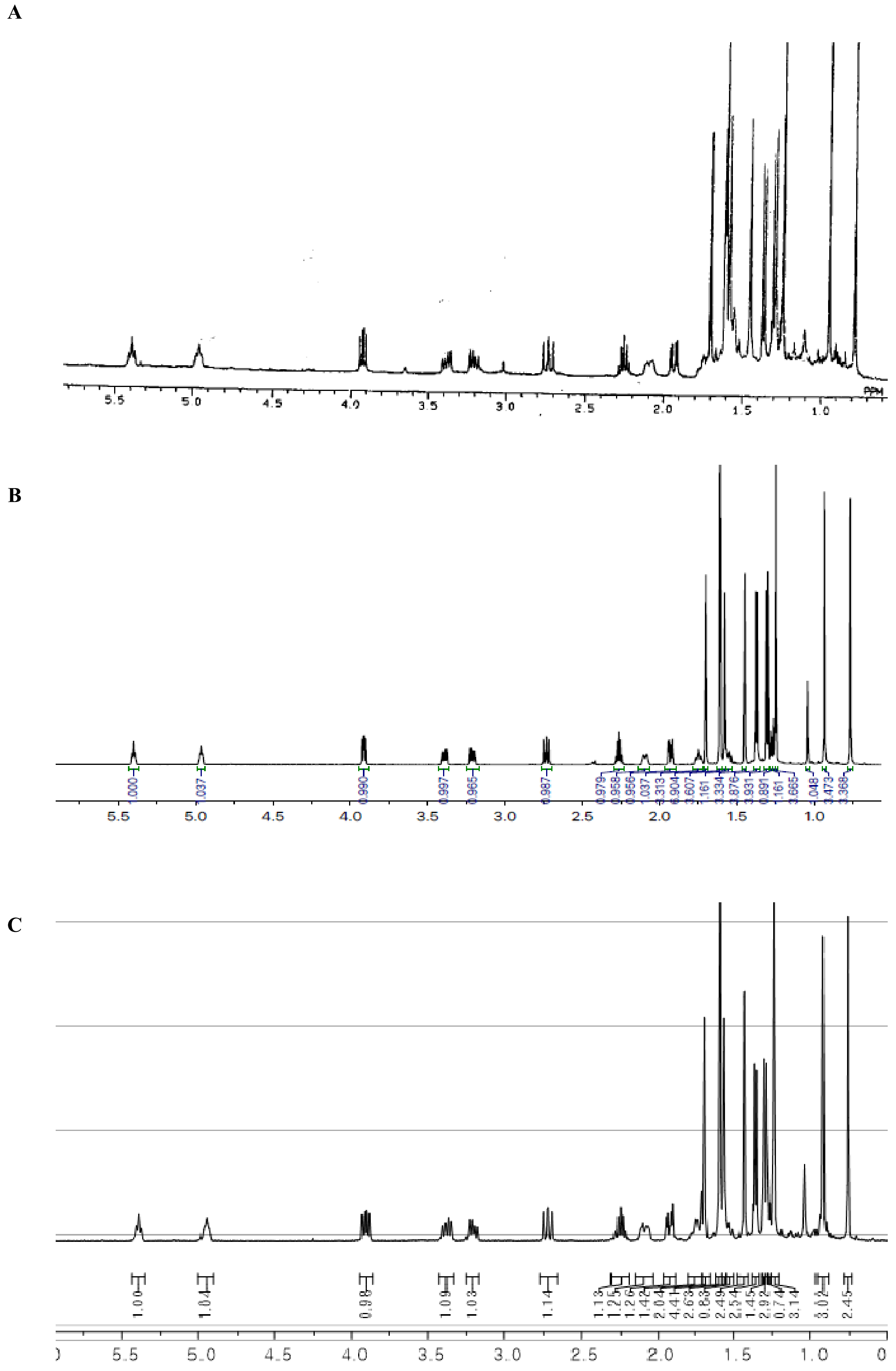

A: natural, **B**: Maimone's result, **C**: this work

5. ^{1}H and ^{13}C NMR spectra for synthesized compounds

Compound 7 ^{1}H NMR

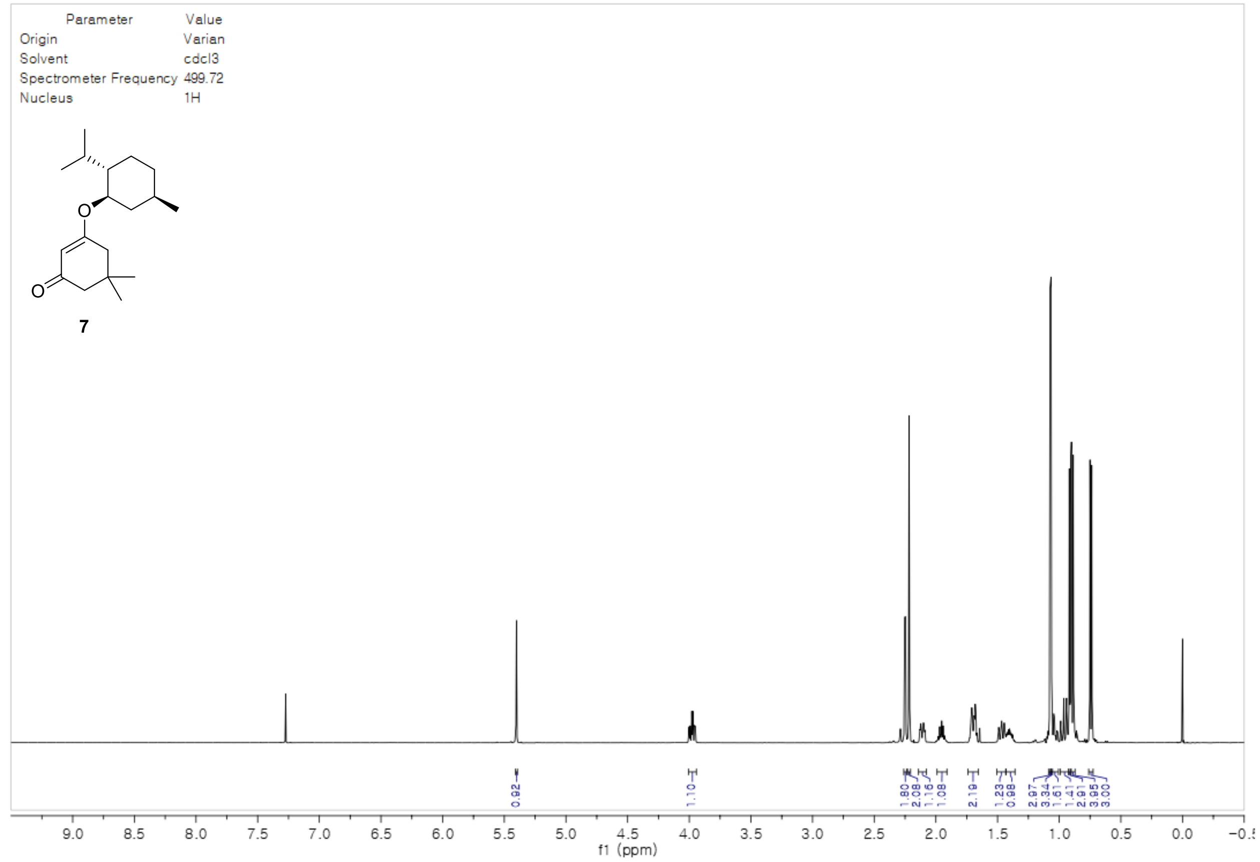

Compound 7 ^{13}C NMR

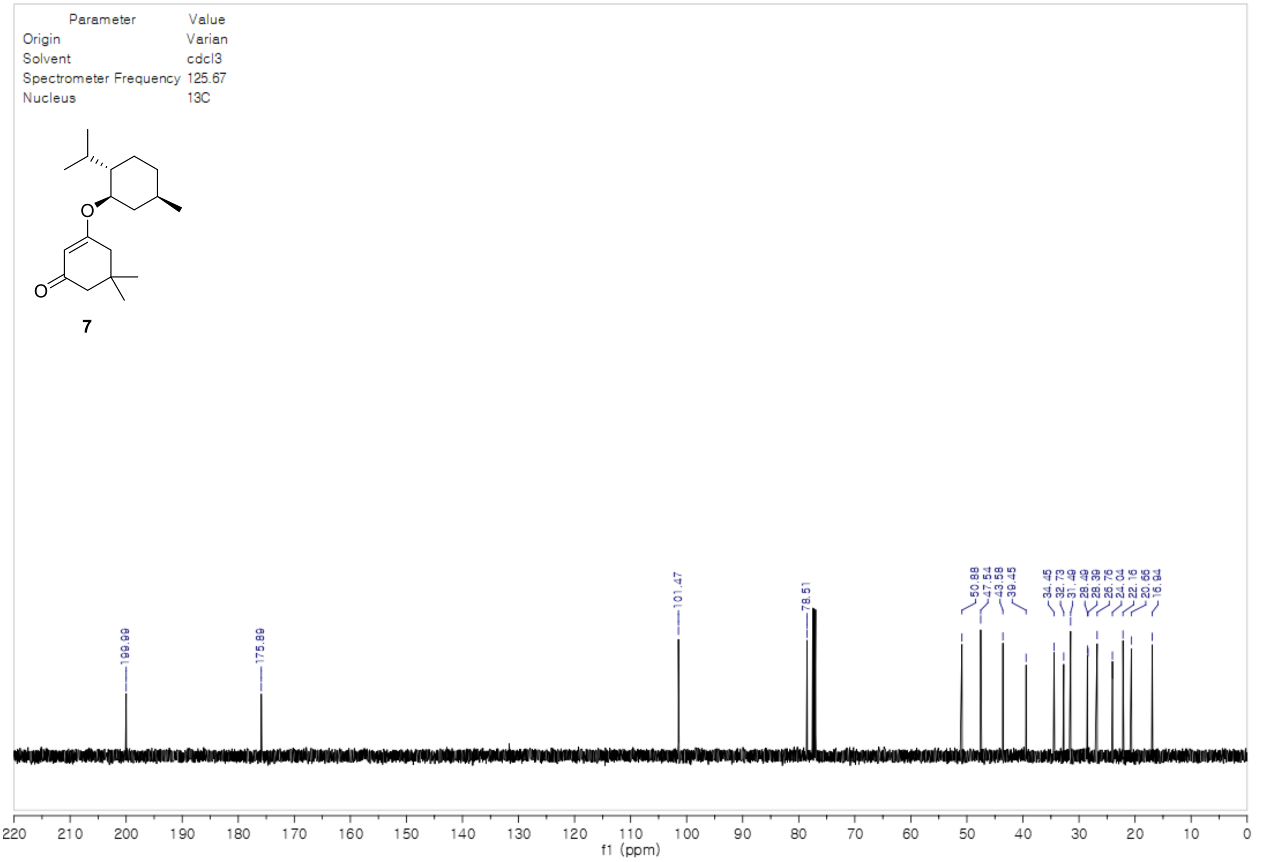

Compound 7' ^{1}H NMR

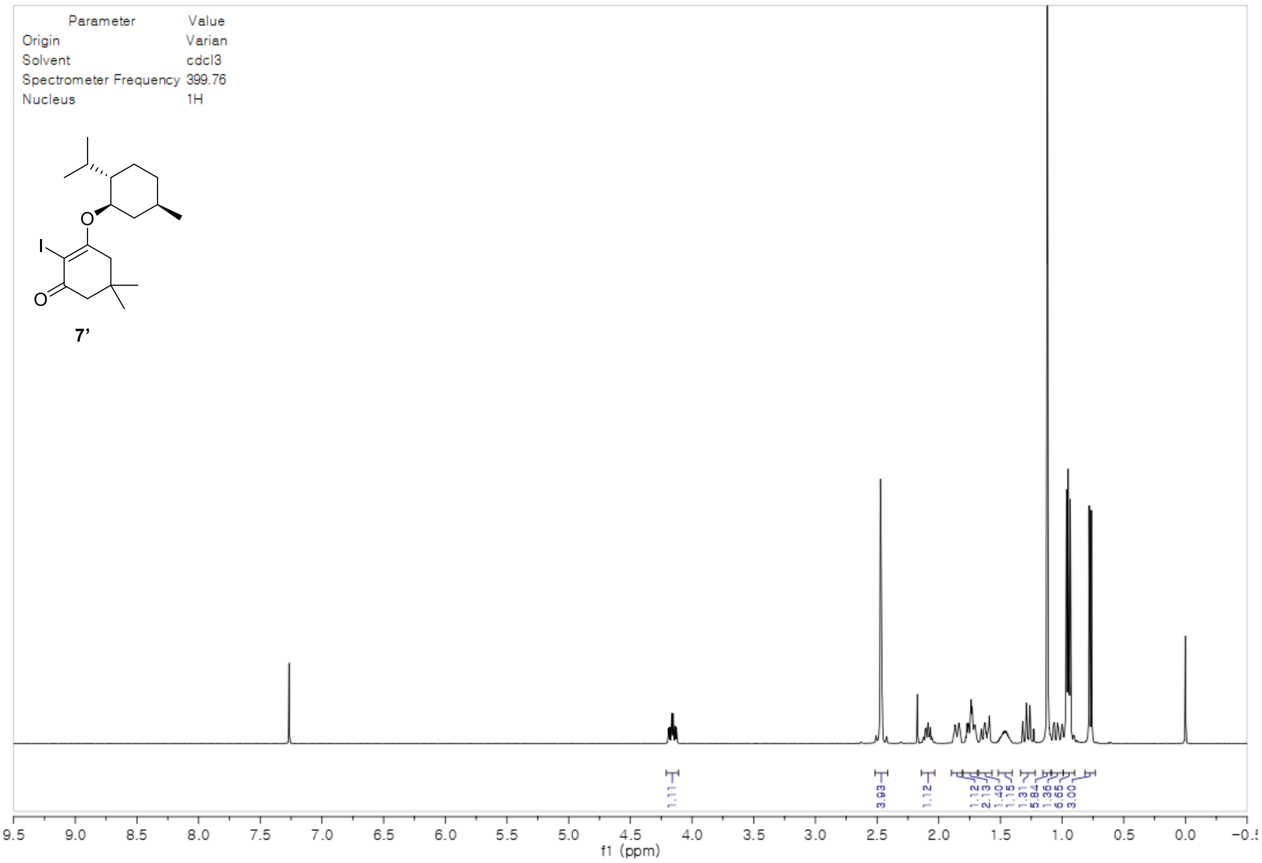

Compound 7' ^{13}C NMR

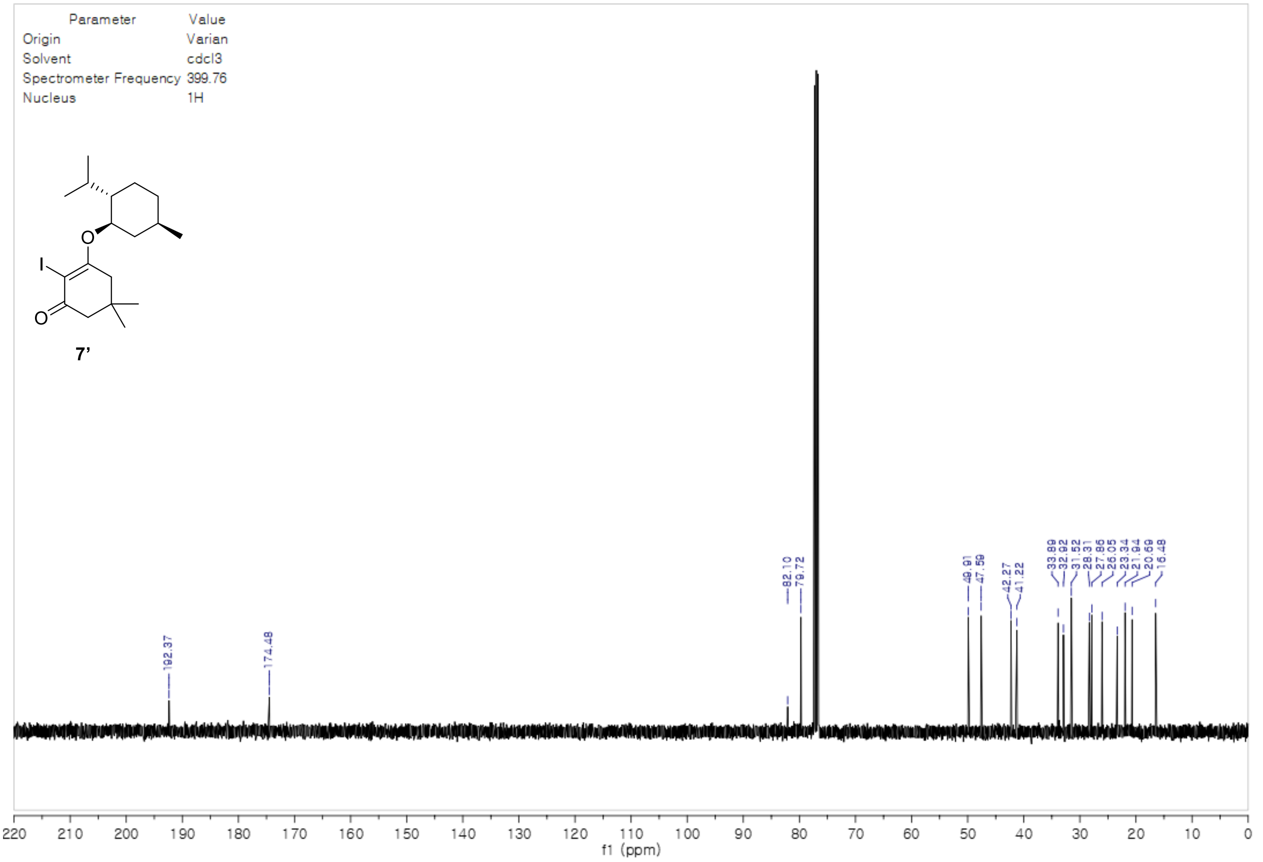

Compound 8c ^{1}H NMR

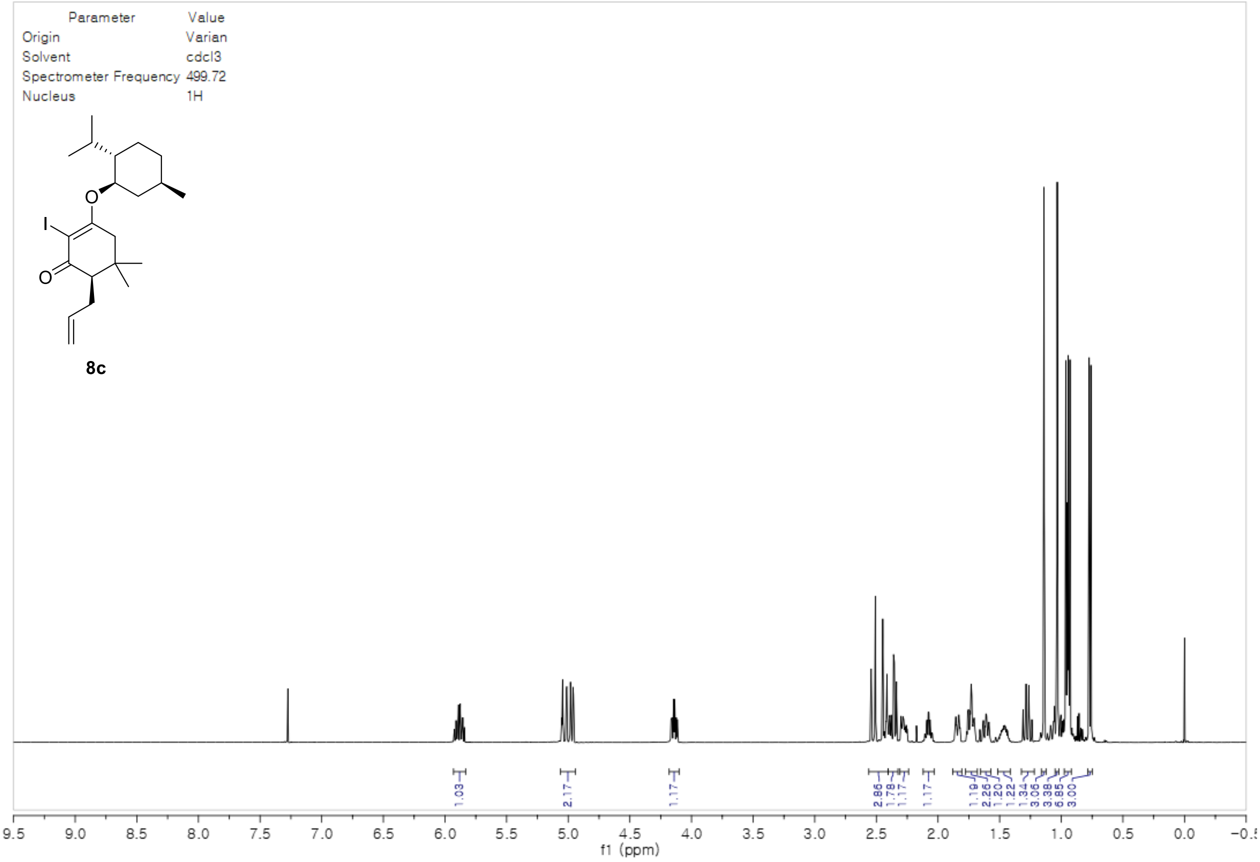

Compound 8c ^{13}C NMR

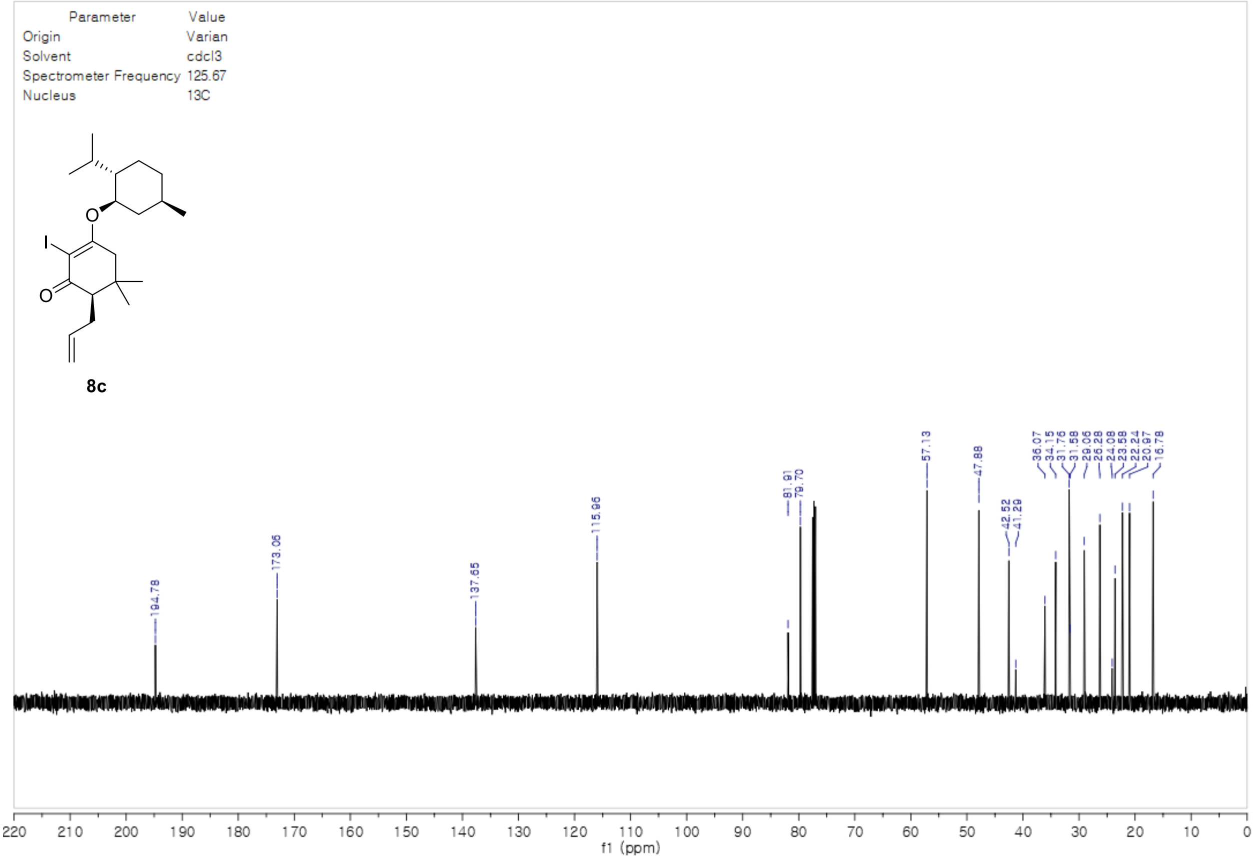

Compound 8d ¹H NMR

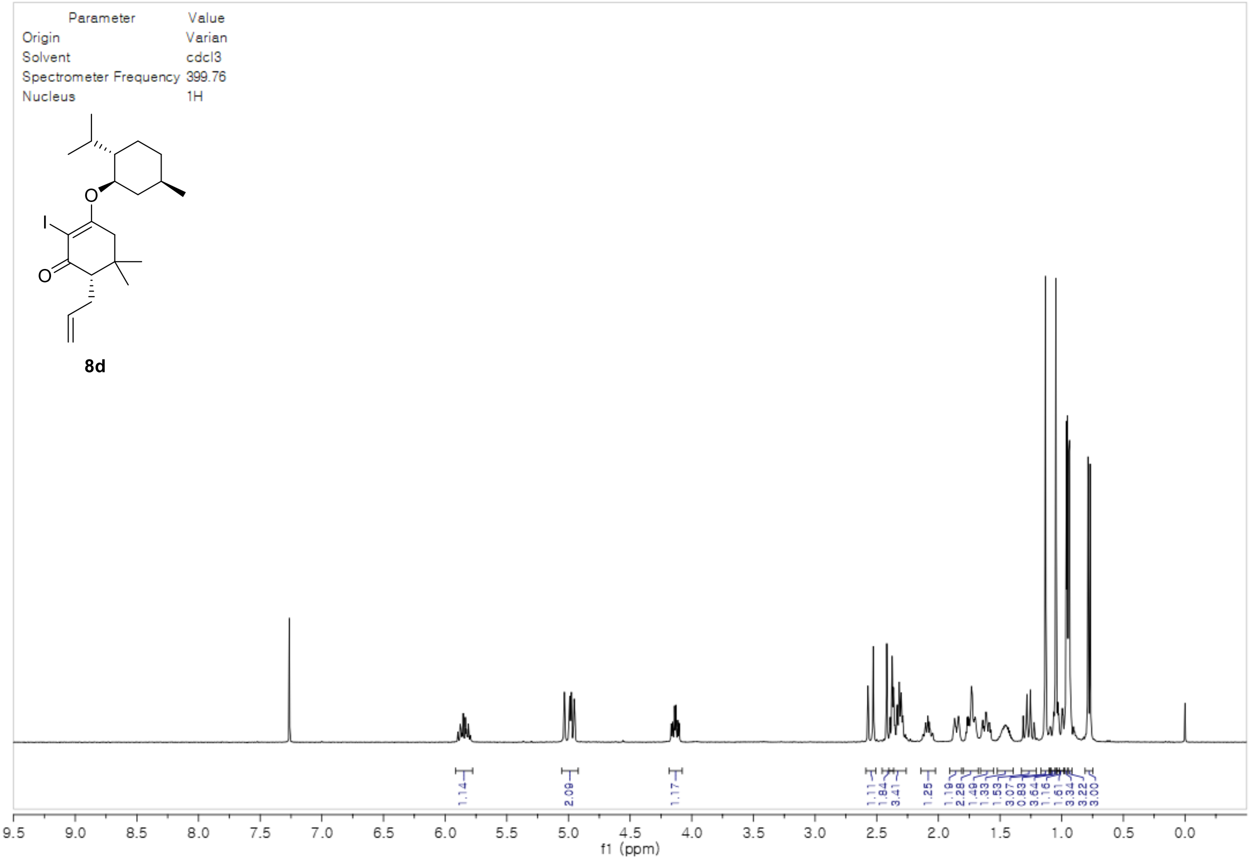

Compound 8d ¹³C NMR

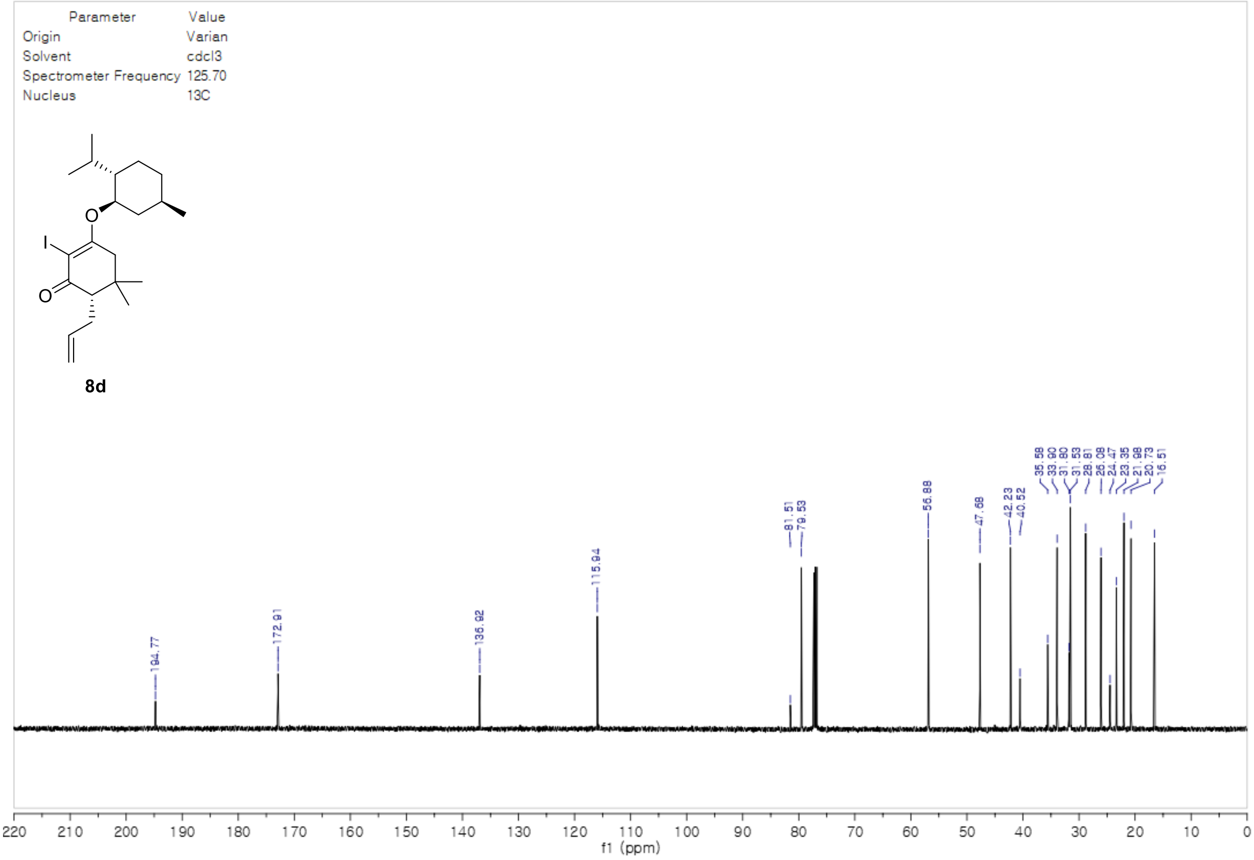

Compound 8b ^{1}H NMR

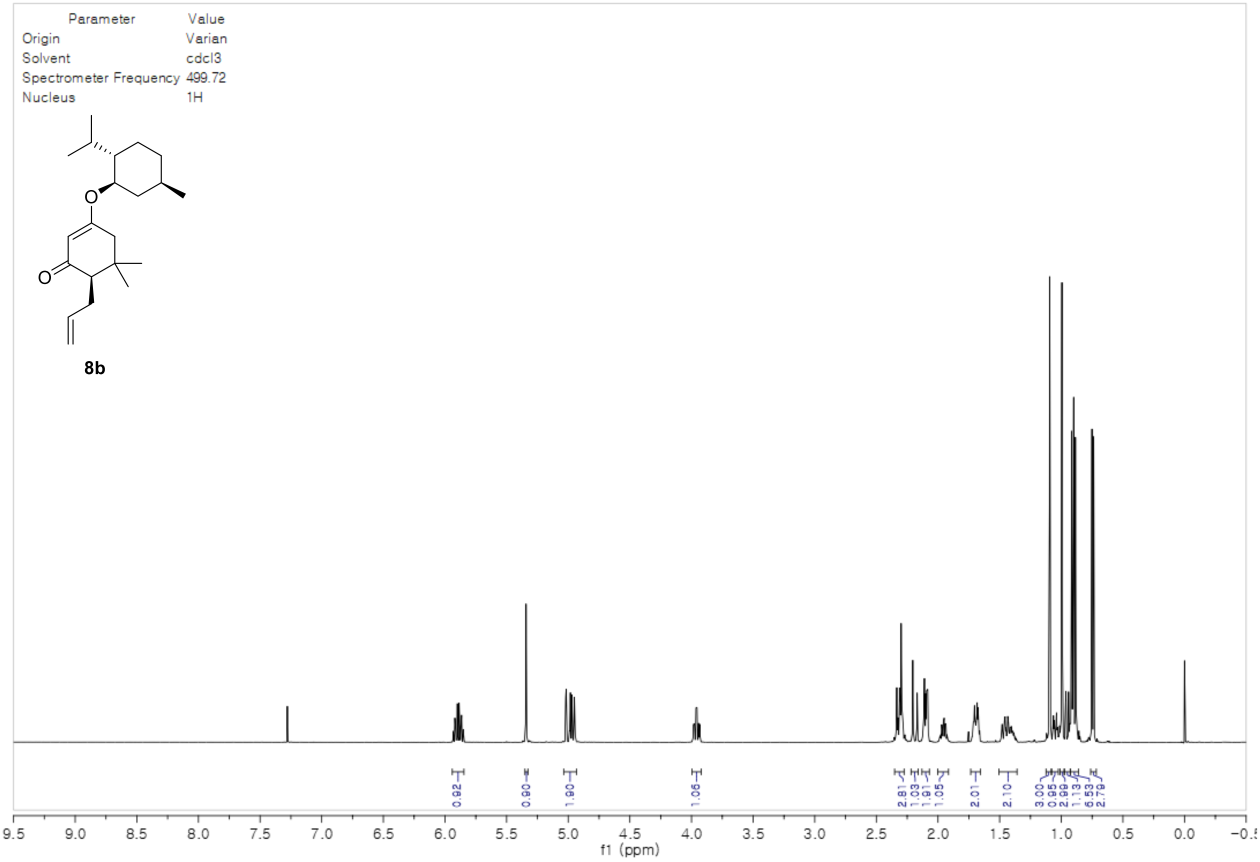

Compound 8b ^{13}C NMR

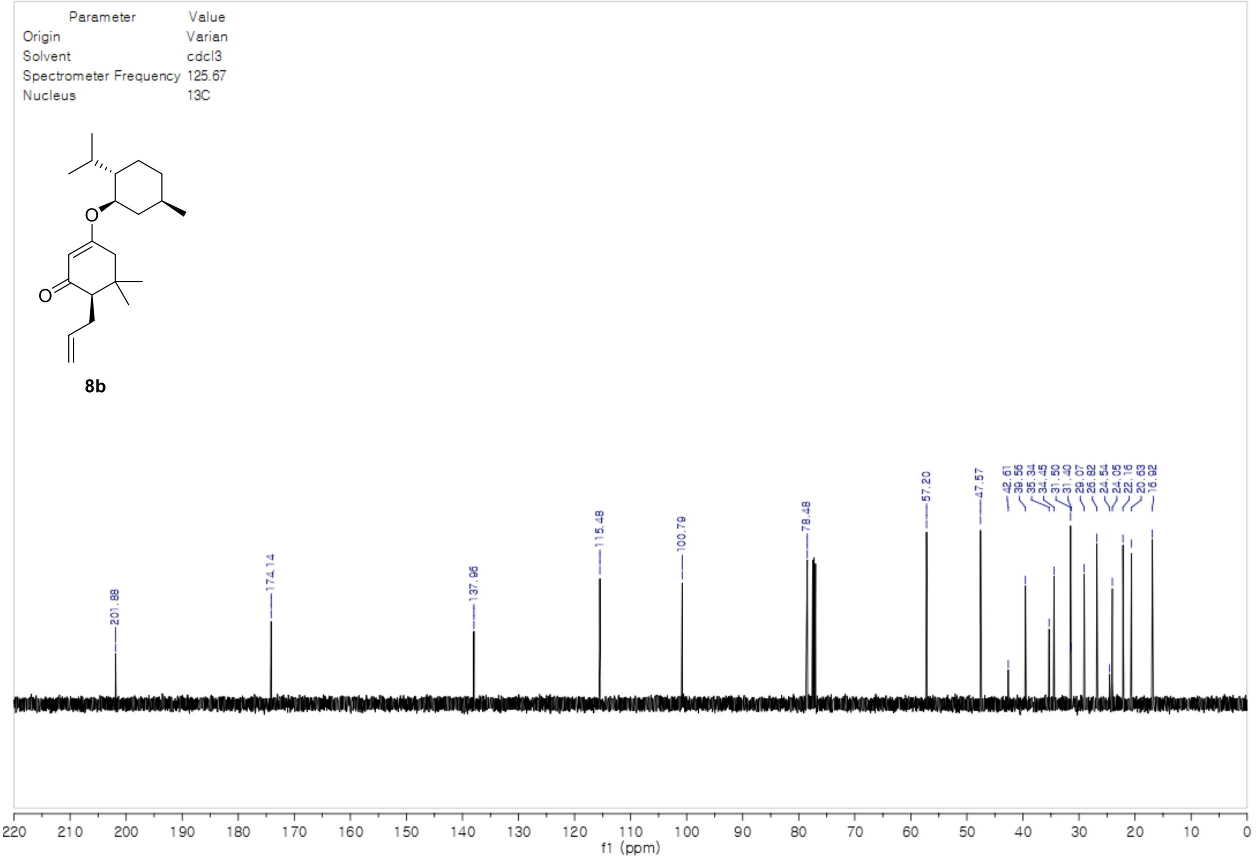

Compound 8a ^{1}H NMR

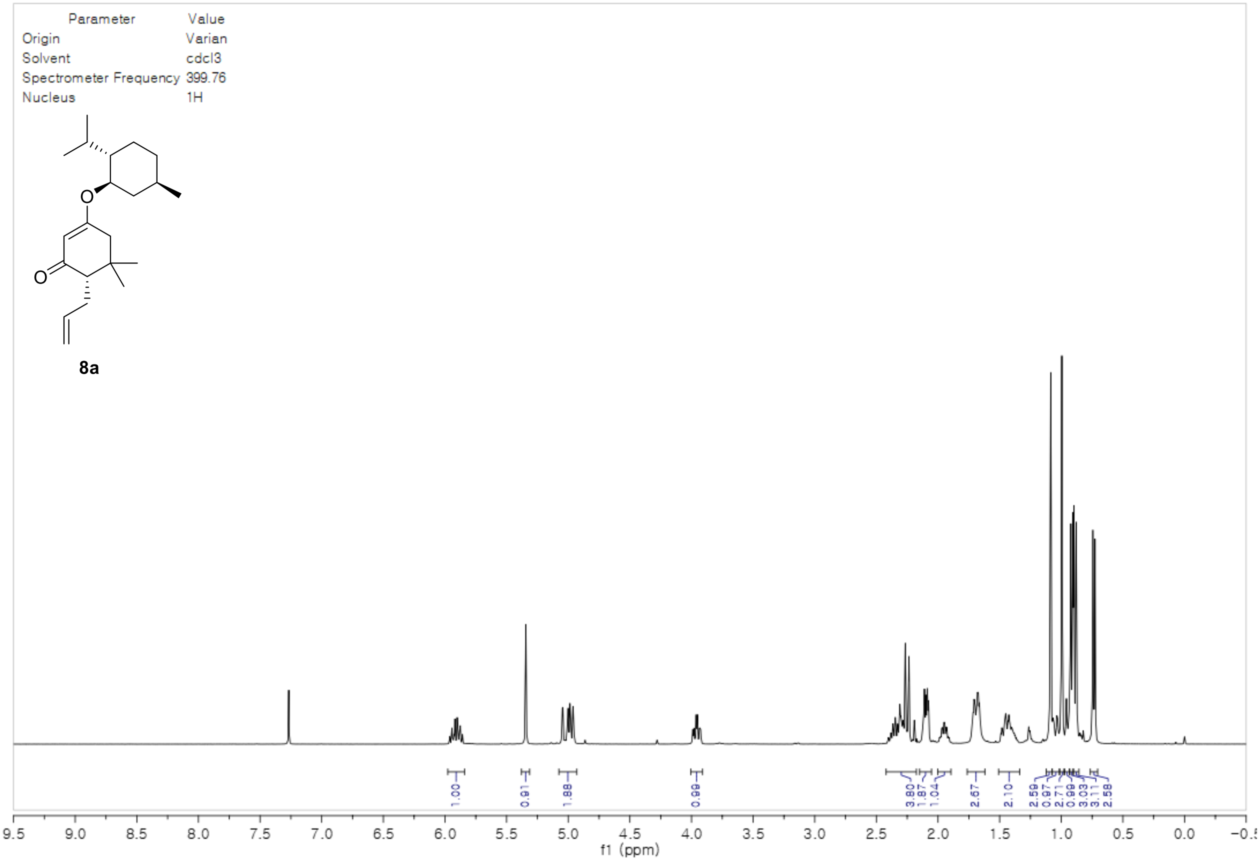

Compound 8a ^{13}C NMR

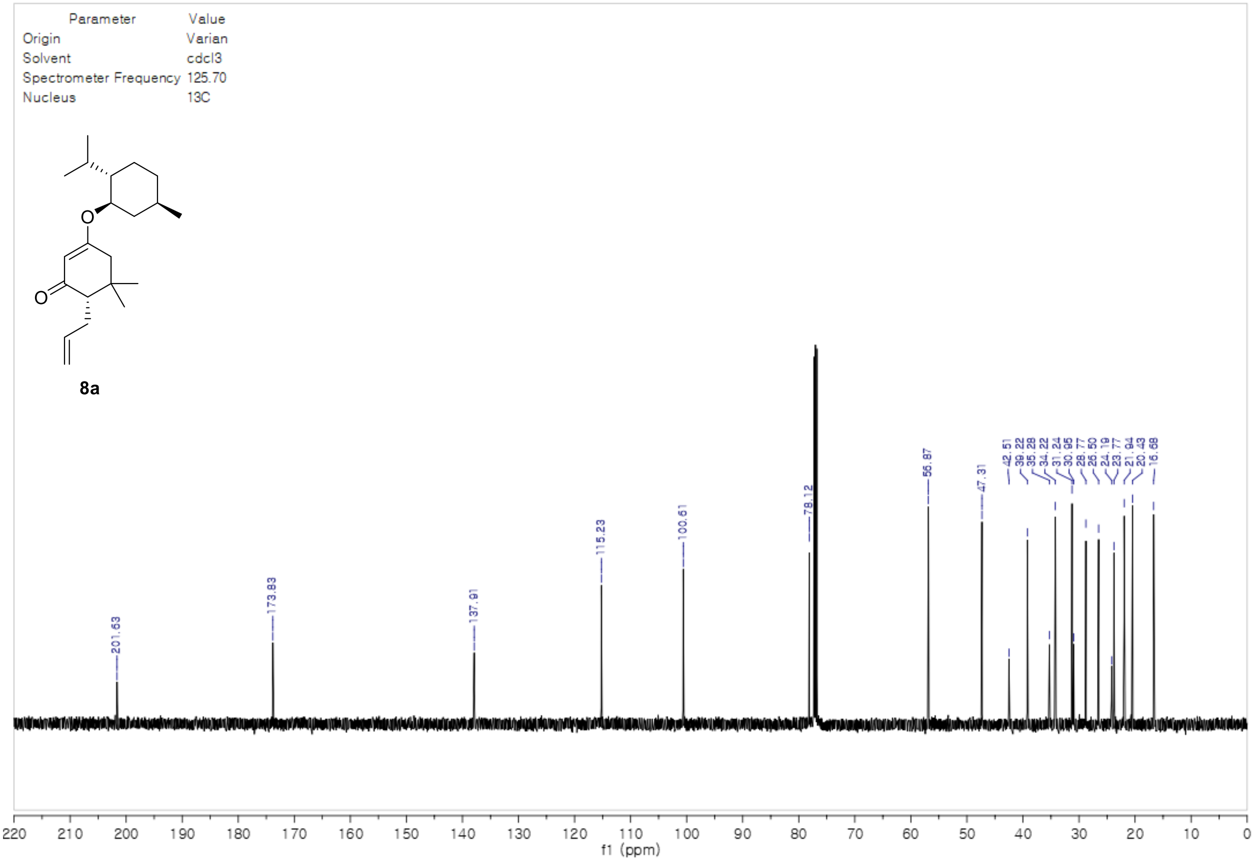

Compound 6 ^{1}H NMR

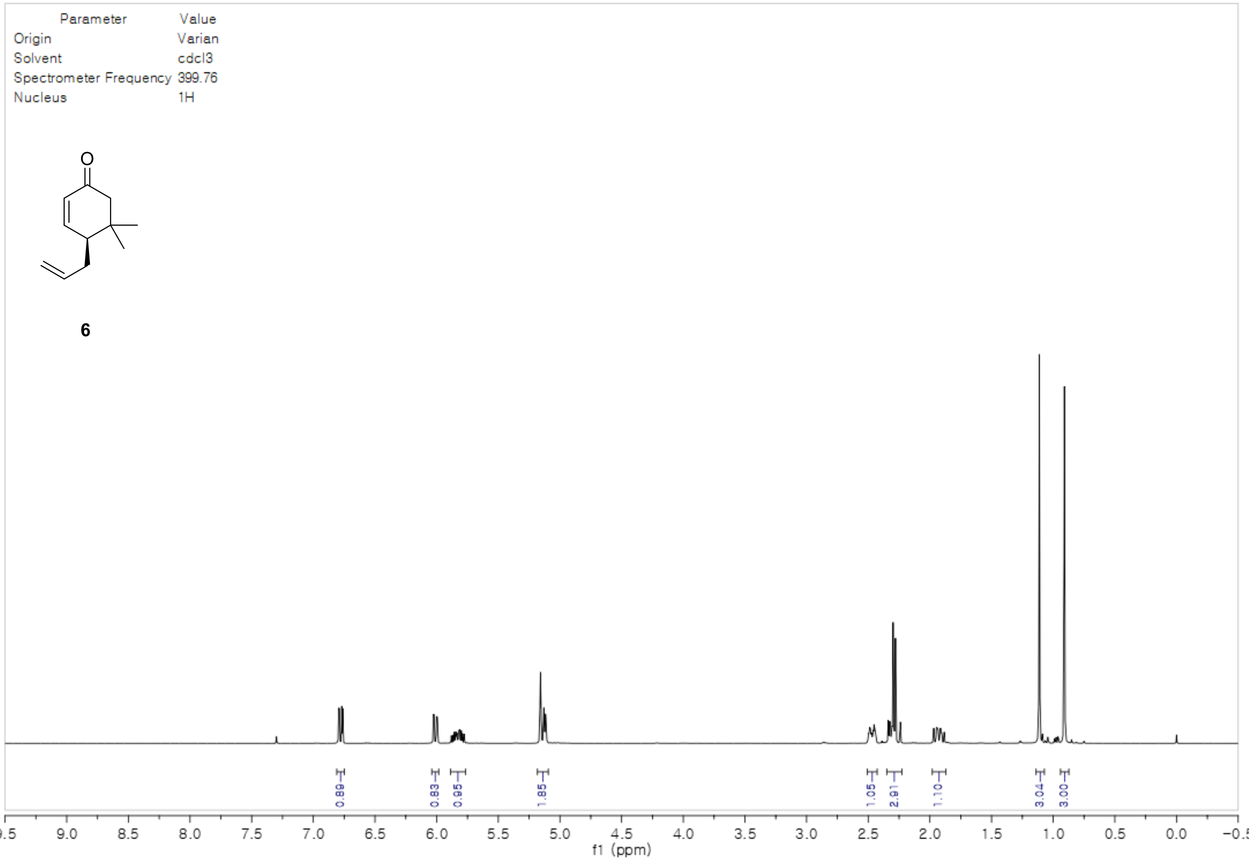

Compound 6 ^{13}C NMR

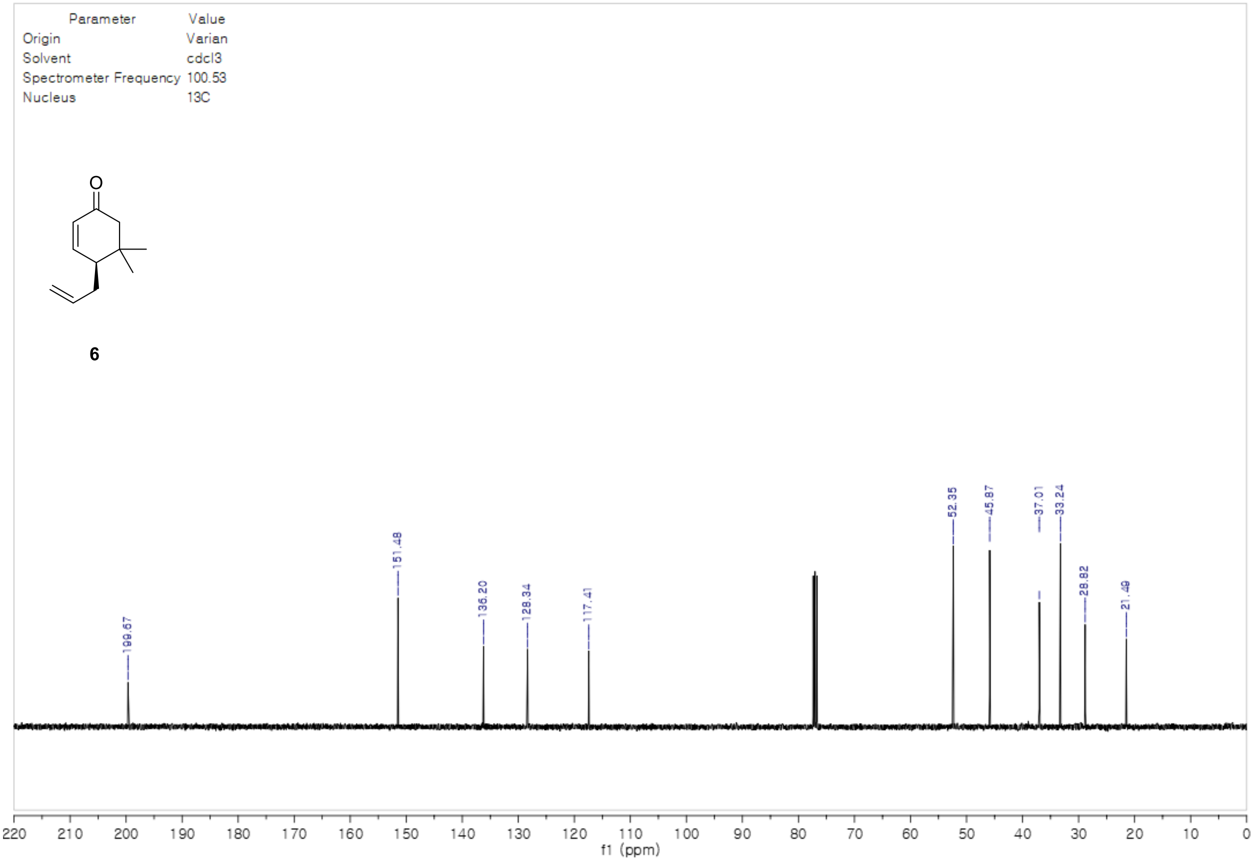

Compound 6'a ^{1}H NMR

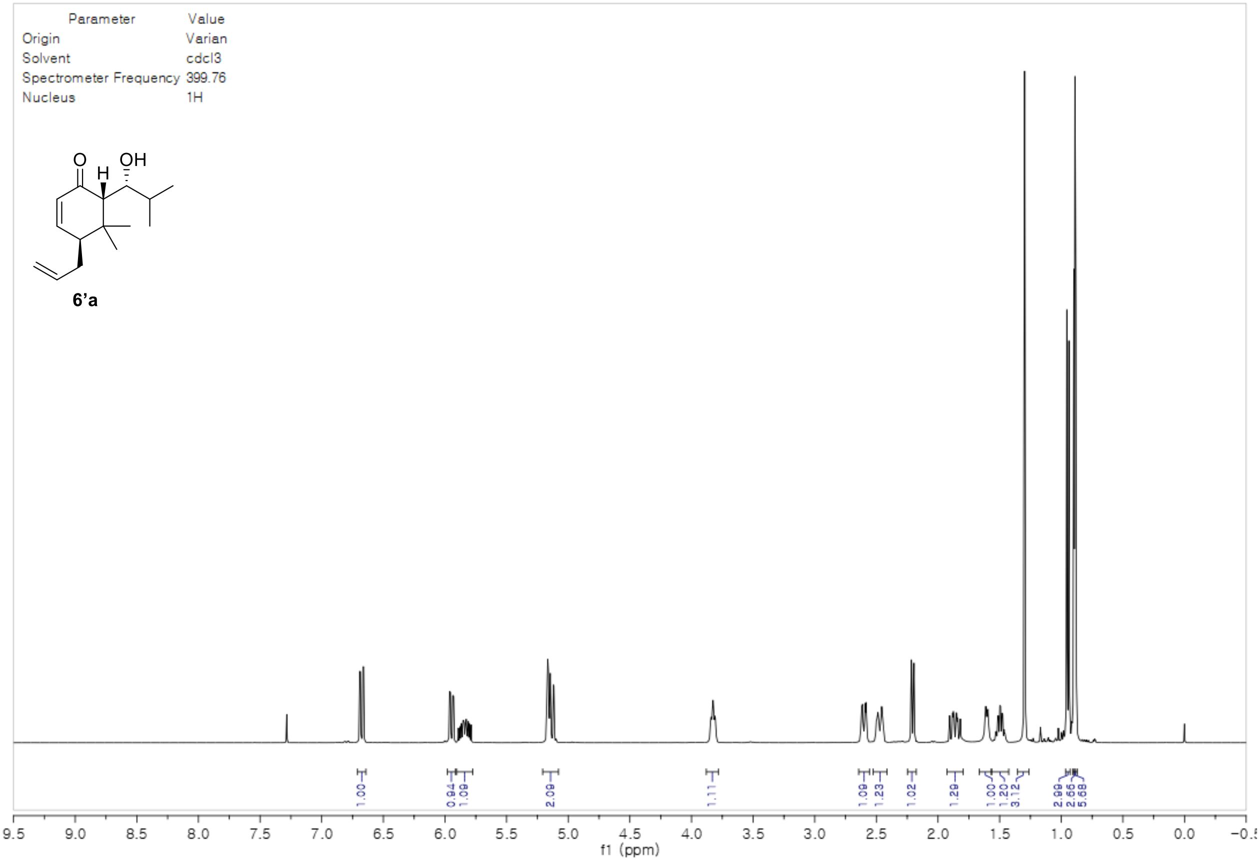

Compound 6'a ^{13}C NMR

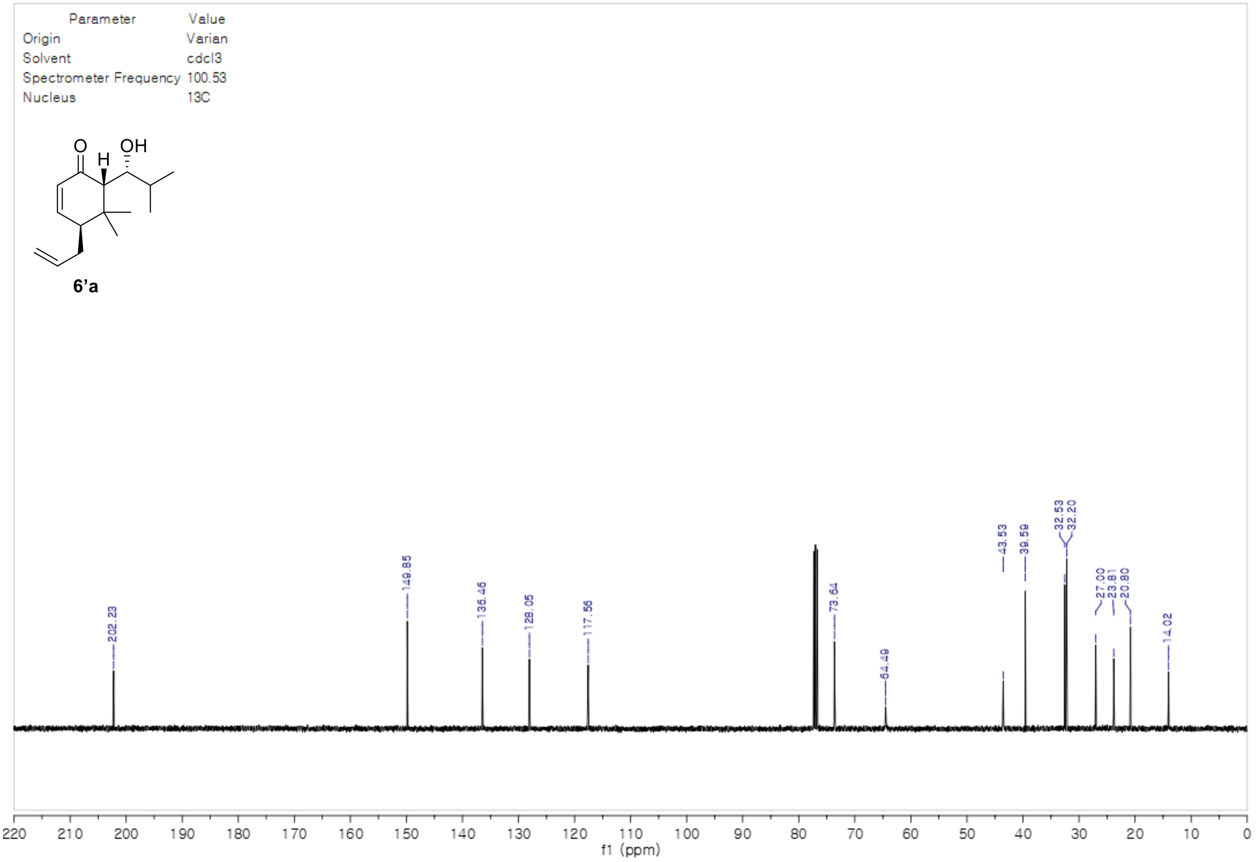

Compound 6’b ¹H NMR

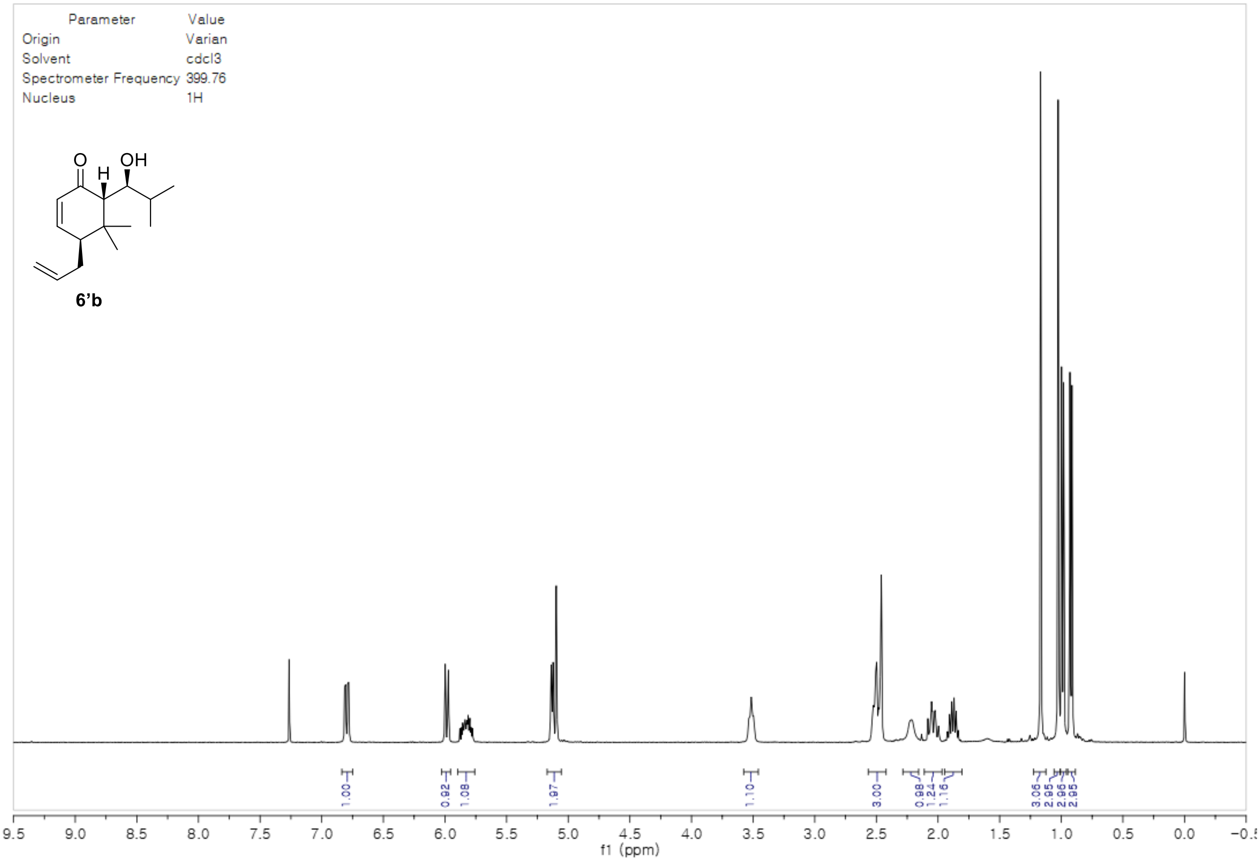

Compound 6’b ¹³C NMR

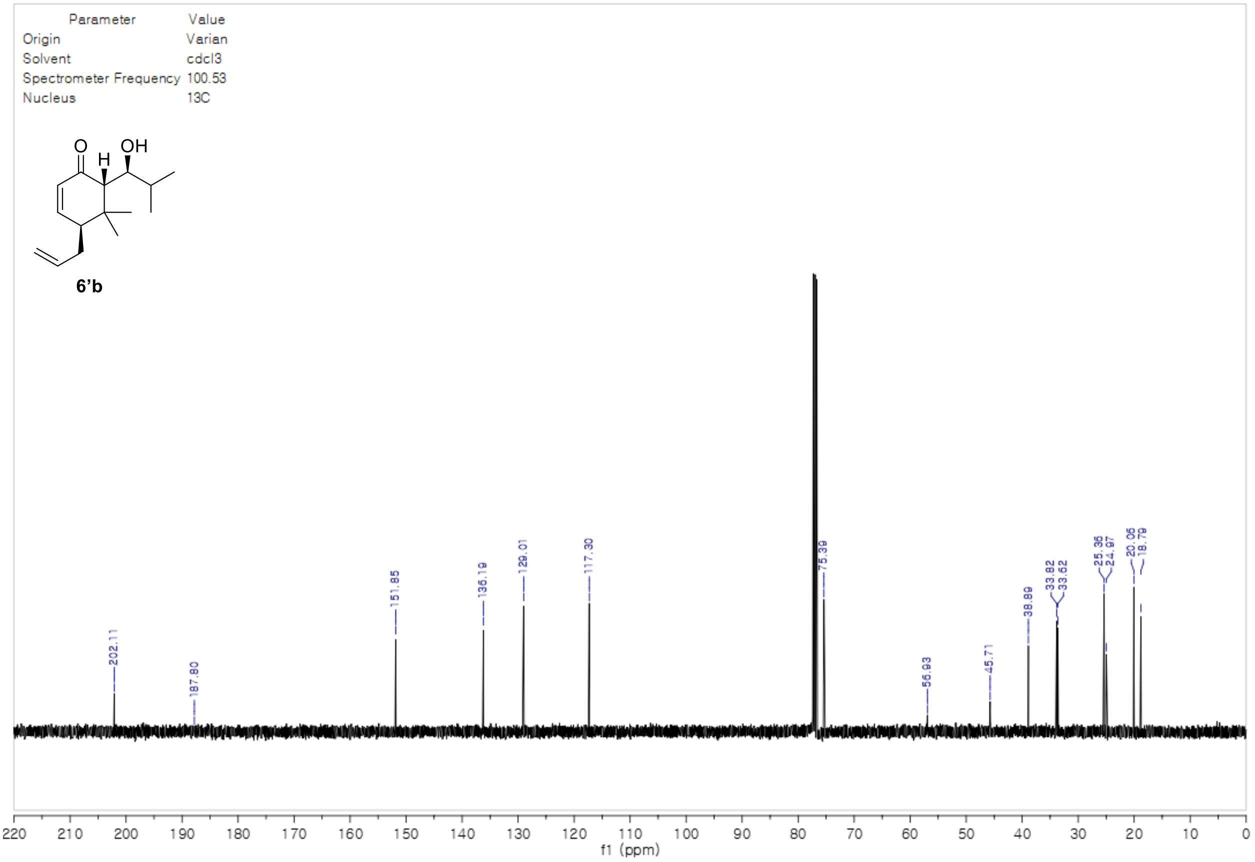

Compound (Z)-9 ^{1}H NMR

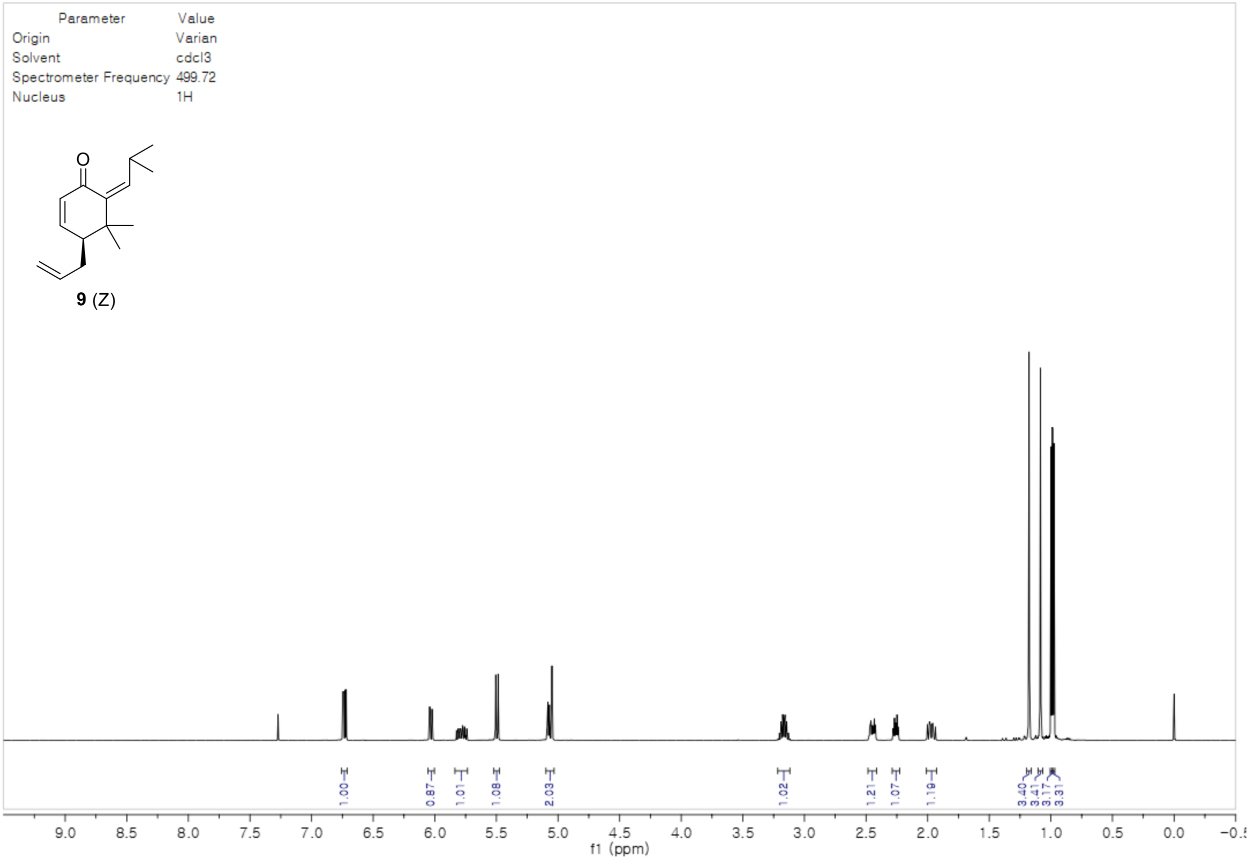

Compound (Z)-9 ^{13}C NMR

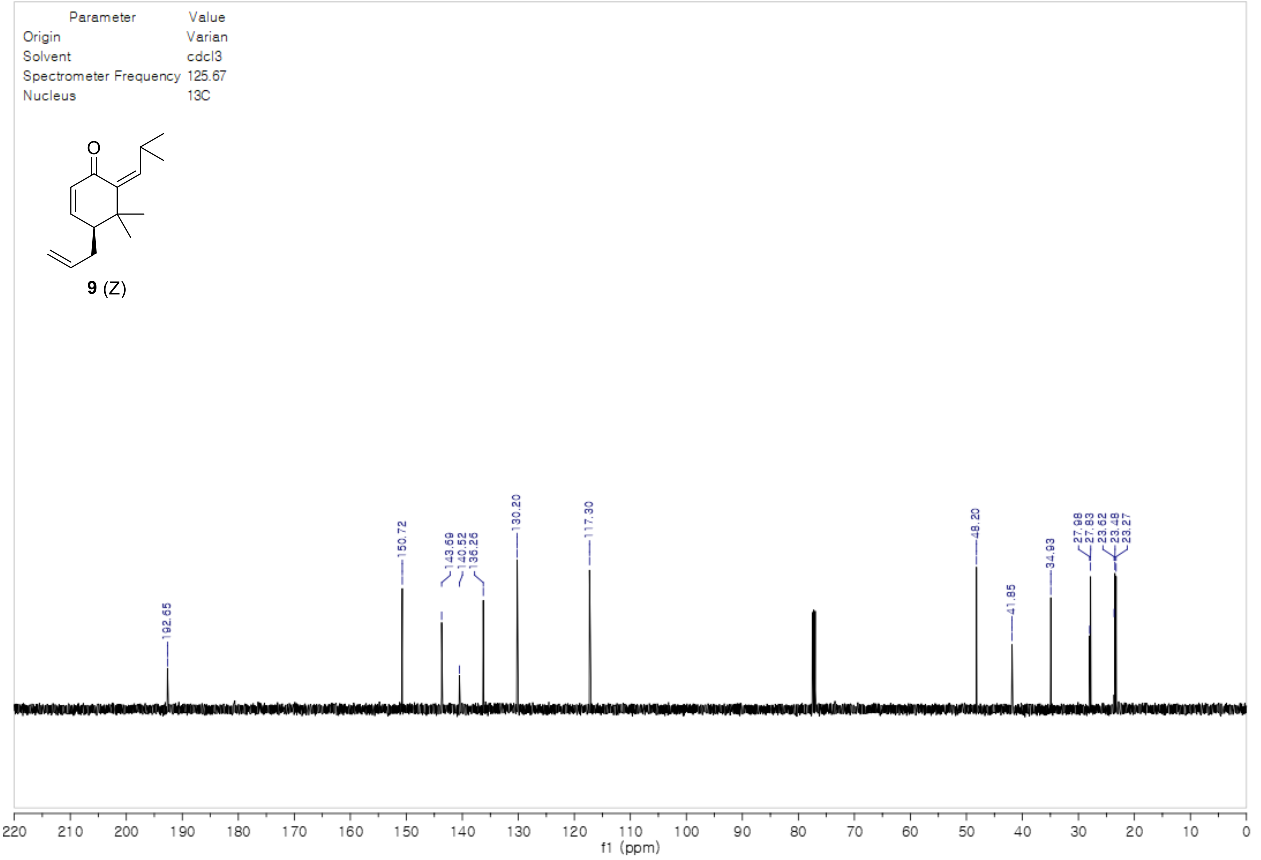

Compound (Z)-9 1D NOESY

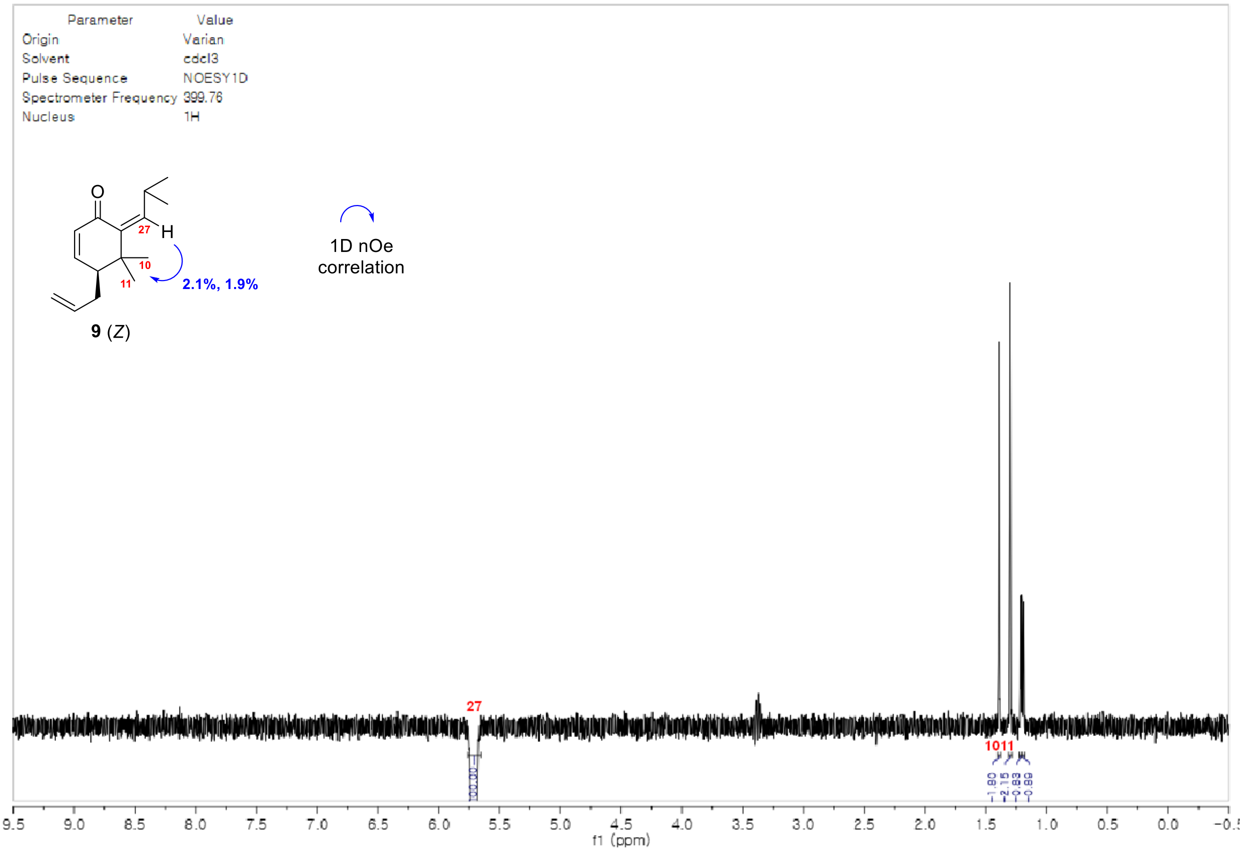

Compound (E)-9 ^{1}H NMR

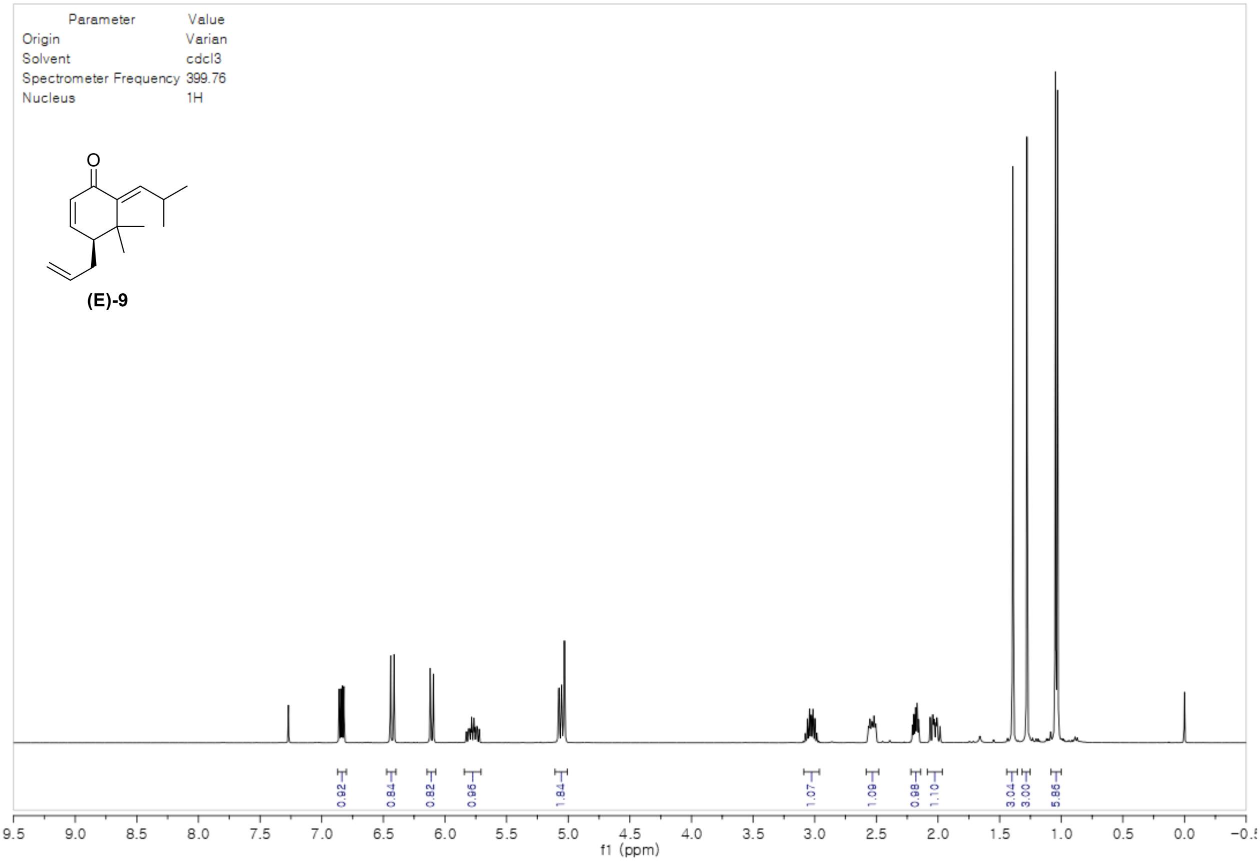

Compound (E)-9 ^{13}C NMR

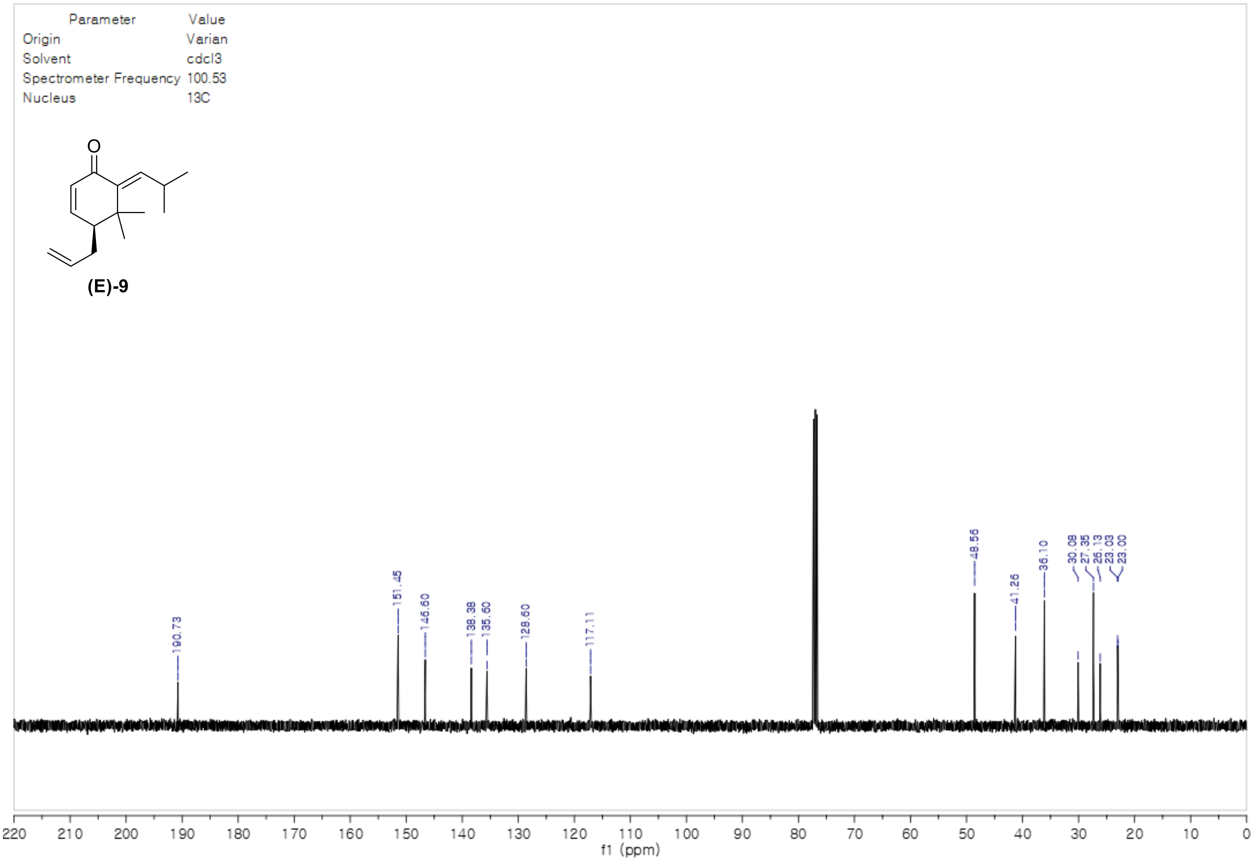

Compound (E)-9 1D NOESY

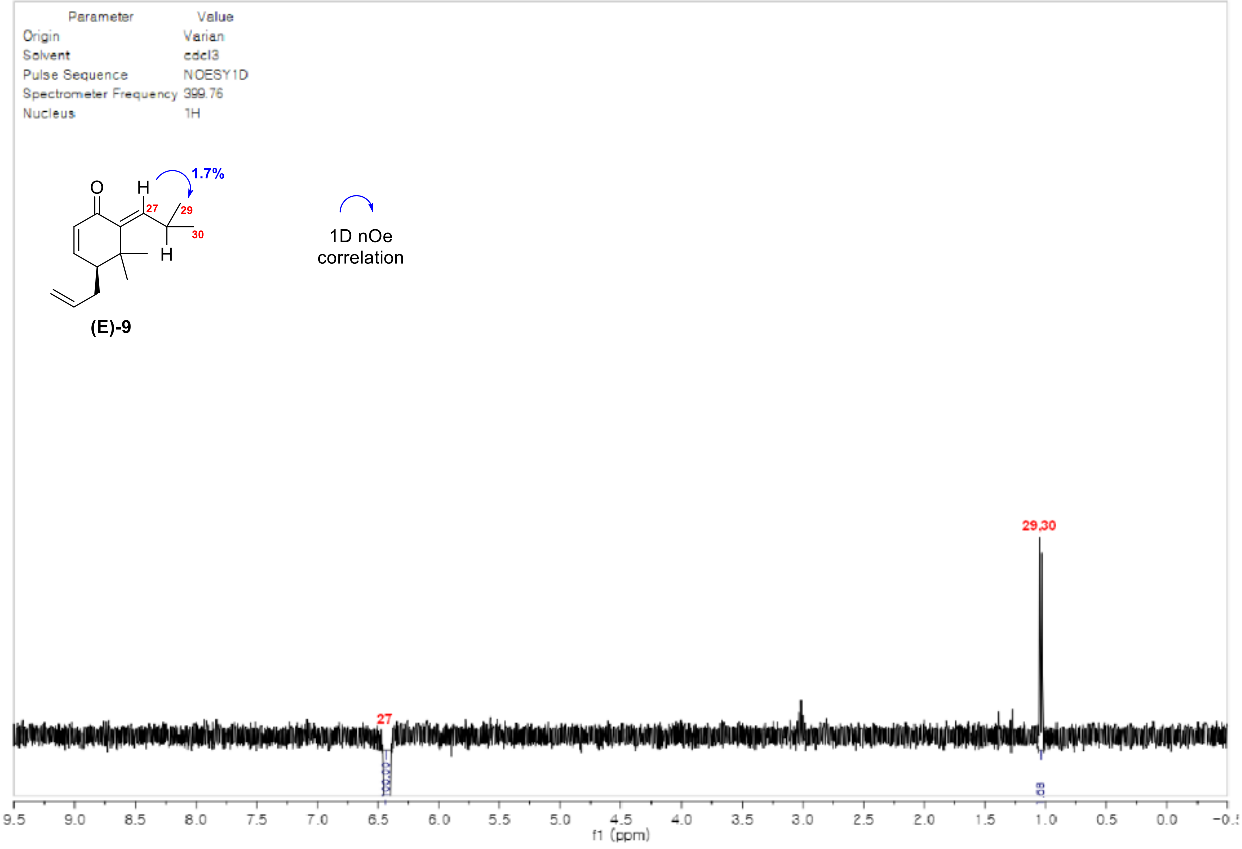

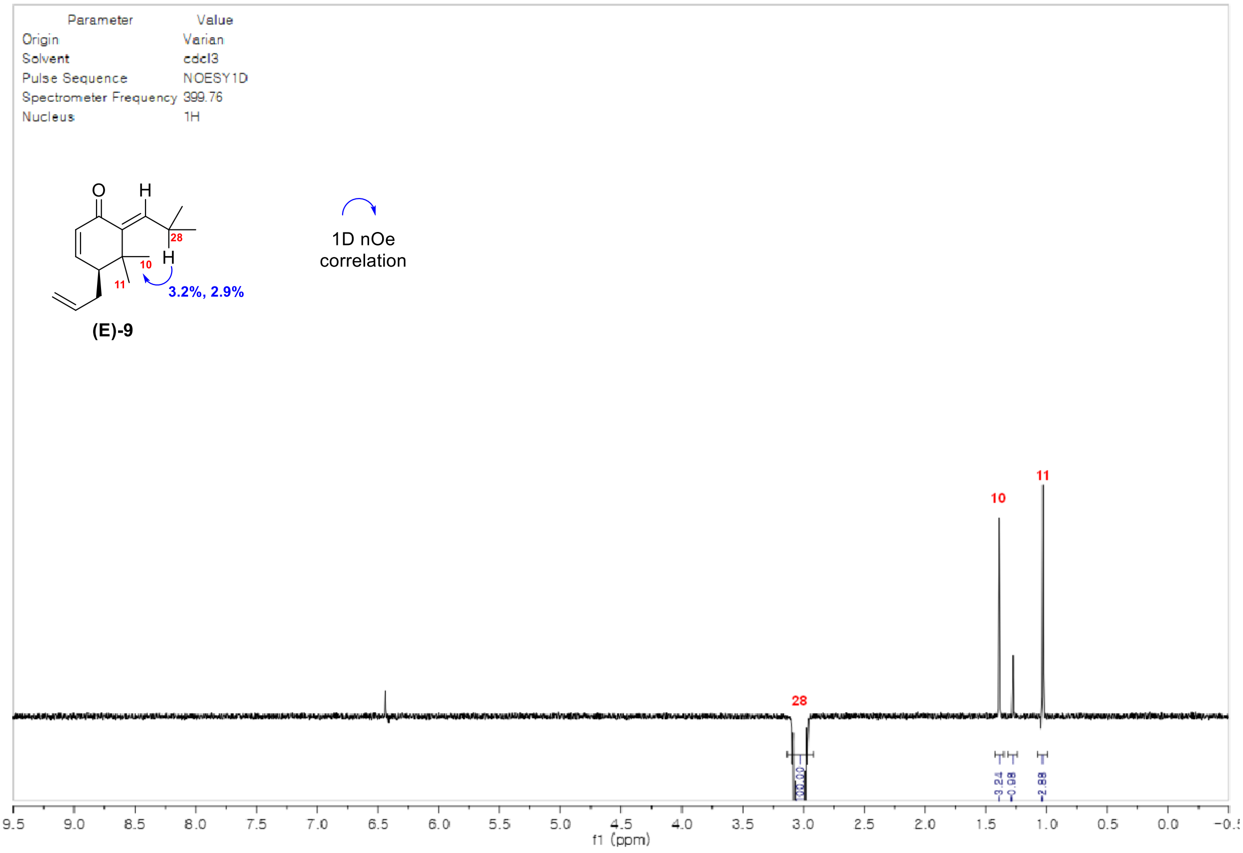

Compound 10 ^{1}H NMR

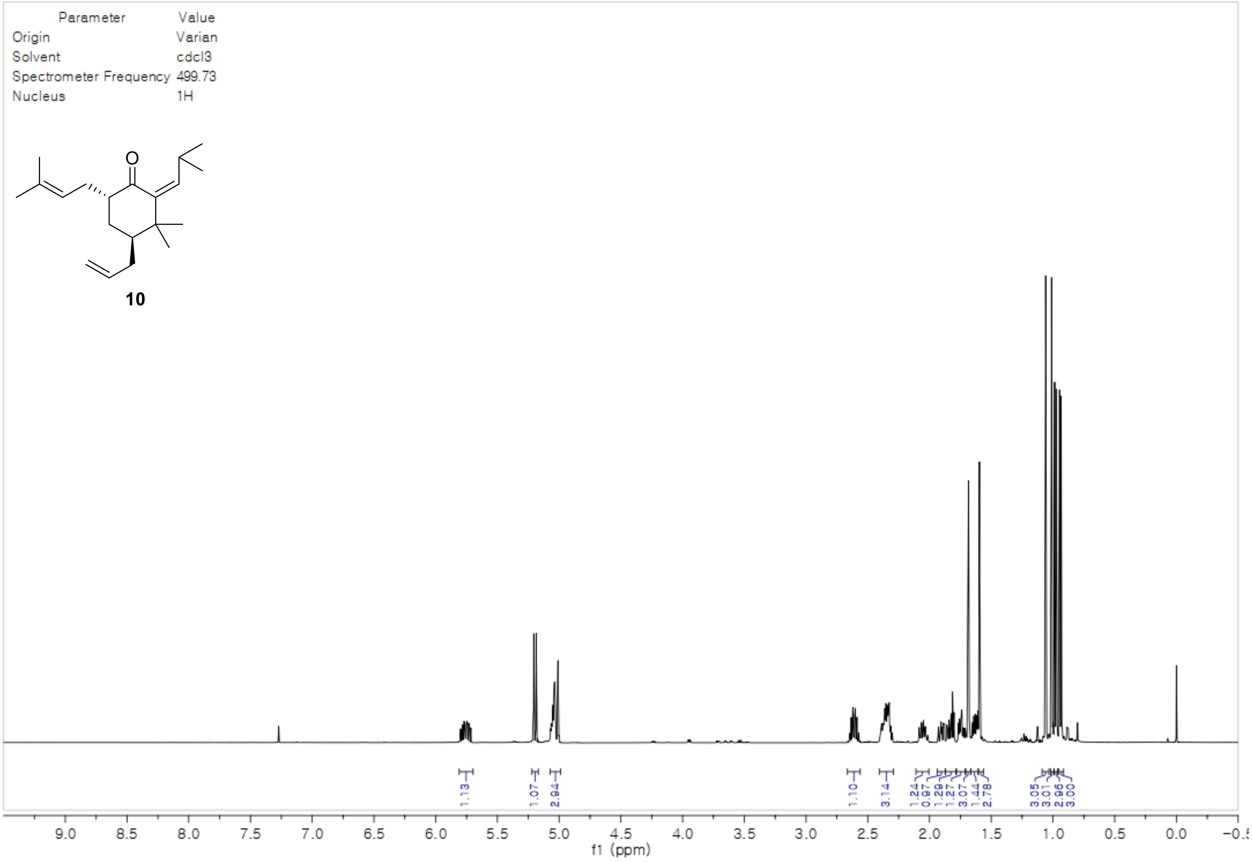

Compound 10 ^{13}C NMR

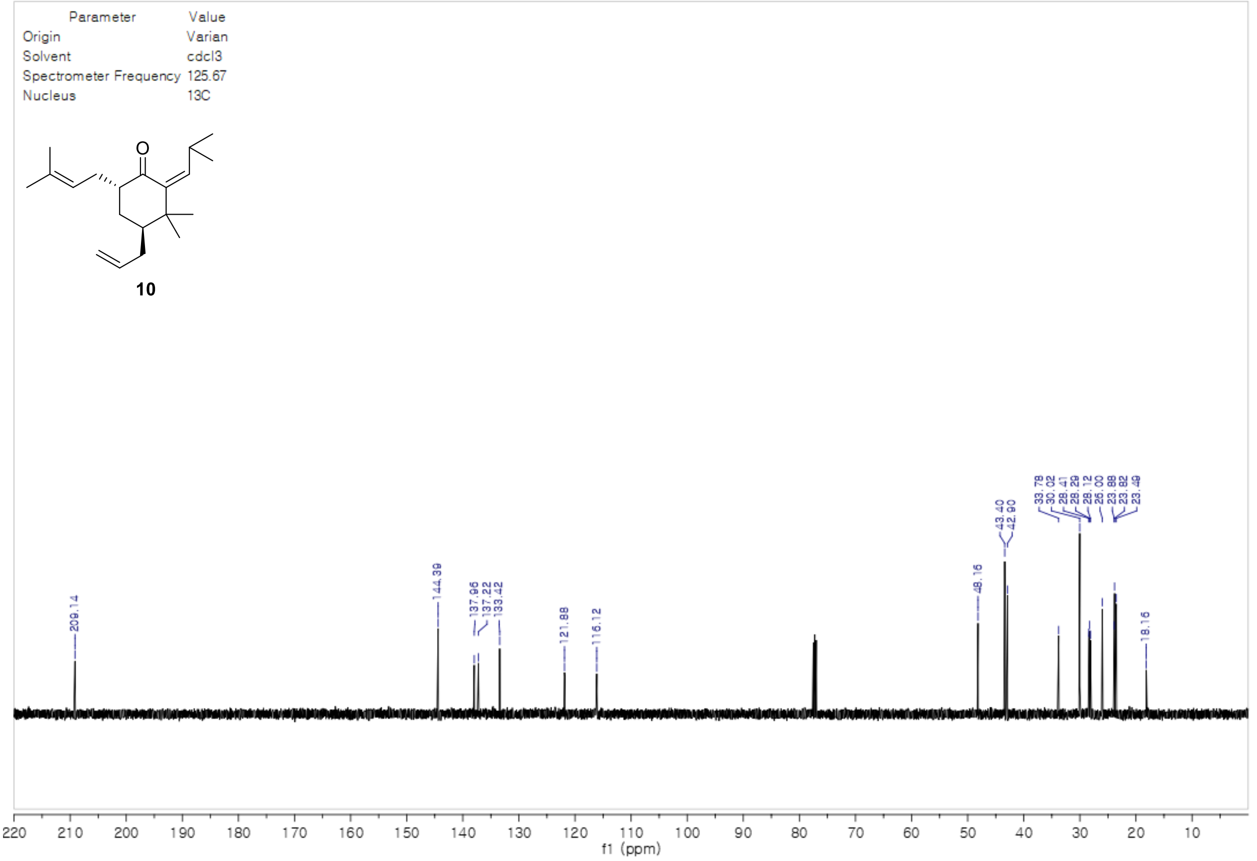

Compound 9’ ^{1}H NMR

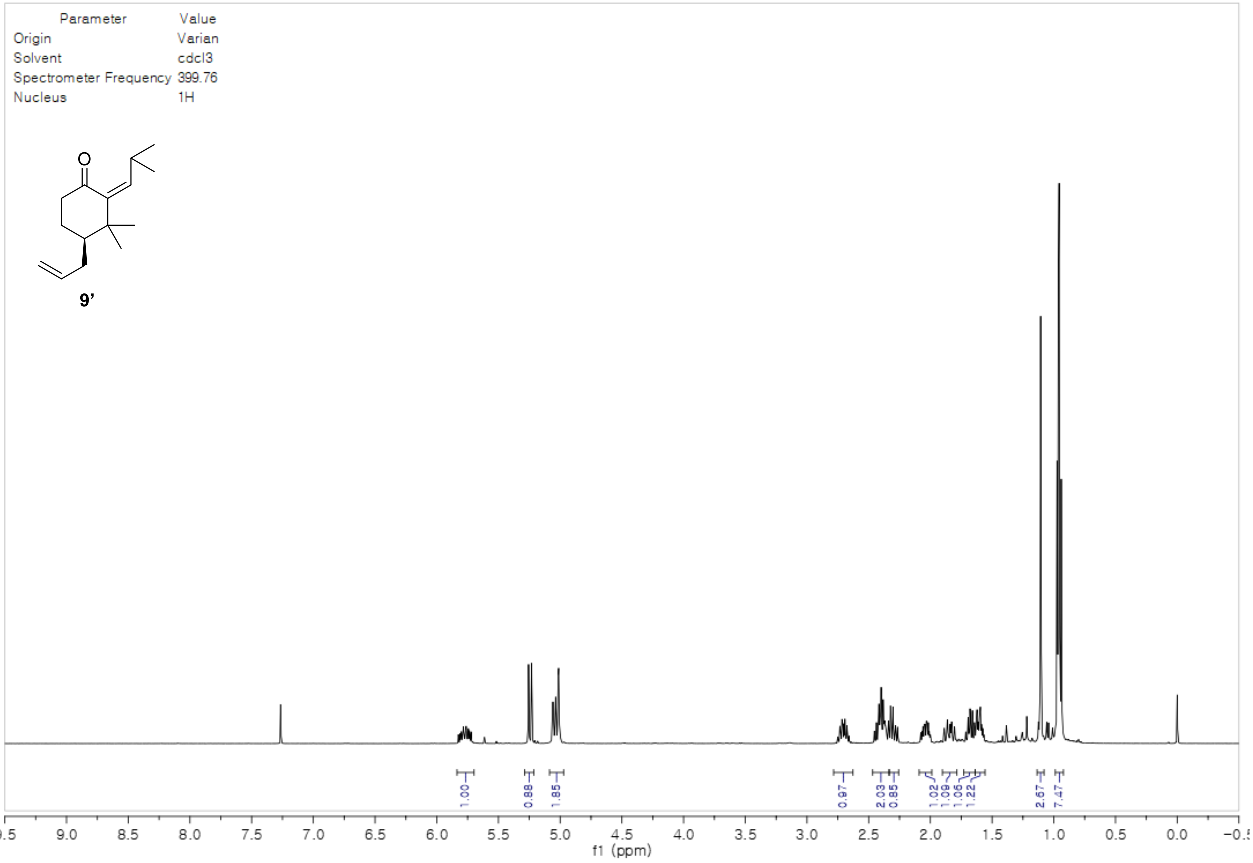

Compound 9’ ^{13}C NMR

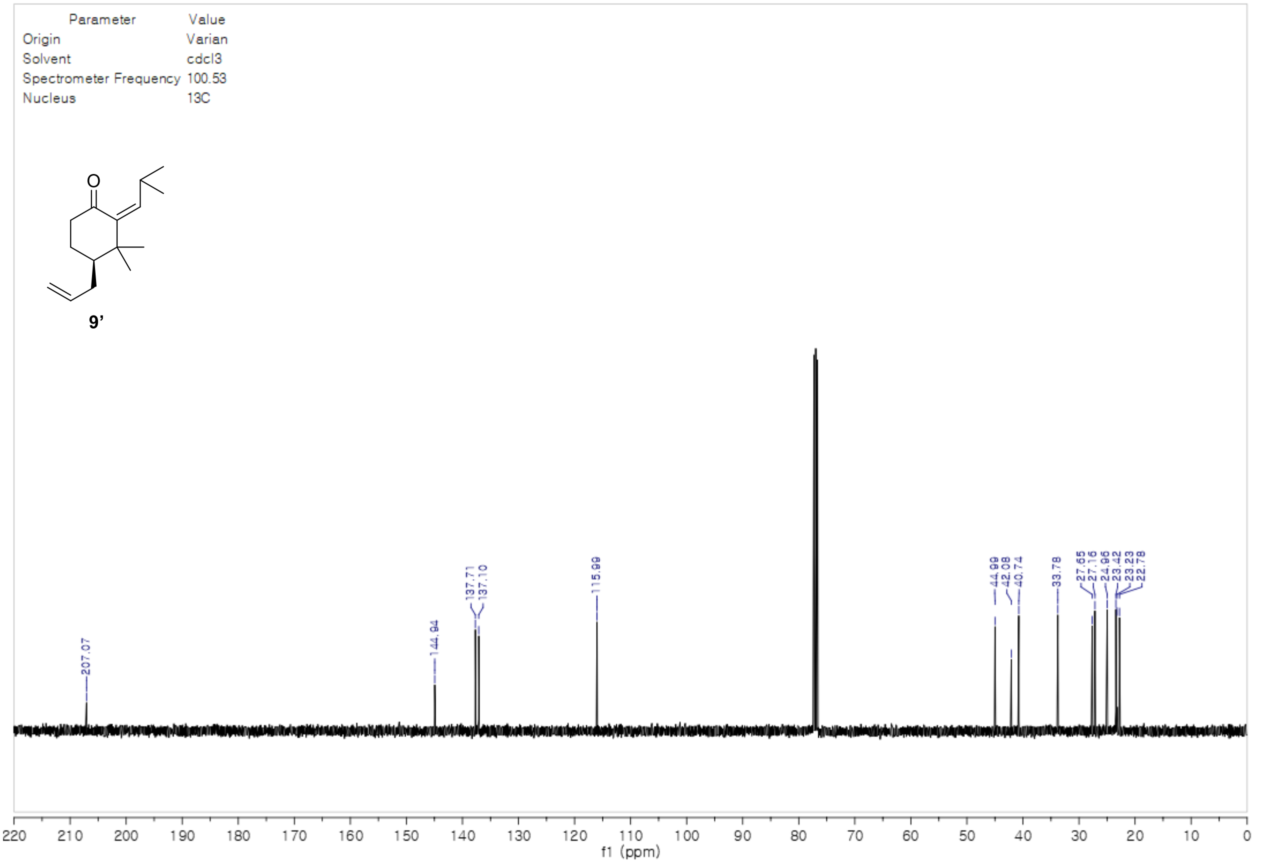

Allyl 3-chloropropiolate ^{1}H NMR

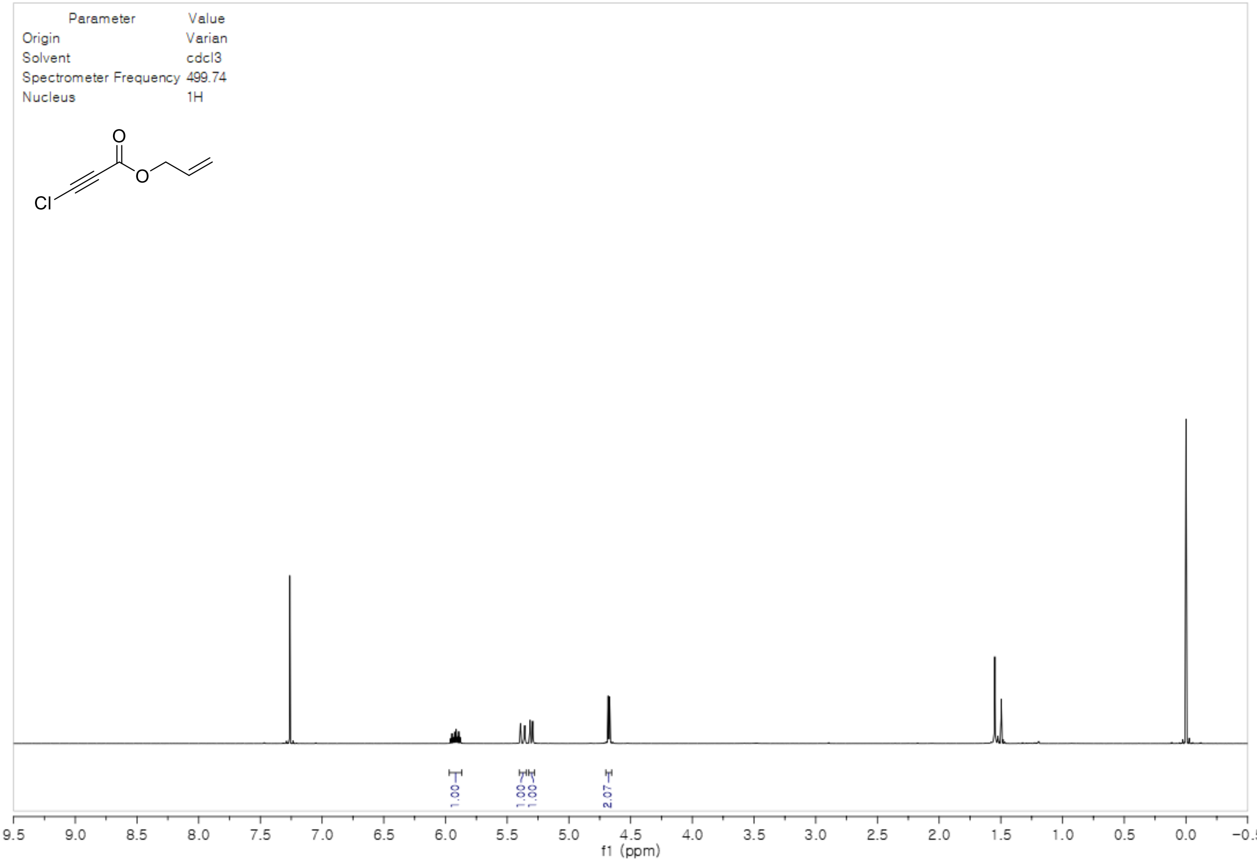

Compound 11 ^{1}H NMR

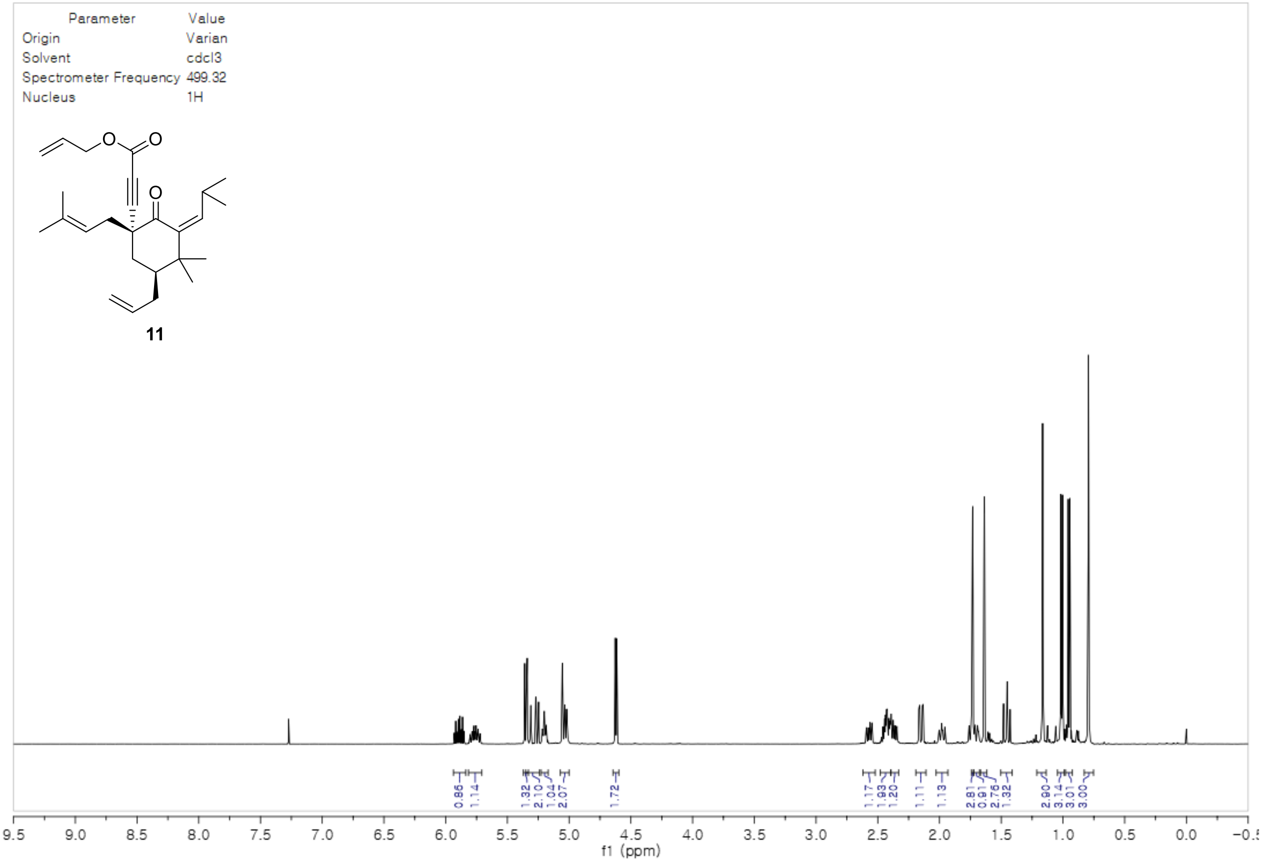

Compound 11 ^{13}C NMR

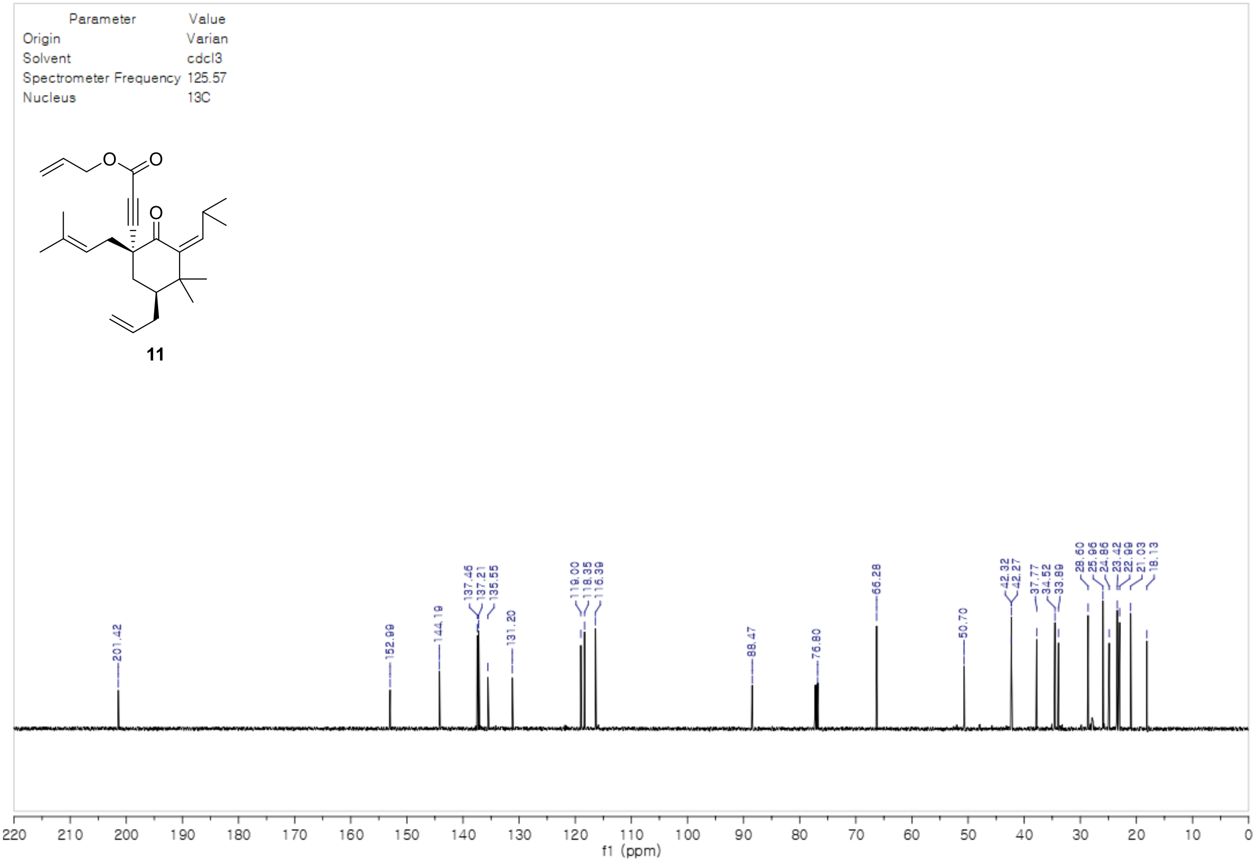

Compound 11' ^{1}H NMR

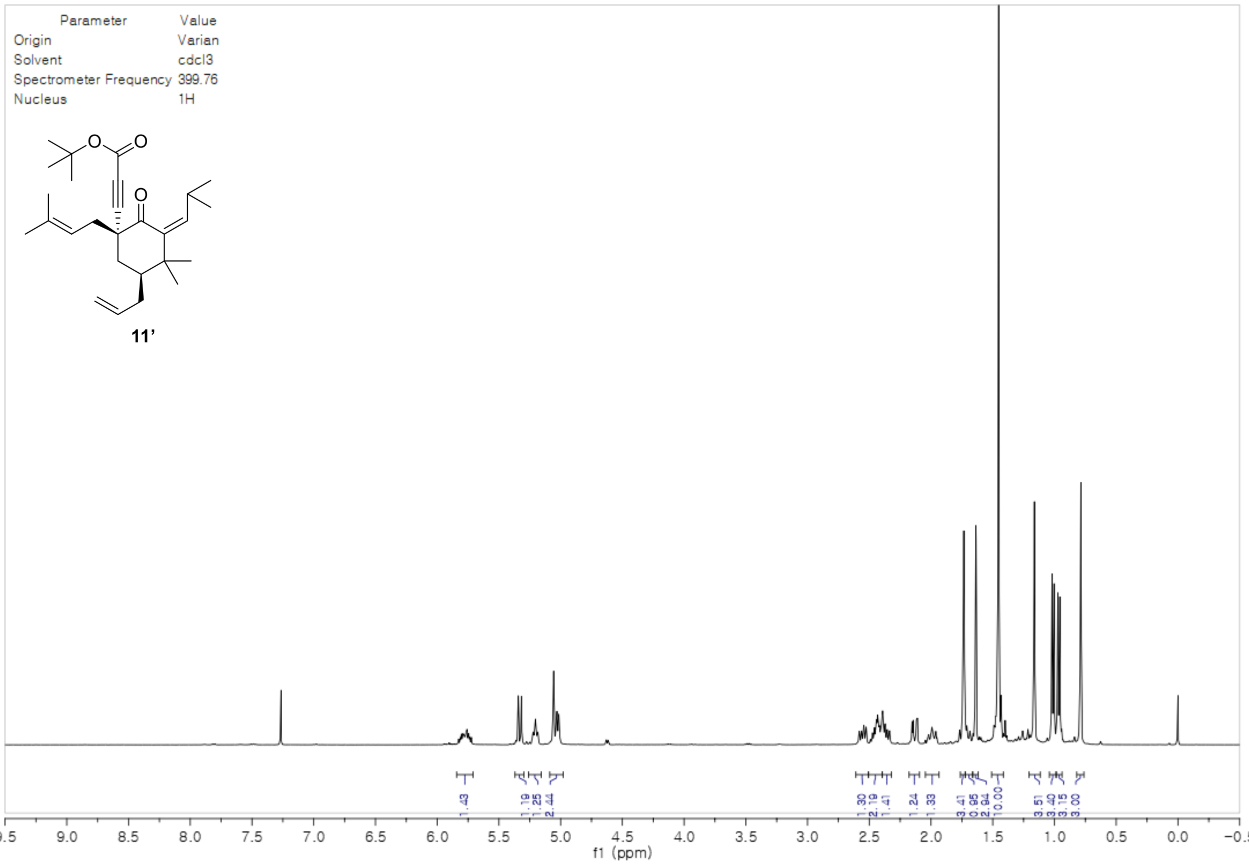

Compound 11' ^{13}C NMR

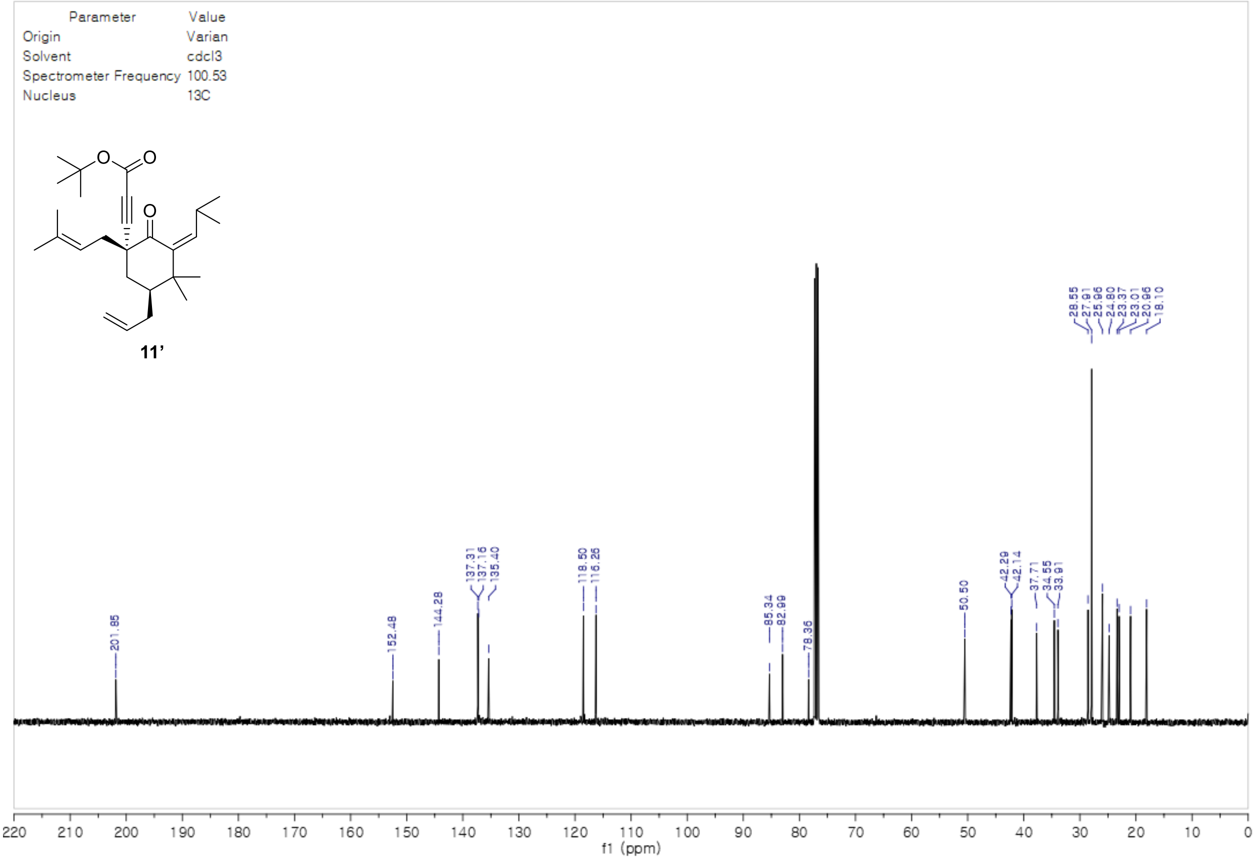

Compound 12a ^{1}H NMR

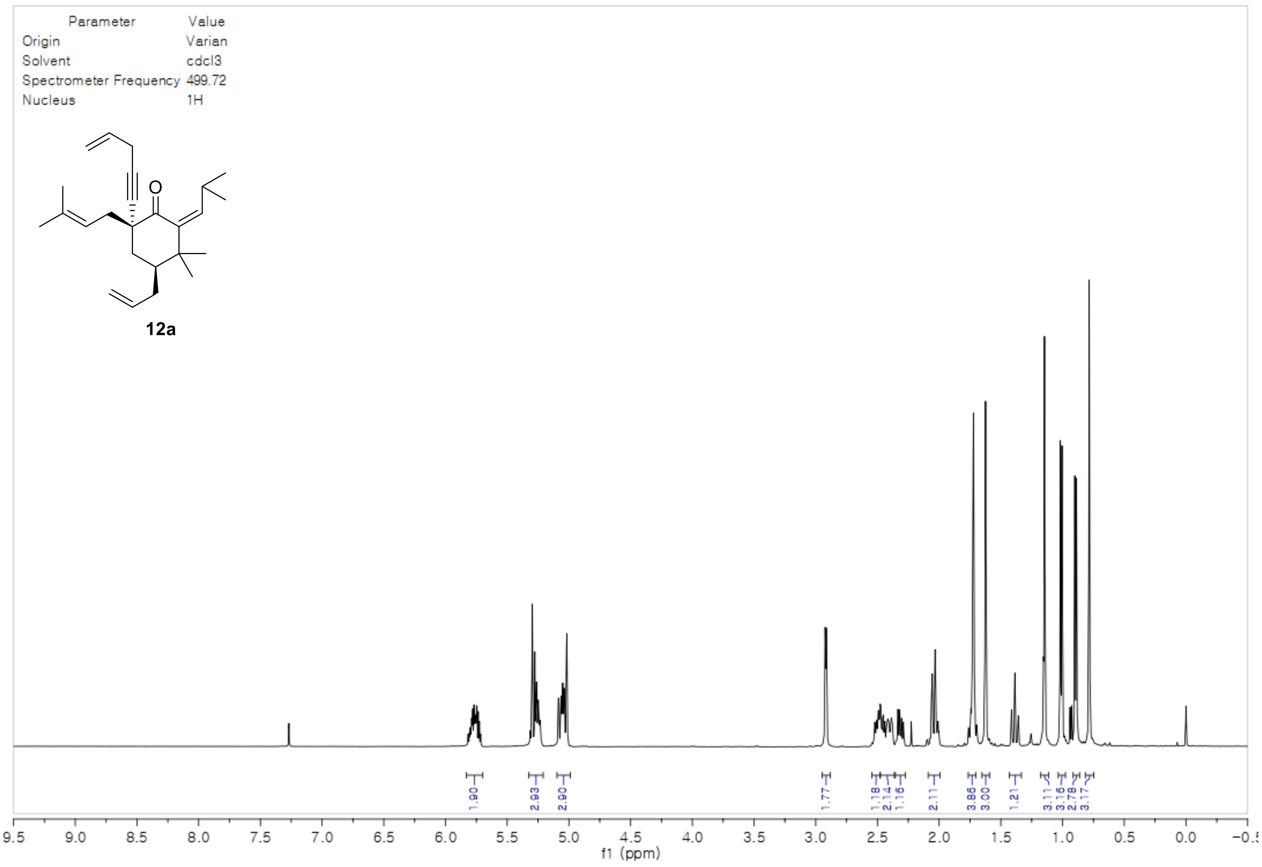

Compound 12a ^{13}C NMR

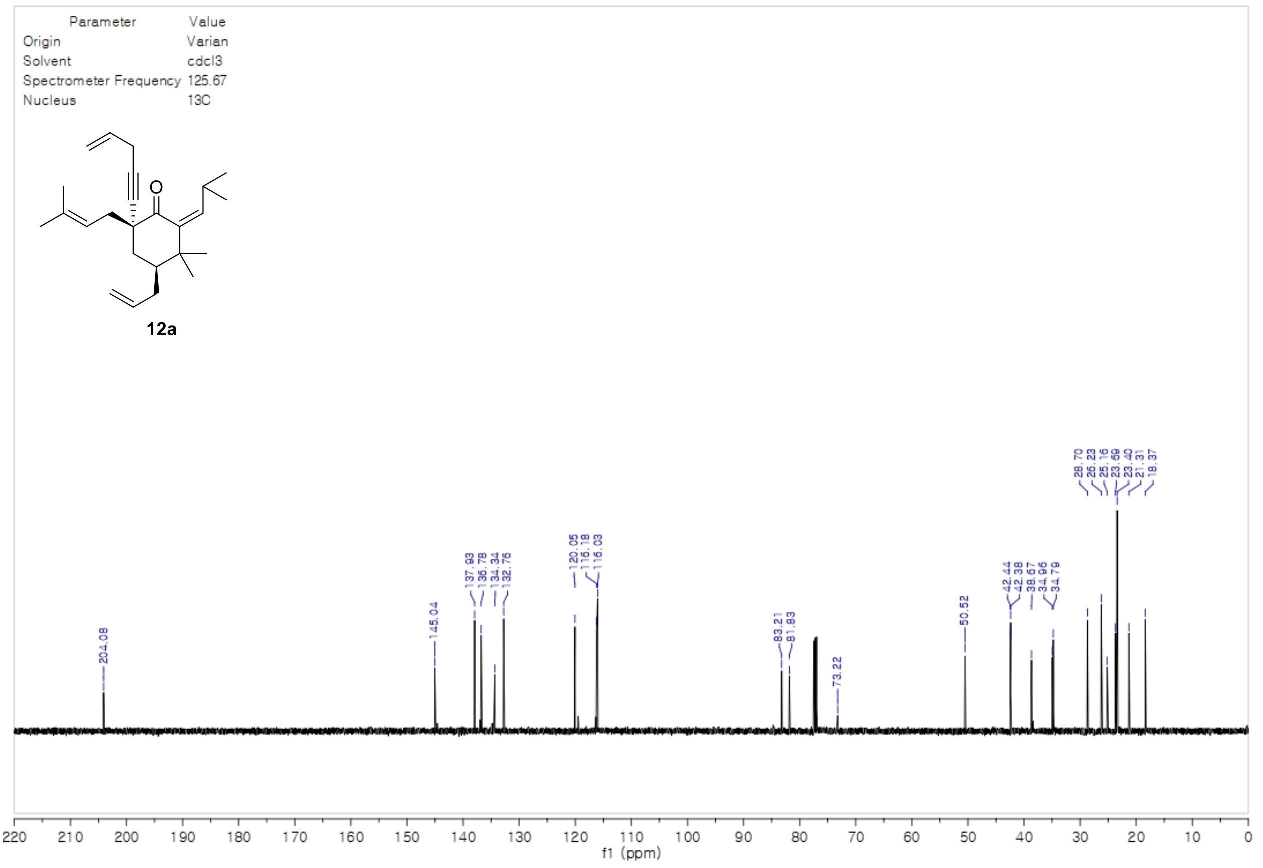

Compound 12'a ^{1}H NMR

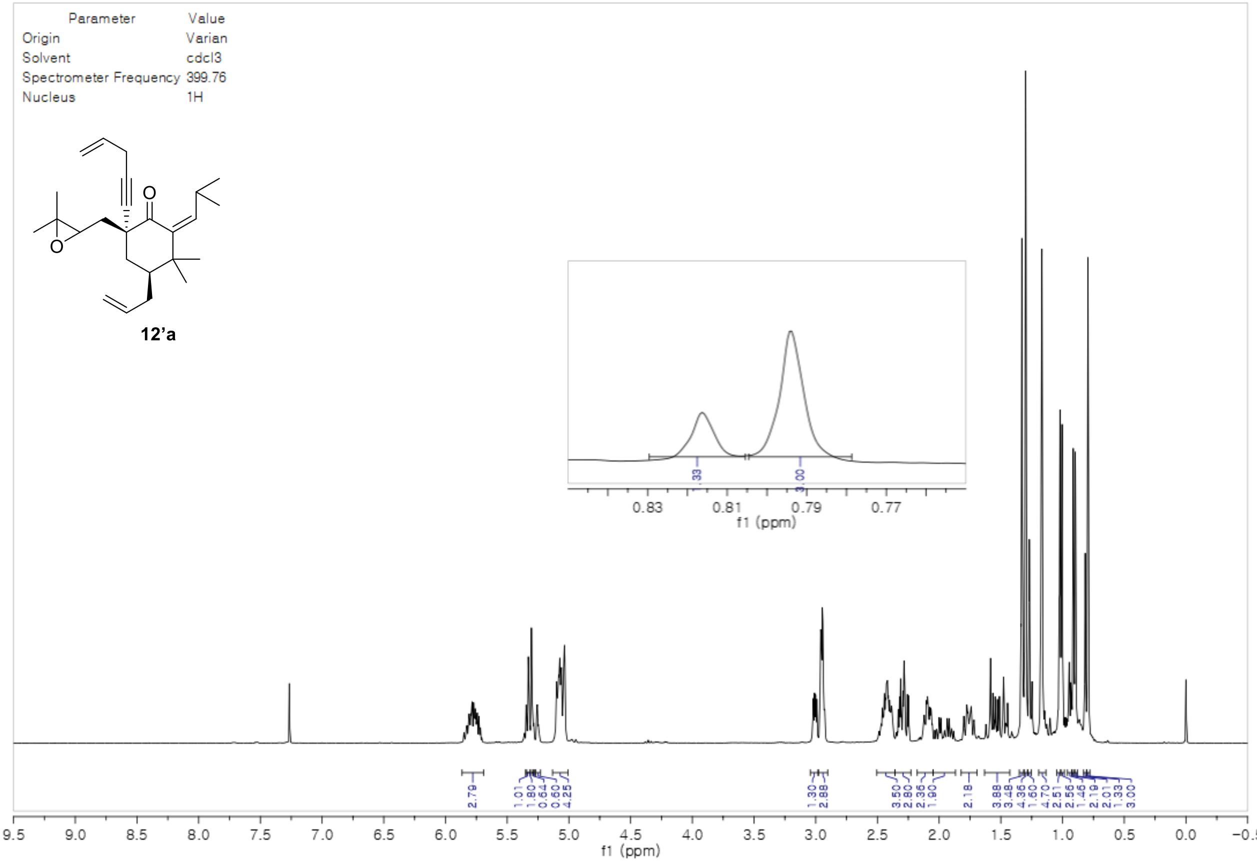

Compound 12'a ^{13}C NMR

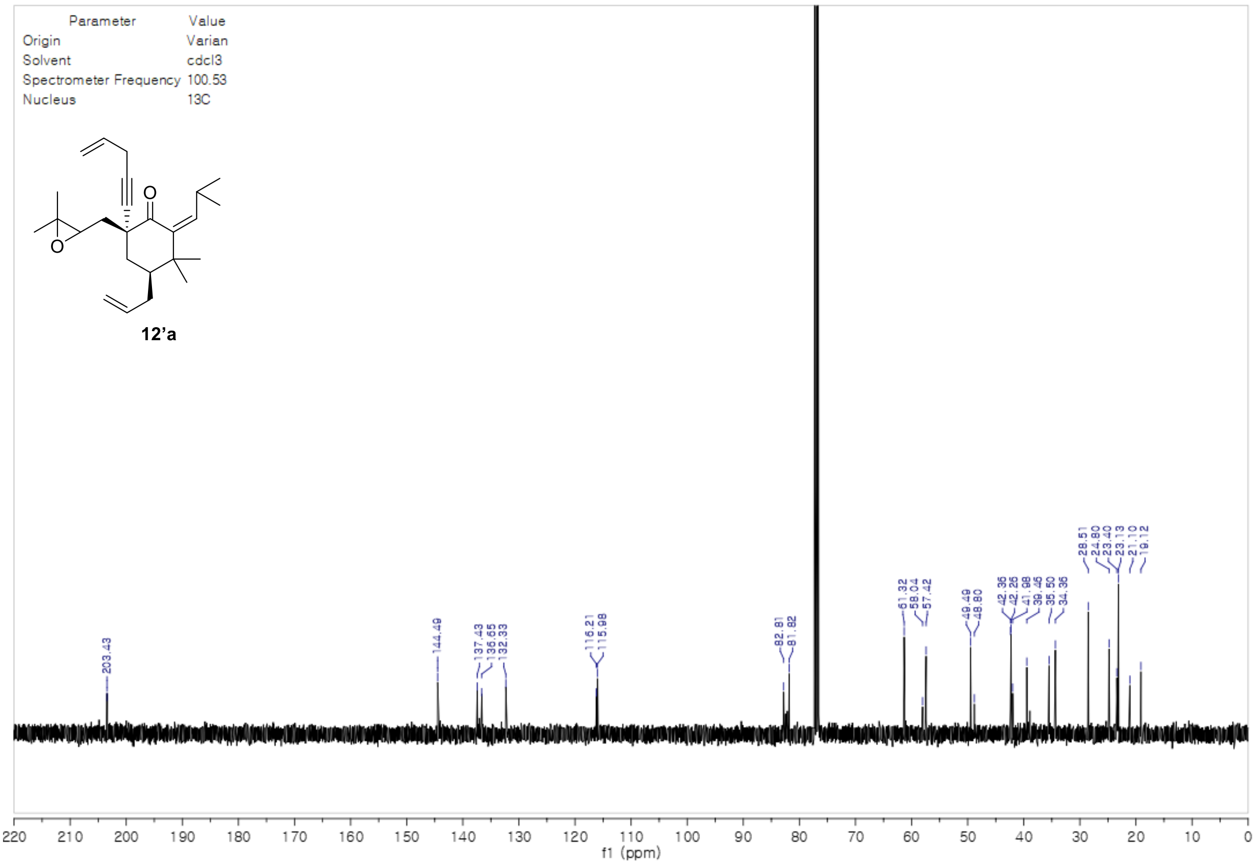

Compound 13a ^{1}H NMR

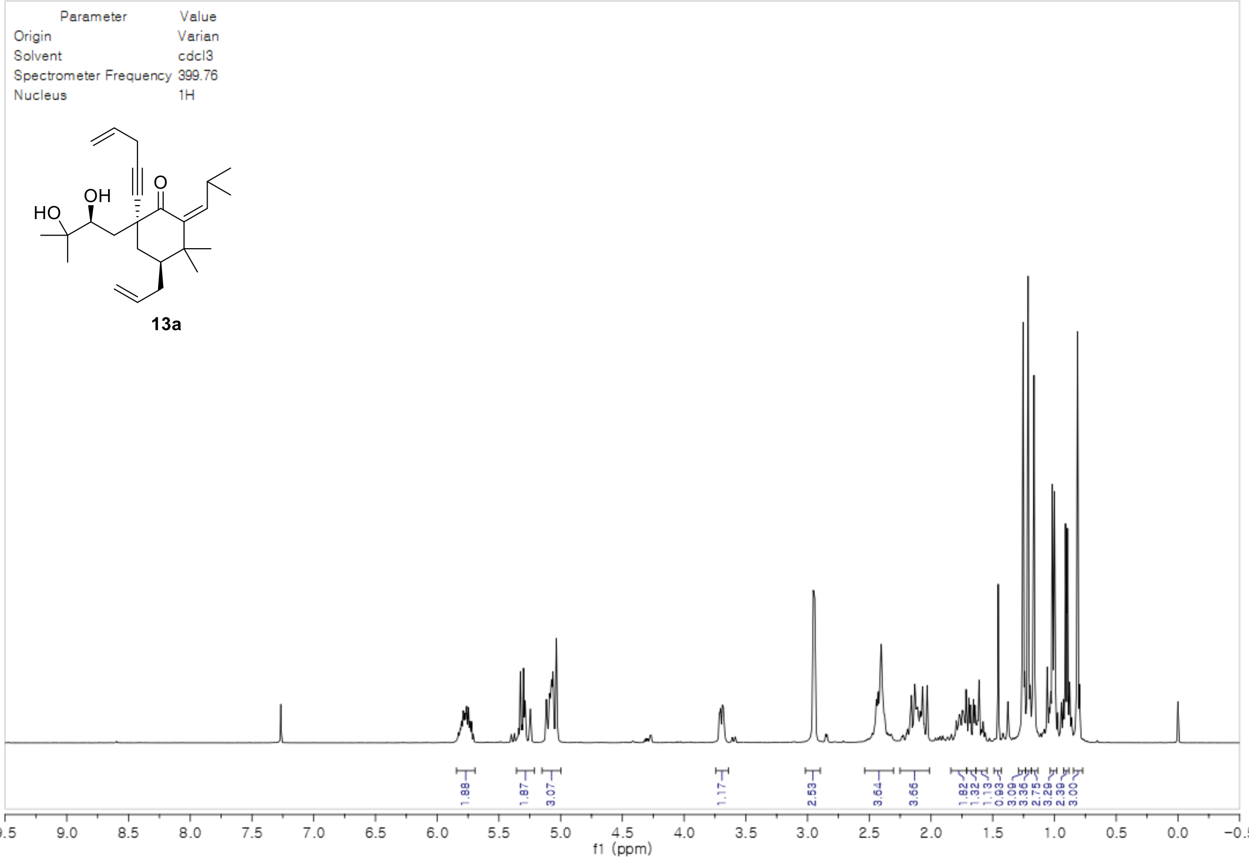

Compound 13a ^{13}C NMR

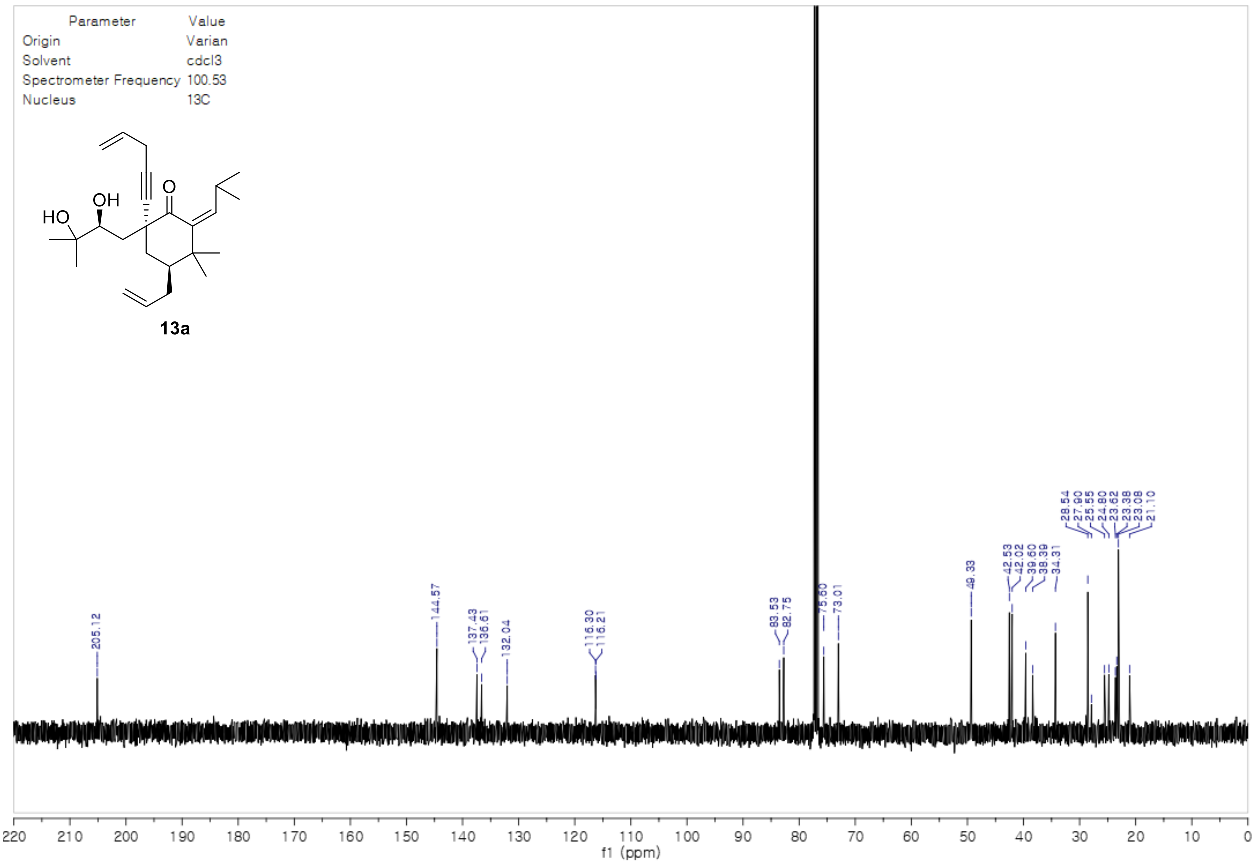

Compound 13a' ^{1}H NMR

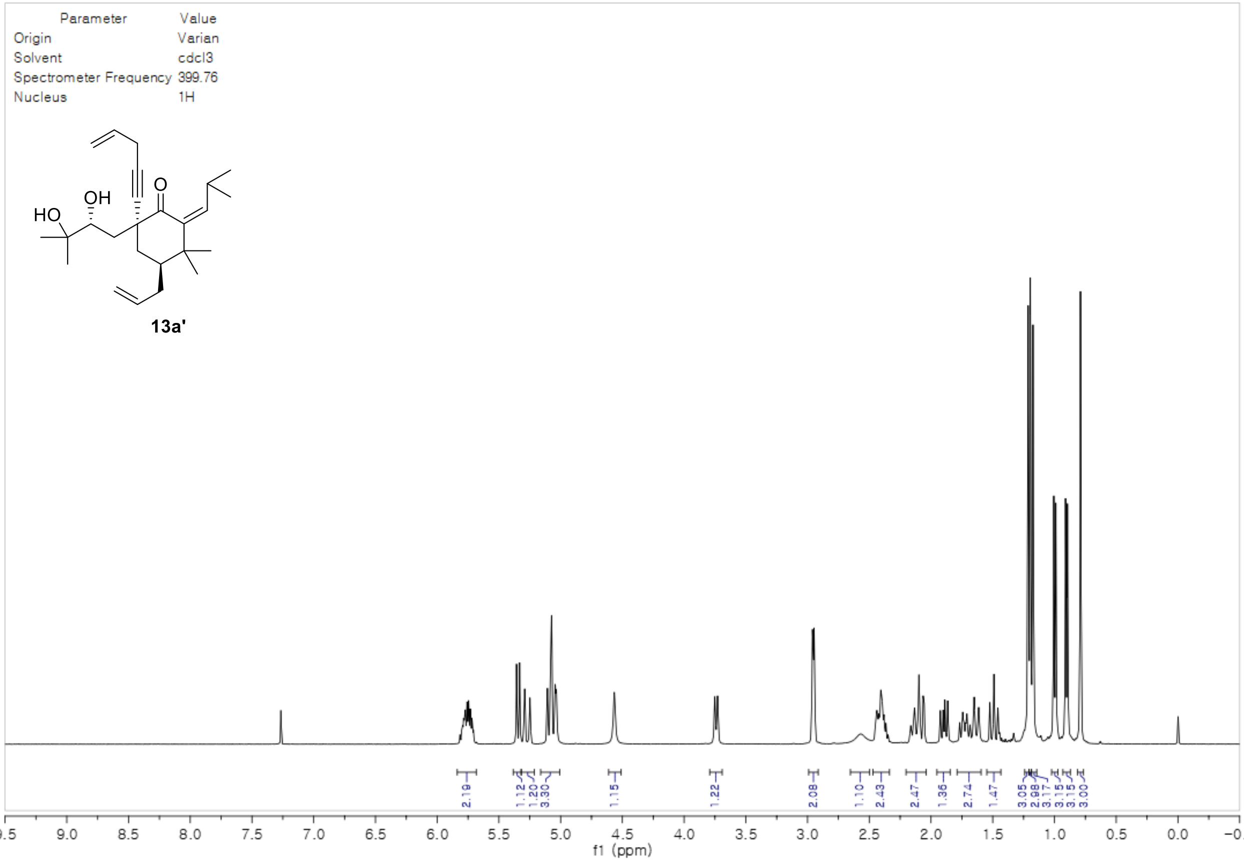

Compound 13a' ^{13}C NMR

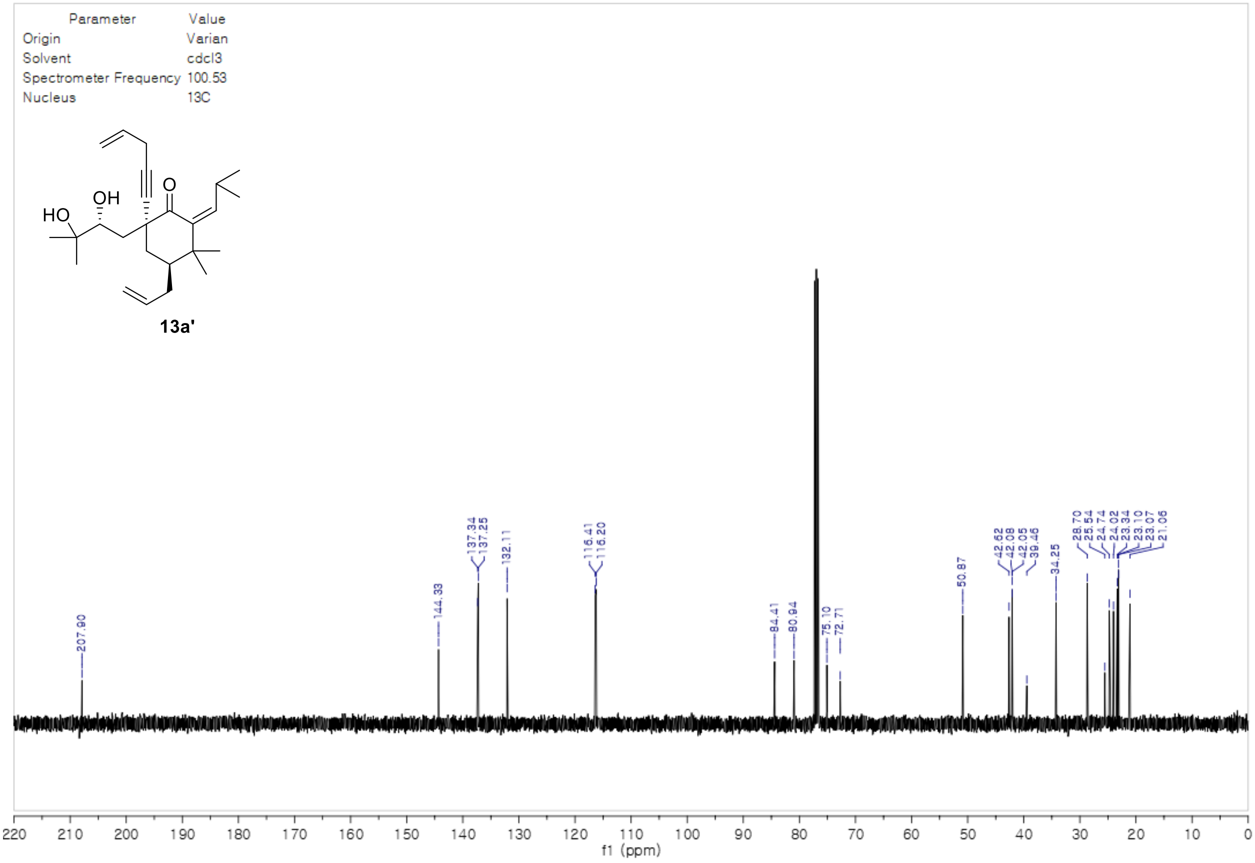

Compound 16 ^{1}H NMR

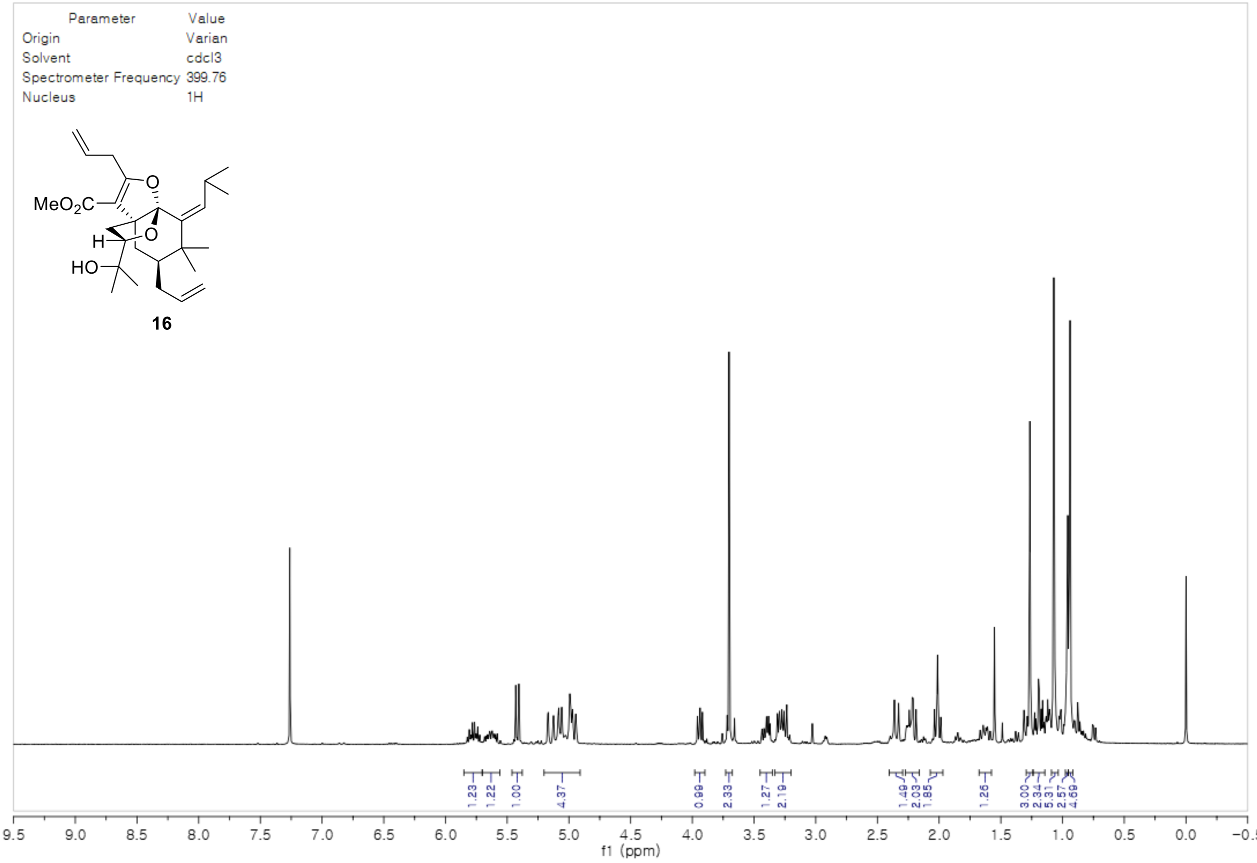

Compound 16 ^{13}C NMR

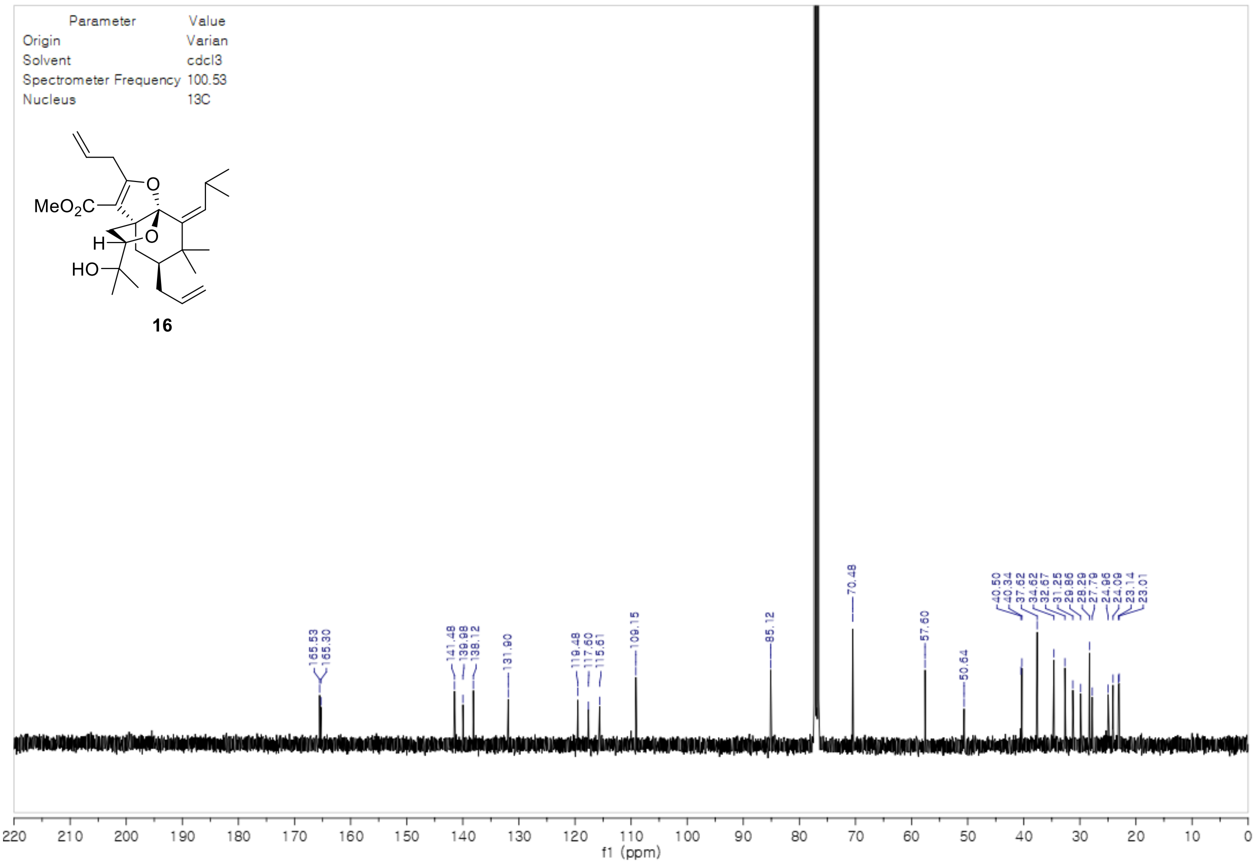

Compound 15' ^{1}H NMR

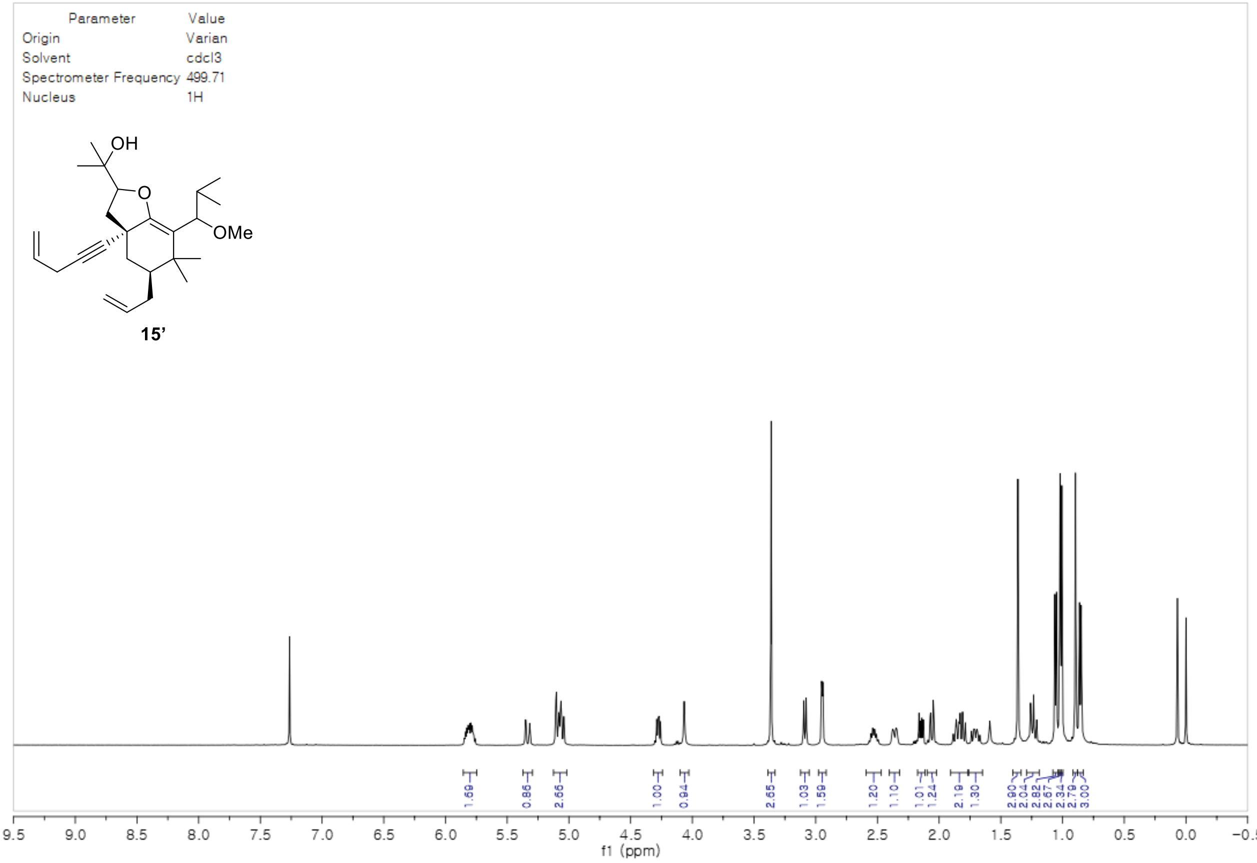

Compound 15' ^{13}C NMR

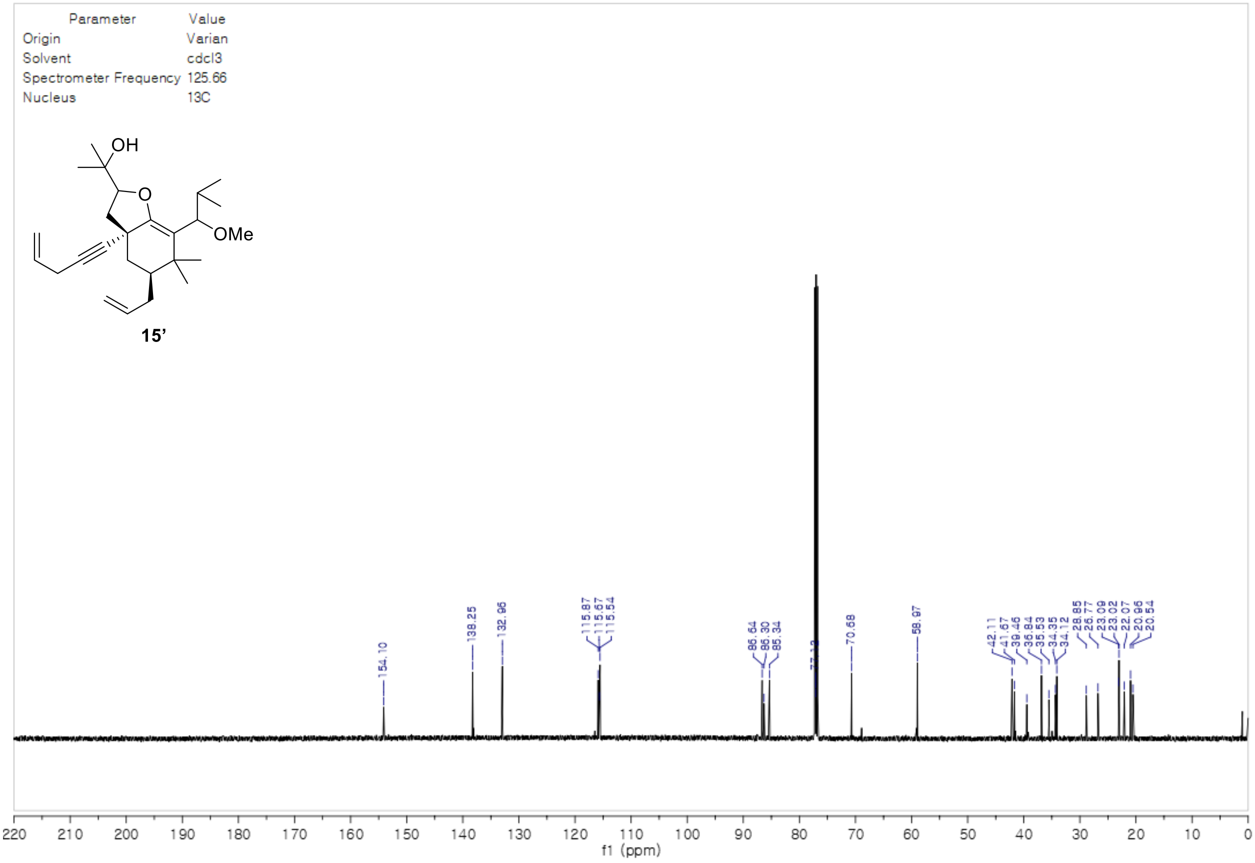

Compound 12b ^{1}H NMR

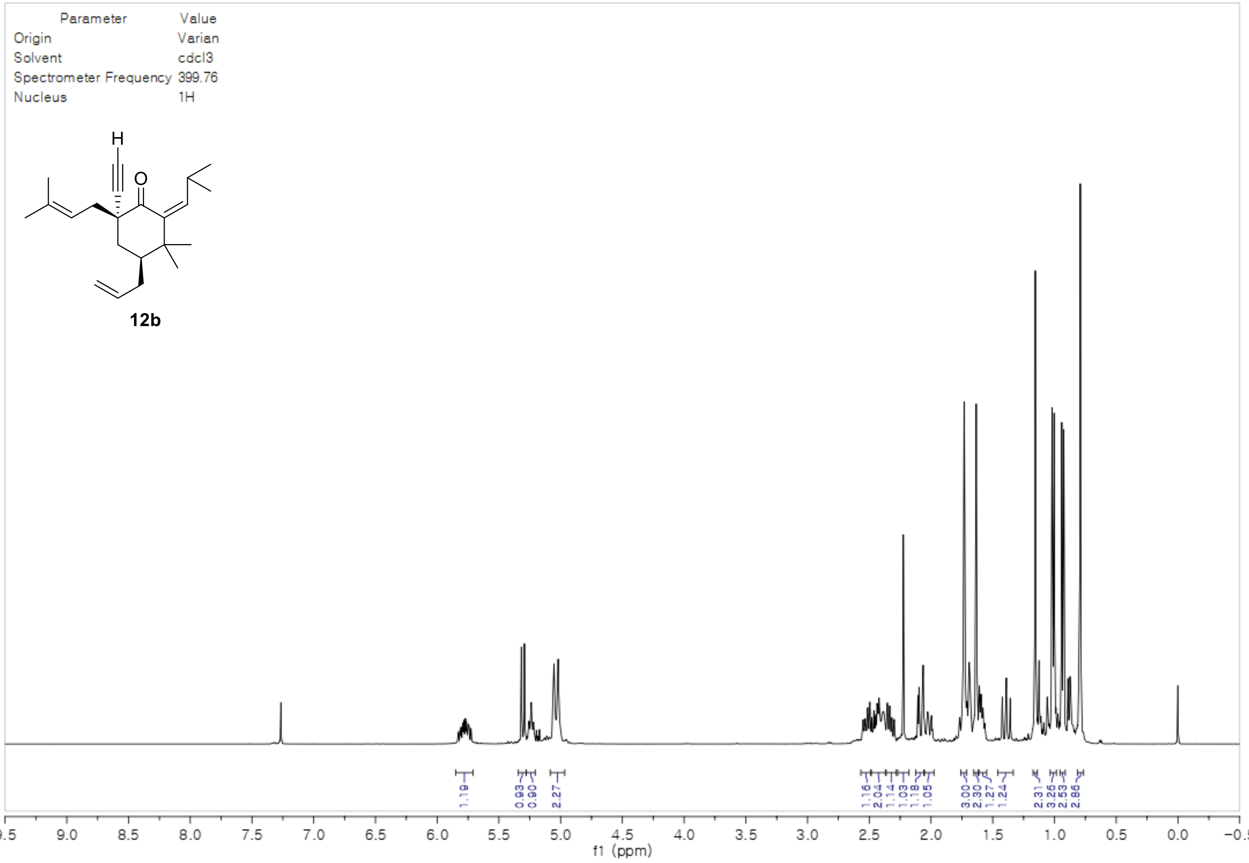

Compound 12b ^{13}C NMR

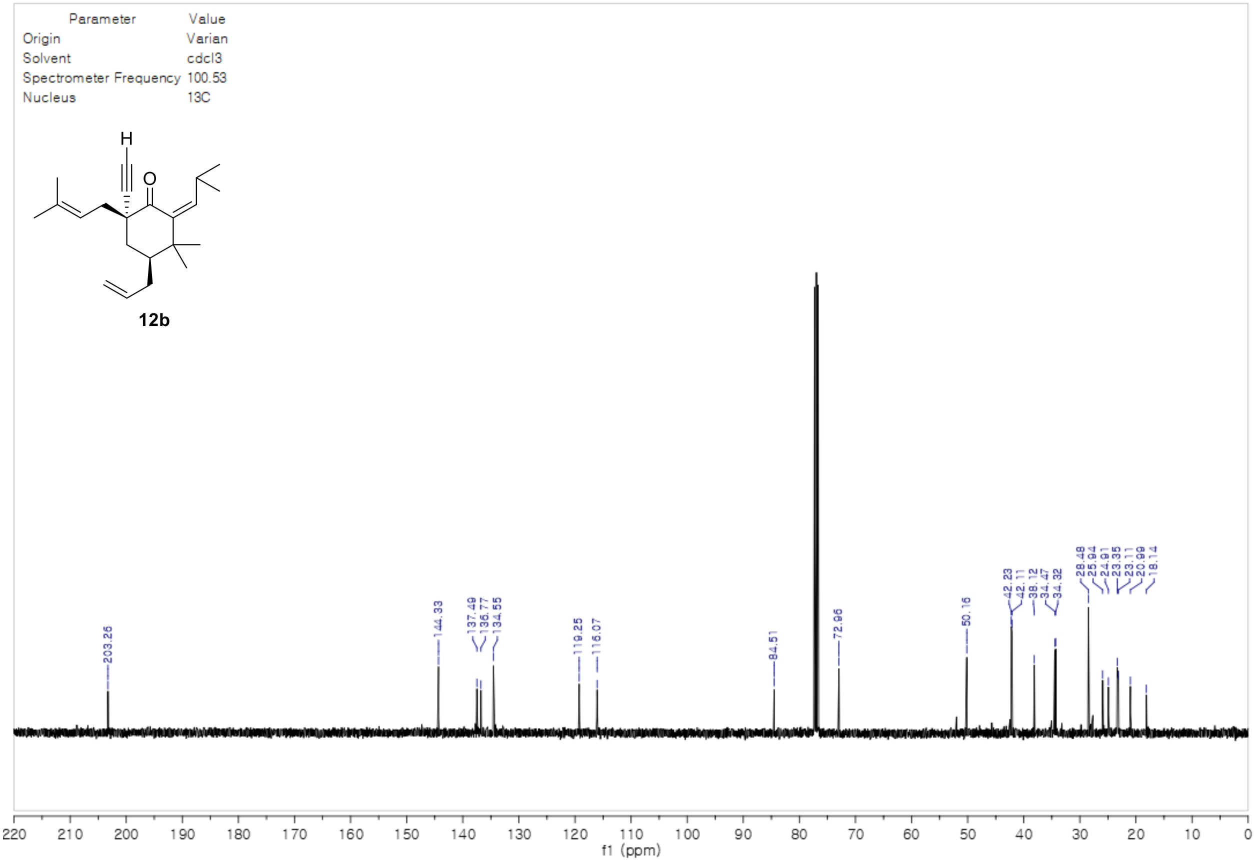

Compound 12’b ^{1}H NMR

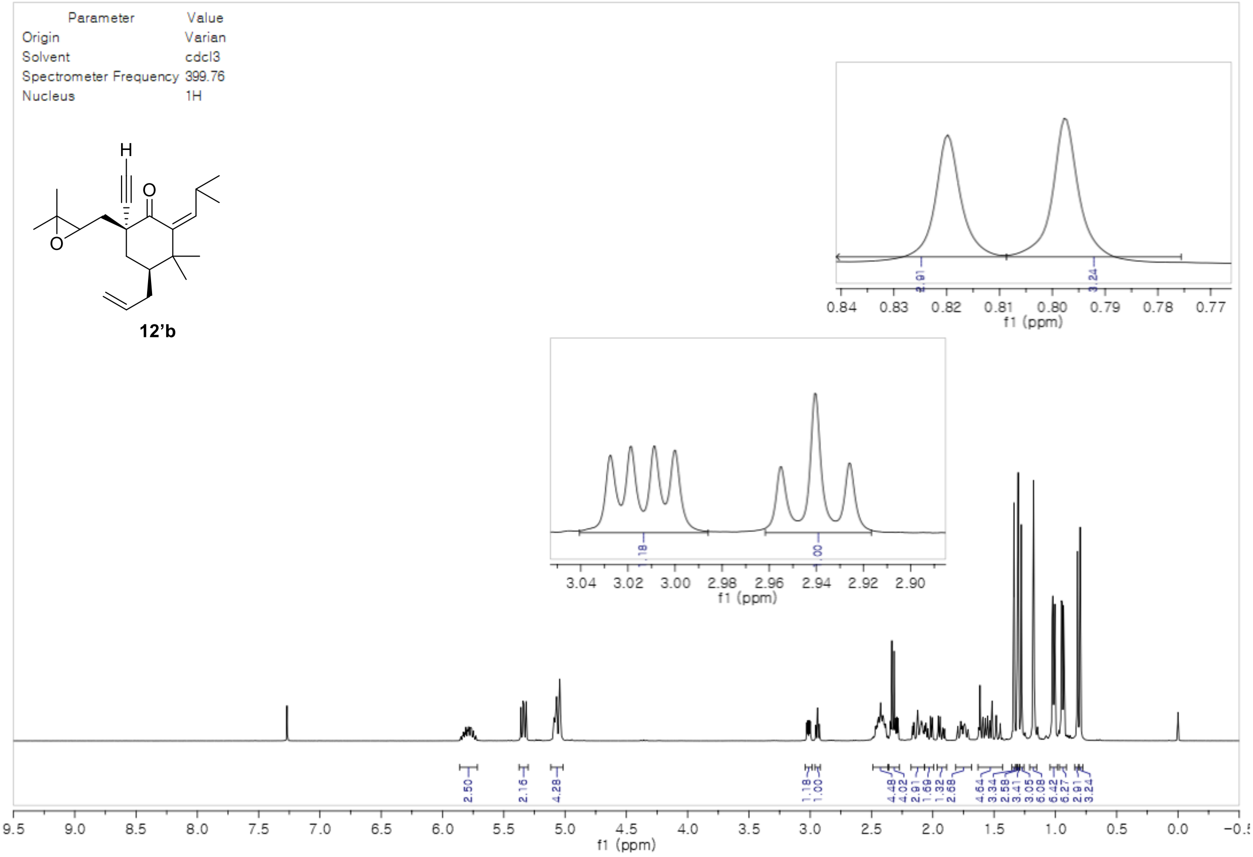

Compound 12’b ^{13}C NMR

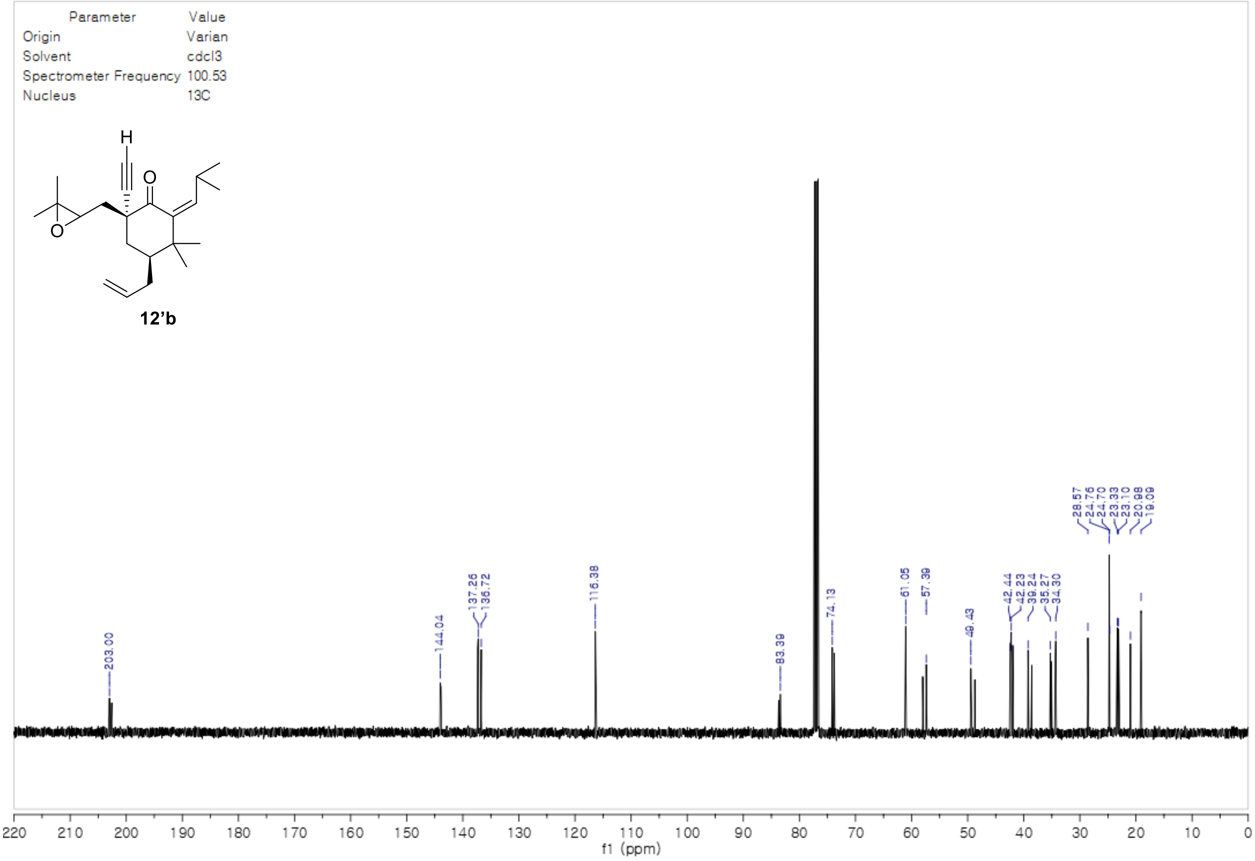

Compound 13b ^{1}H NMR

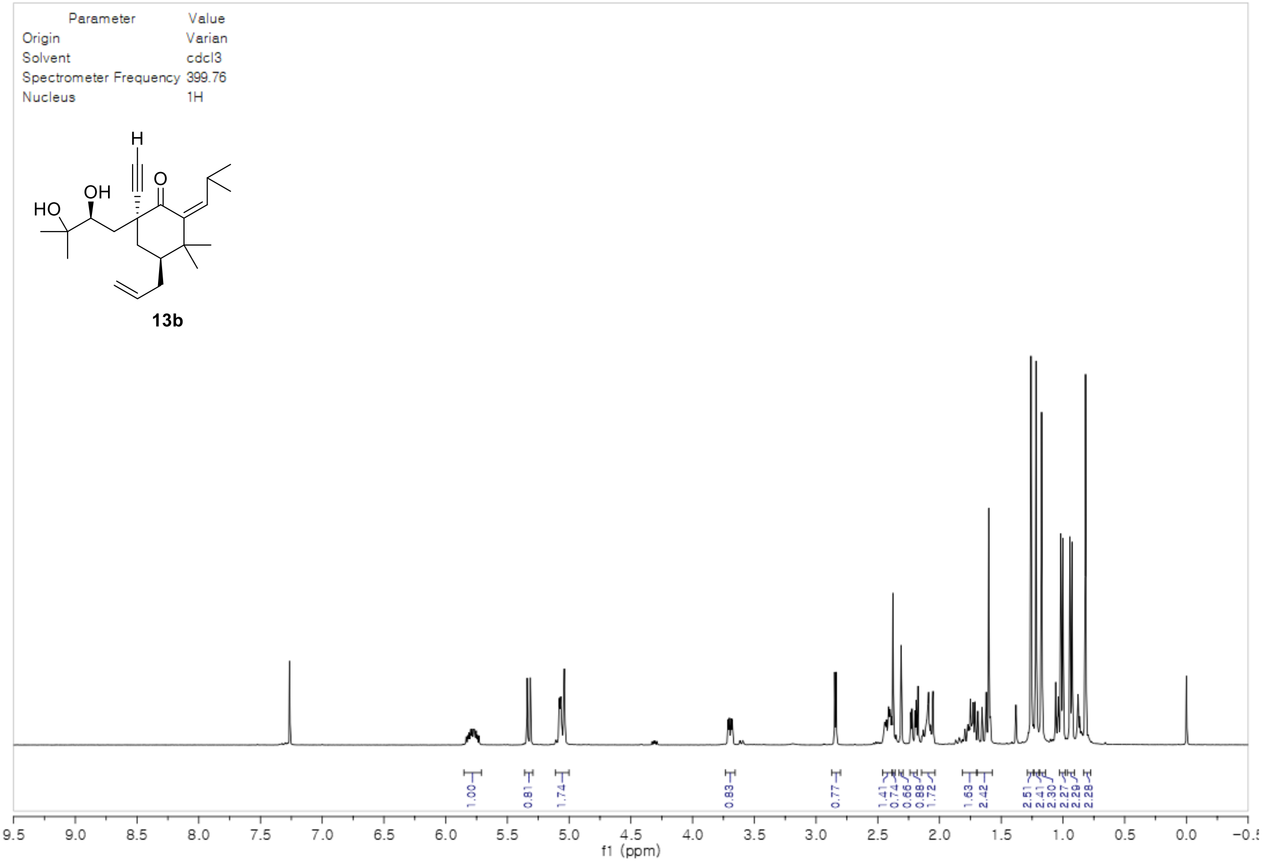

Compound 13b ^{13}C NMR

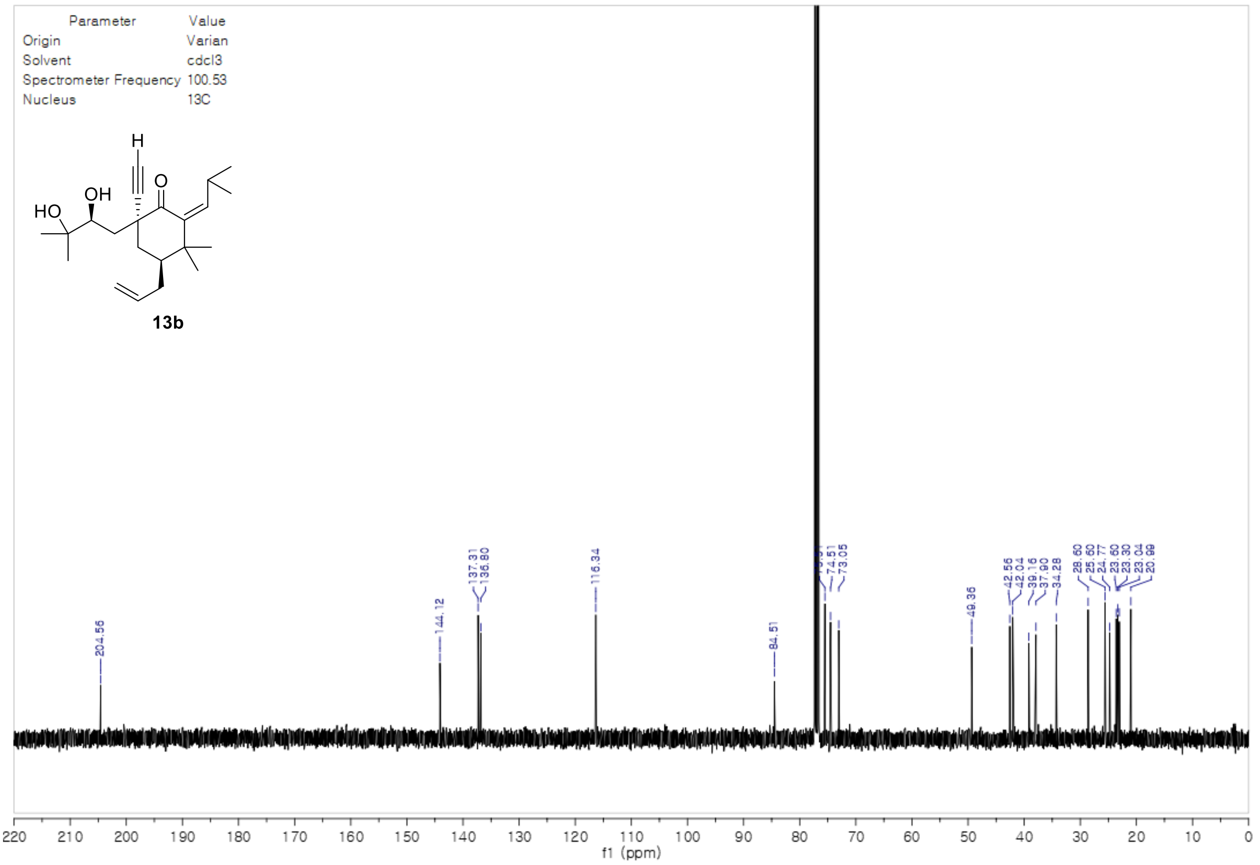

Compound 13b' ^{1}H NMR

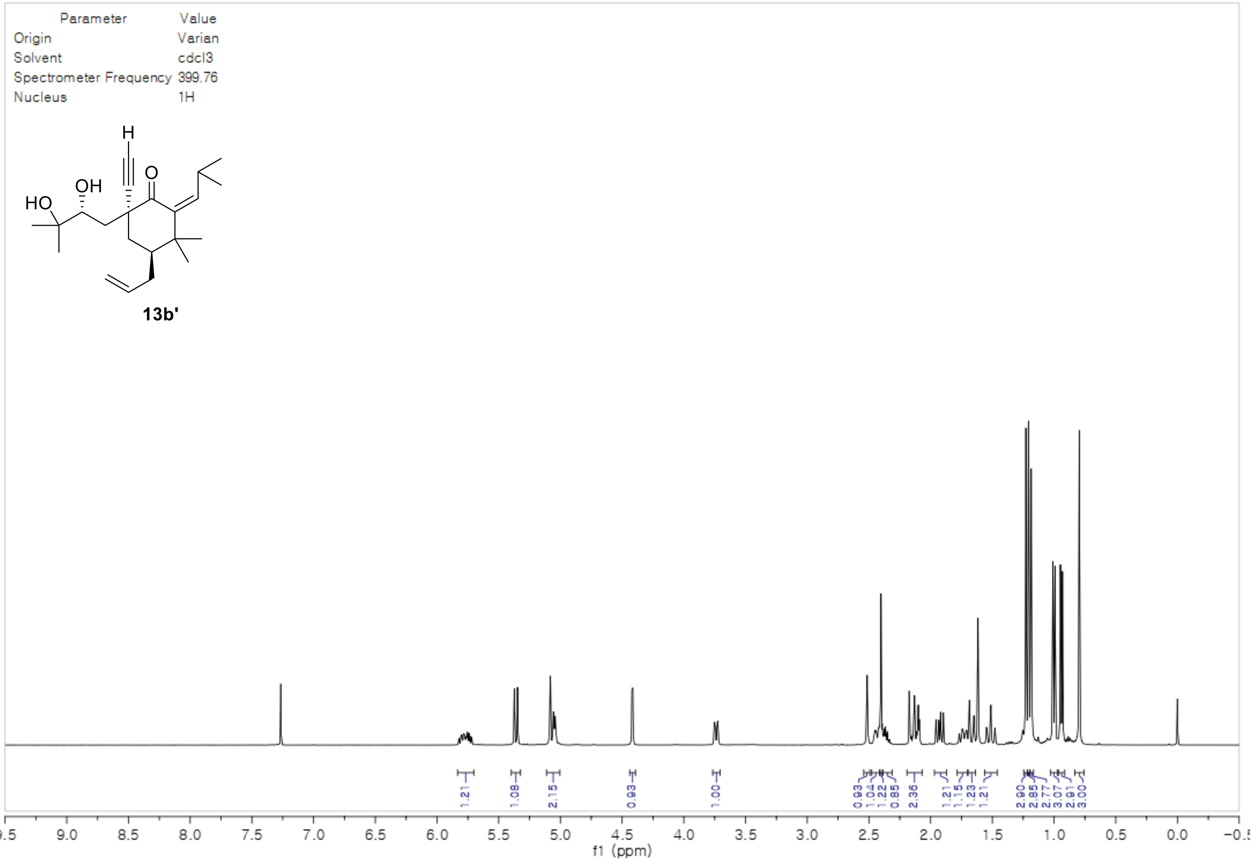

Compound 13b' ^{13}C NMR

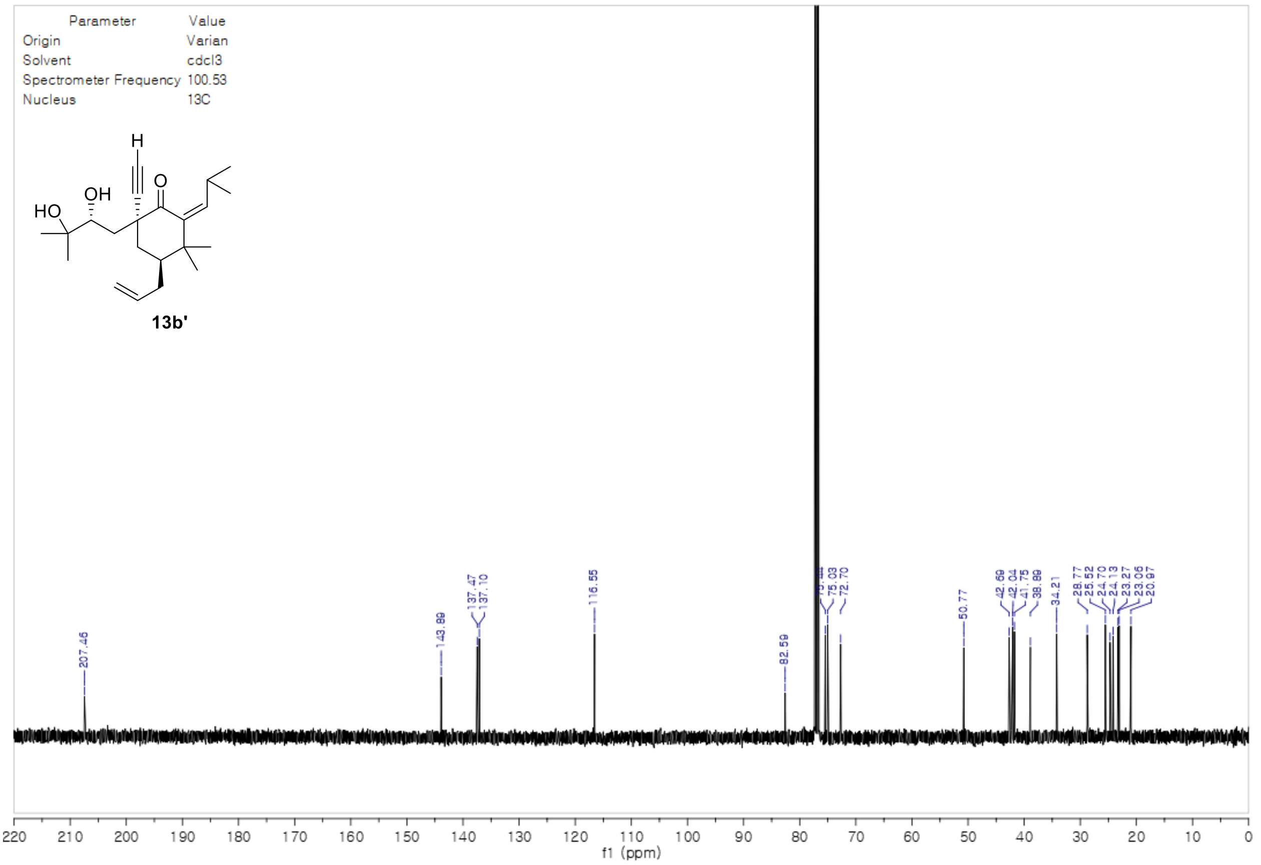

Compound 17 ^{1}H NMR

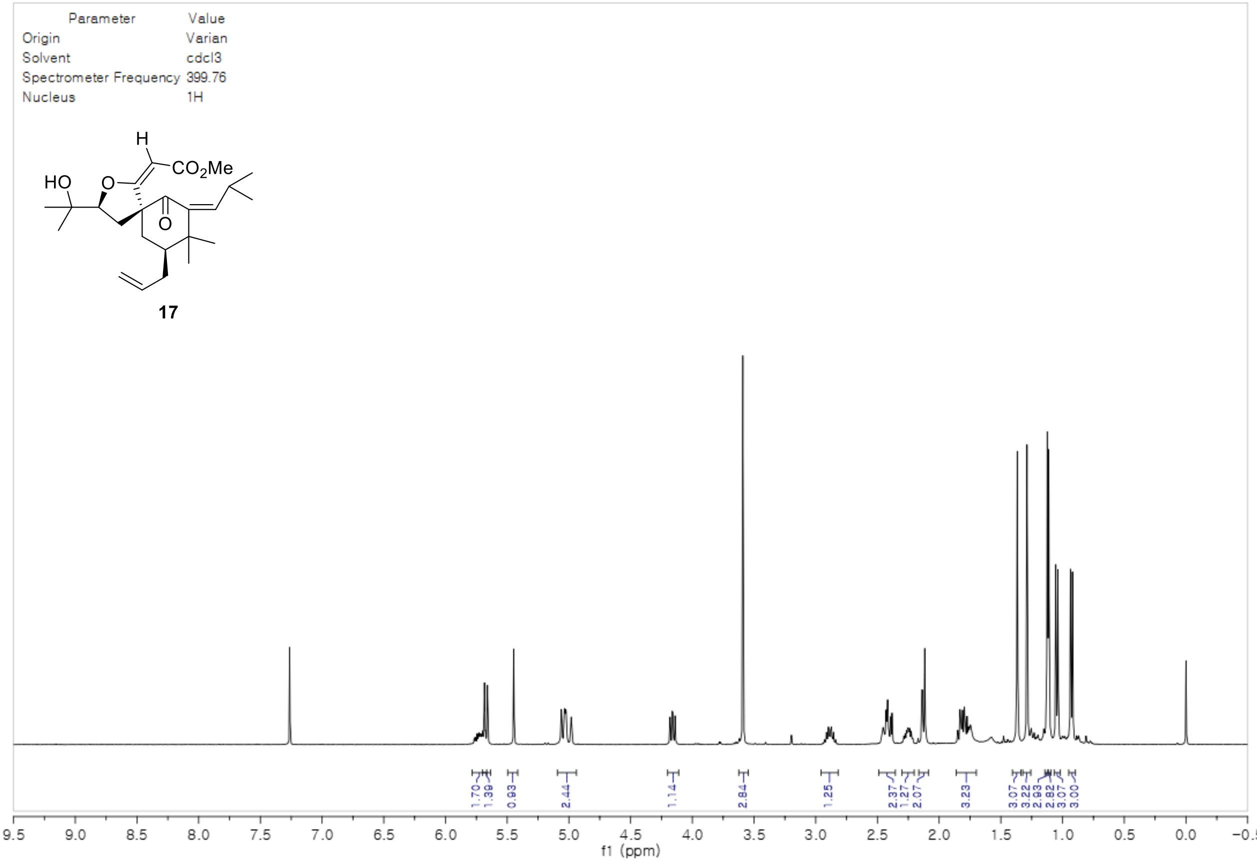

Compound 17 ^{13}C NMR

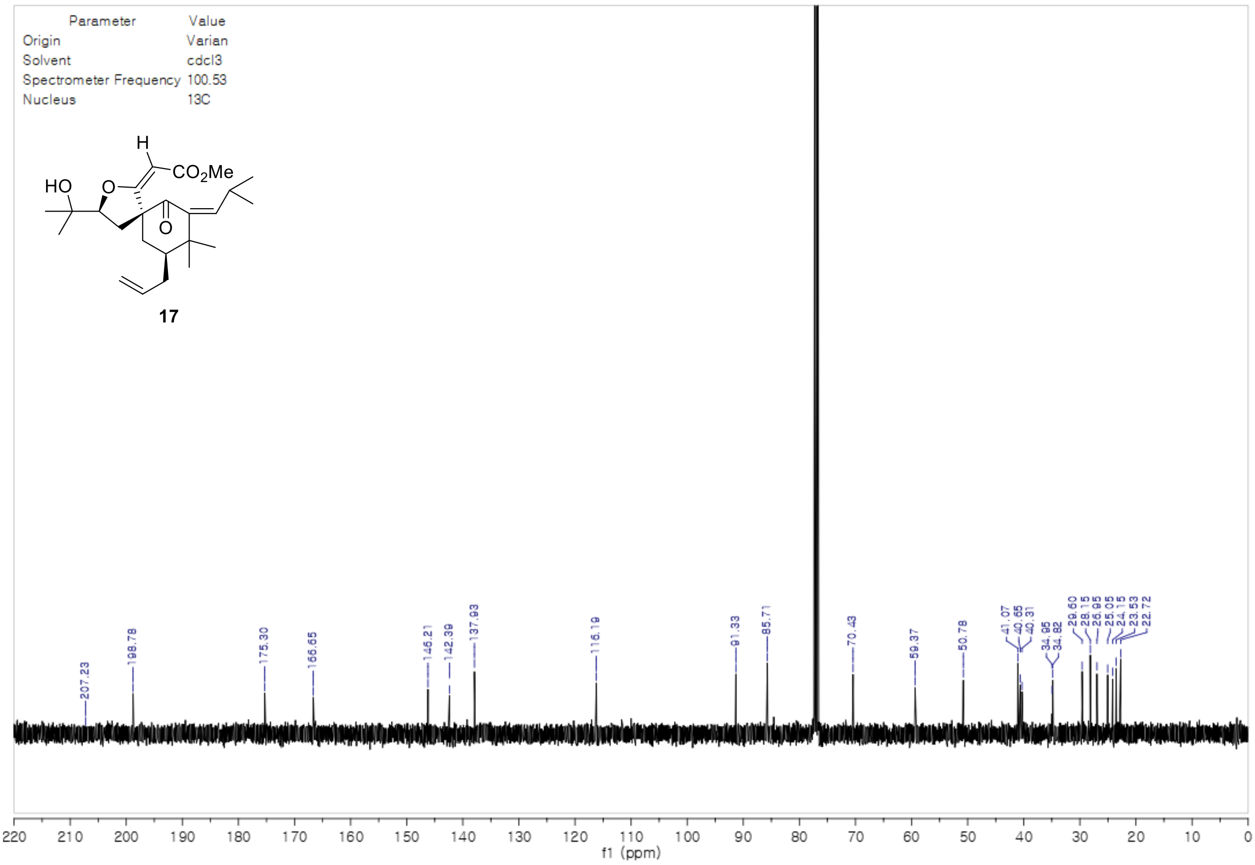

Compound 20 ^{1}H NMR

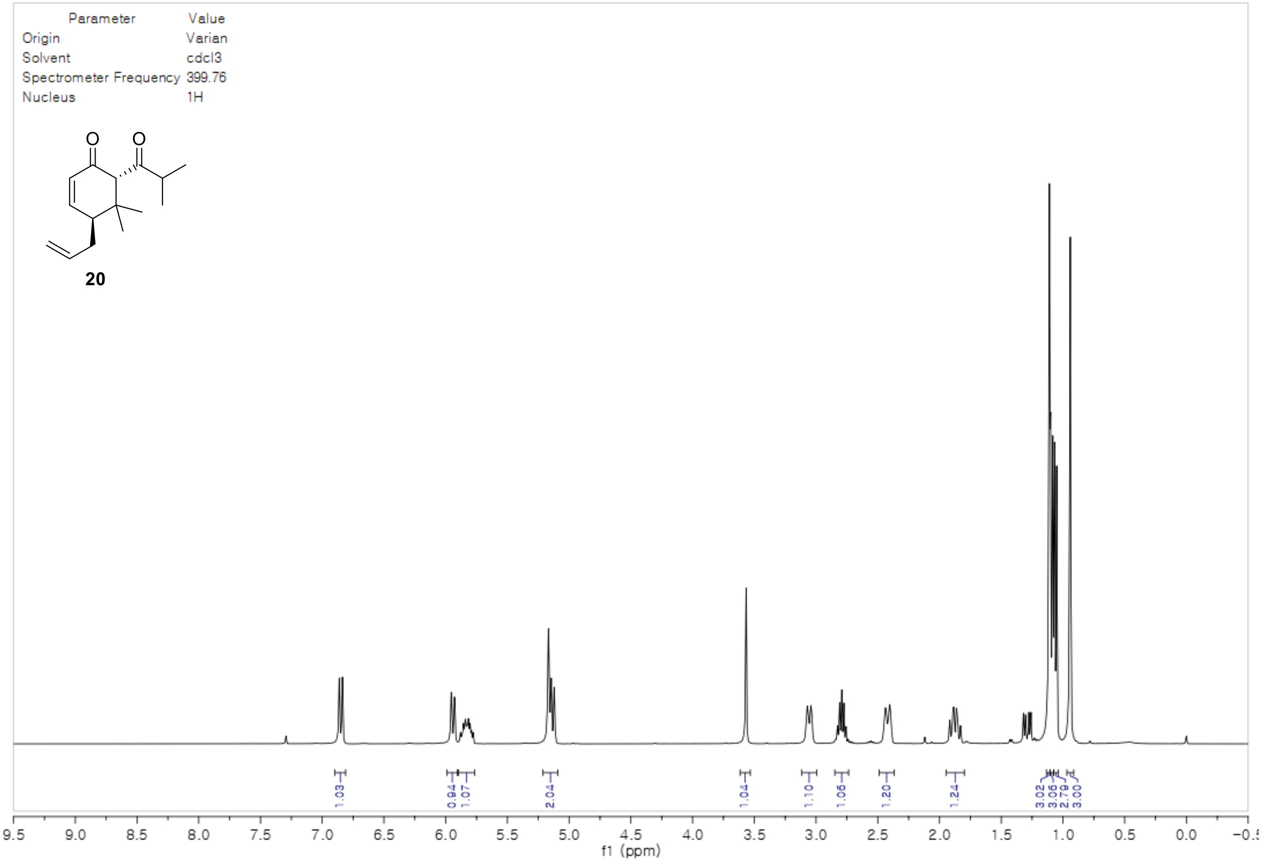

Compound 20 ^{13}C NMR

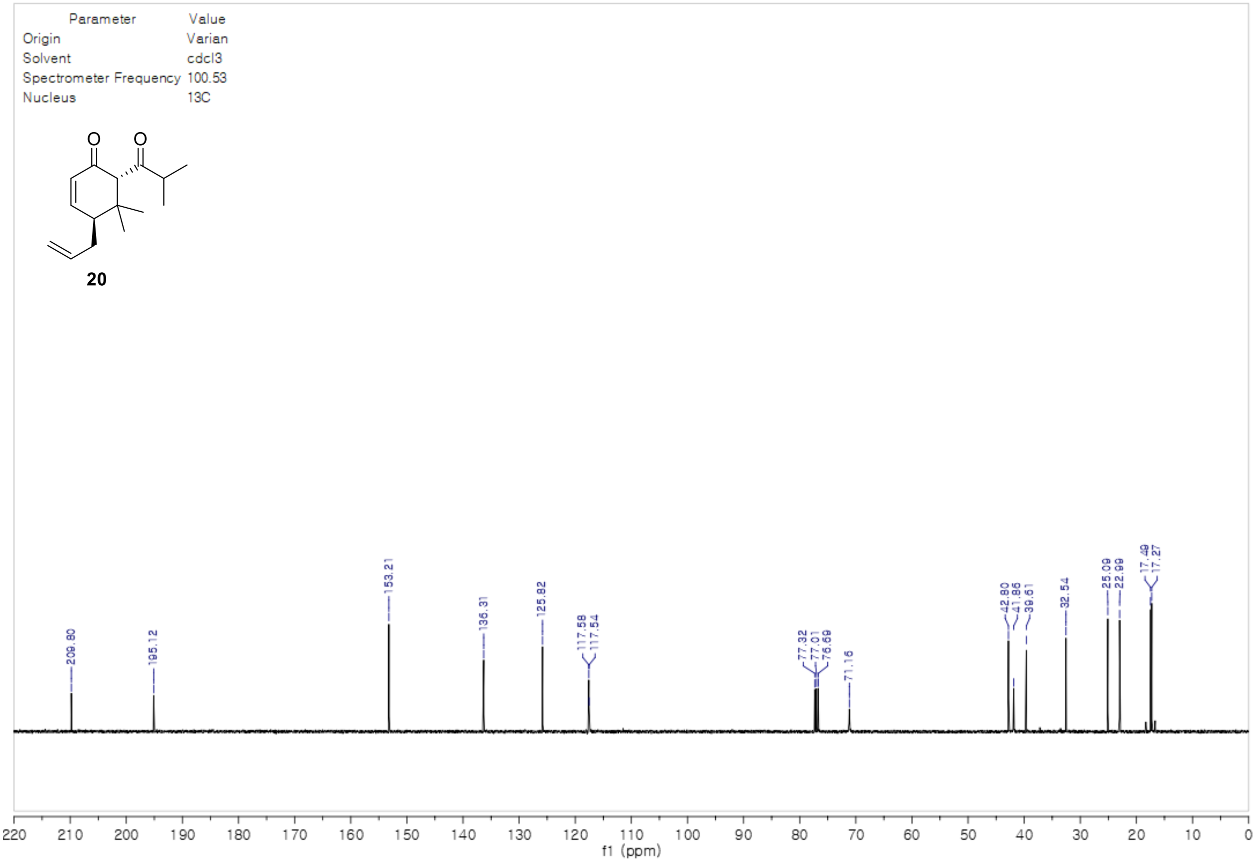

Compound 21 ^{1}H NMR

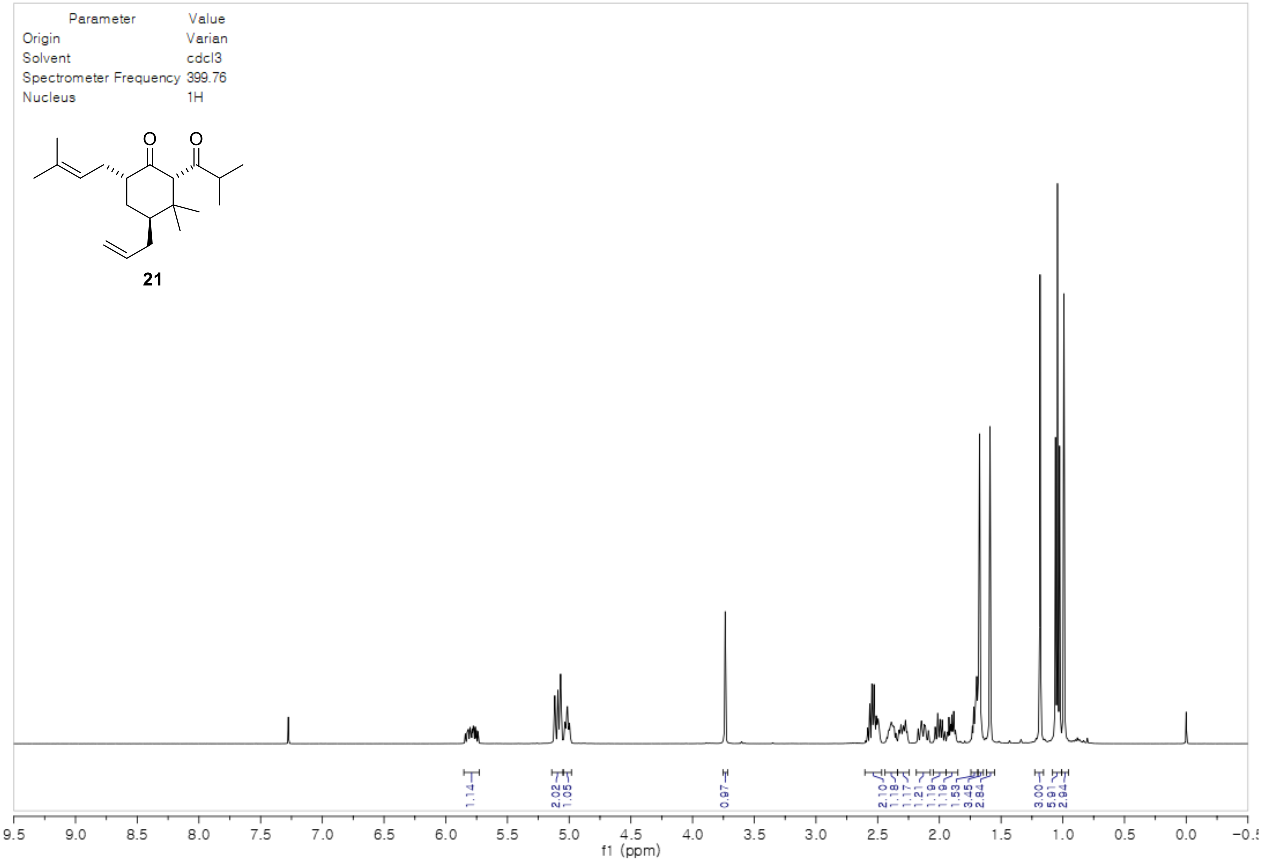

Compound 21 ^{13}C NMR

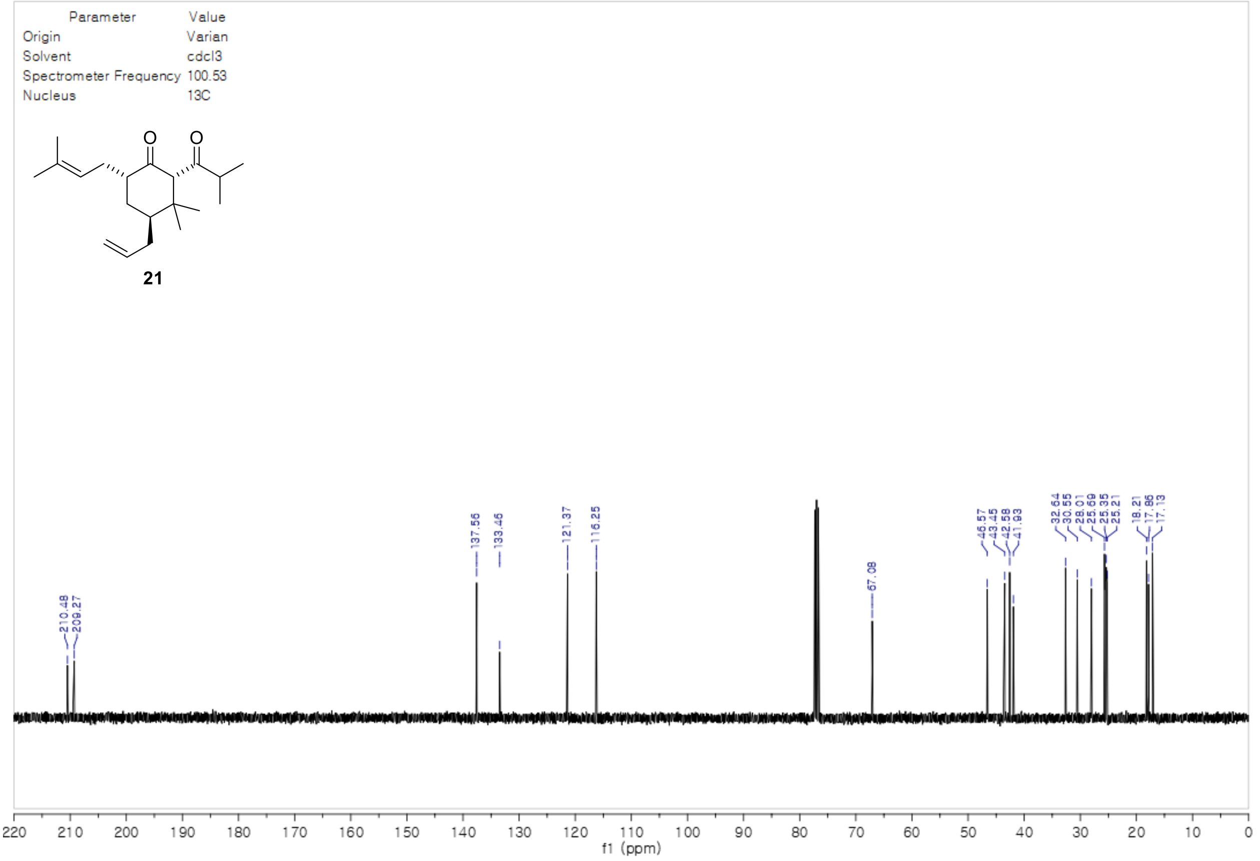

Compound 21 COSY

Parameter	Value (f2, f1)
1 Origin	Varian
2 Solvent	cdcl3
3 Pulse Sequence	gCOSY
4 Spectrometer Frequency	(399.76, 399.76)
5 Nucleus	(1H, 1H)

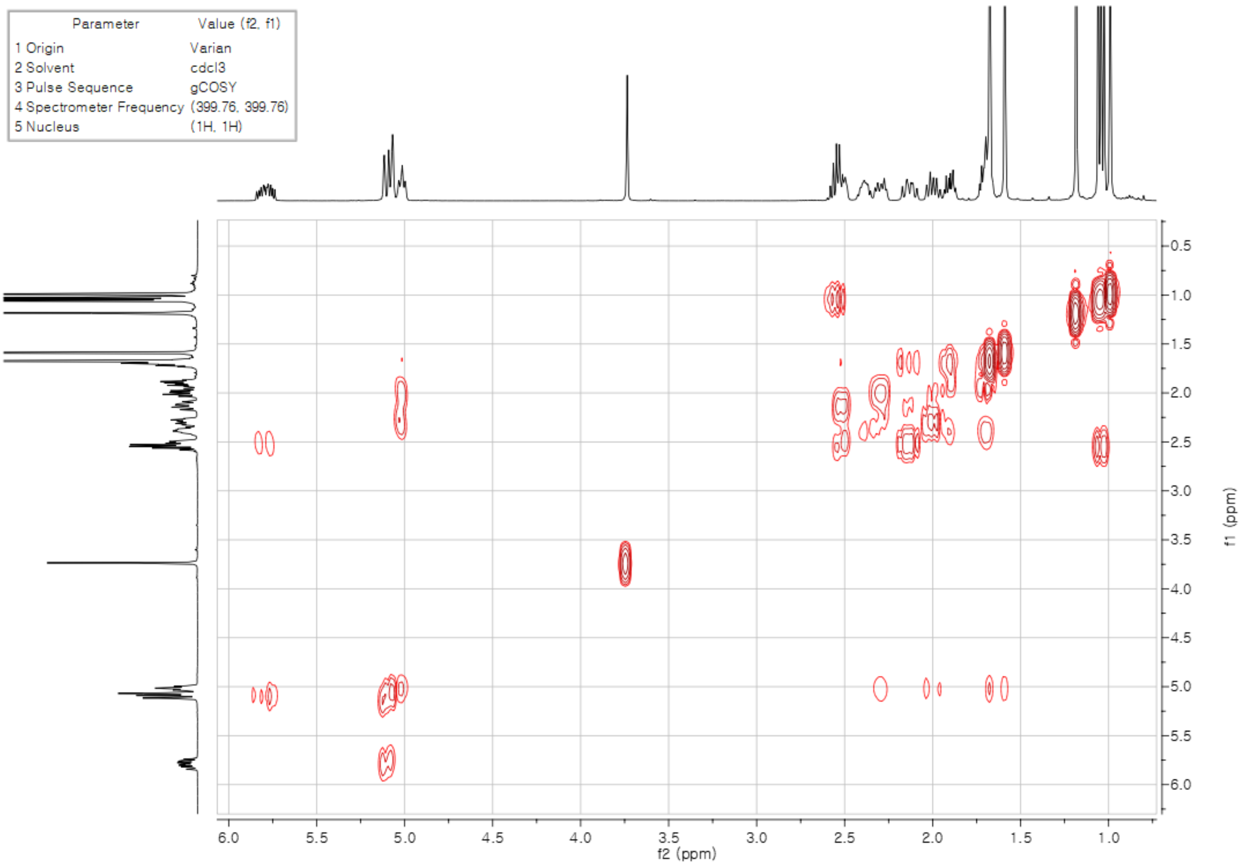

Compound 21 HMBC

Parameter	Value (f2, f1)
1 Origin	Varian
2 Solvent	cdcl3
3 Pulse Sequence	gHMBCAD
4 Spectrometer Frequency	(399.76, 100.53)
5 Nucleus	(1H, 13C)

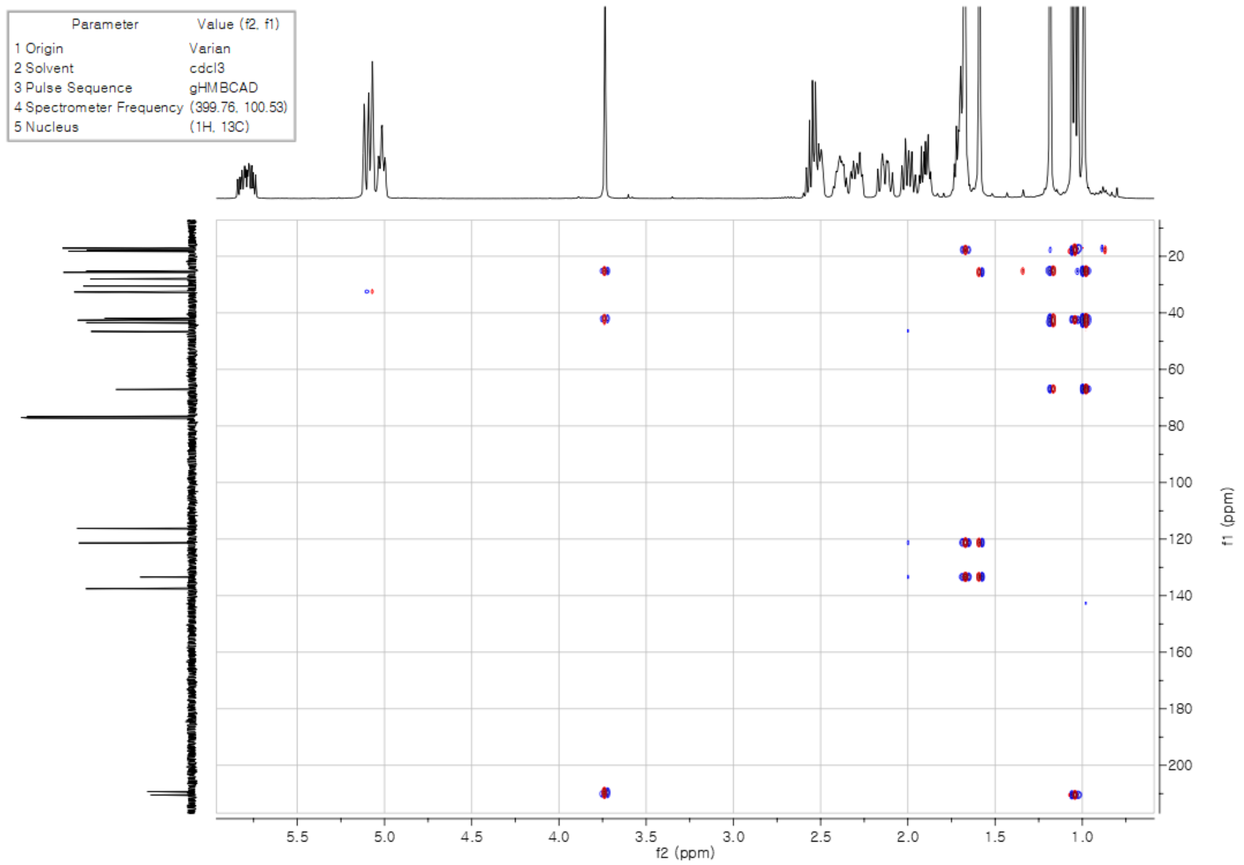

Compound 21 1D NOESY

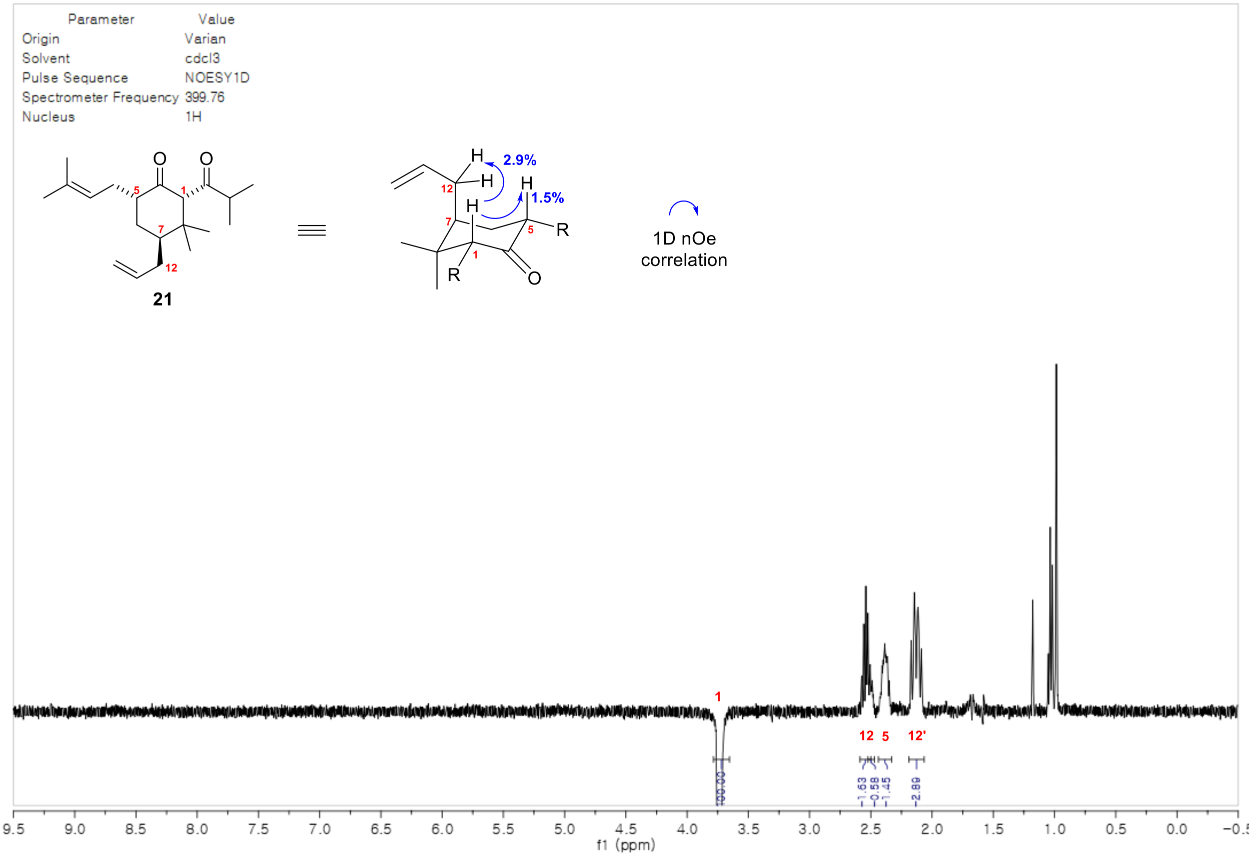

Ethyl 3-chloropropiolate ^{1}H NMR

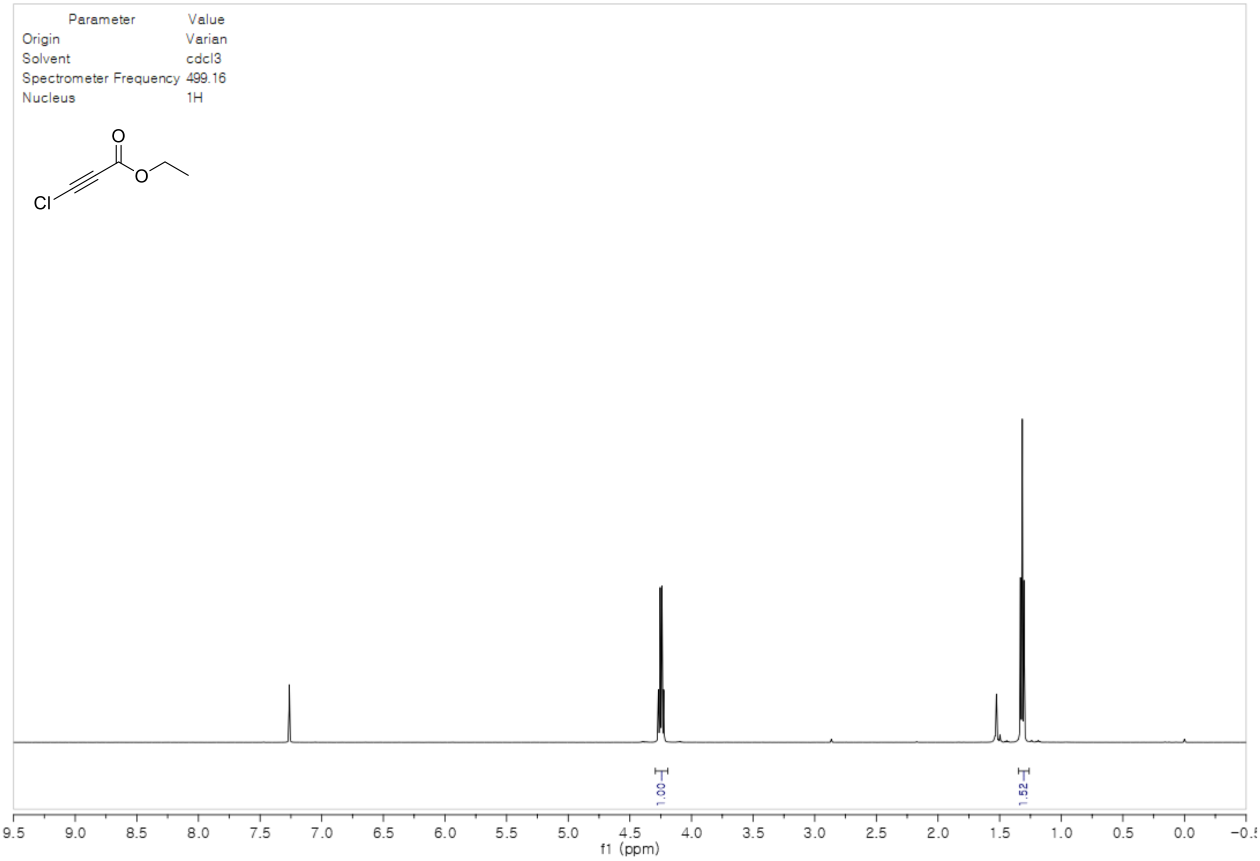

Ethyl 3-chloropropiolate ^{13}C NMR

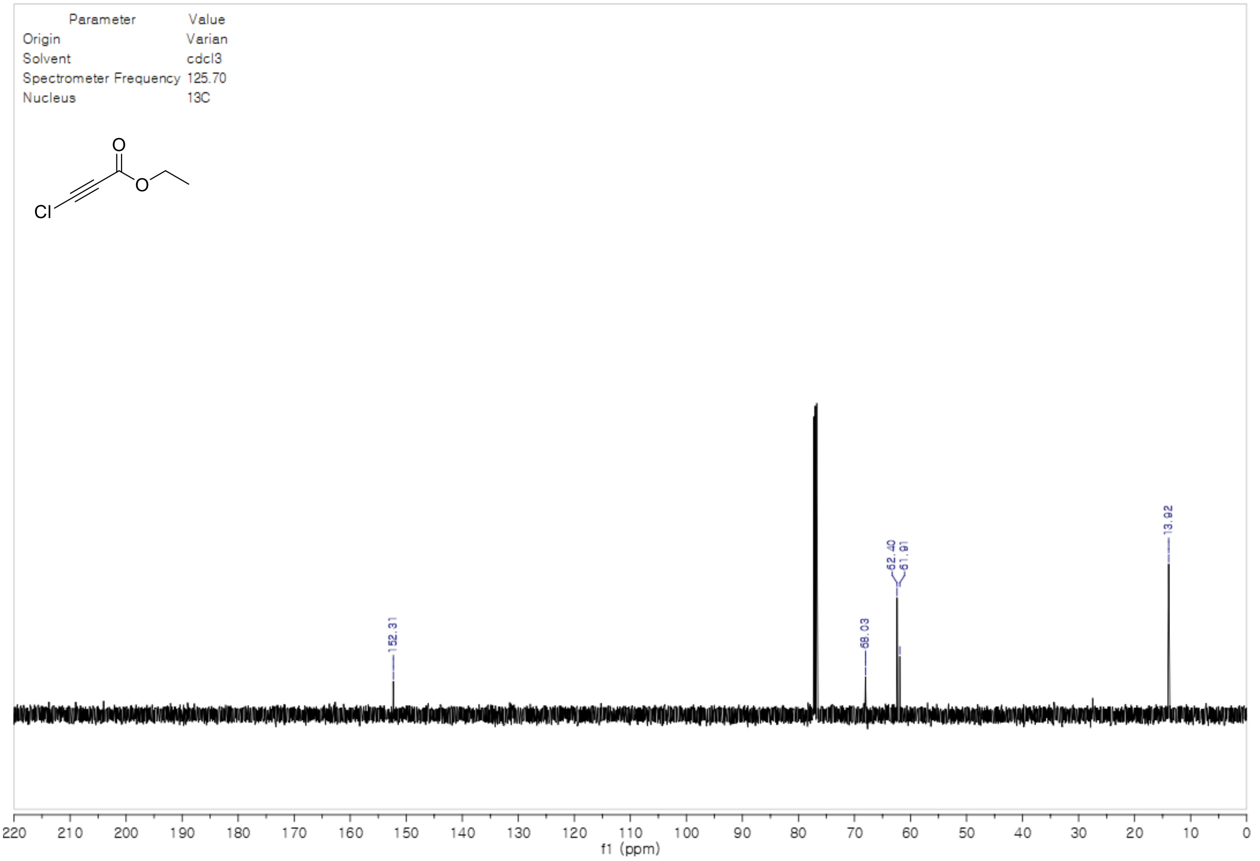

Compound 22b ¹H NMR

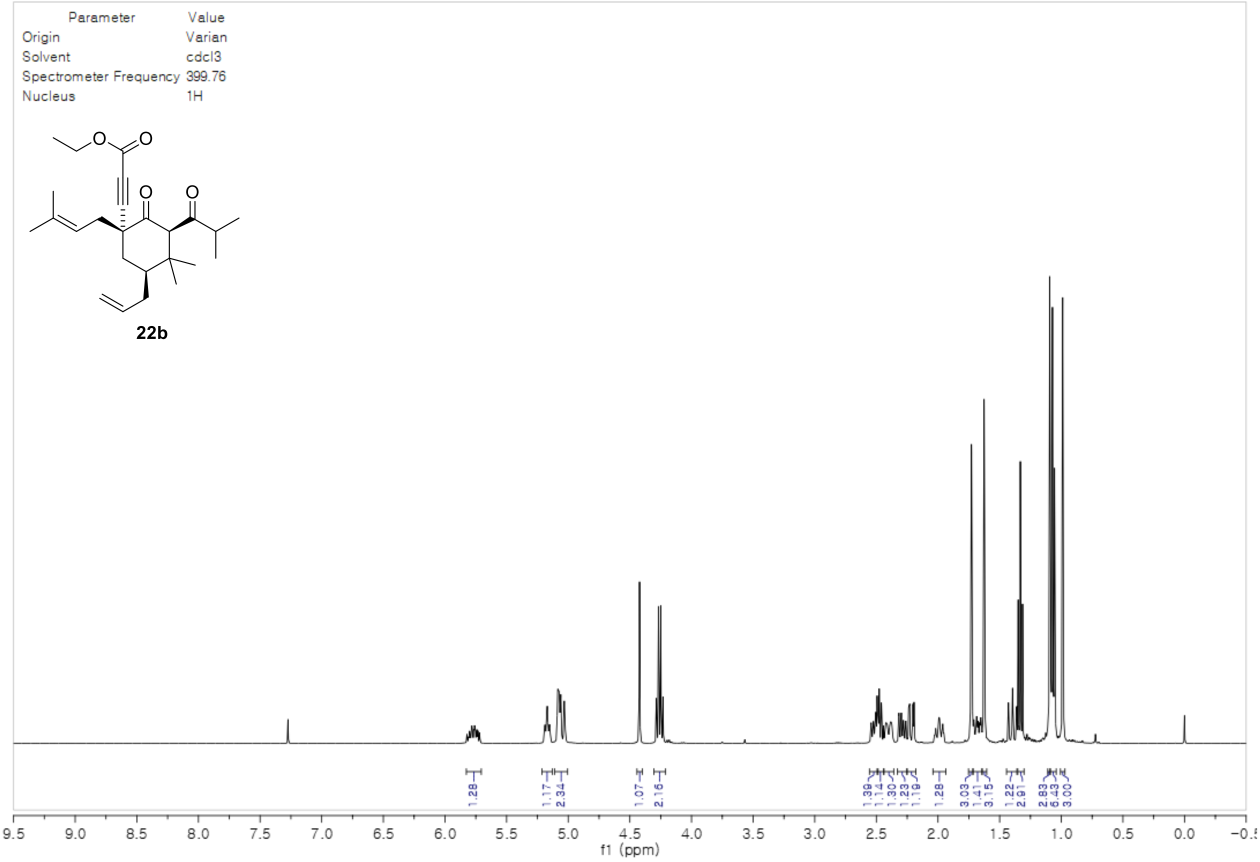

Compound 22b ¹³C NMR

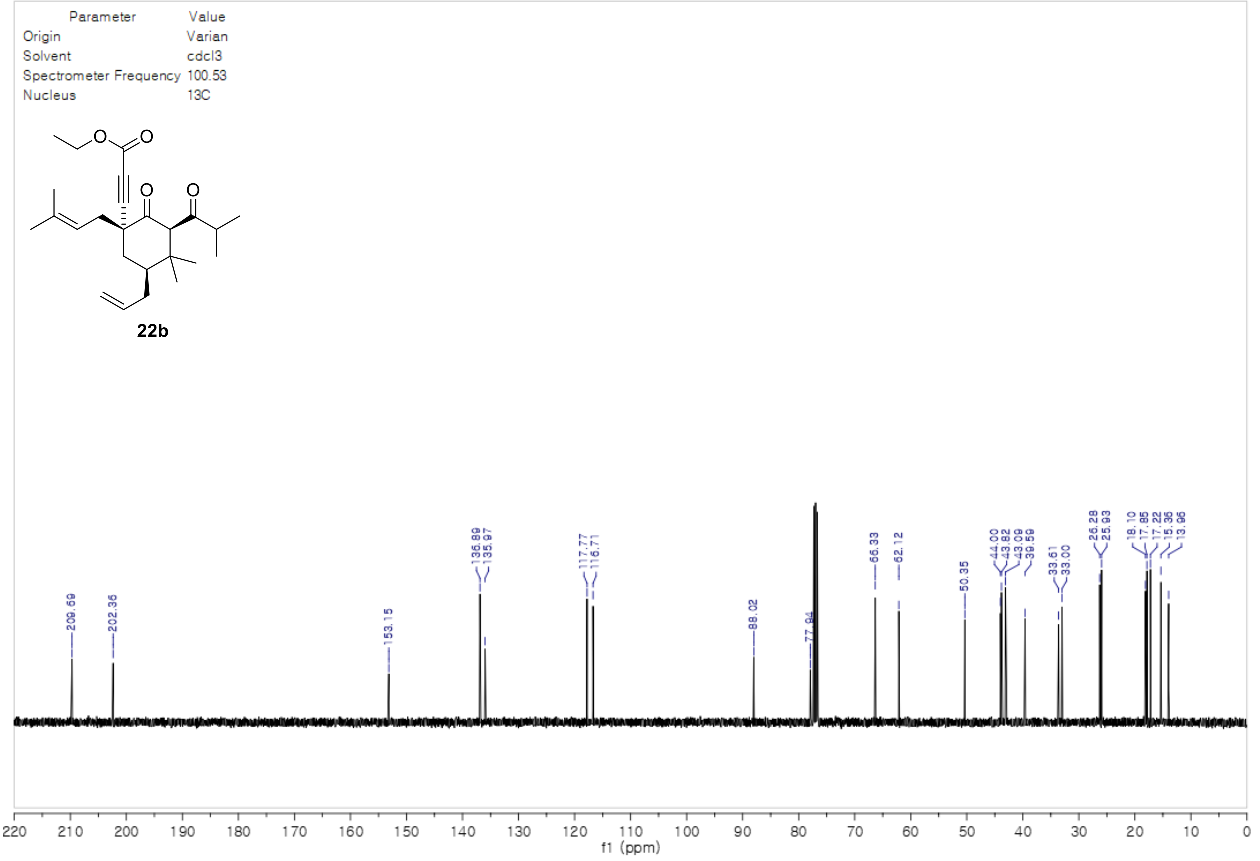

Compound 22b COSY

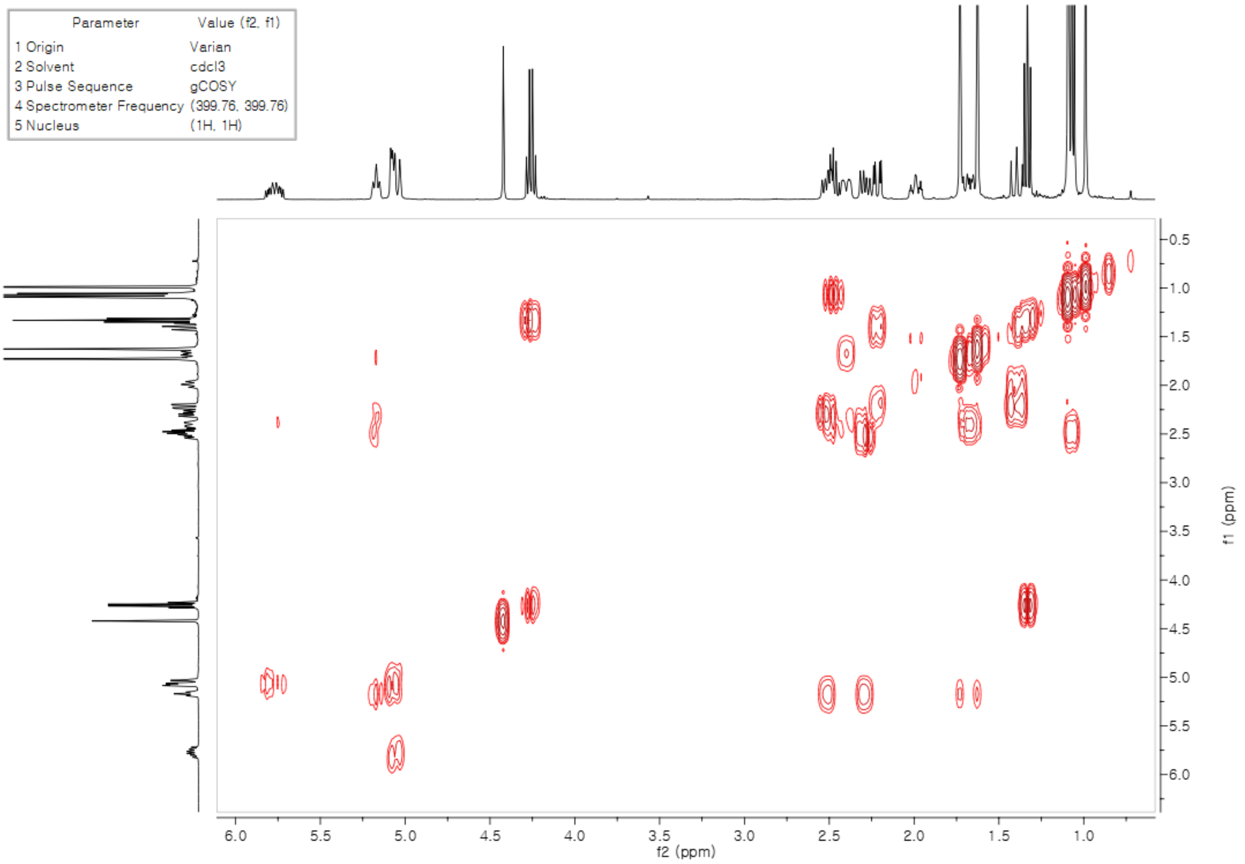

Compound 22b HSQC

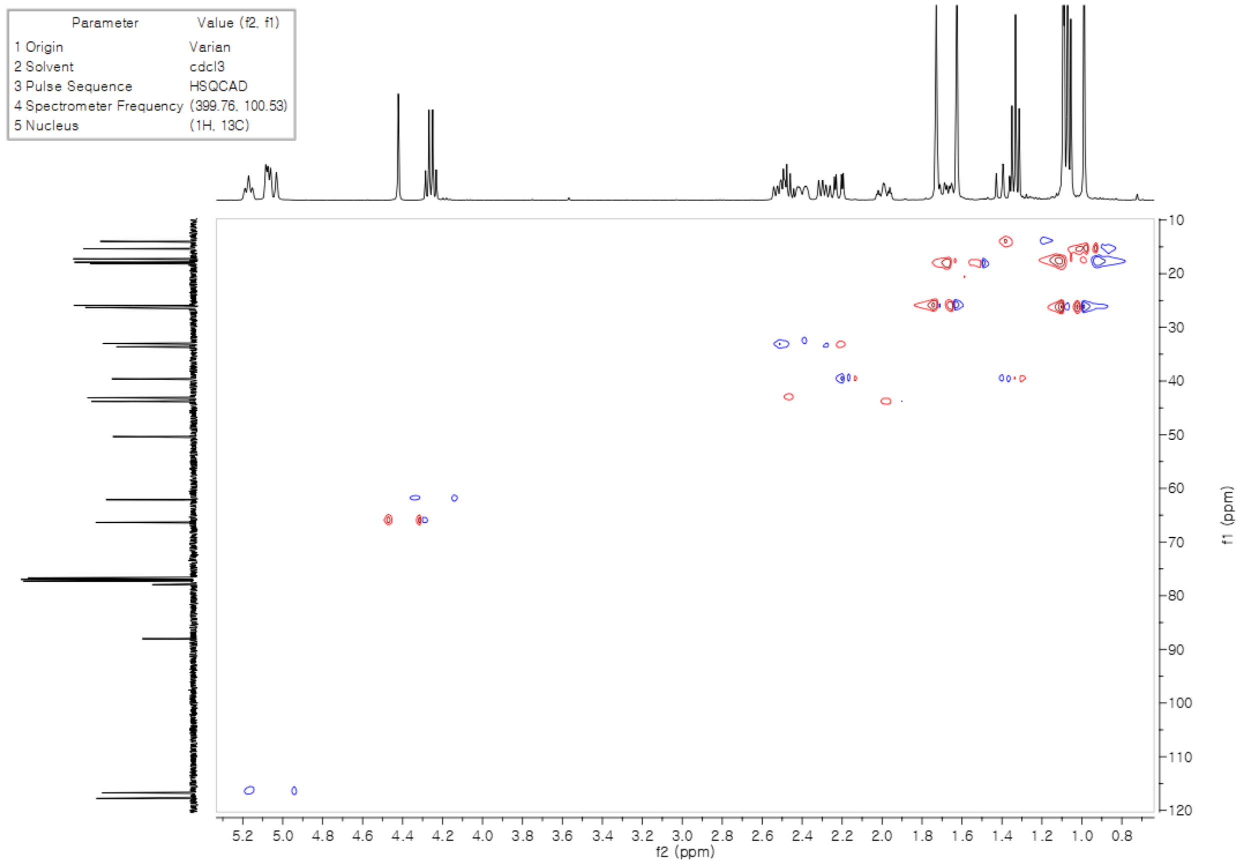

Compound 22b HMBC

Parameter	Value (f2, f1)
1 Origin	Varian
2 Solvent	cdcl3
3 Pulse Sequence	gHMBCAD
4 Spectrometer Frequency	(399.76, 100.53)
5 Nucleus	(1H, 13C)

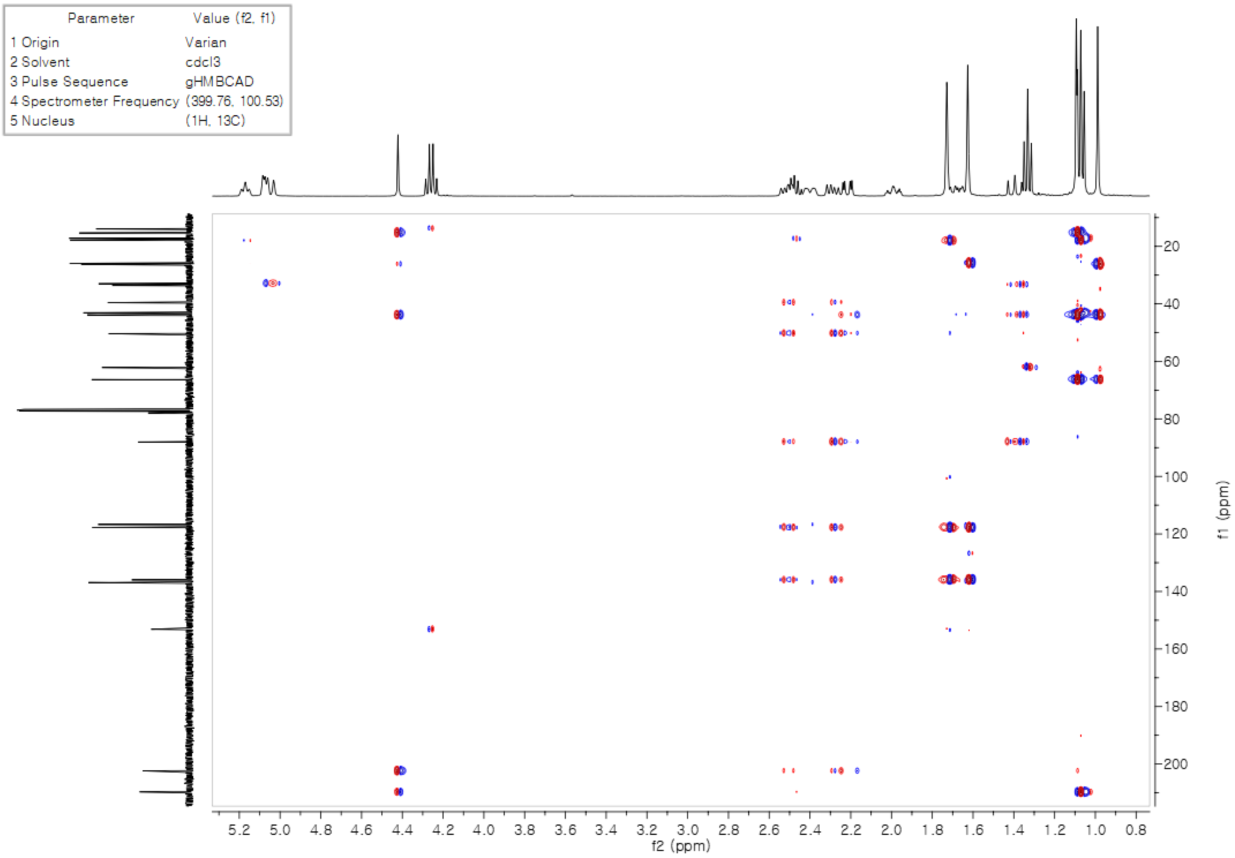

Compound 22b 1D NOESY

Parameter	Value
Origin	Varian
Solvent	cdcl3
Pulse Sequence	NOESY1D
Spectrometer Frequency	399.76
Nucleus	1H

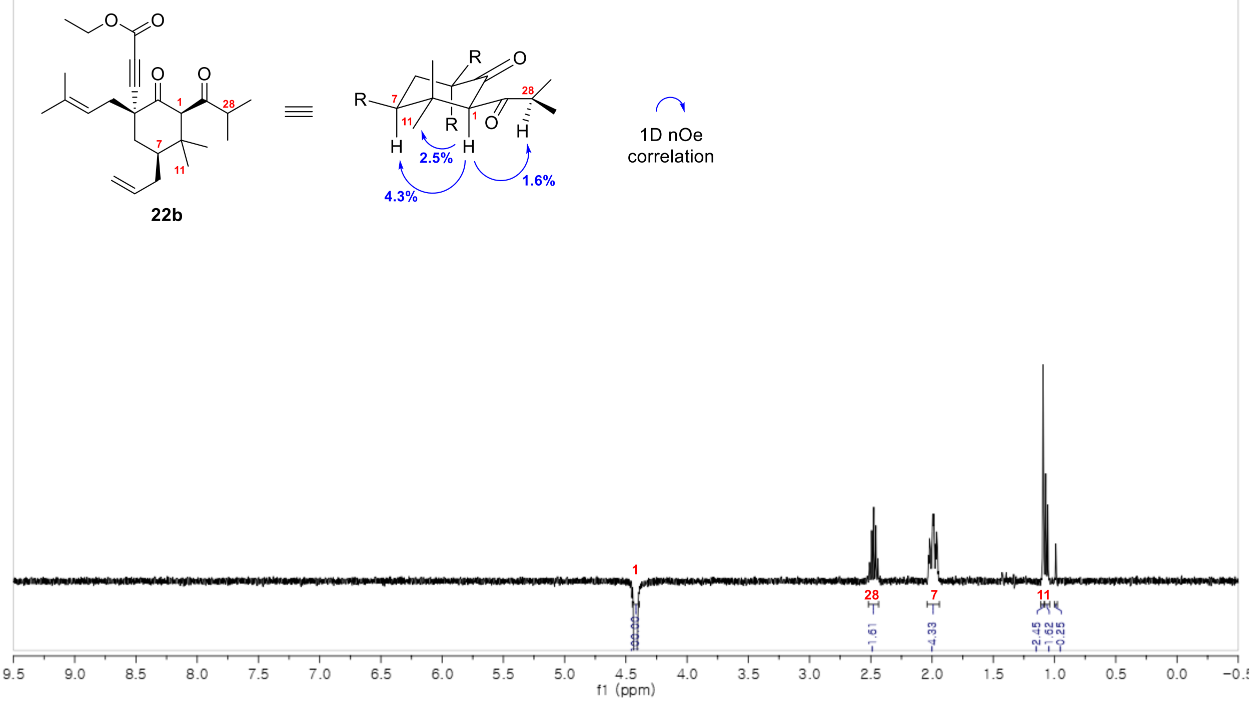

Compound 22a ^{1}H NMR

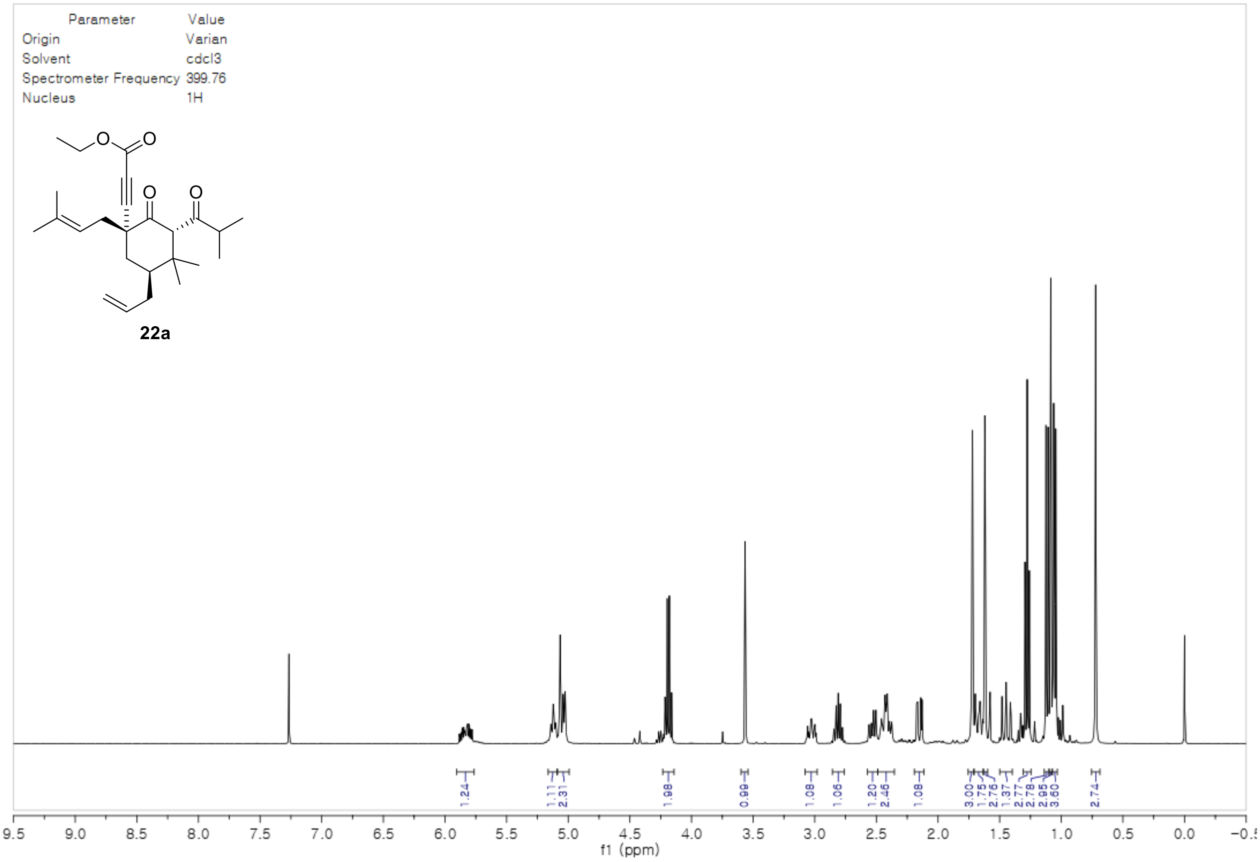

Compound 22a ^{13}C NMR

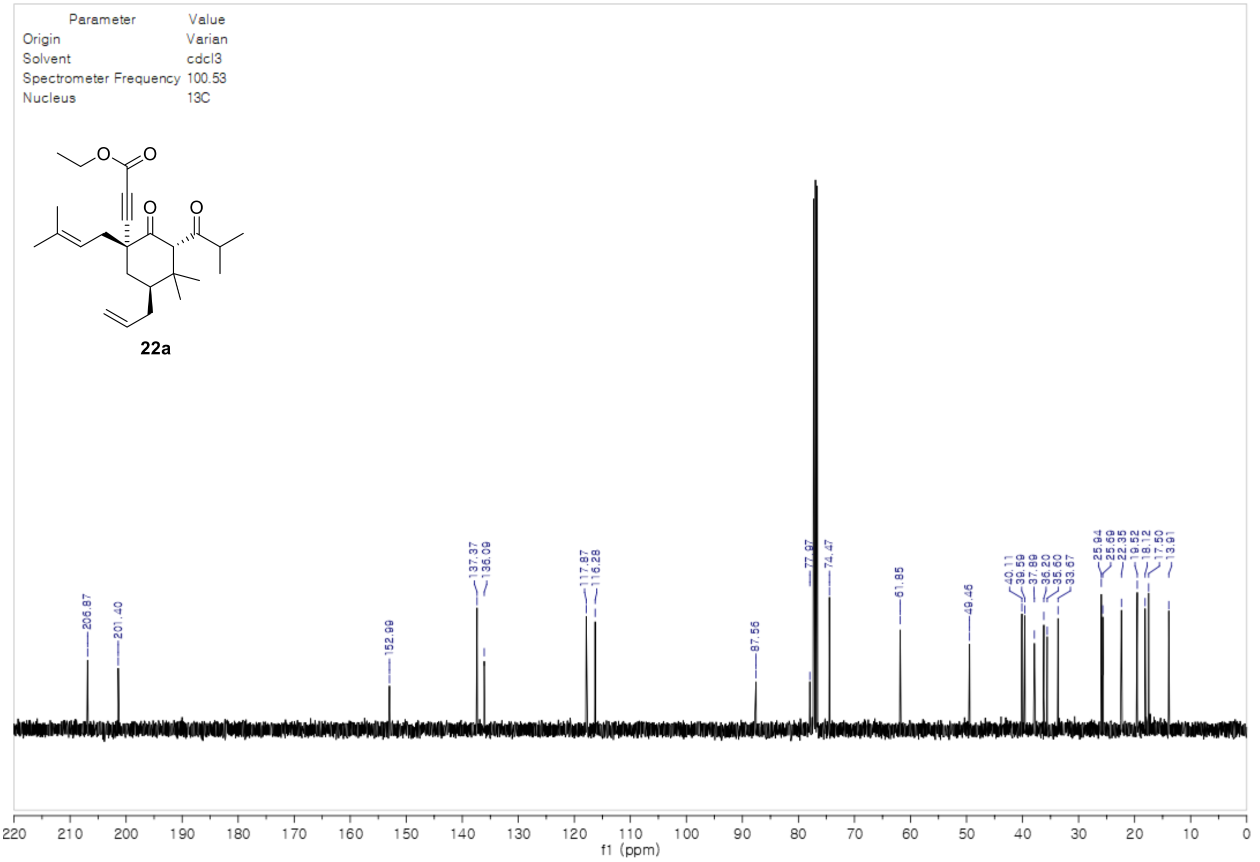

Compound 22a COSY

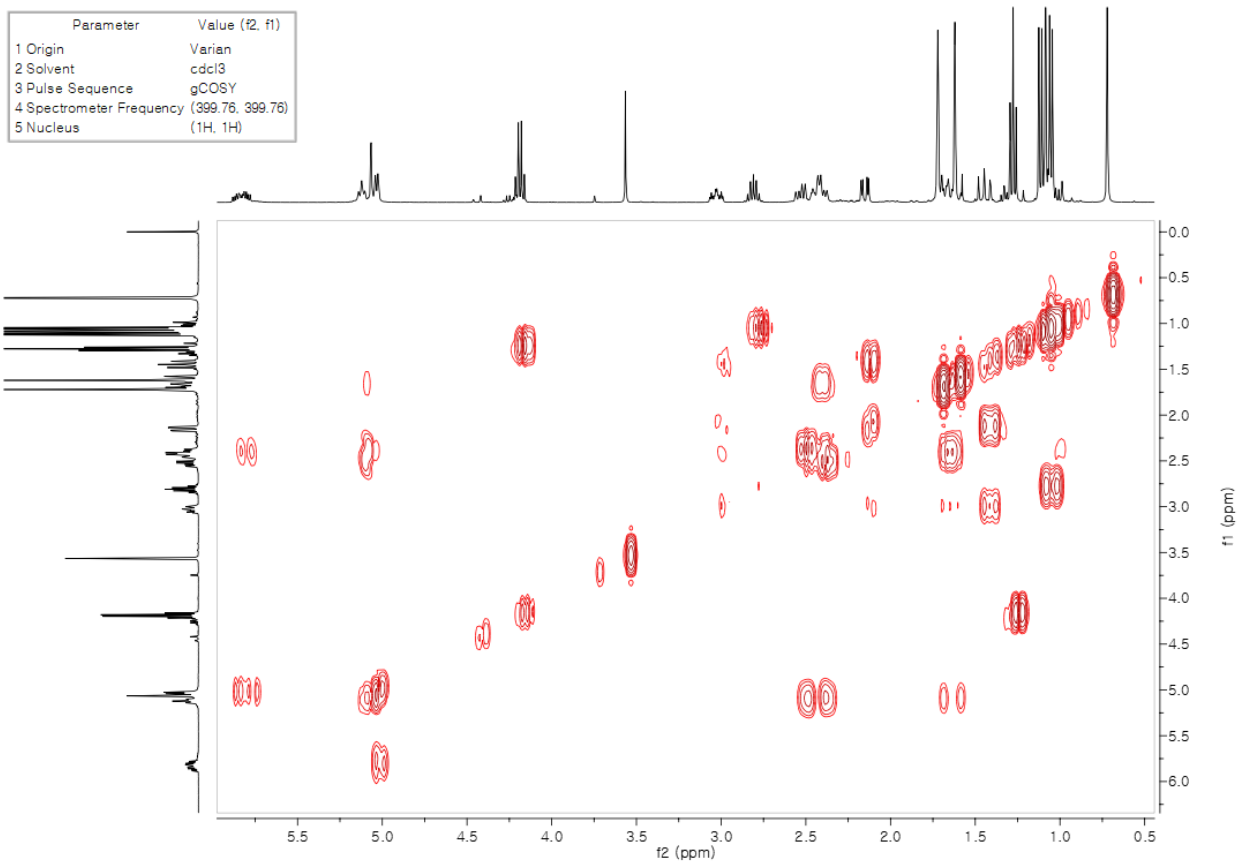

Compound 22a 1D NOESY

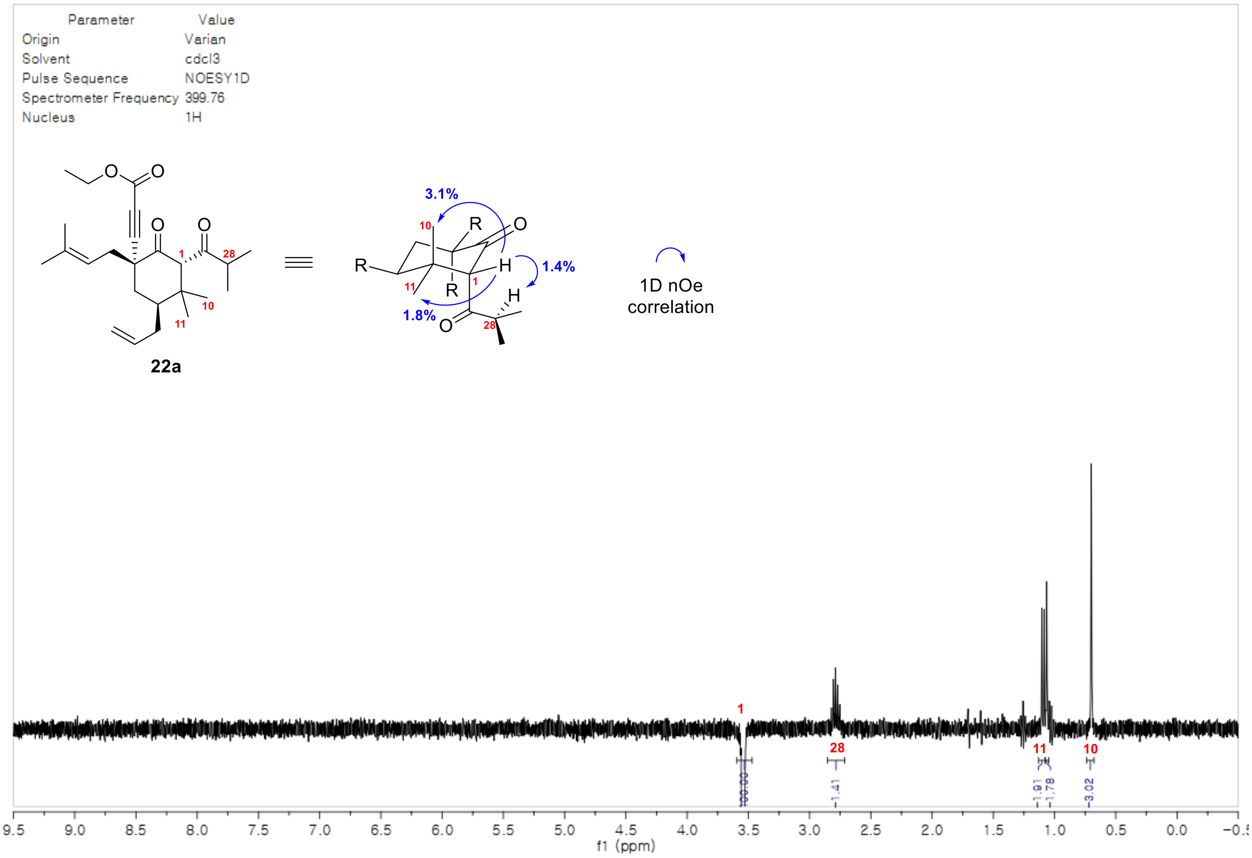

Compound 22' ^{1}H NMR

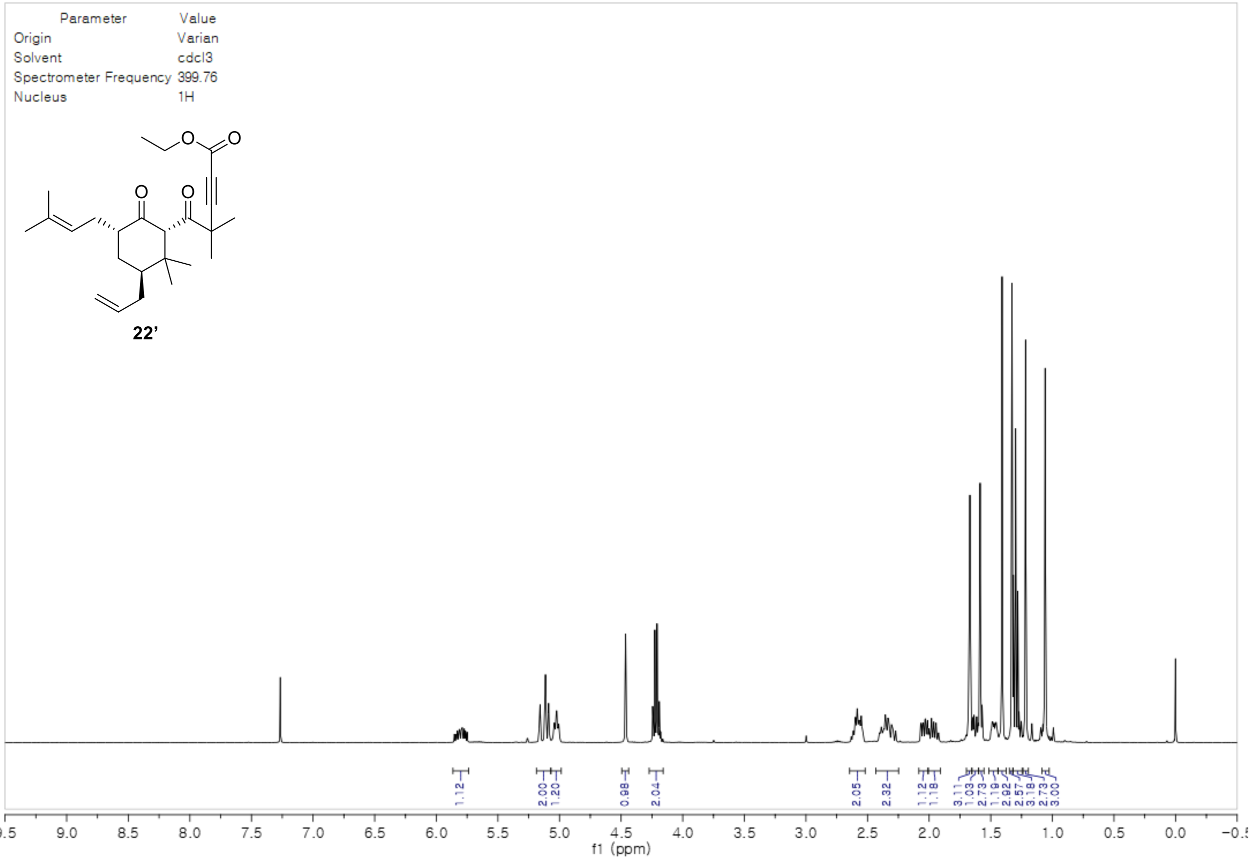

Compound 22' ^{13}C NMR

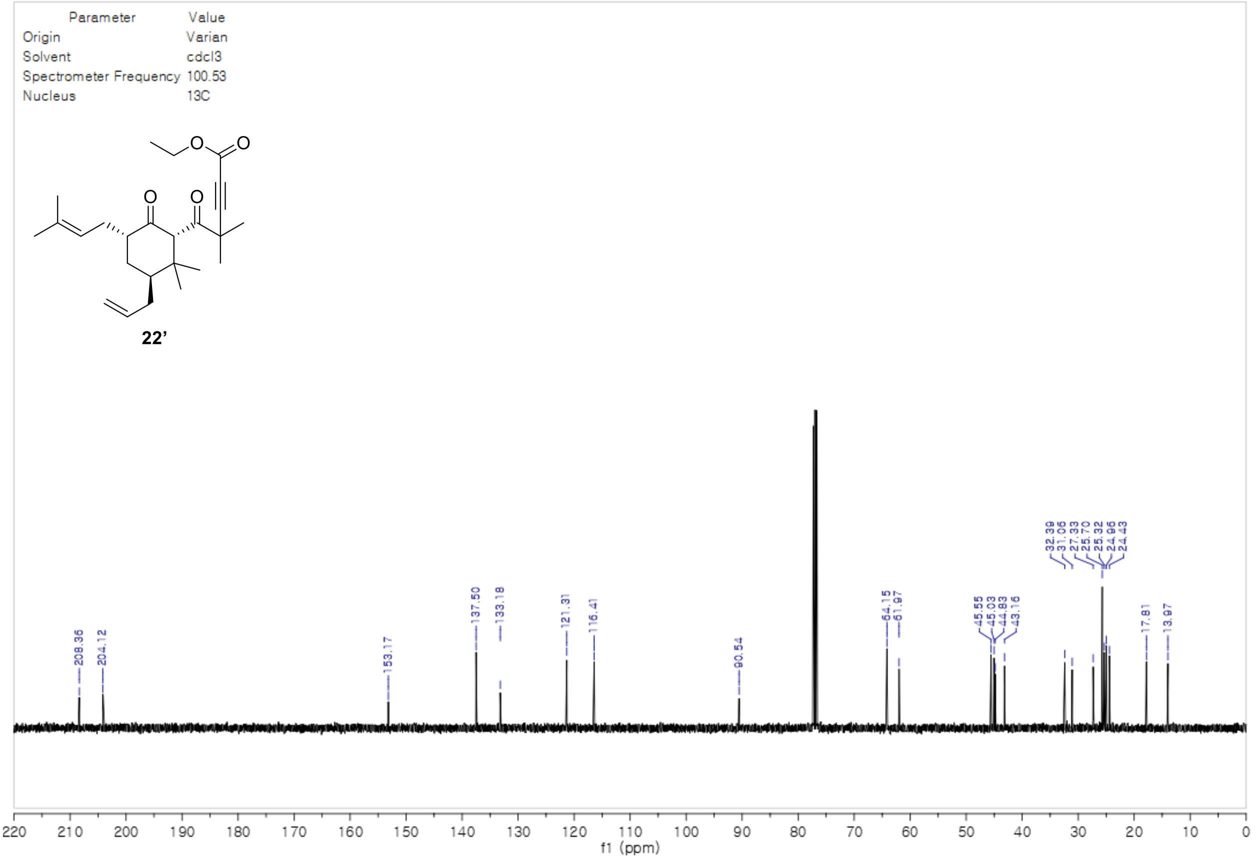

Compound 22' COSY

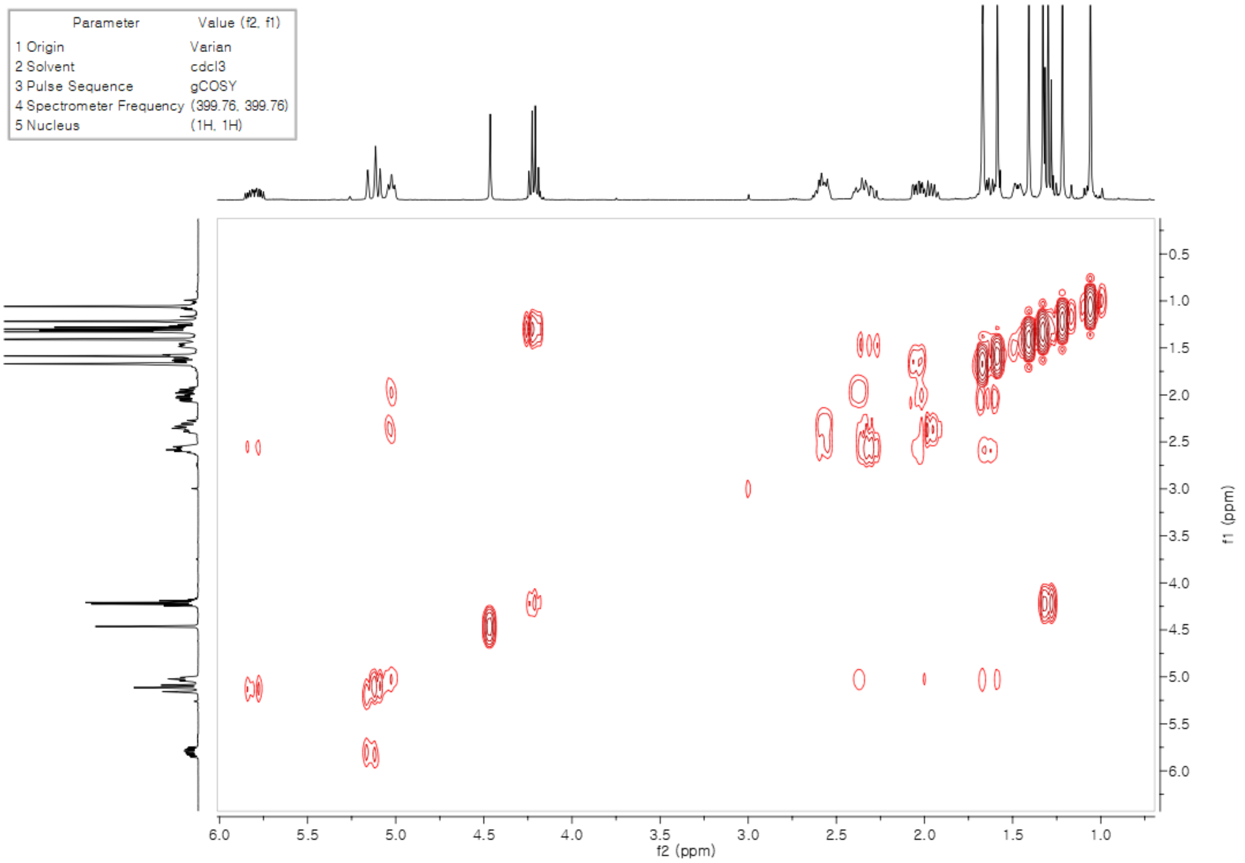

Compound 22' HSQC

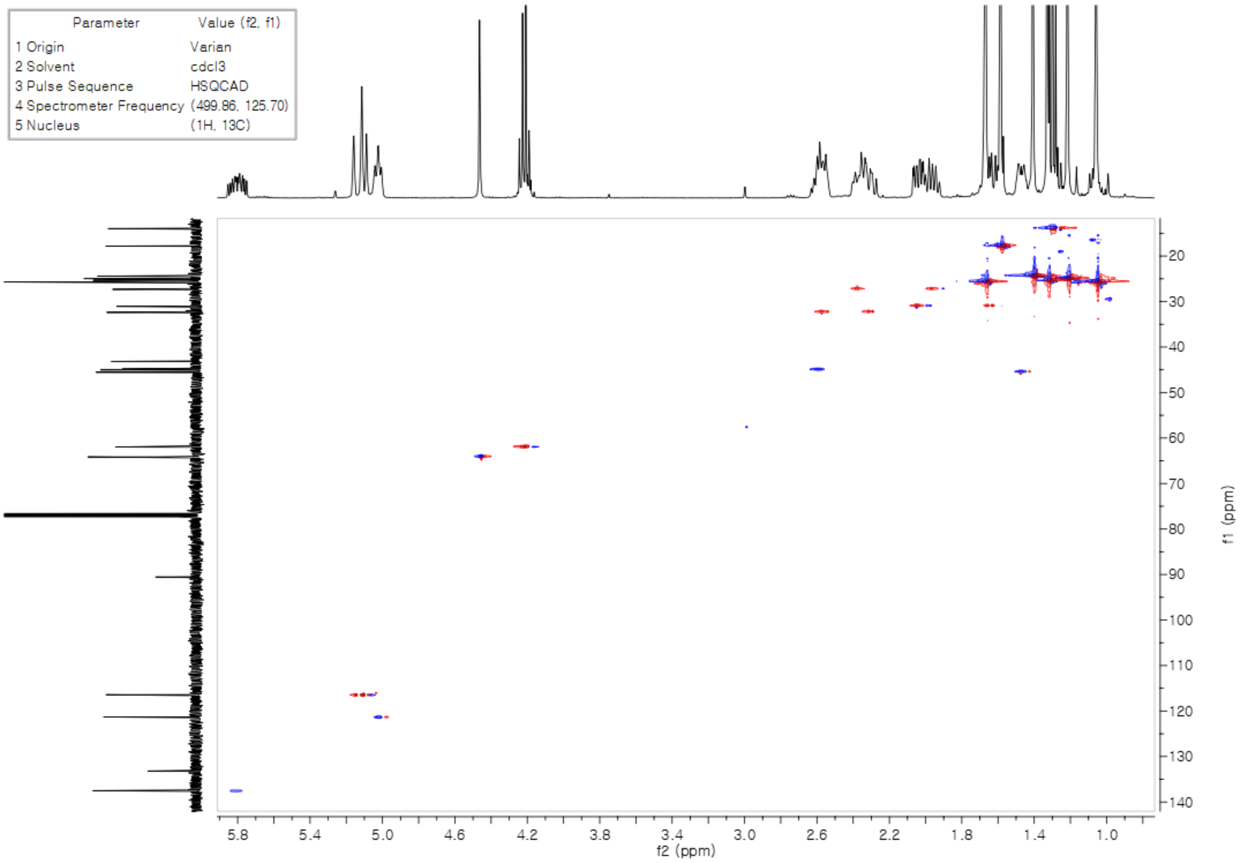

Compound 22' HMBC

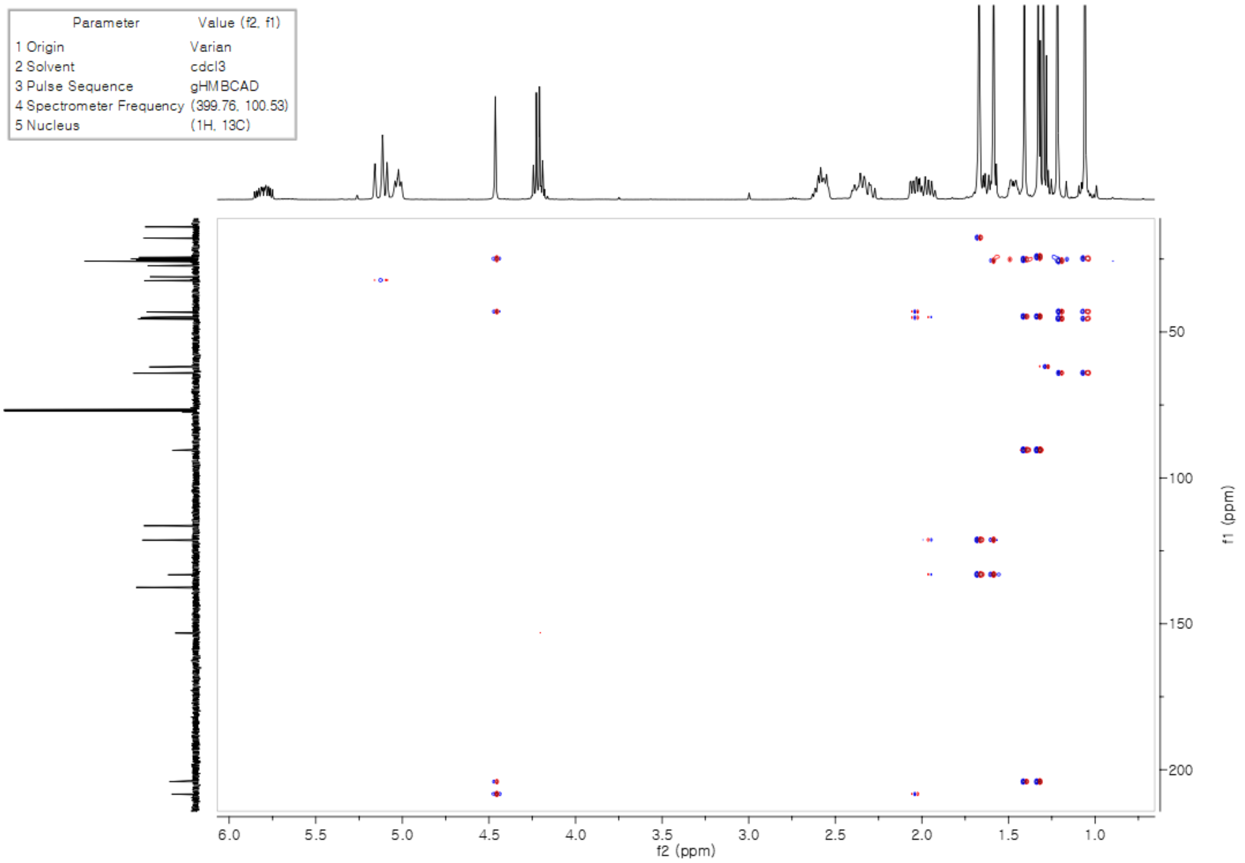

Compound 22' 1D NOESY

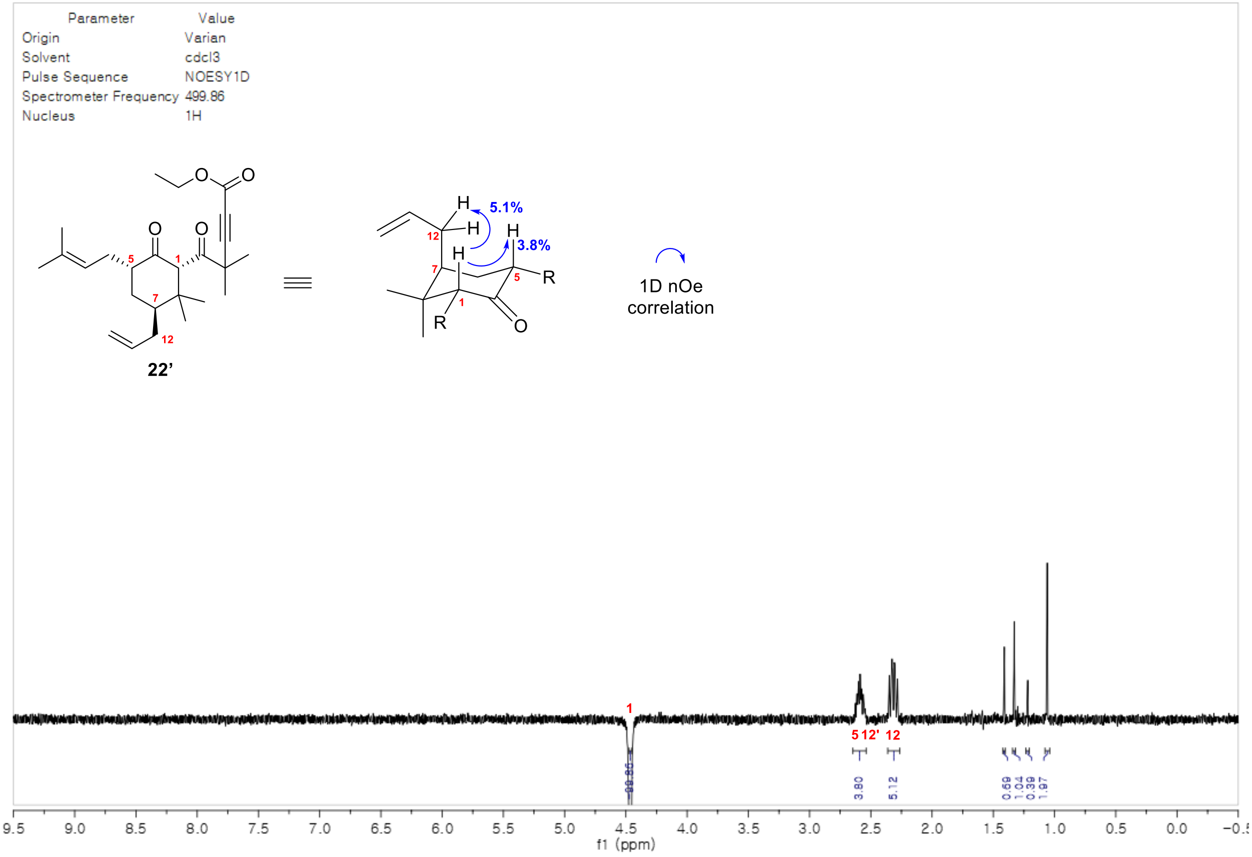

Compound 23 ^{1}H NMR

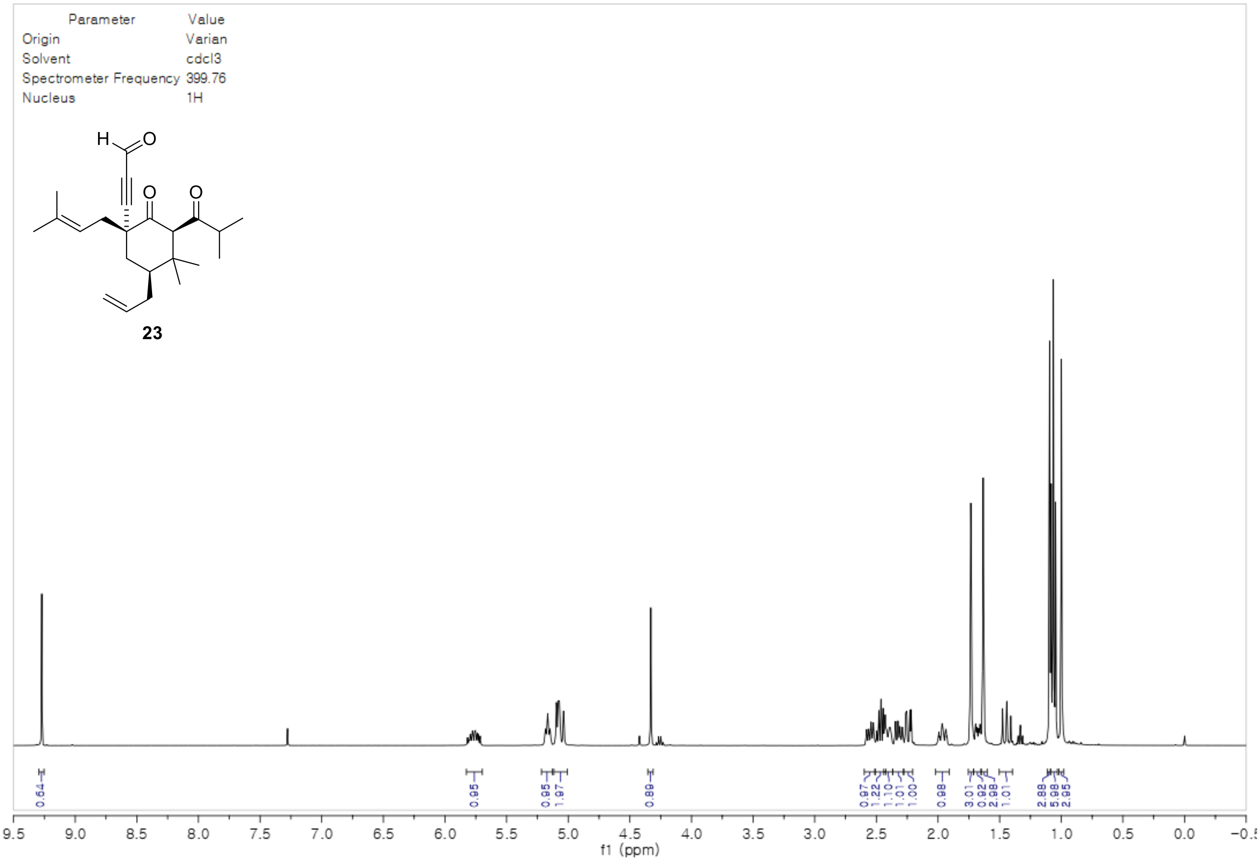

Compound 23 ^{13}C NMR

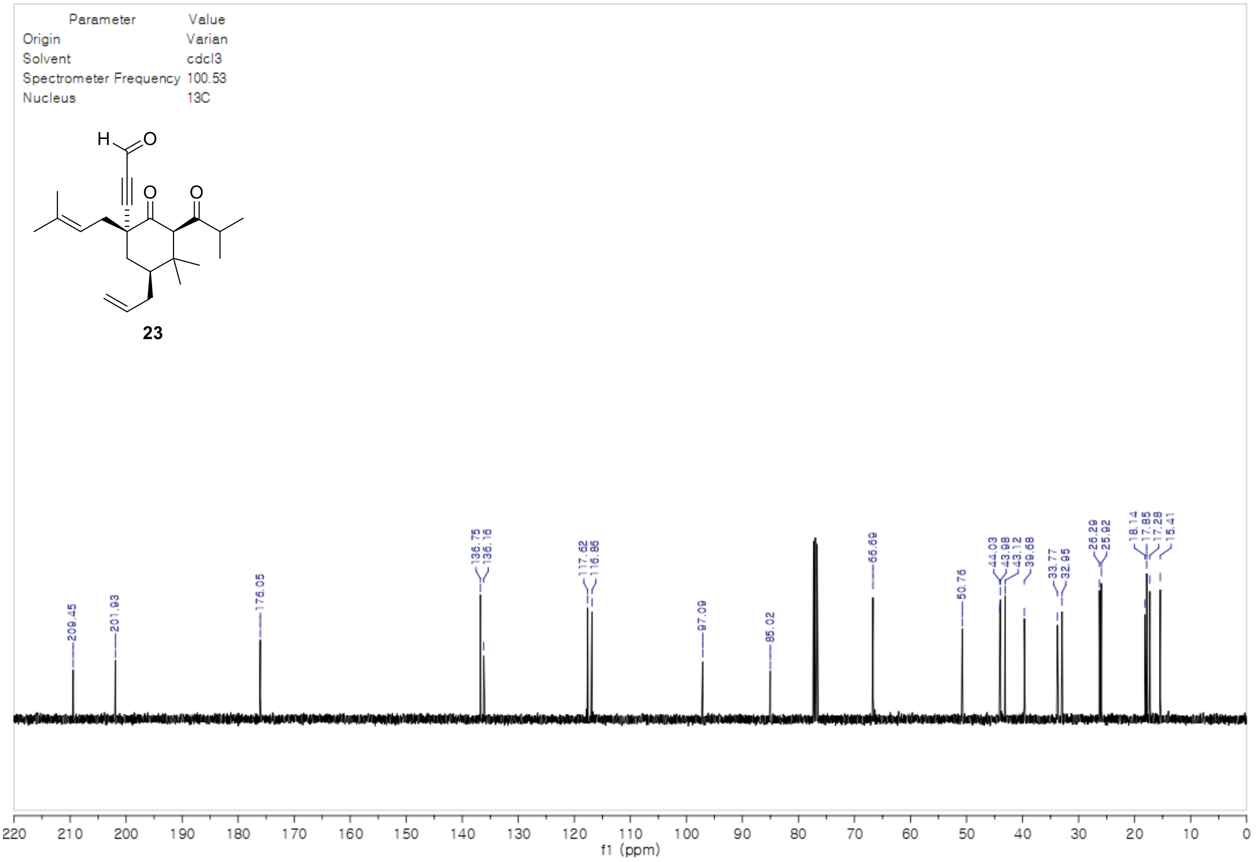

Compound 23' ¹H NMR

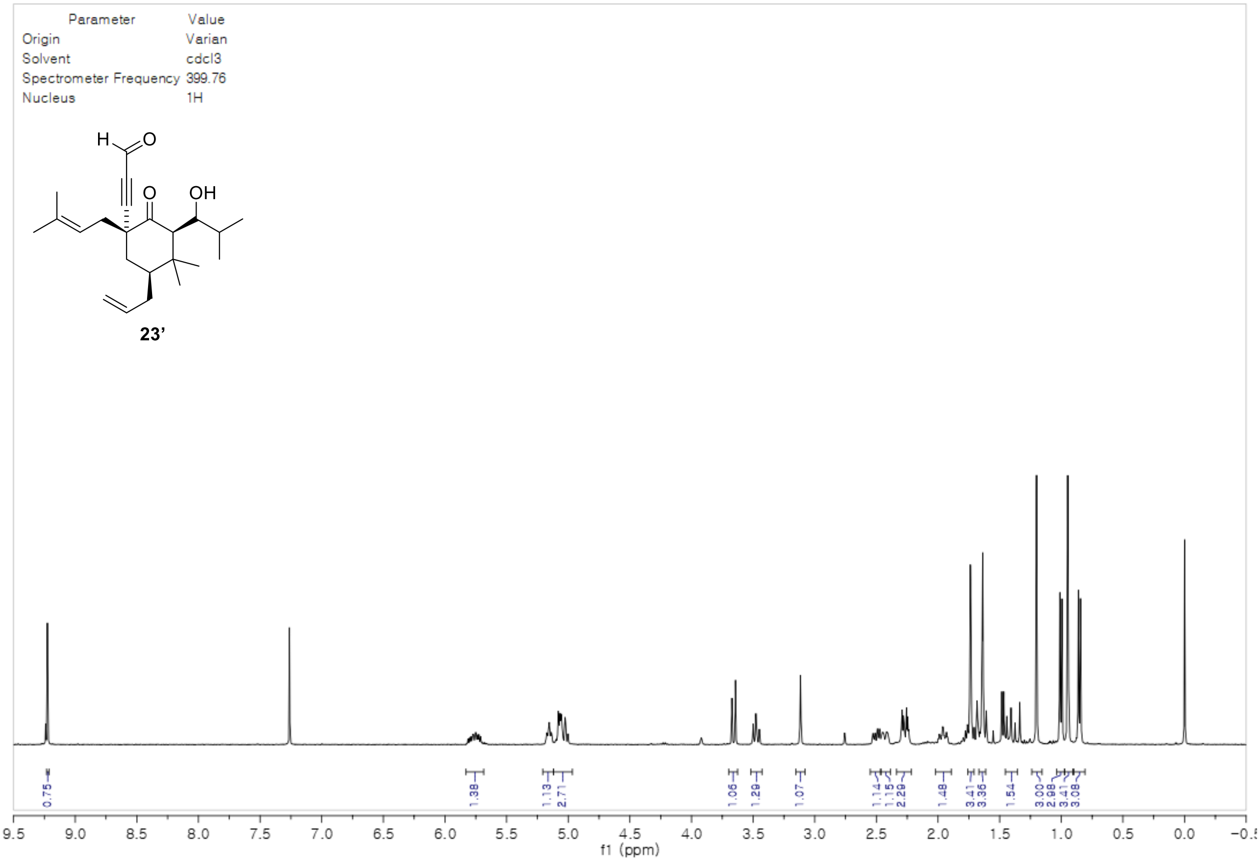

Compound 23' ¹³C NMR

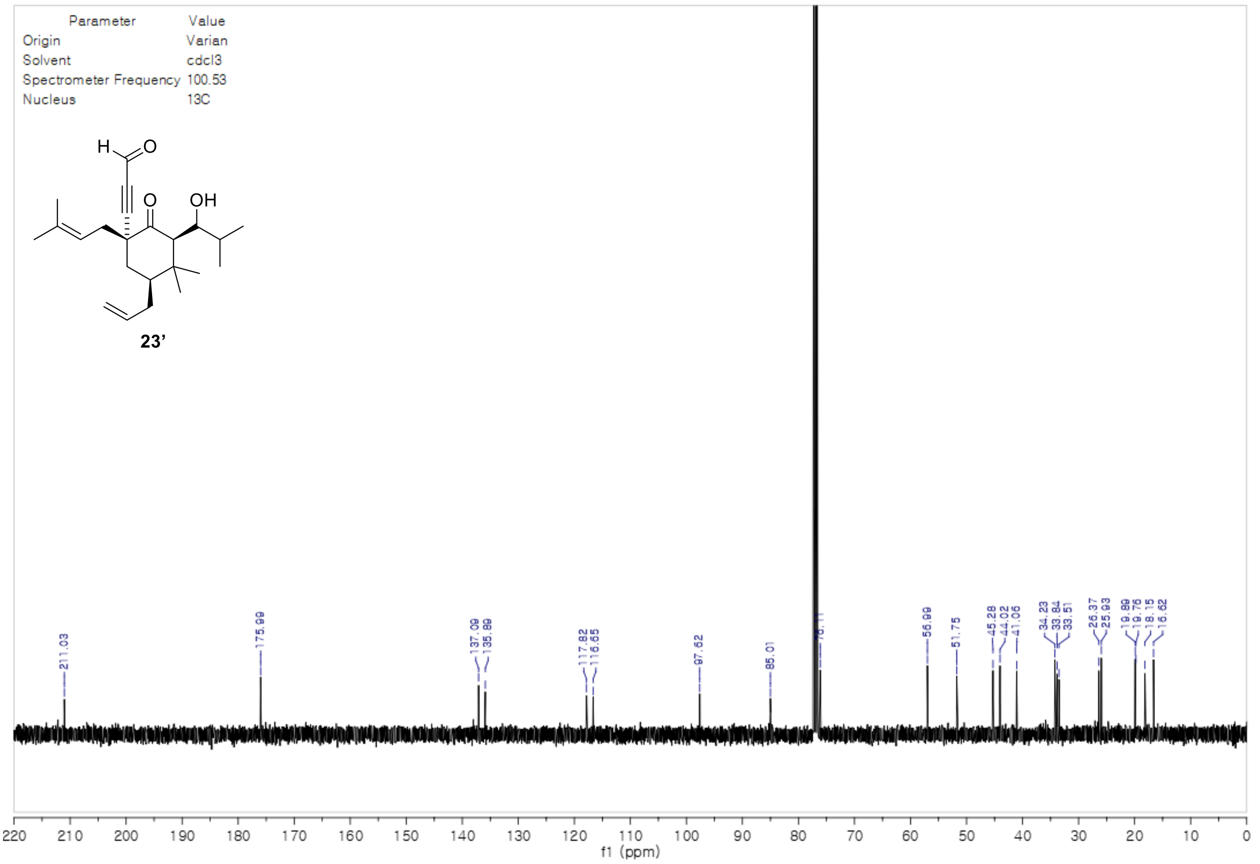

Compound 23' COSY

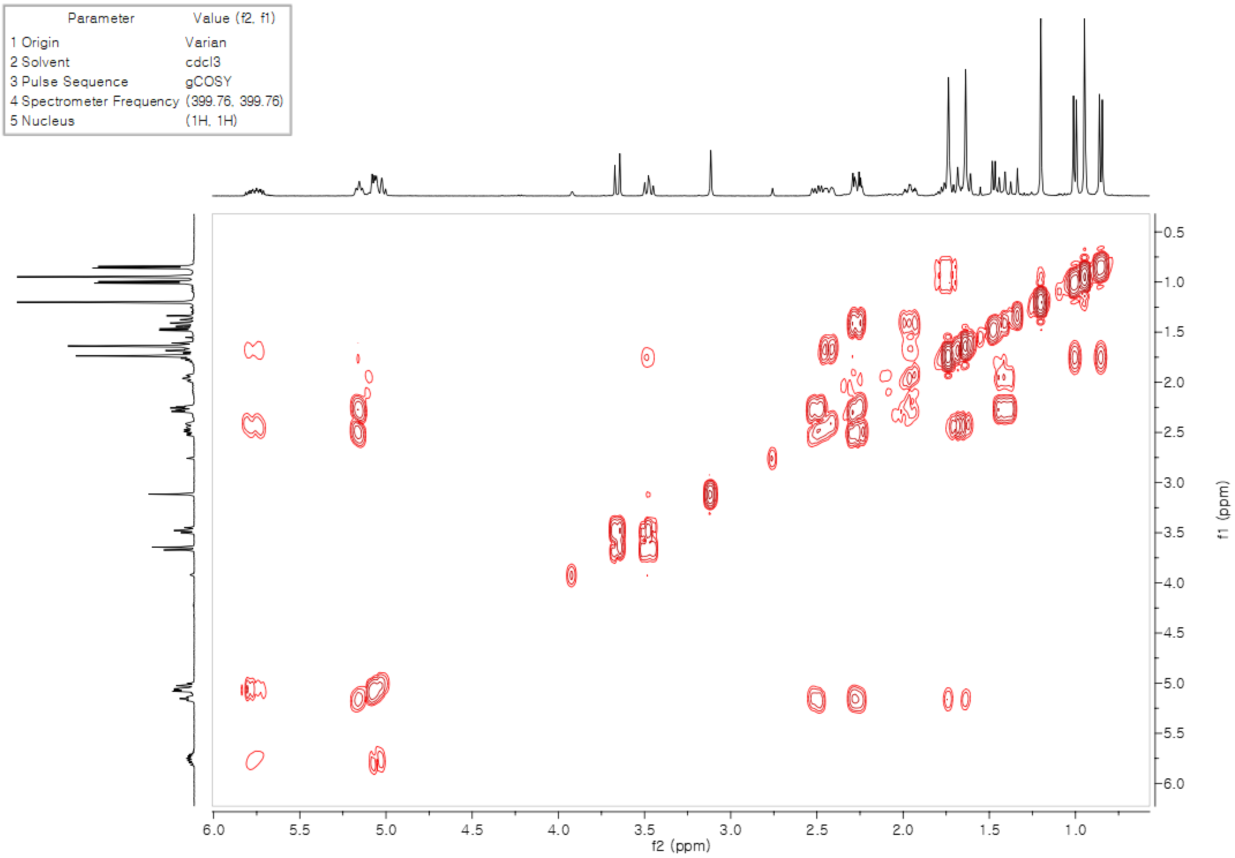

Compound 24' ¹H NMR

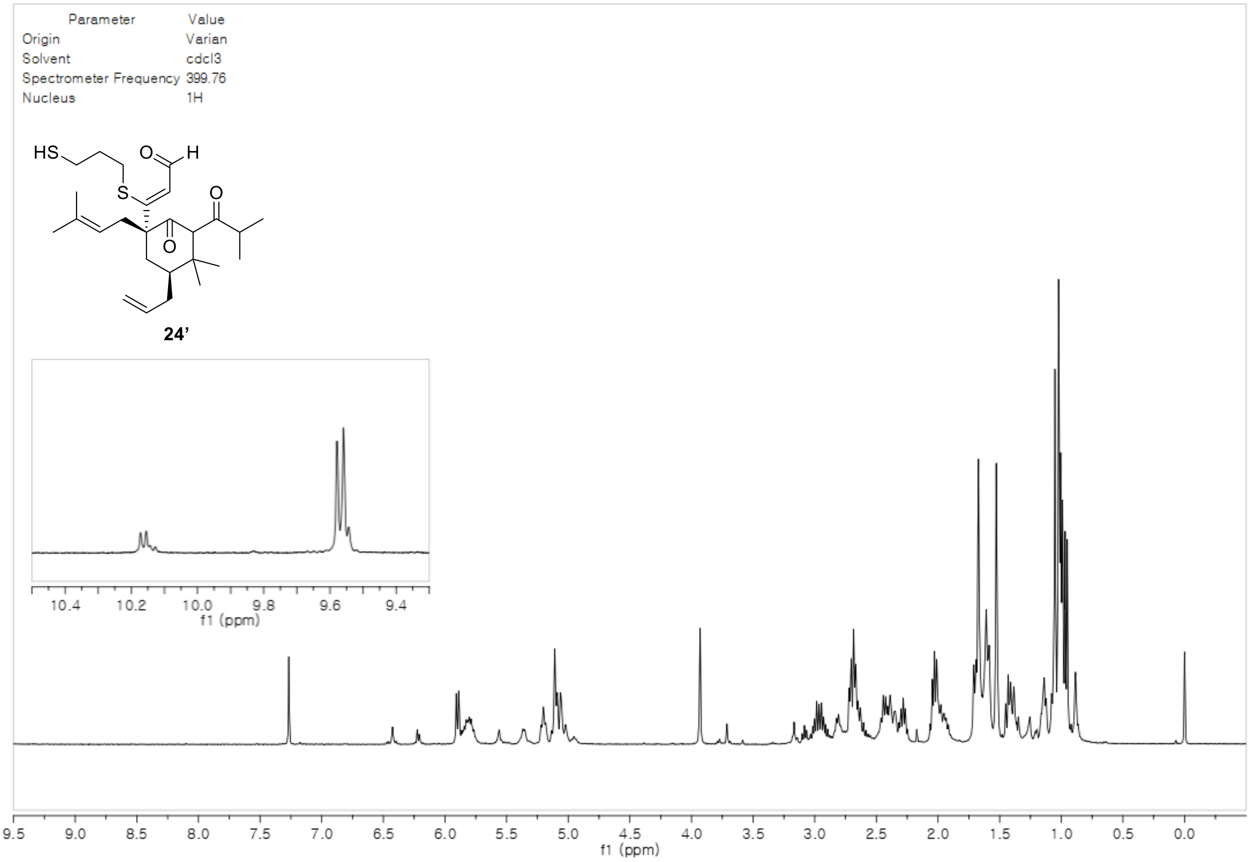

Compound 24' COSY

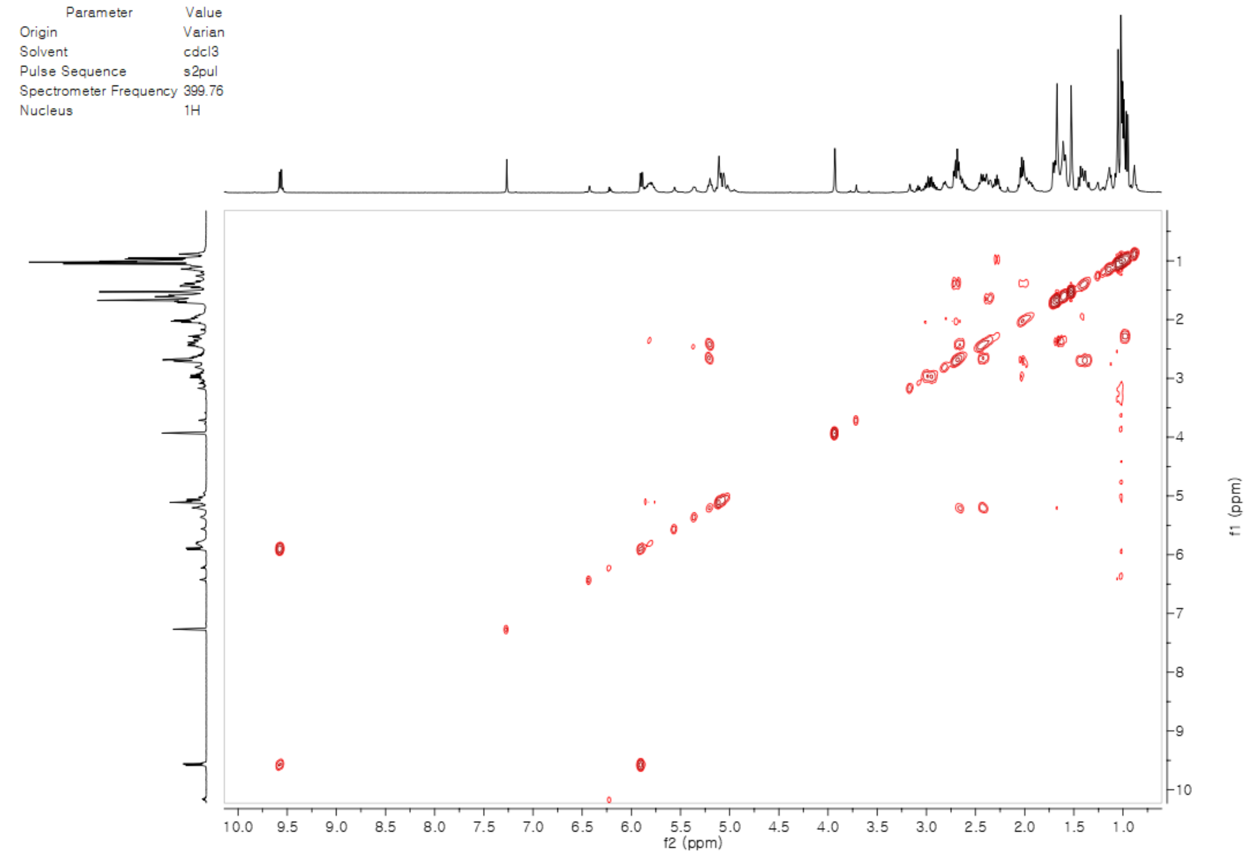

Compound 26 ^{1}H NMR

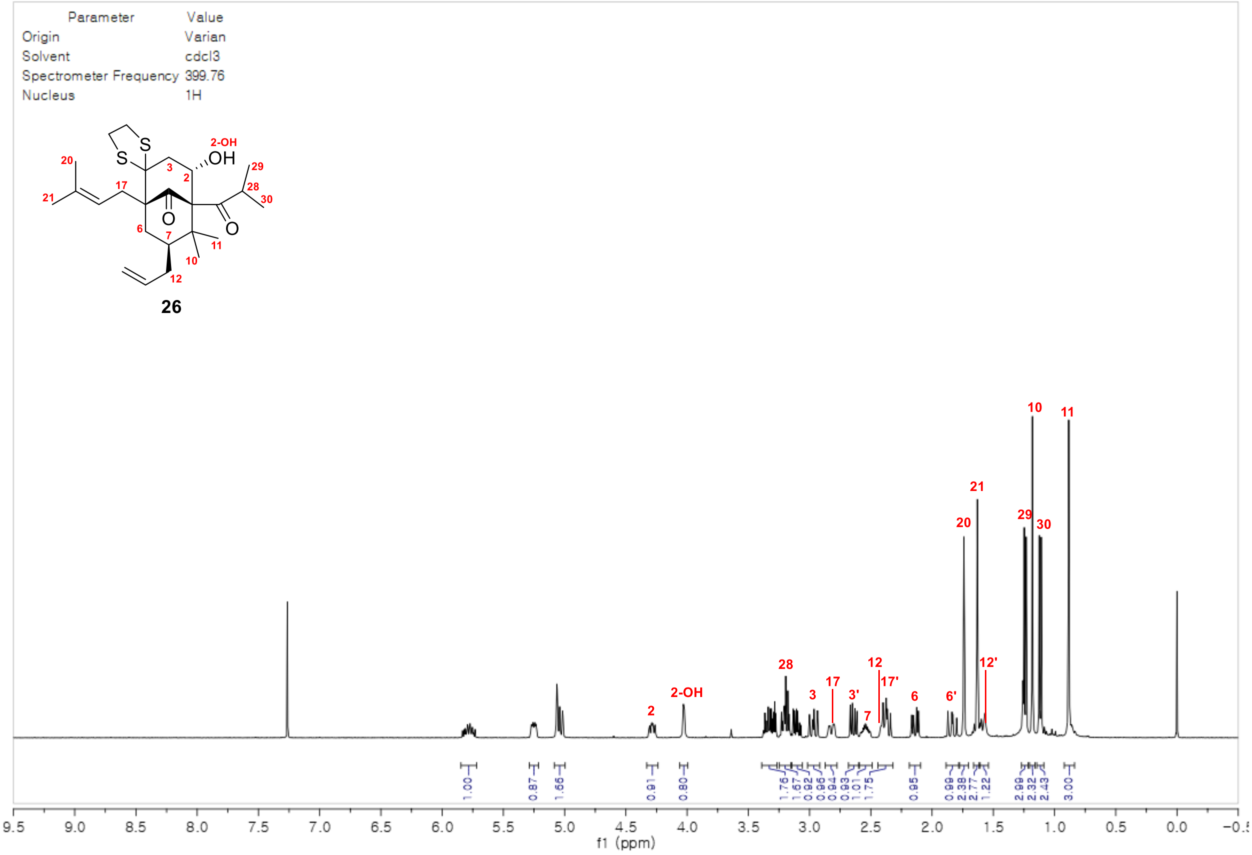

Compound 26 ^{13}C NMR

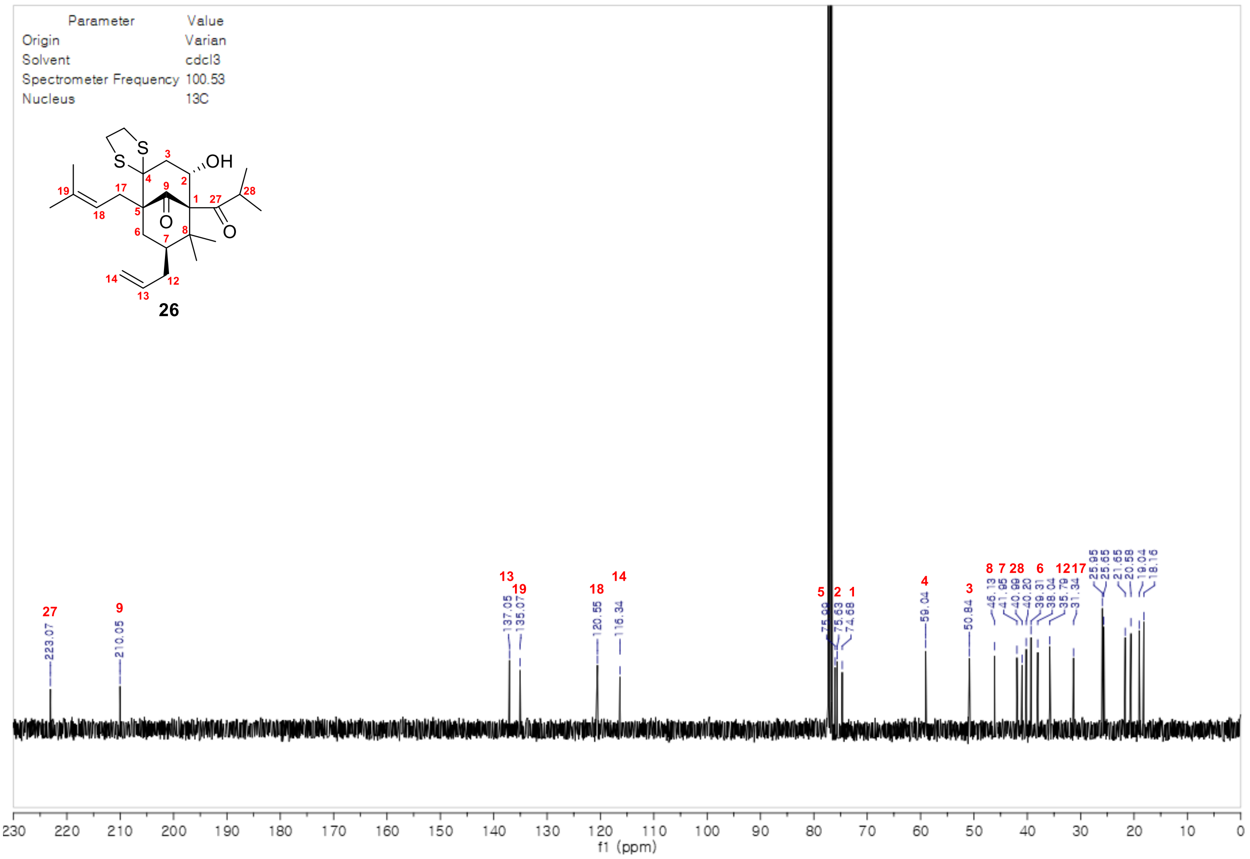

Compound 26 COSY

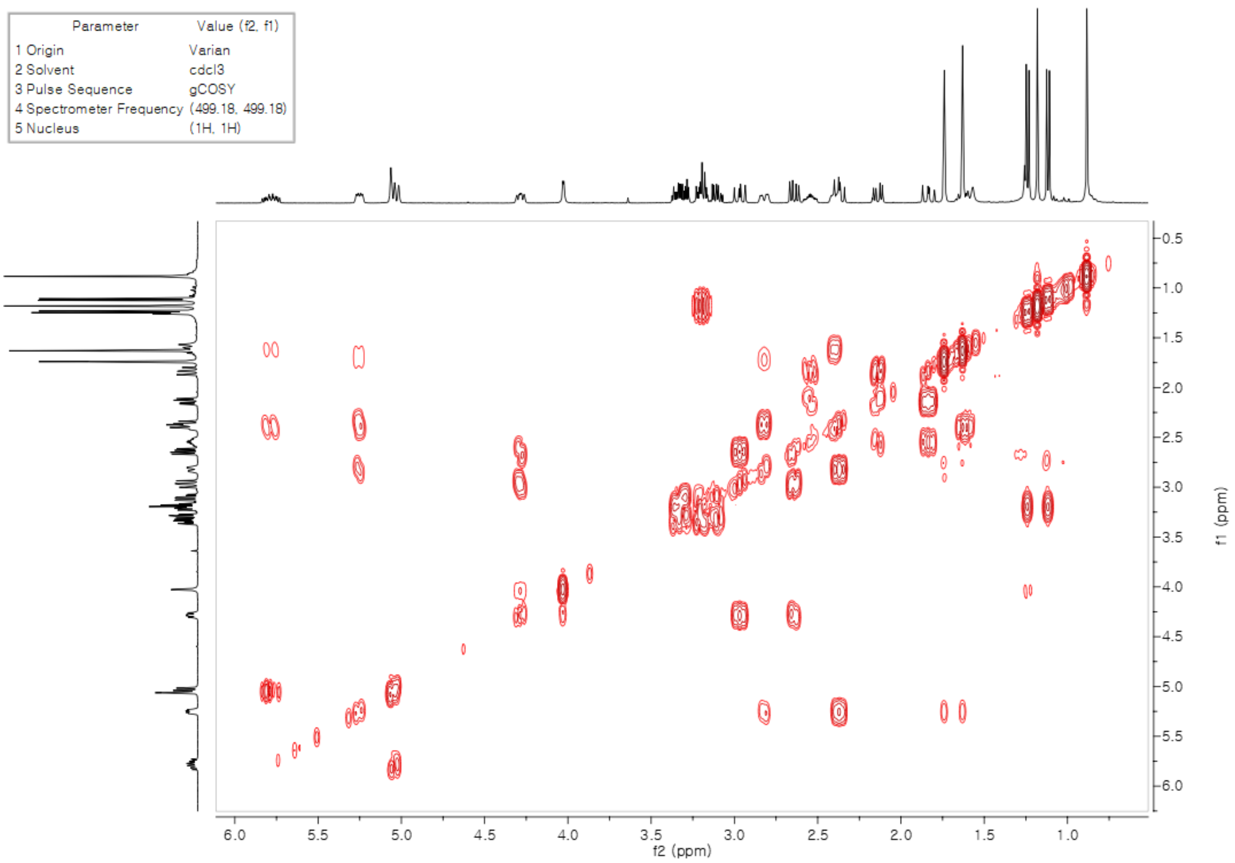

Compound 26 HSQC

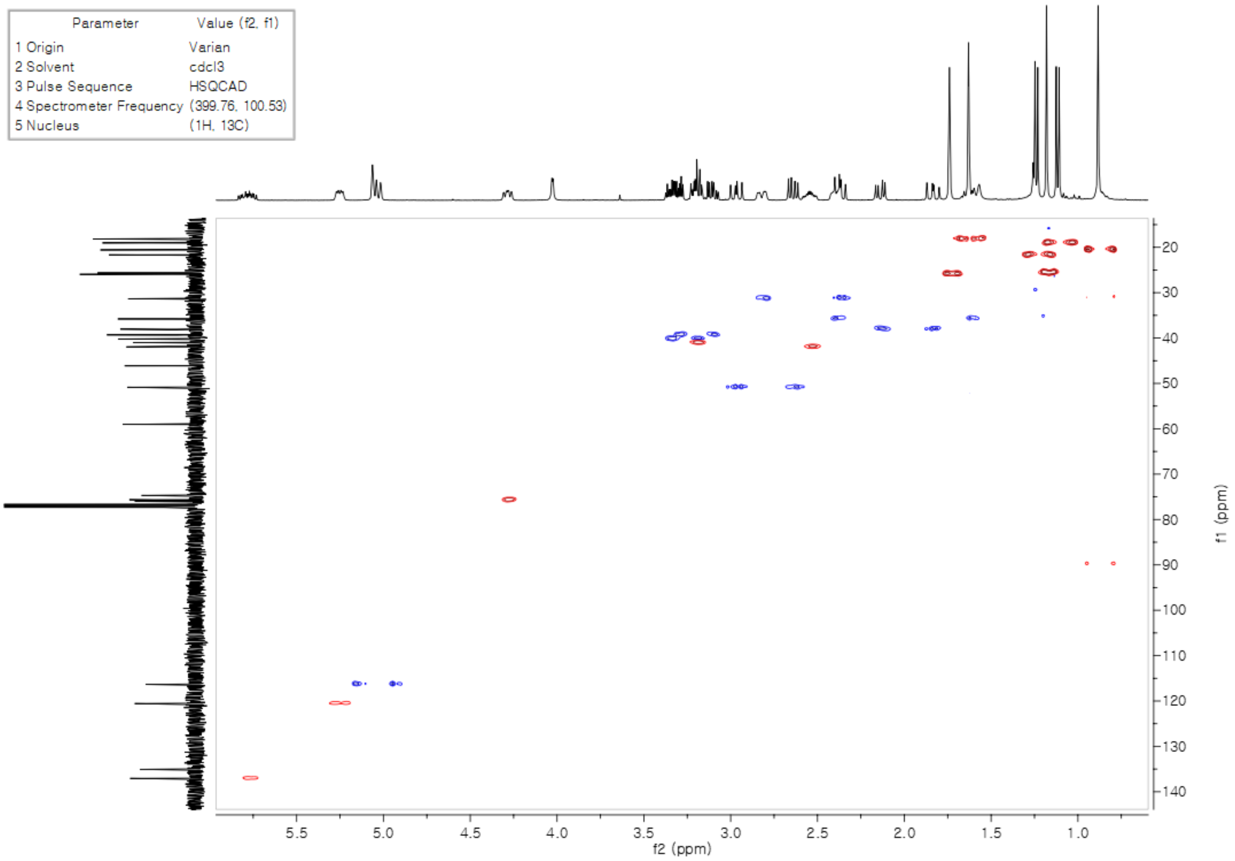

Compound 26 HMBC

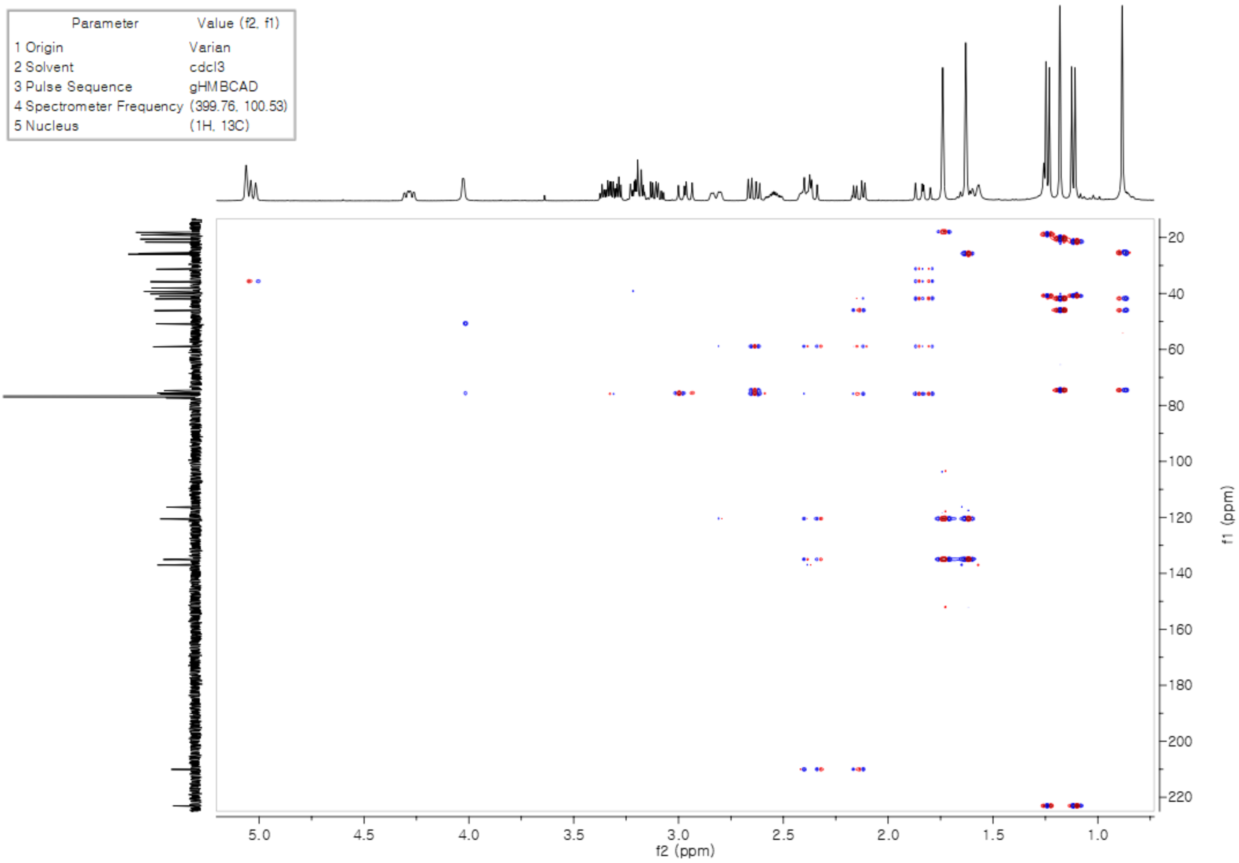

Compound 26 1D NOESY

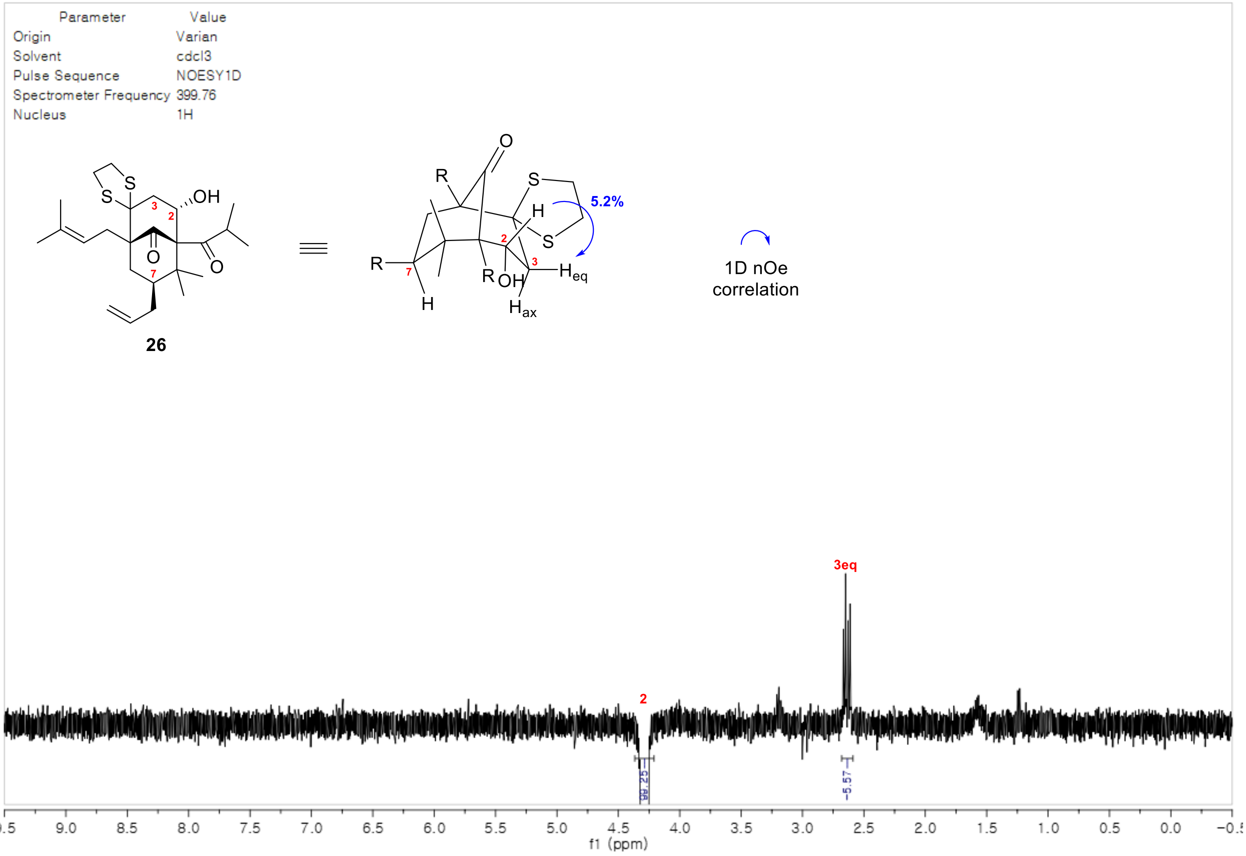

Compound 26 1D NOESY

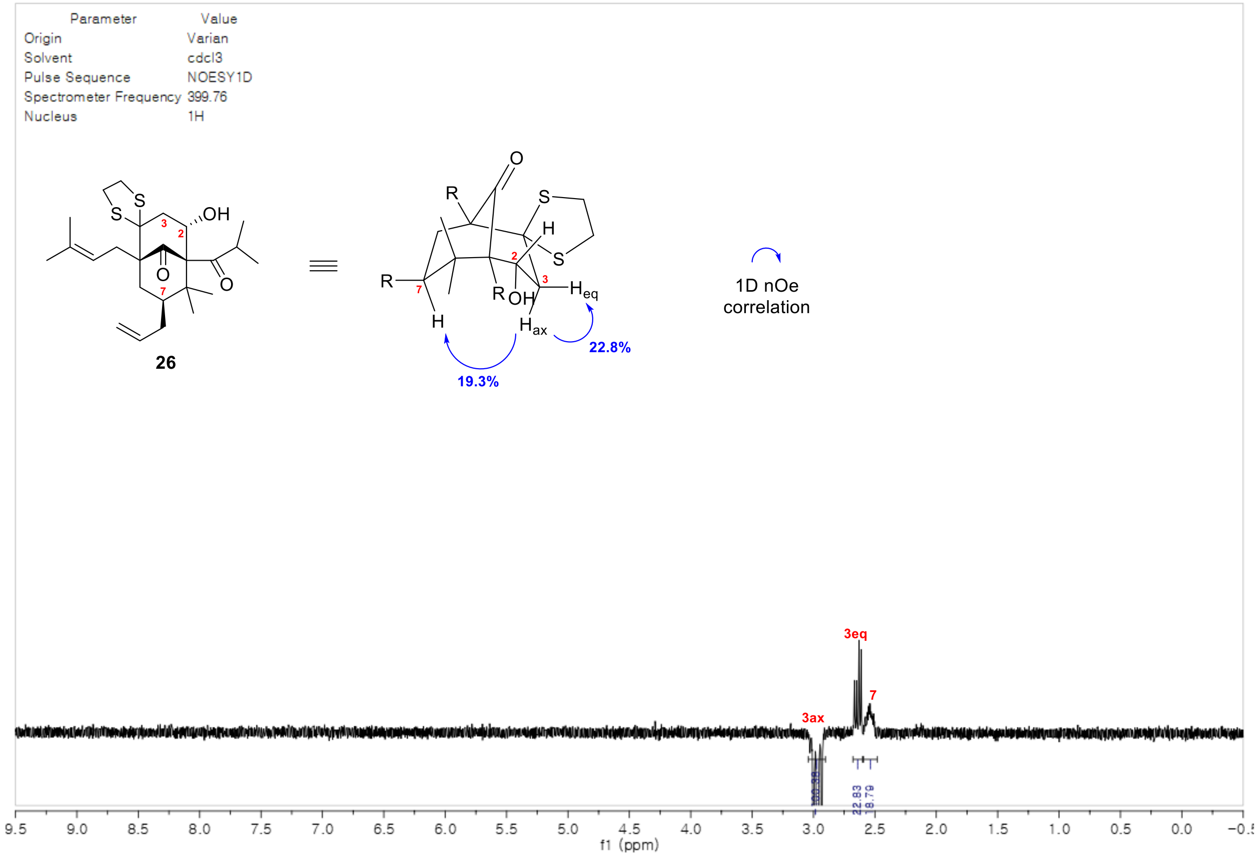

Compound 25 ^{1}H NMR

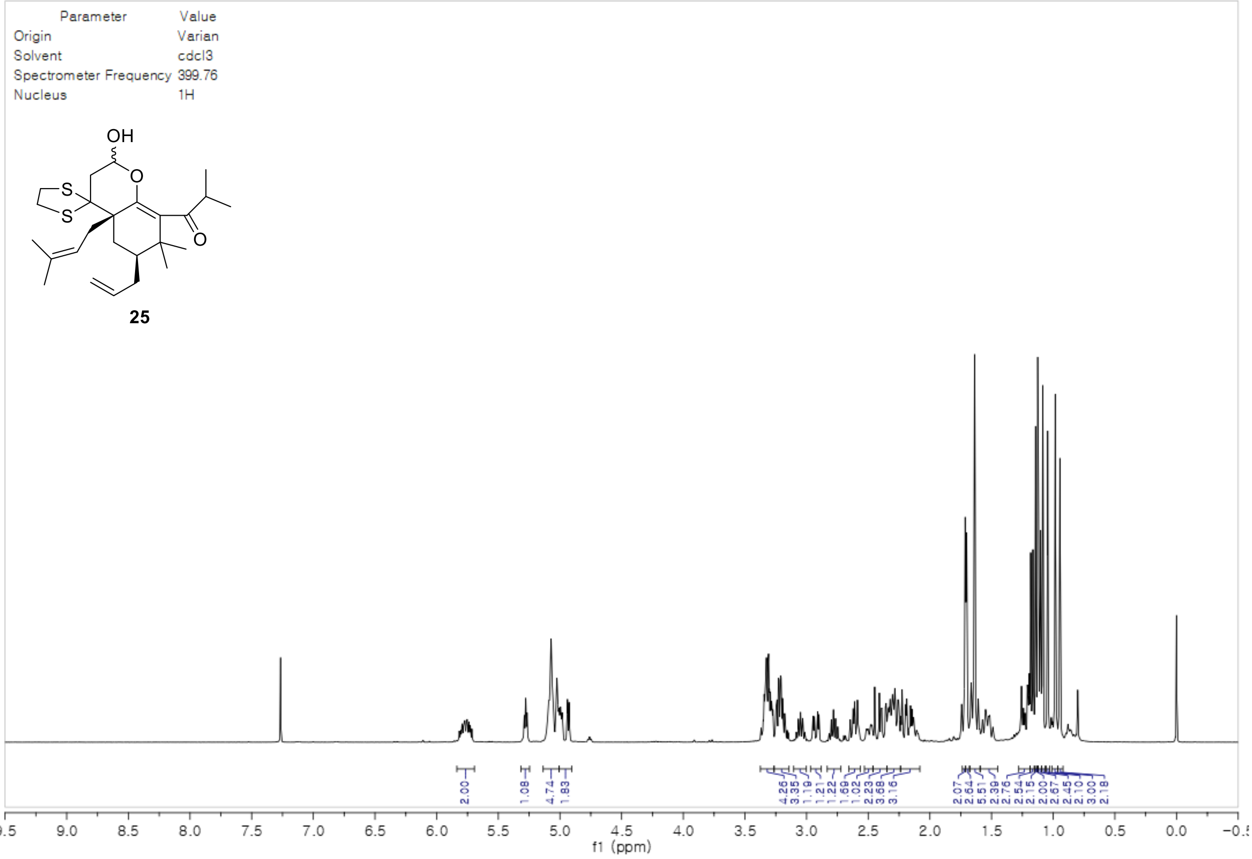

Compound 25 ^{13}C NMR

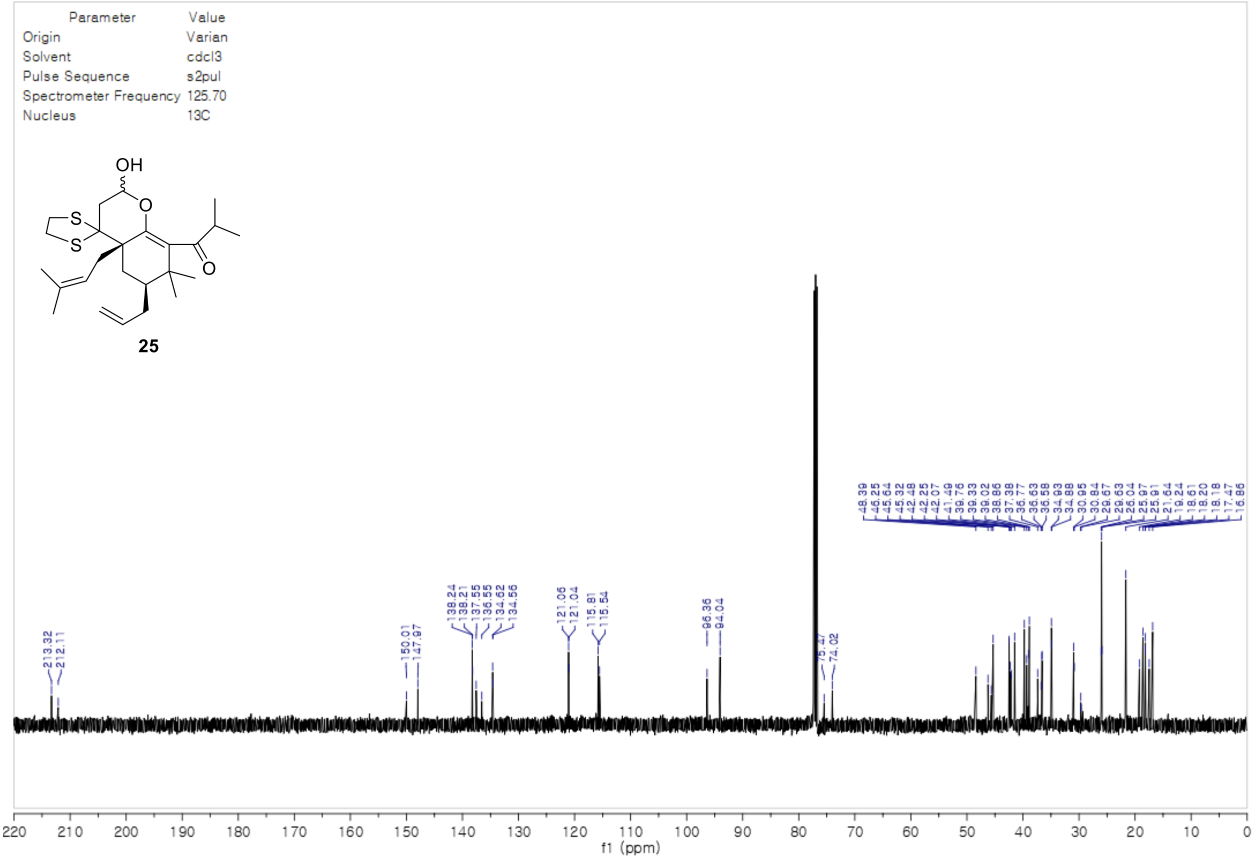

Compound 27 ^{1}H NMR

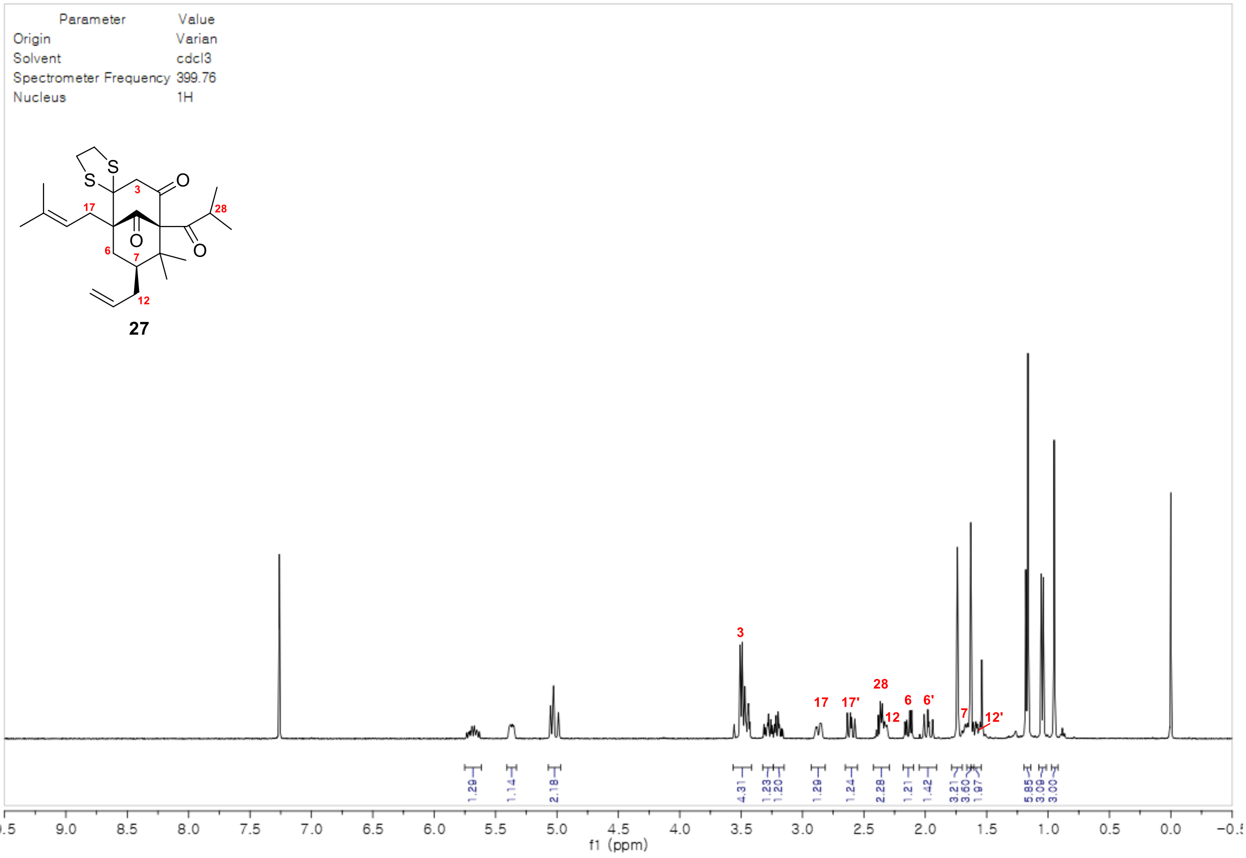

Compound 27 ^{13}C NMR

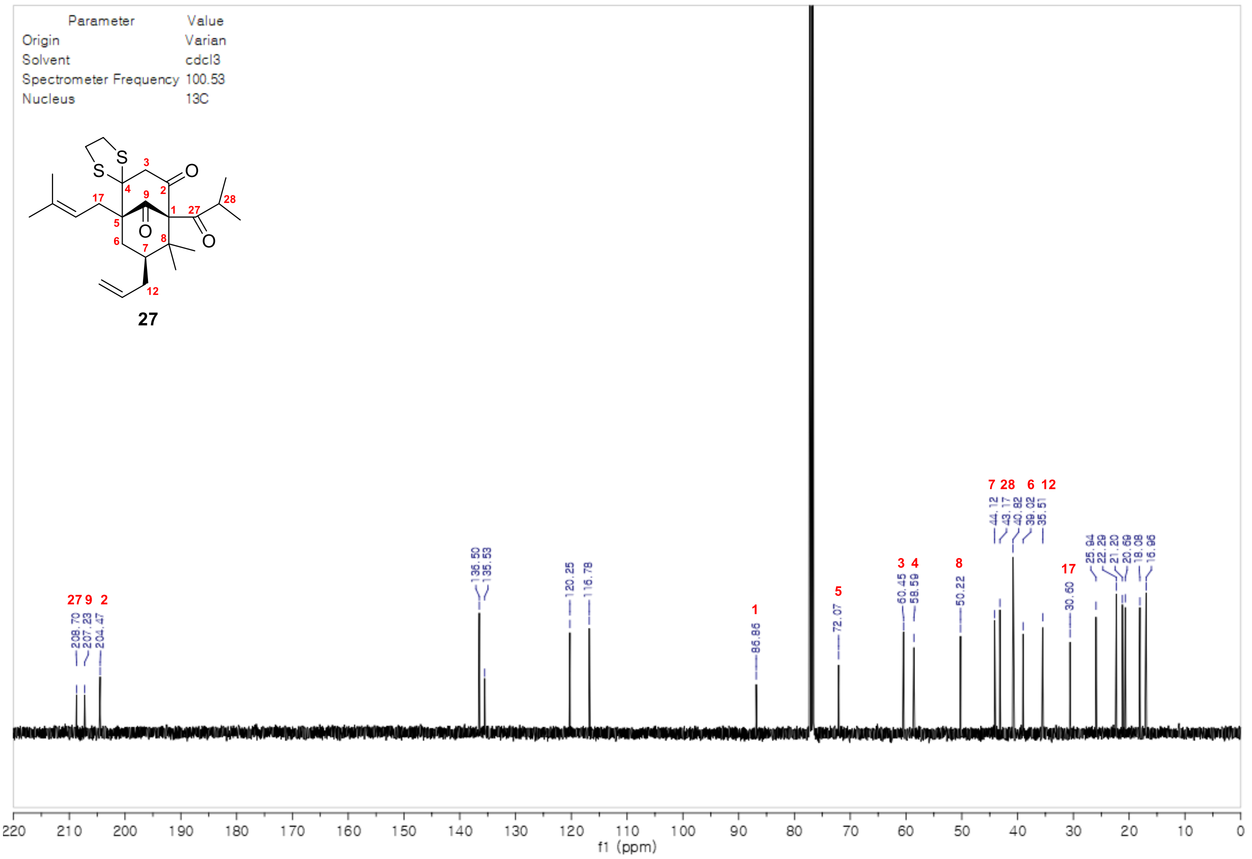

Compound 27 COSY

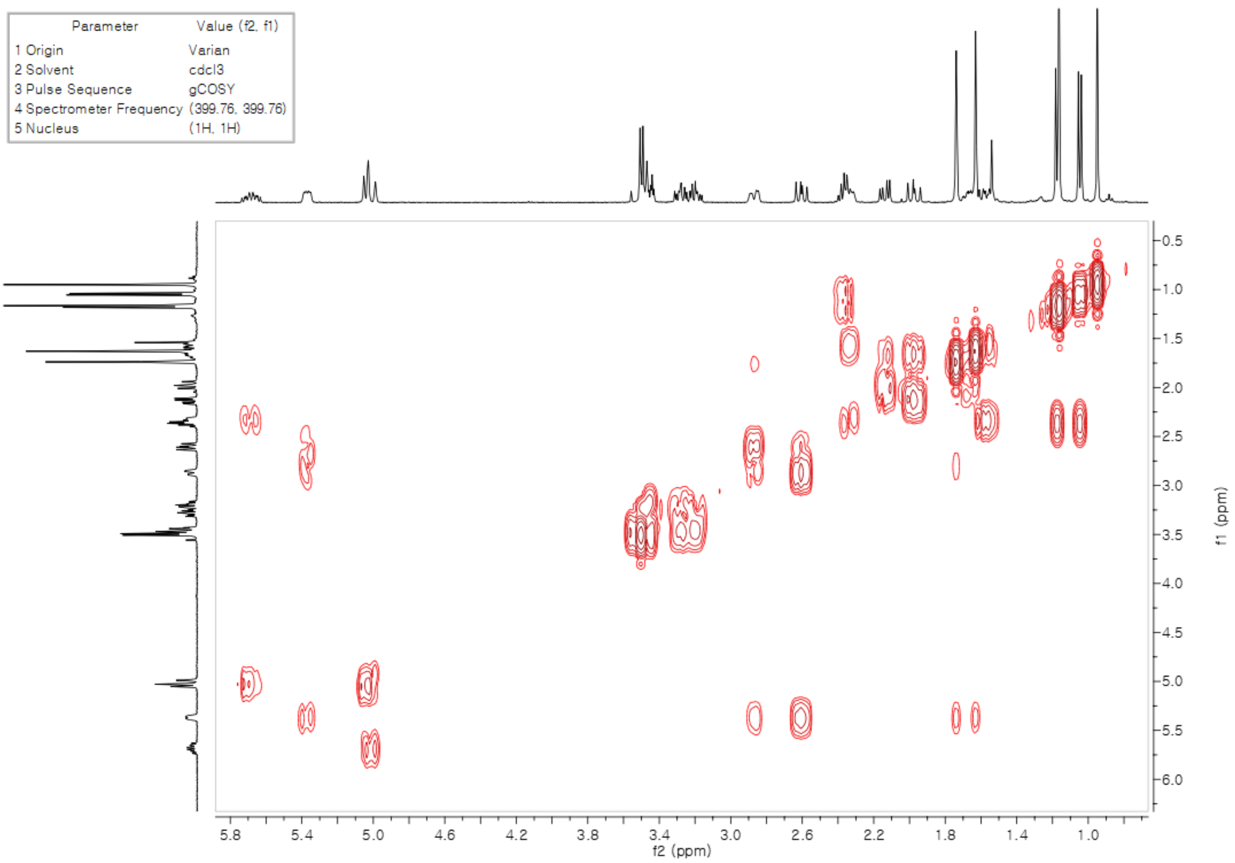

Compound 27 HSQC

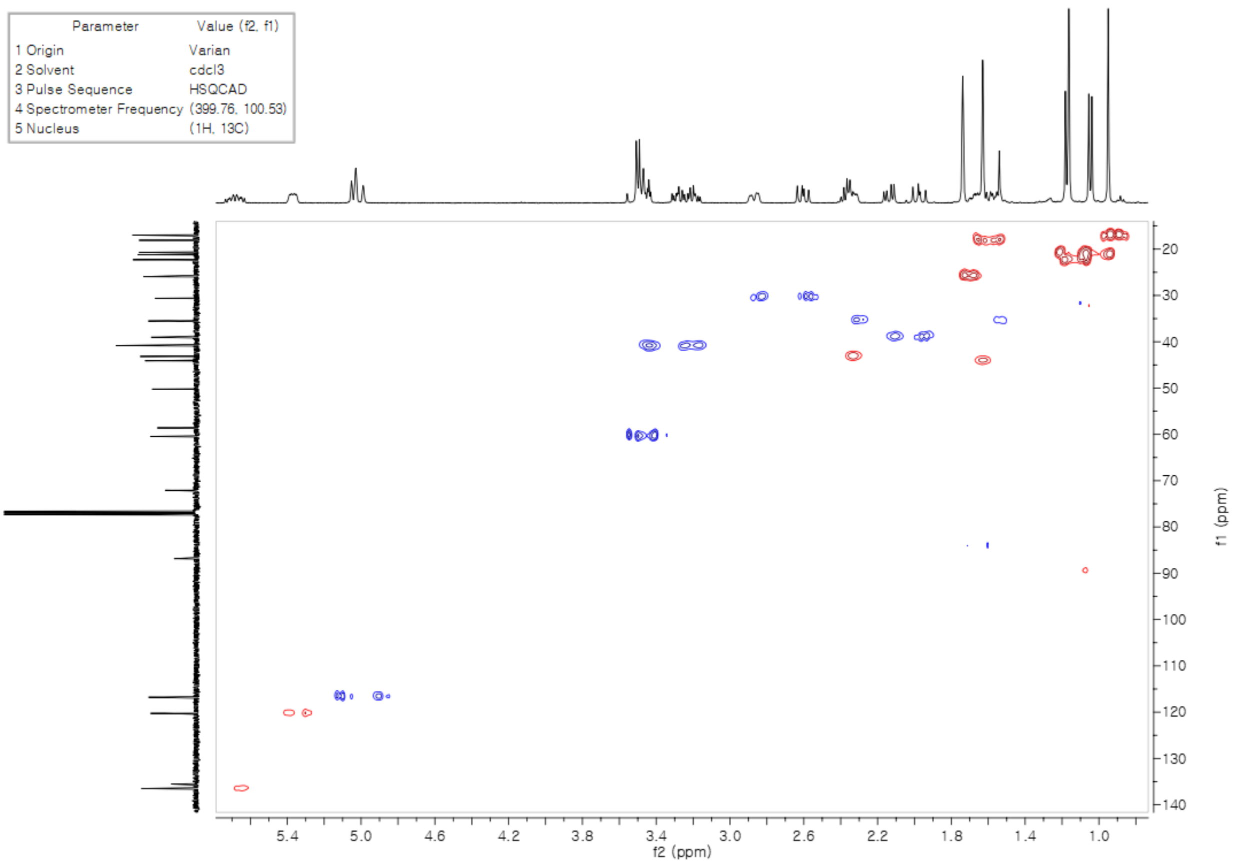

Compound 27 HMBC

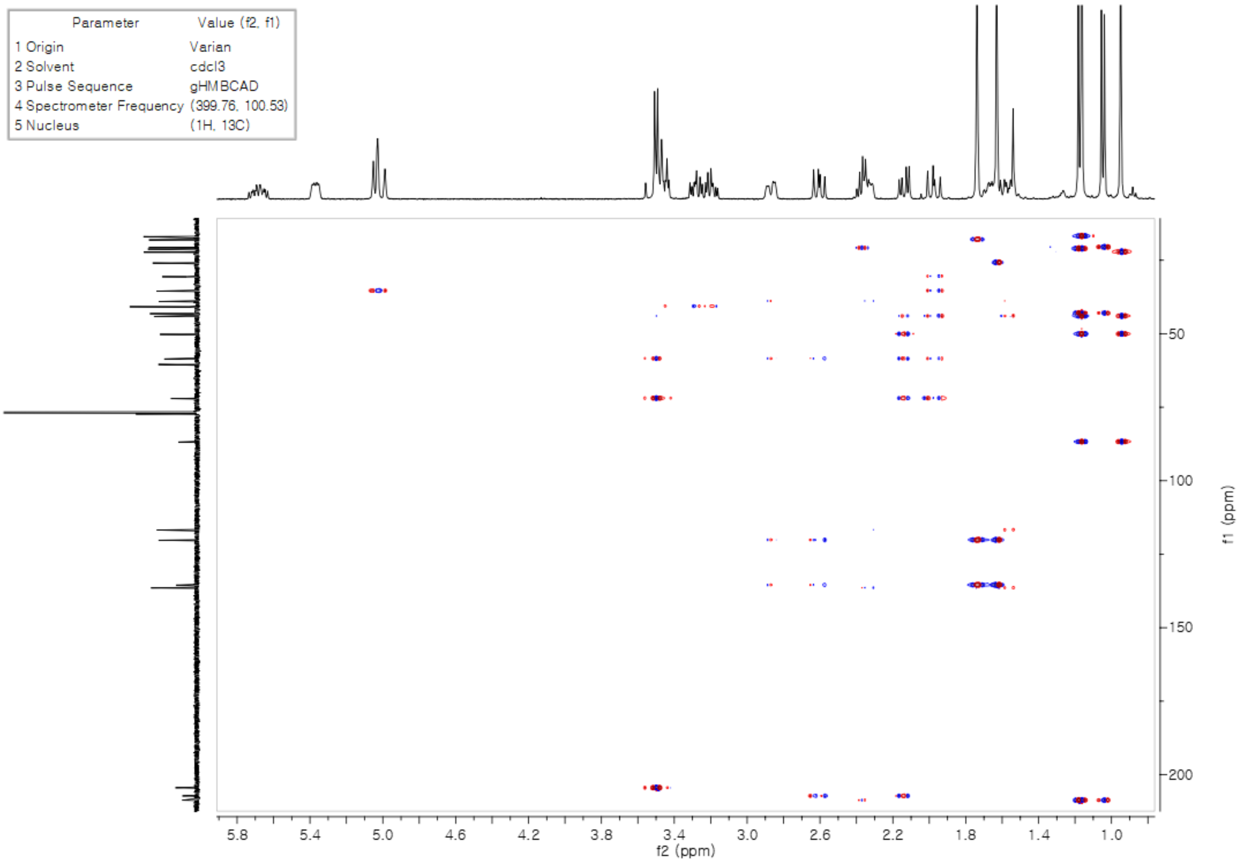

Compound 25' ^{1}H NMR

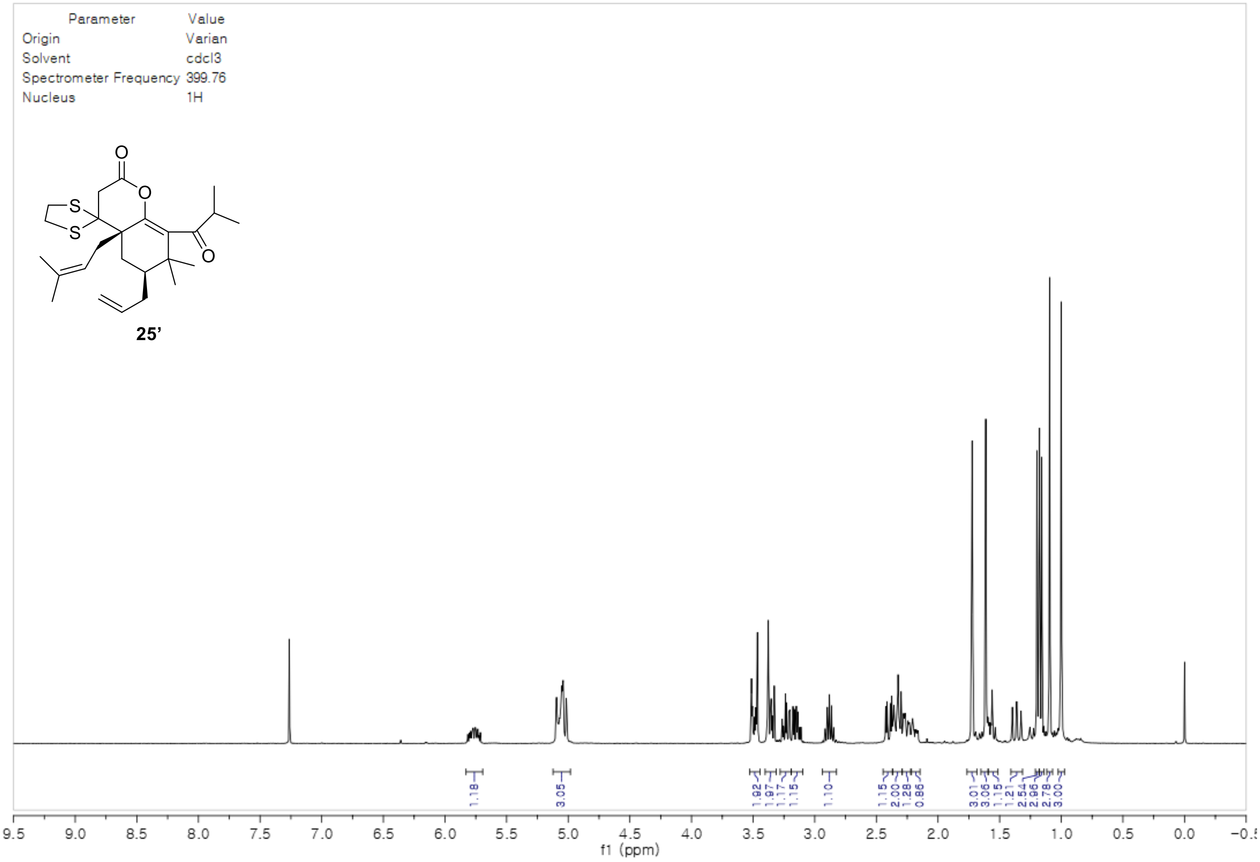

Compound 25' ^{13}C NMR

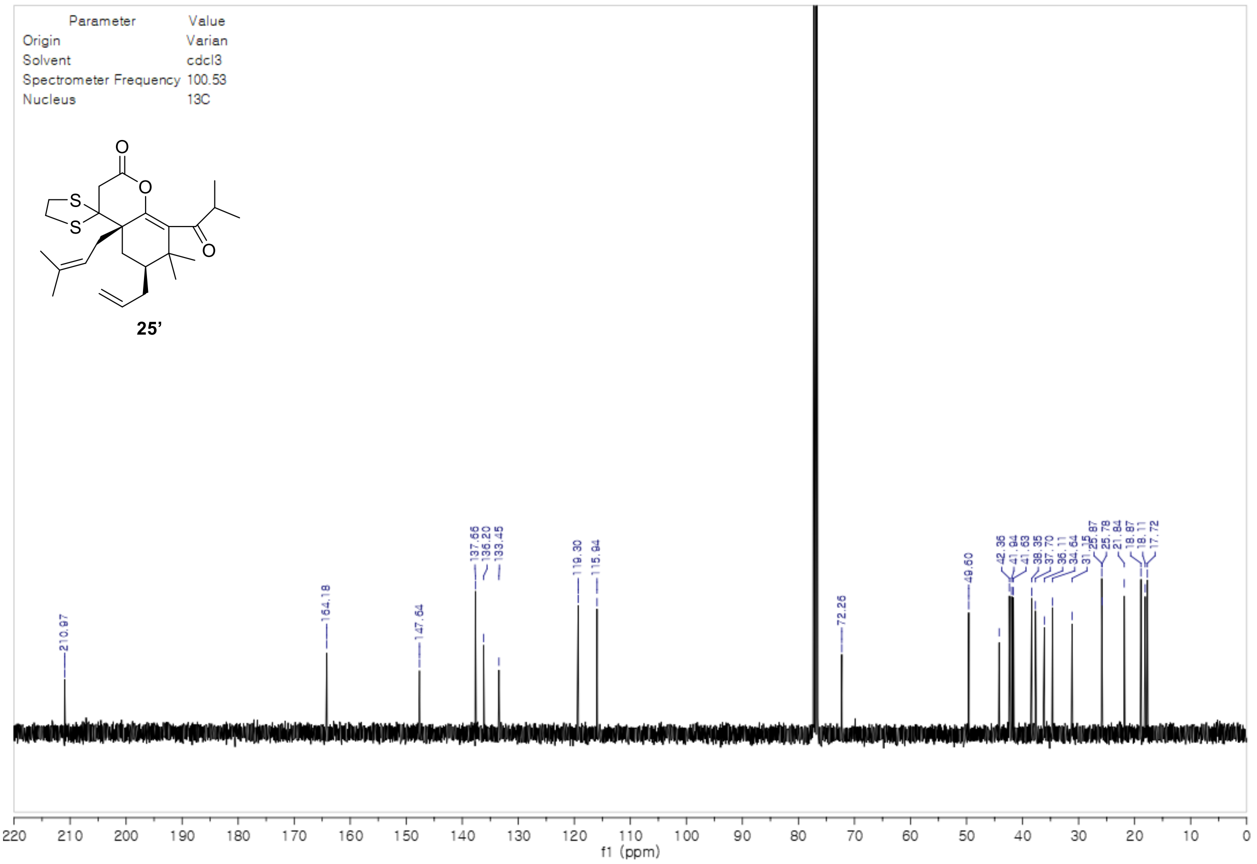

Compound 30 ^{1}H NMR

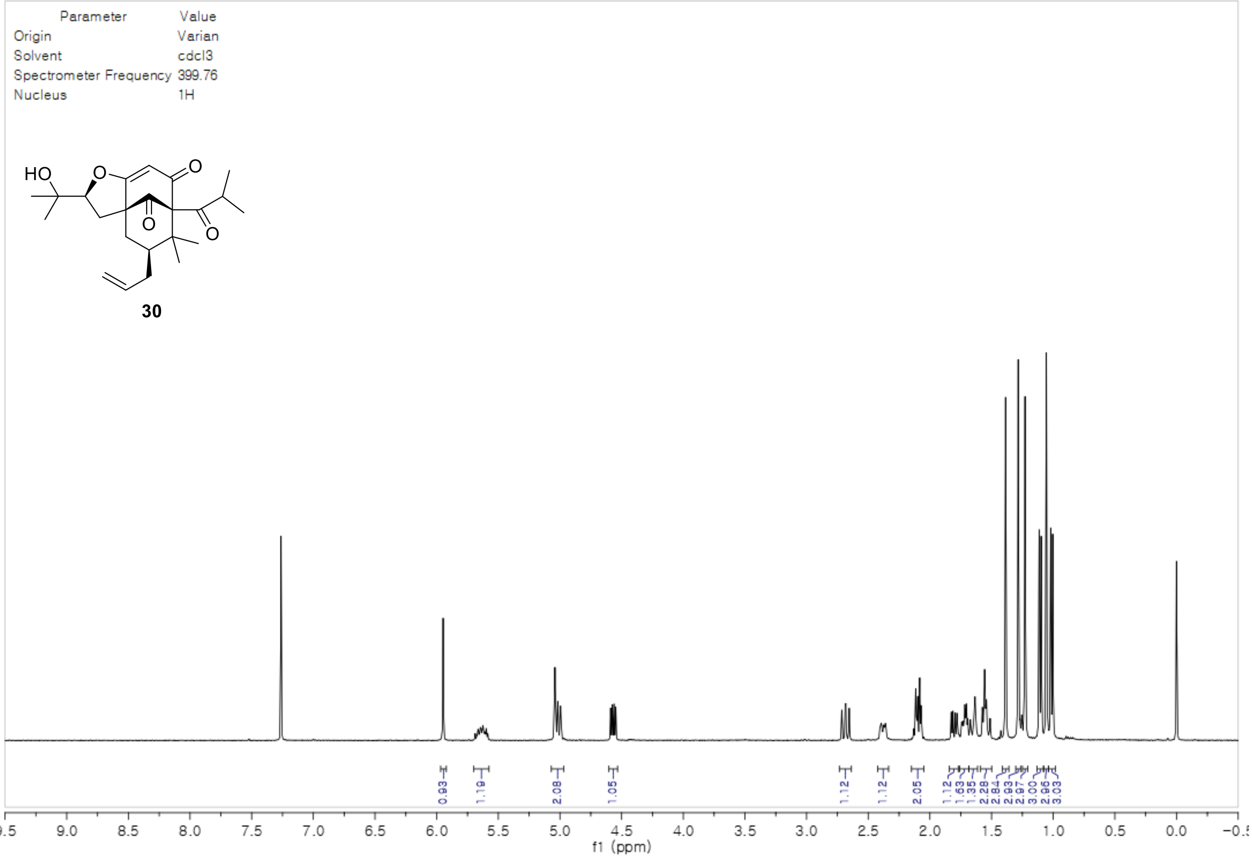

Compound 30 ^{13}C NMR

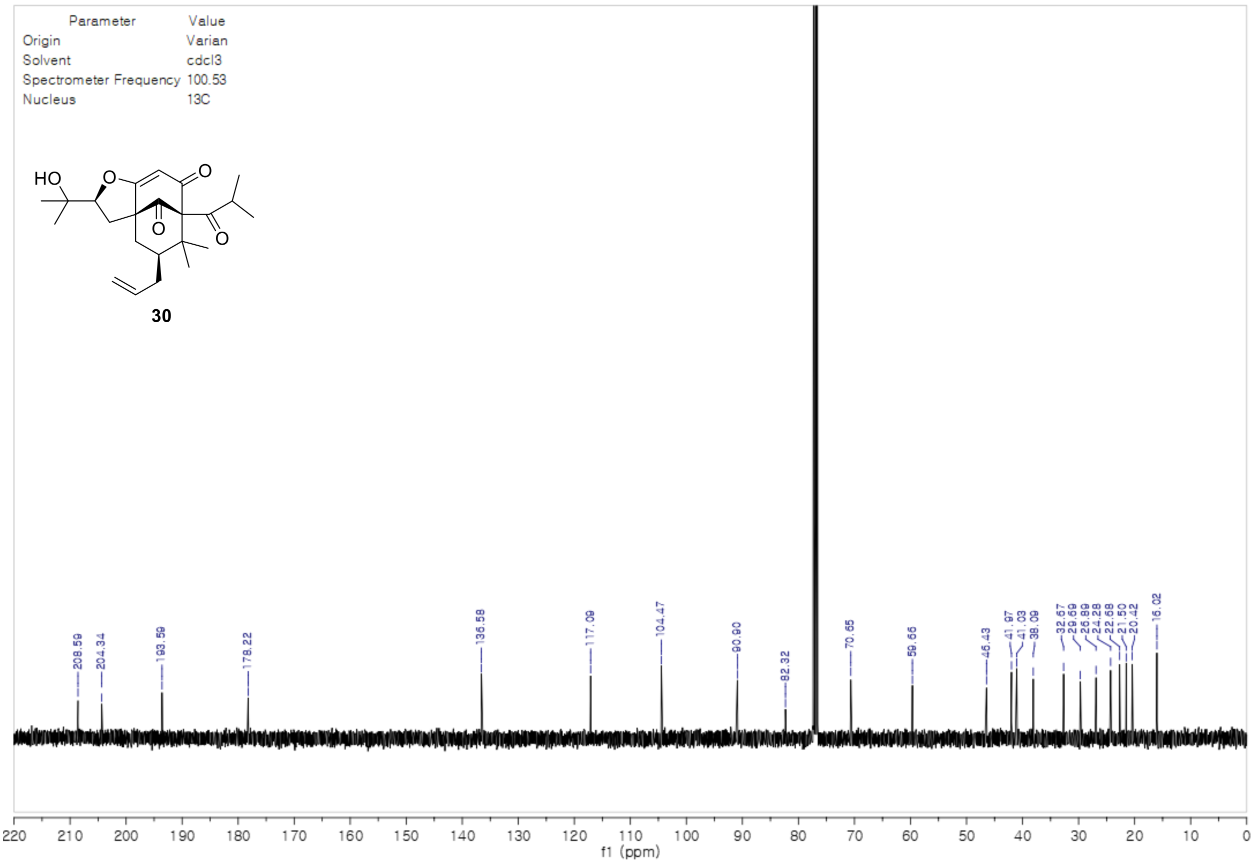

Compound 30 COSY

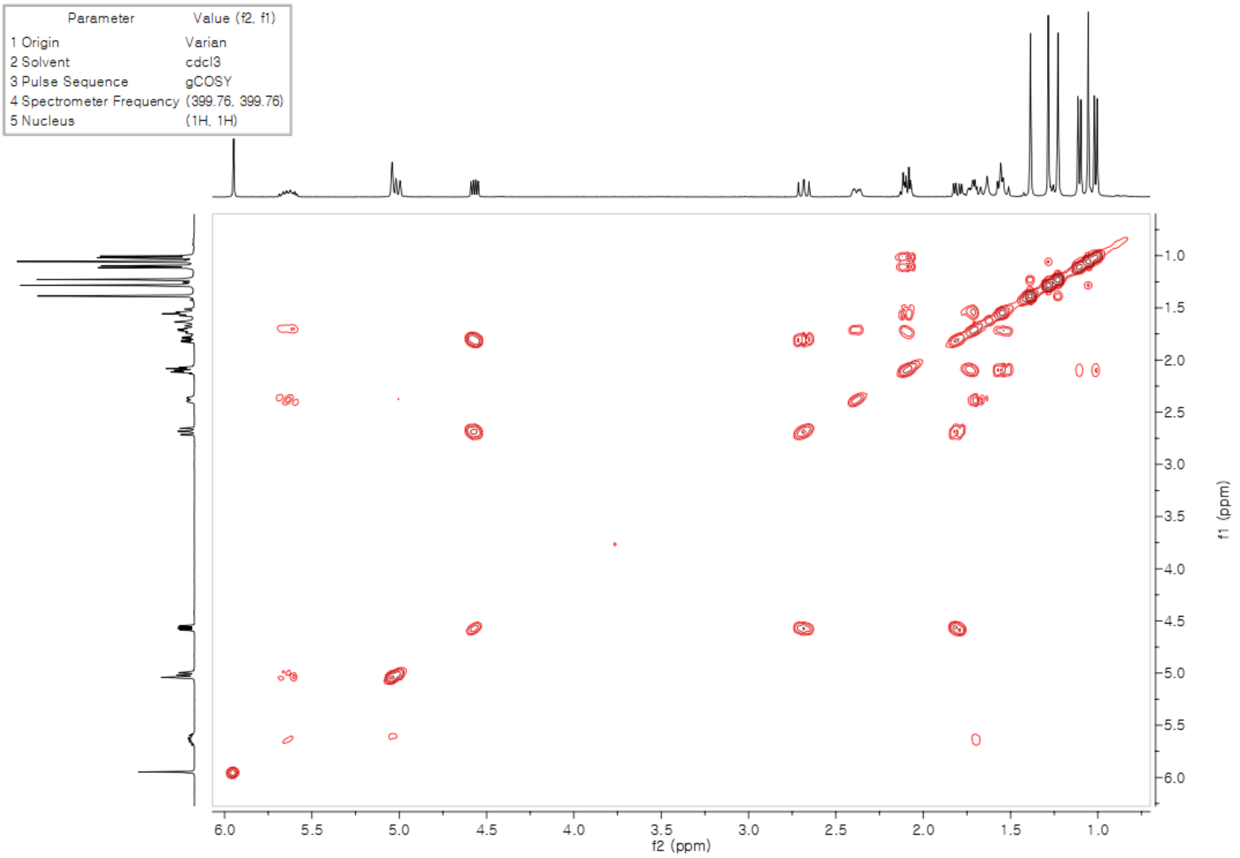

Compound *epi*-30 ^{1}H NMR

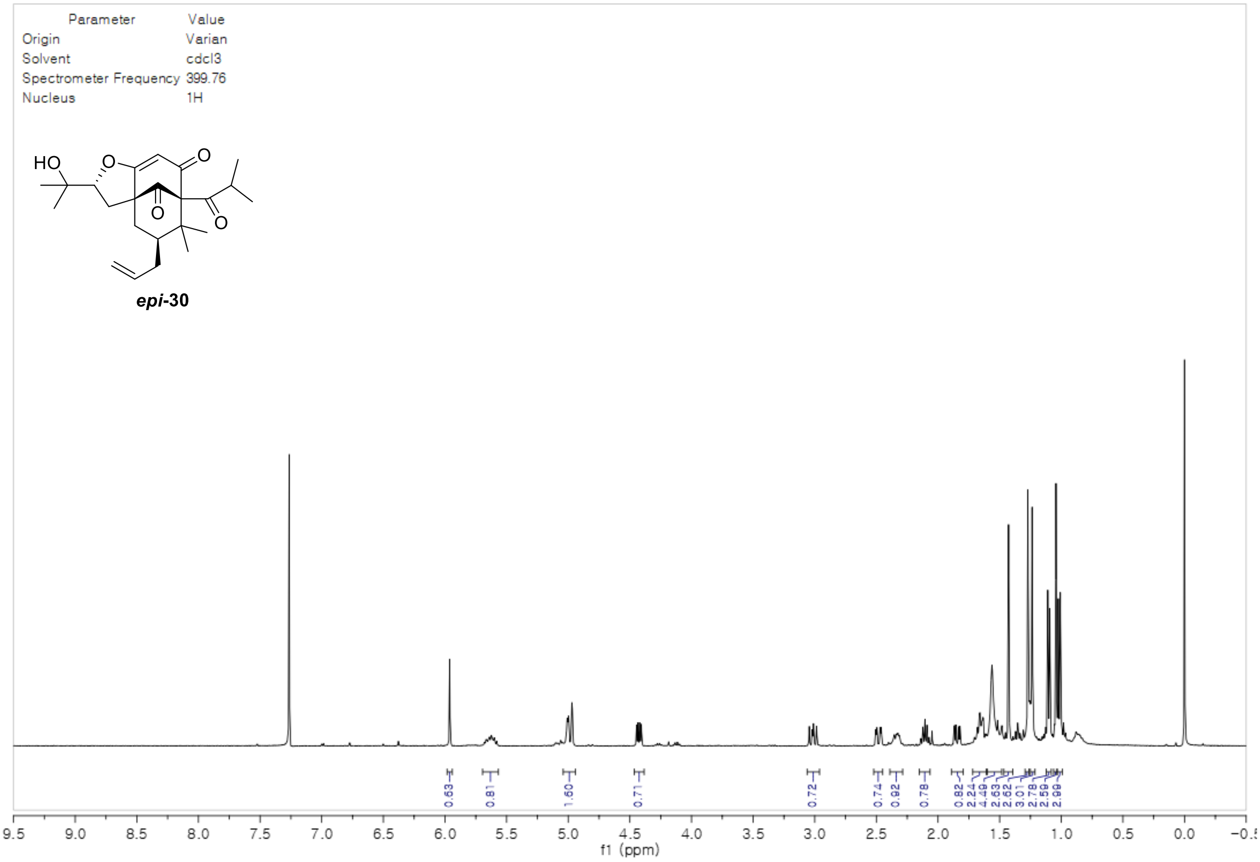

Compound *epi*-30 ^{13}C NMR

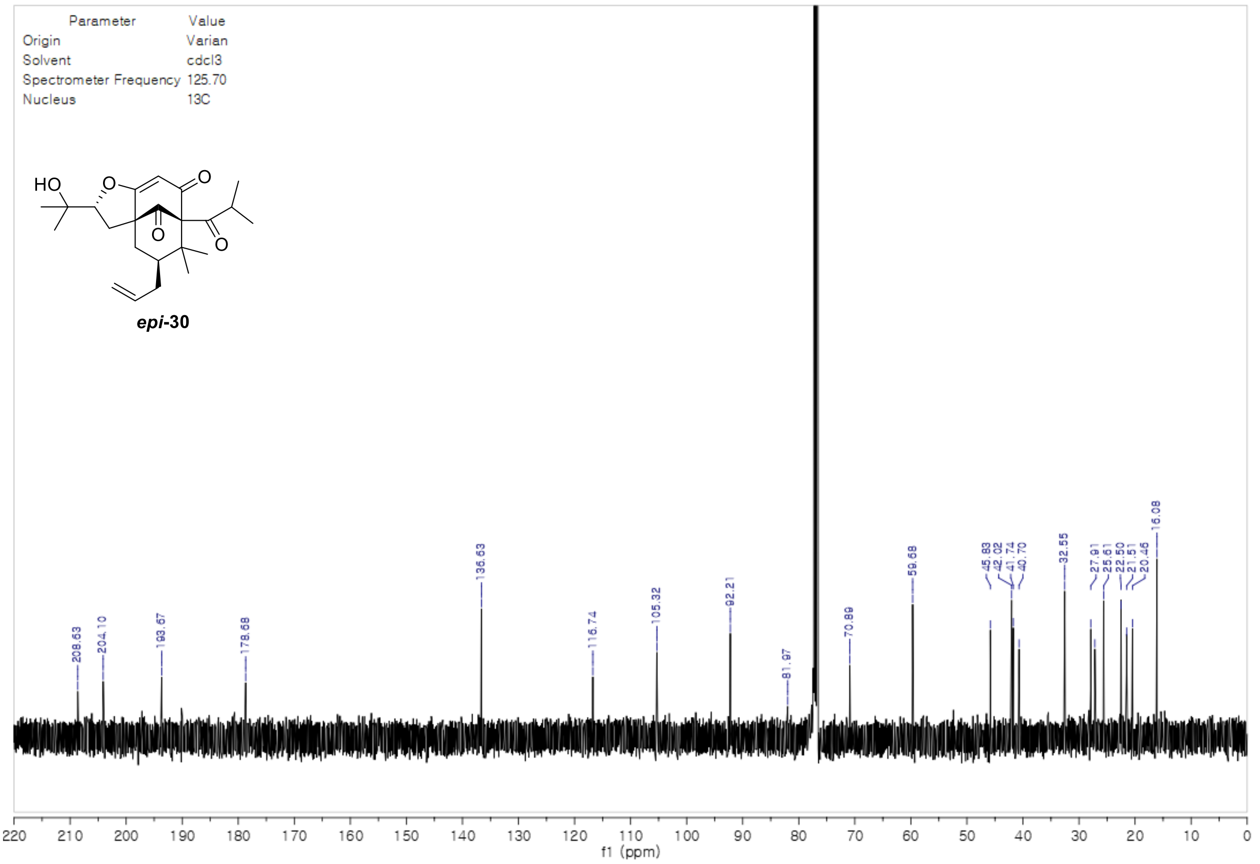

Compound 30' ^1H NMR

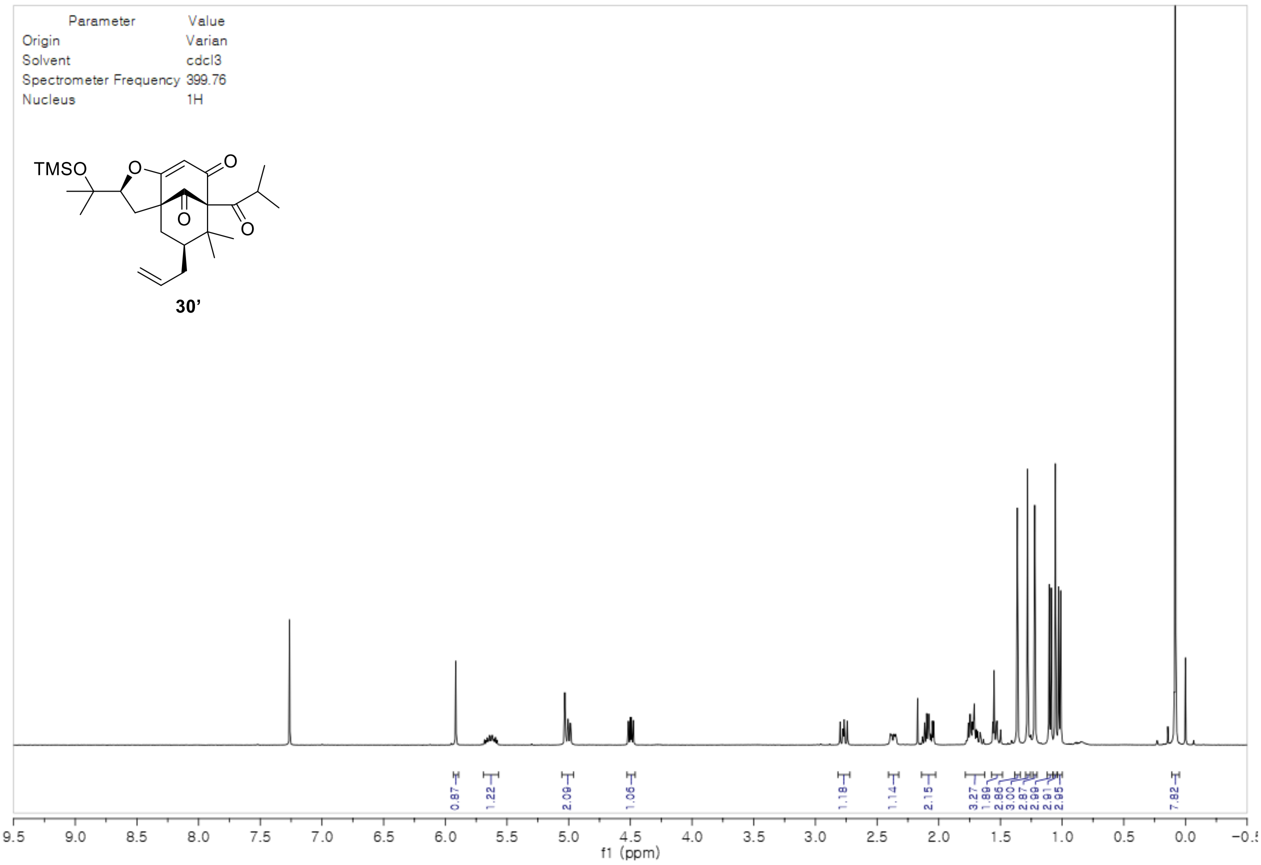

Compound 30' ^{13}C NMR

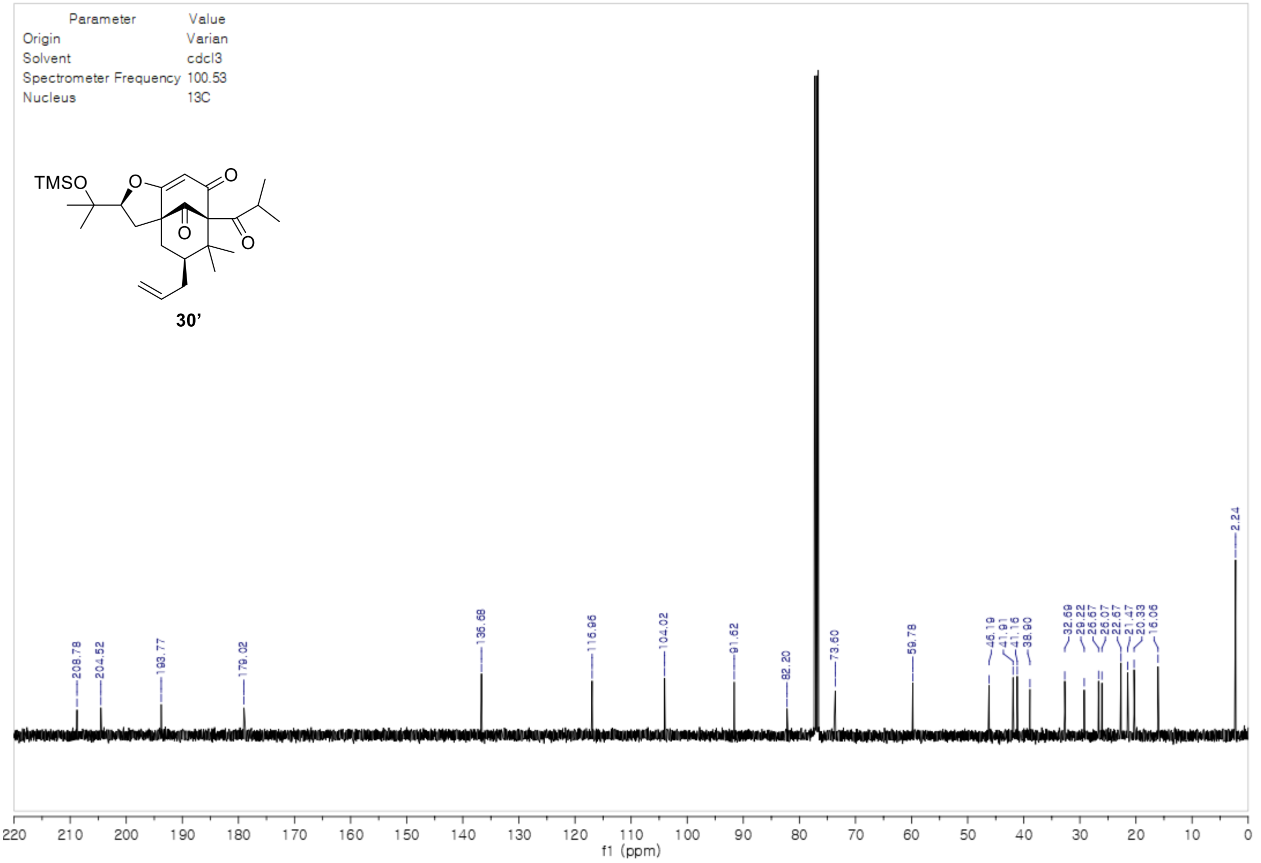

Compound 31' ¹H NMR

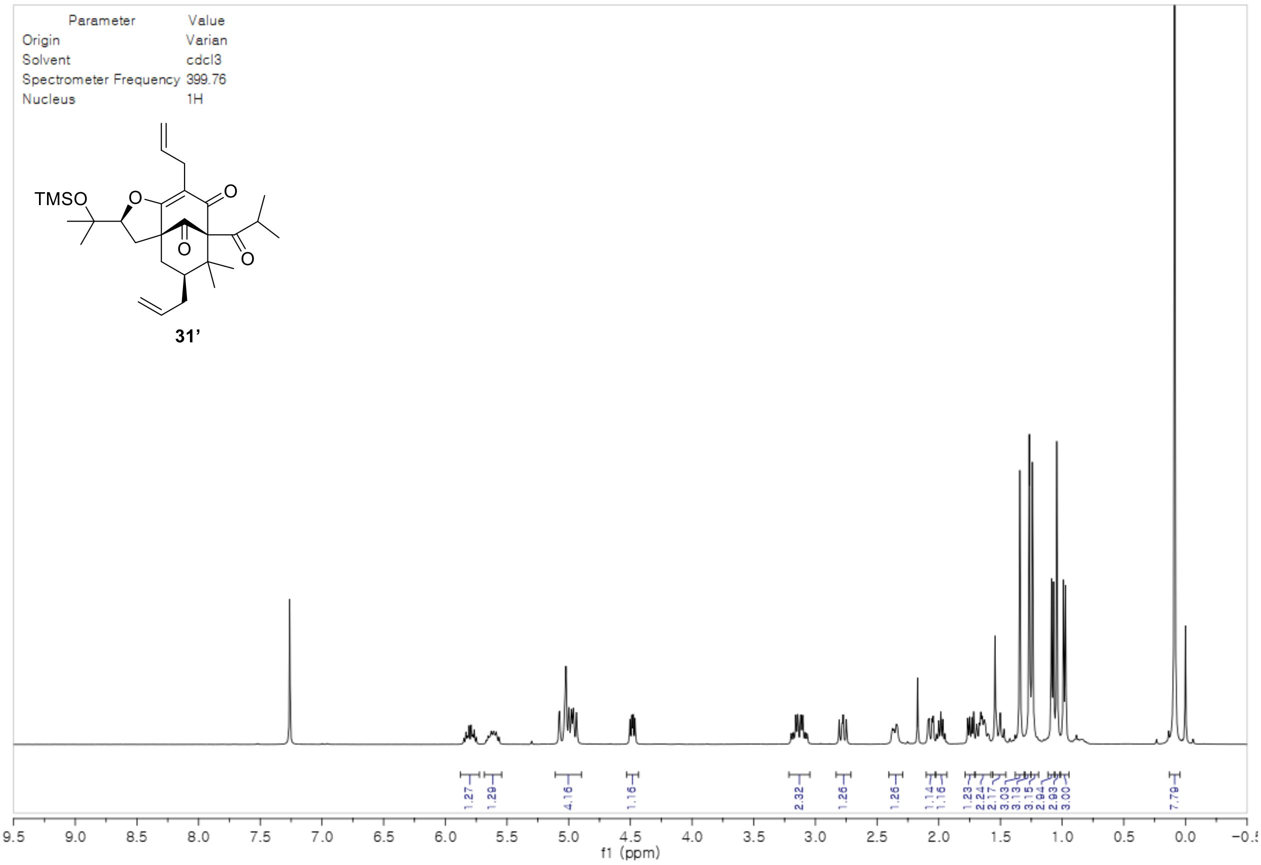

Compound 31' ¹³C NMR

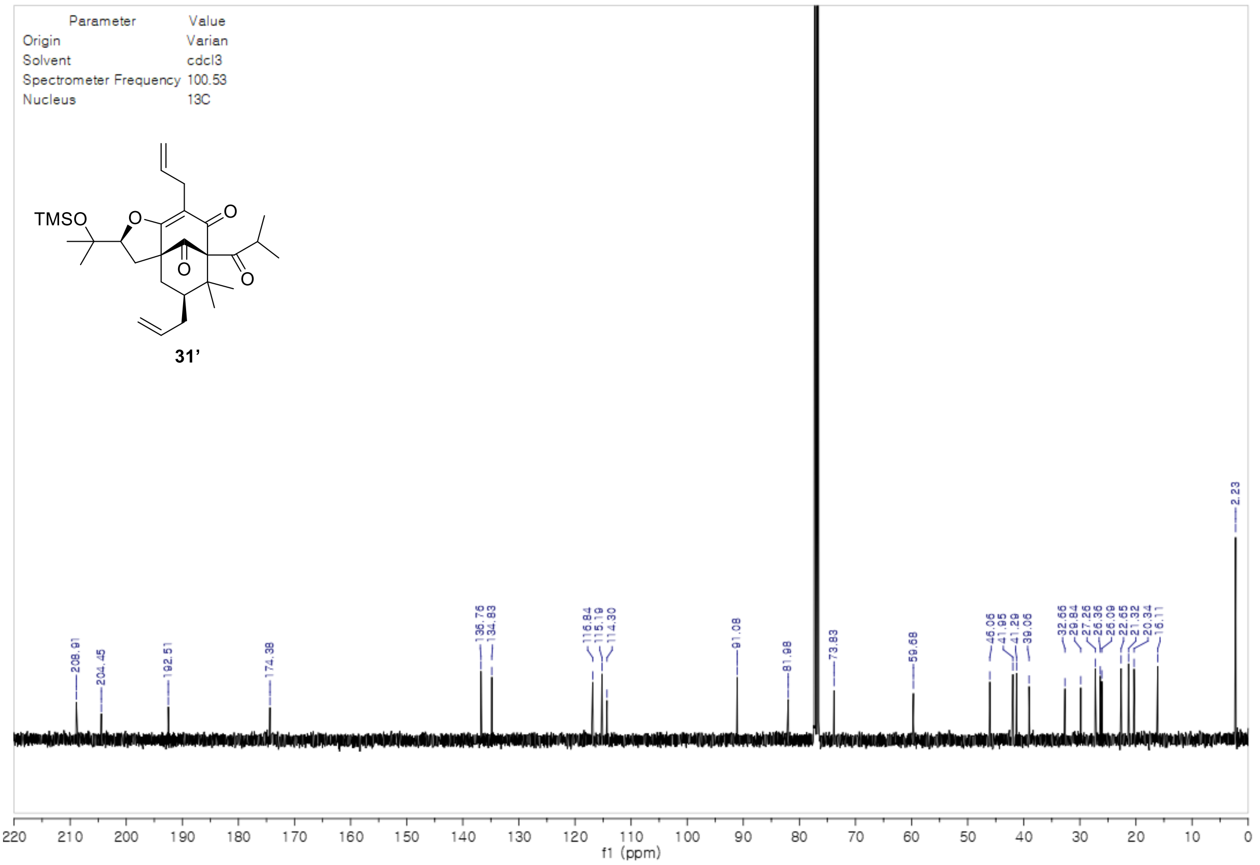

Compound 31 ^{1}H NMR

Parameter	Value
Origin	Varian
Solvent	cdcl3
Spectrometer Frequency	399.76
Nucleus	1H

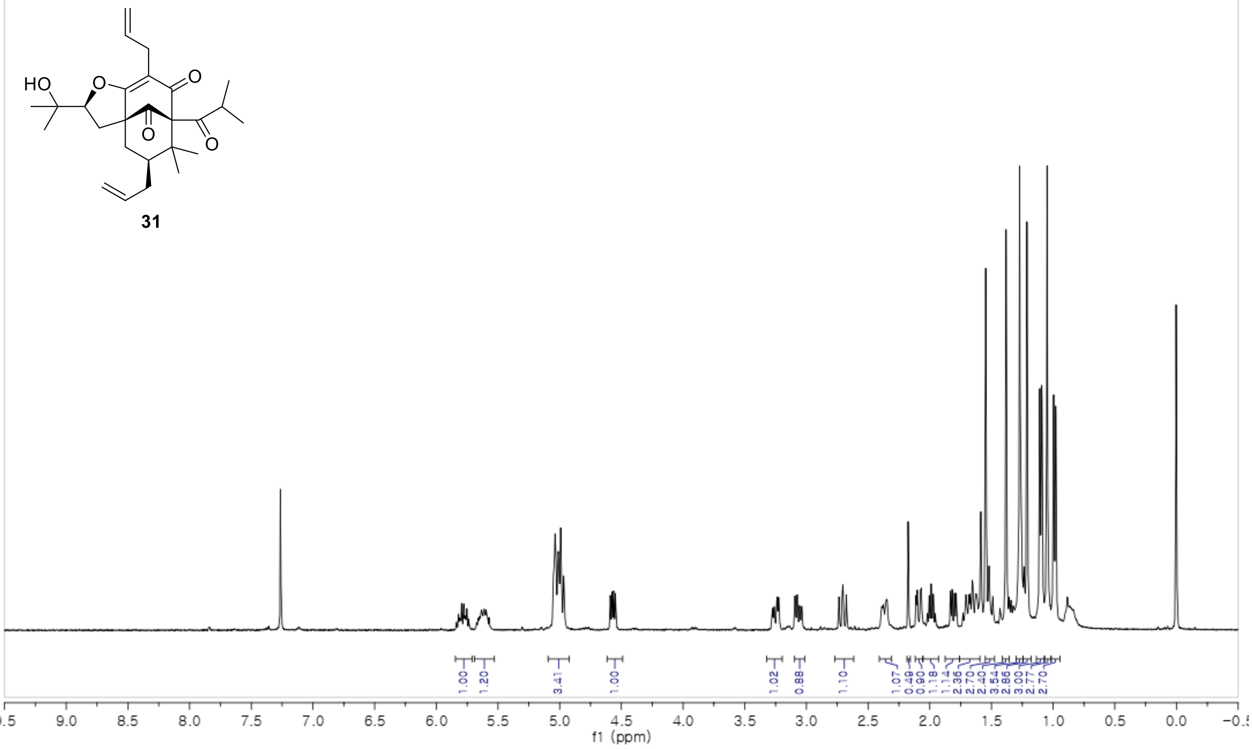

Compound 31 ^{13}C NMR

Parameter	Value
Origin	Varian
Solvent	cdcl3
Spectrometer Frequency	100.53
Nucleus	13C

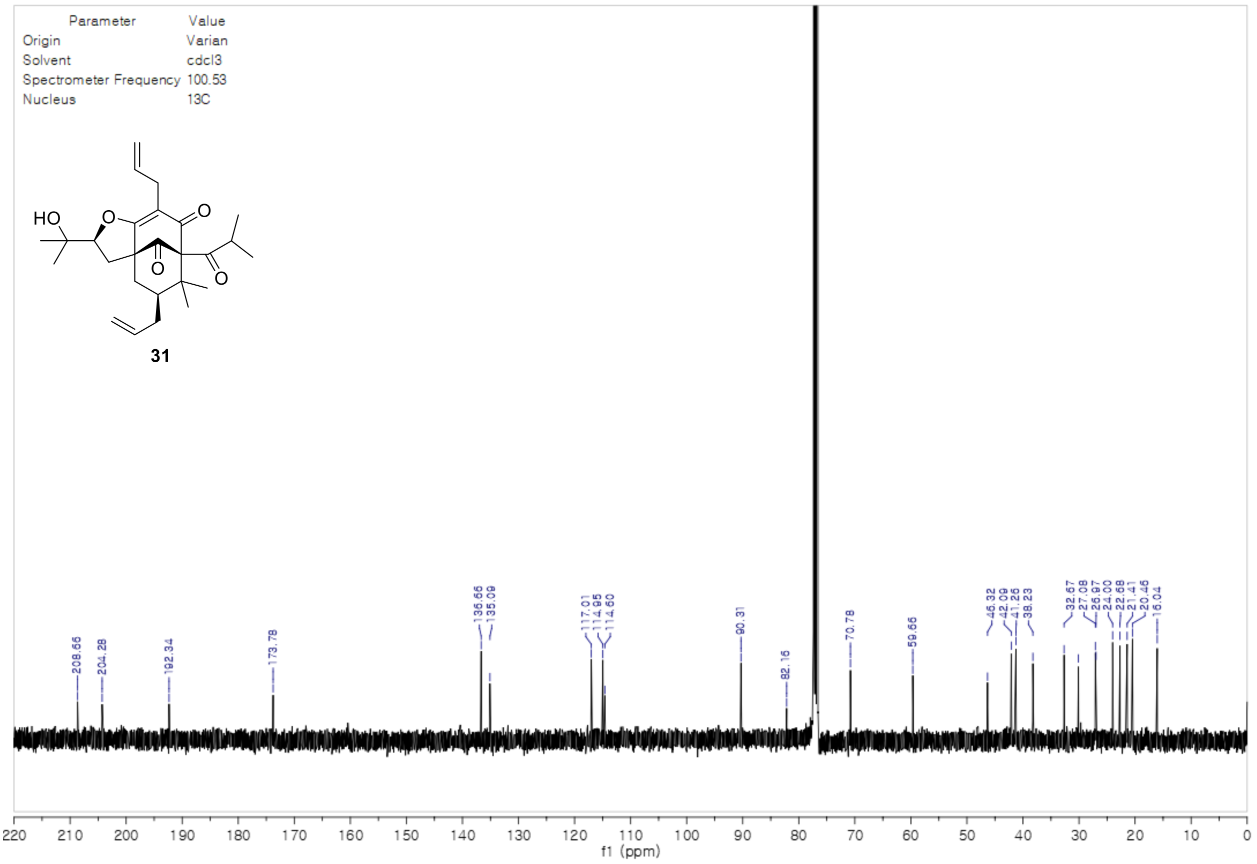

Garsubellin A (1) ^{1}H NMR

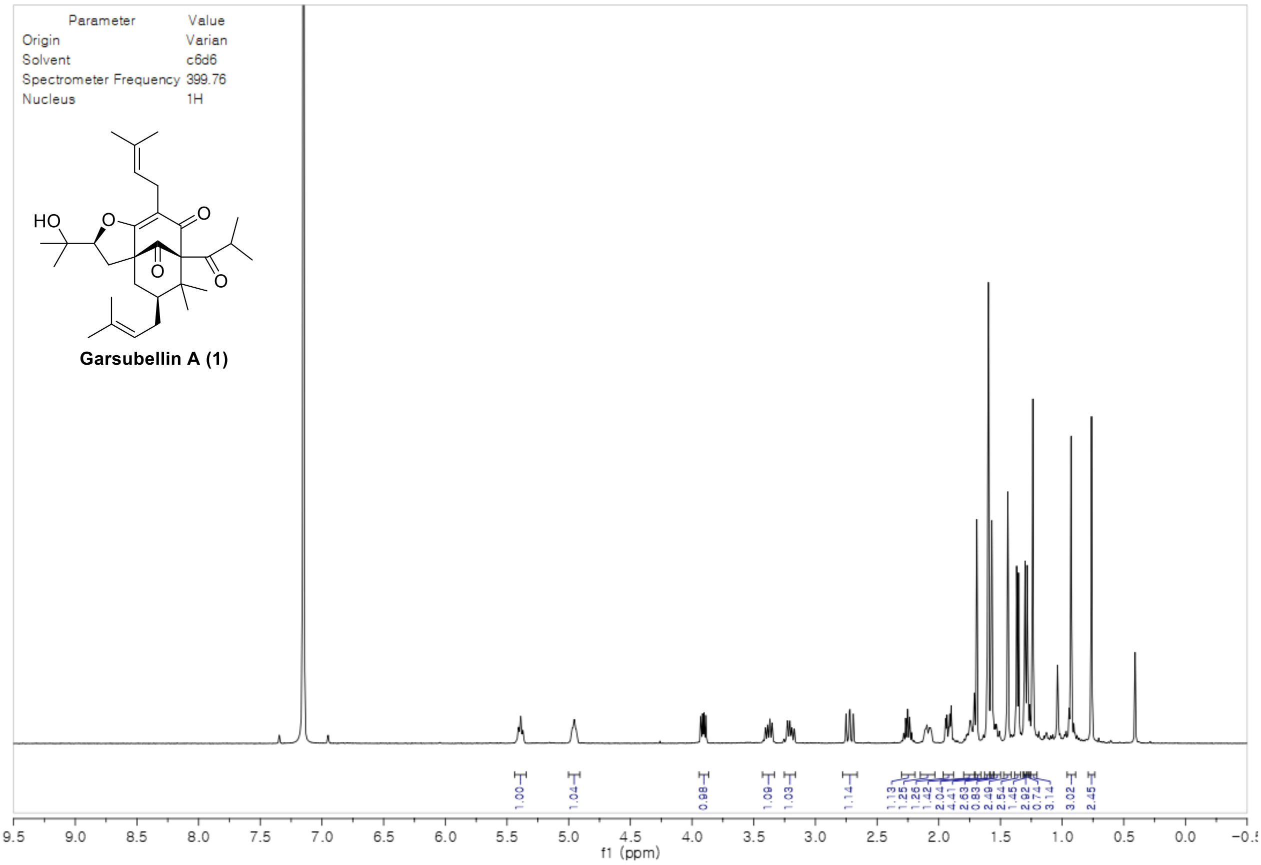

Garsubellin A (1) ^{13}C NMR

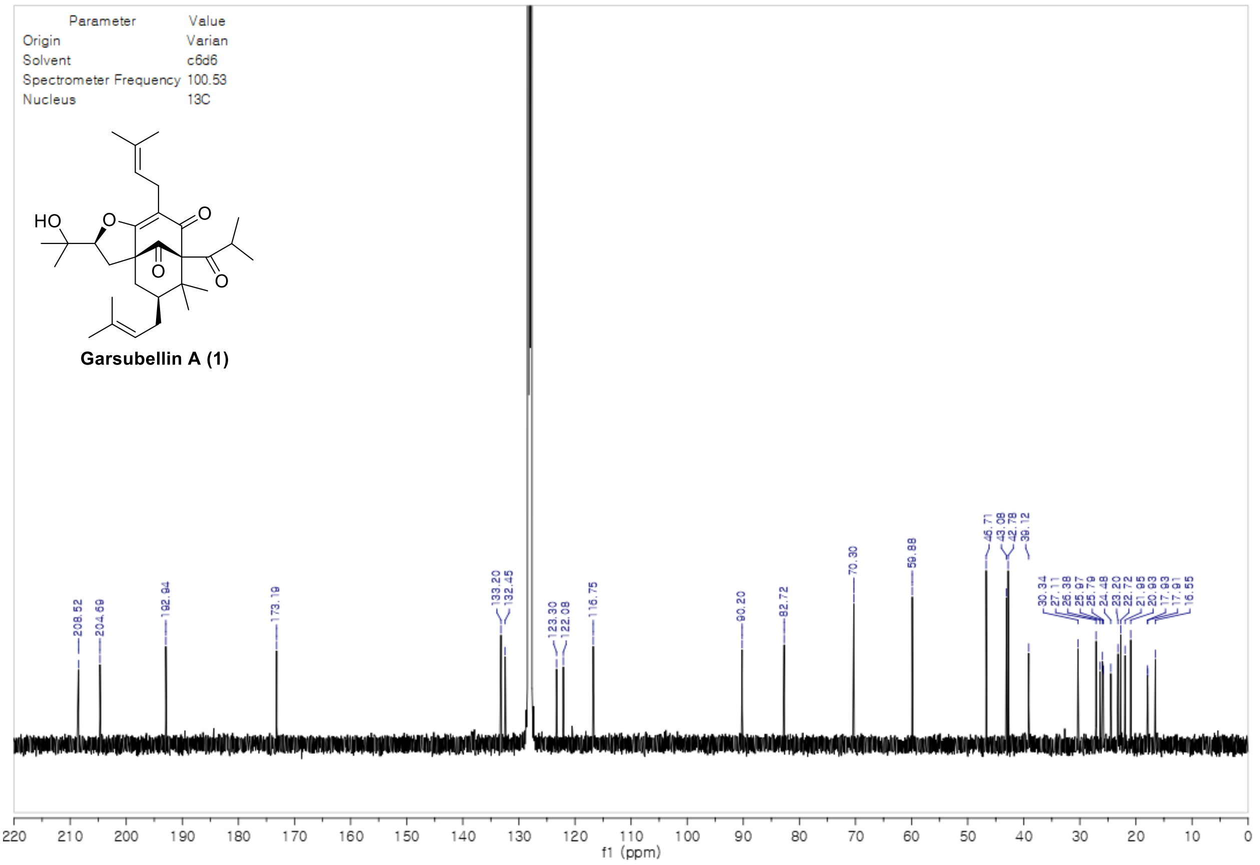

Compound 28’a ¹H NMR

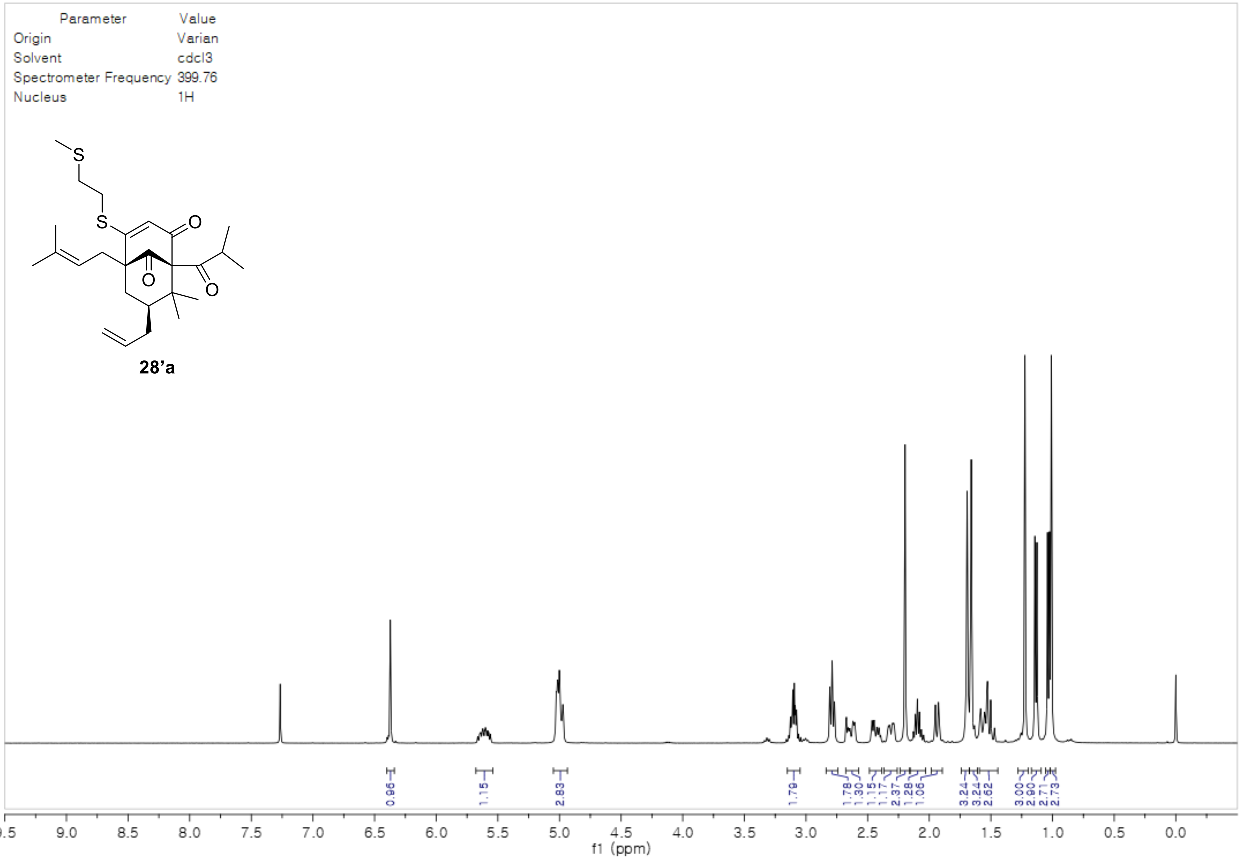

Compound 28’a ¹³C NMR

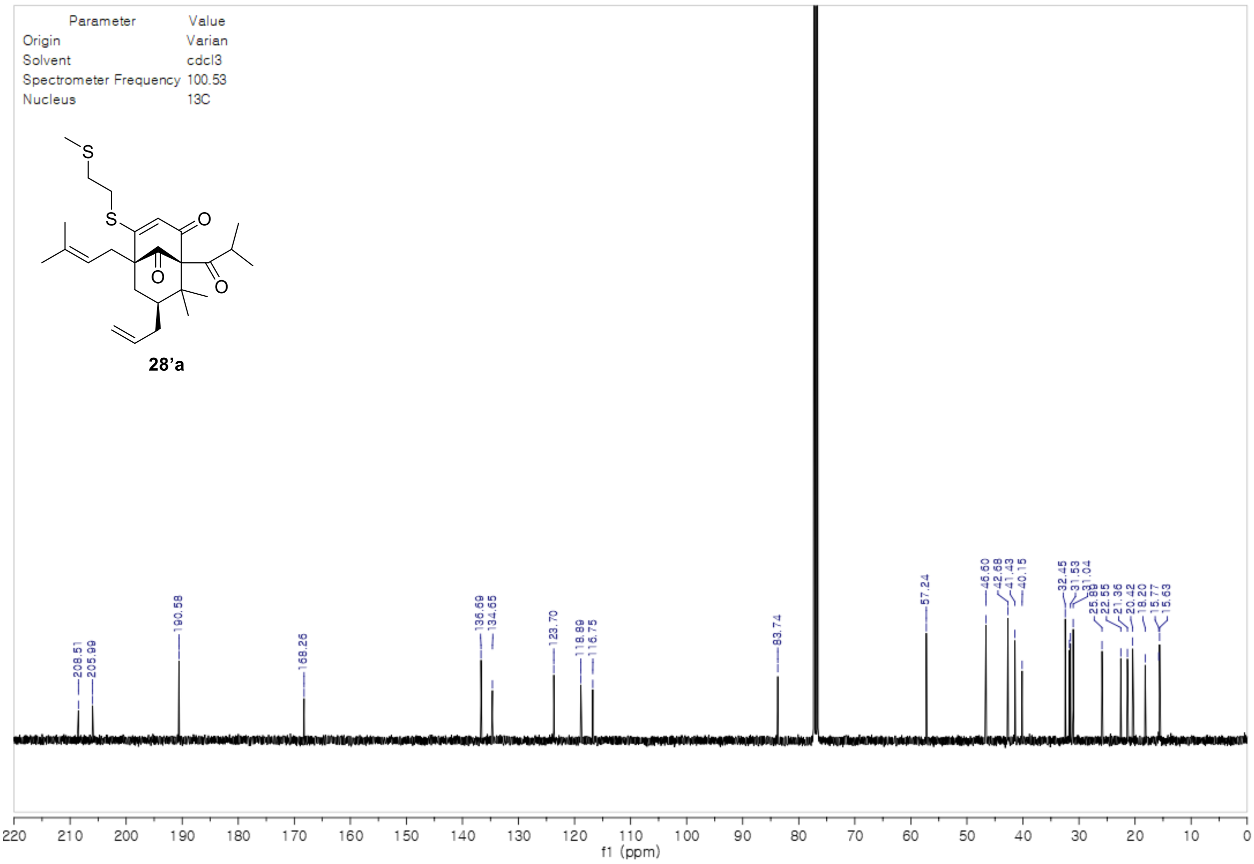

Compound 28’b ^{1}H NMR

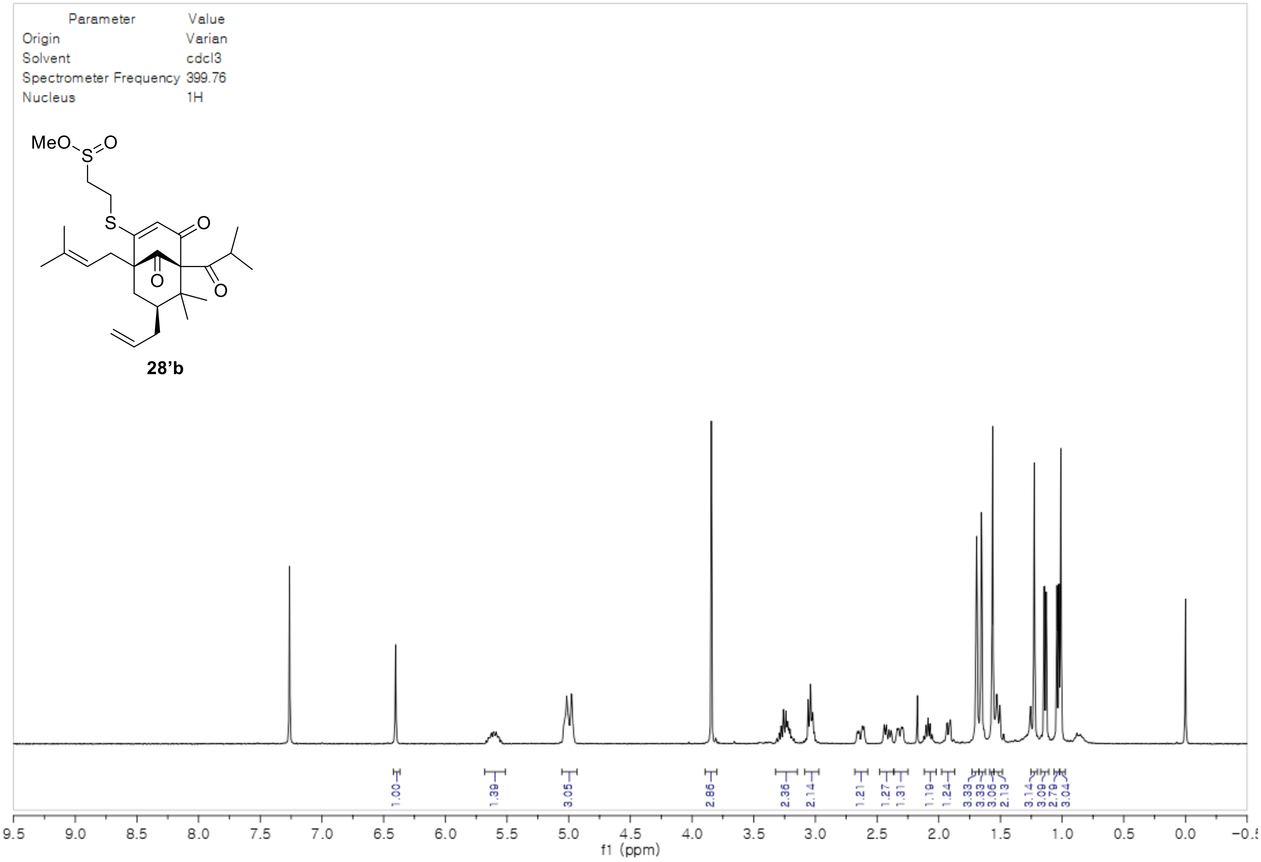

Compound 28’b ^{13}C NMR

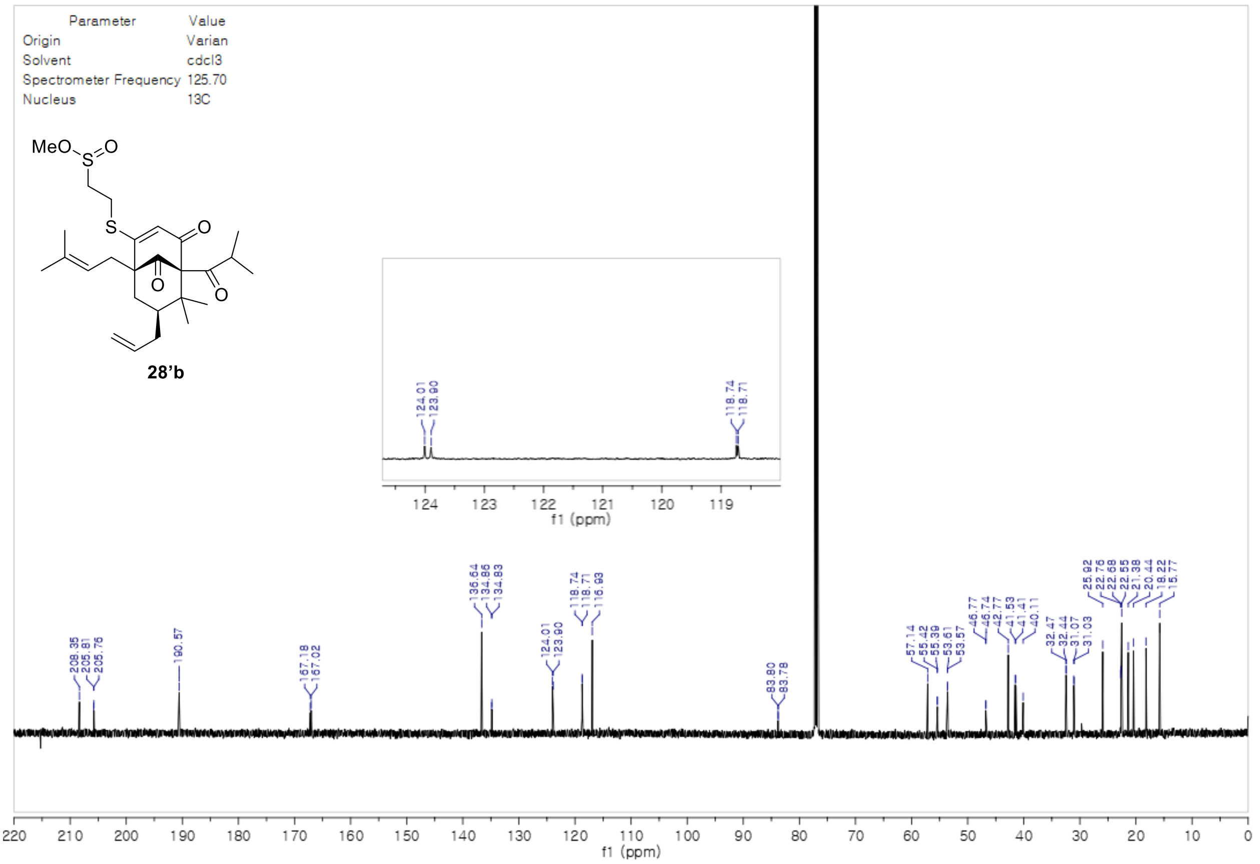

Compound 27’ ^{1}H NMR

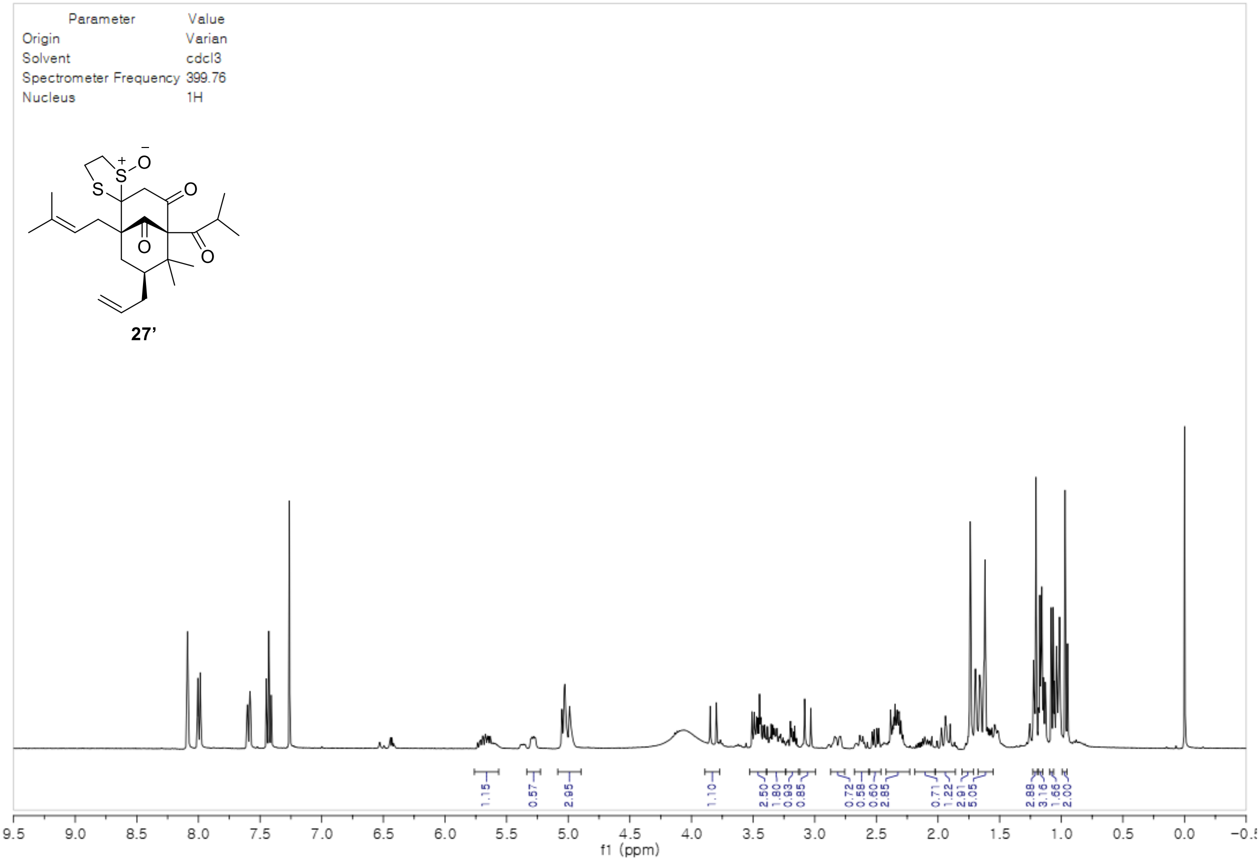

Compound 27’ COSY

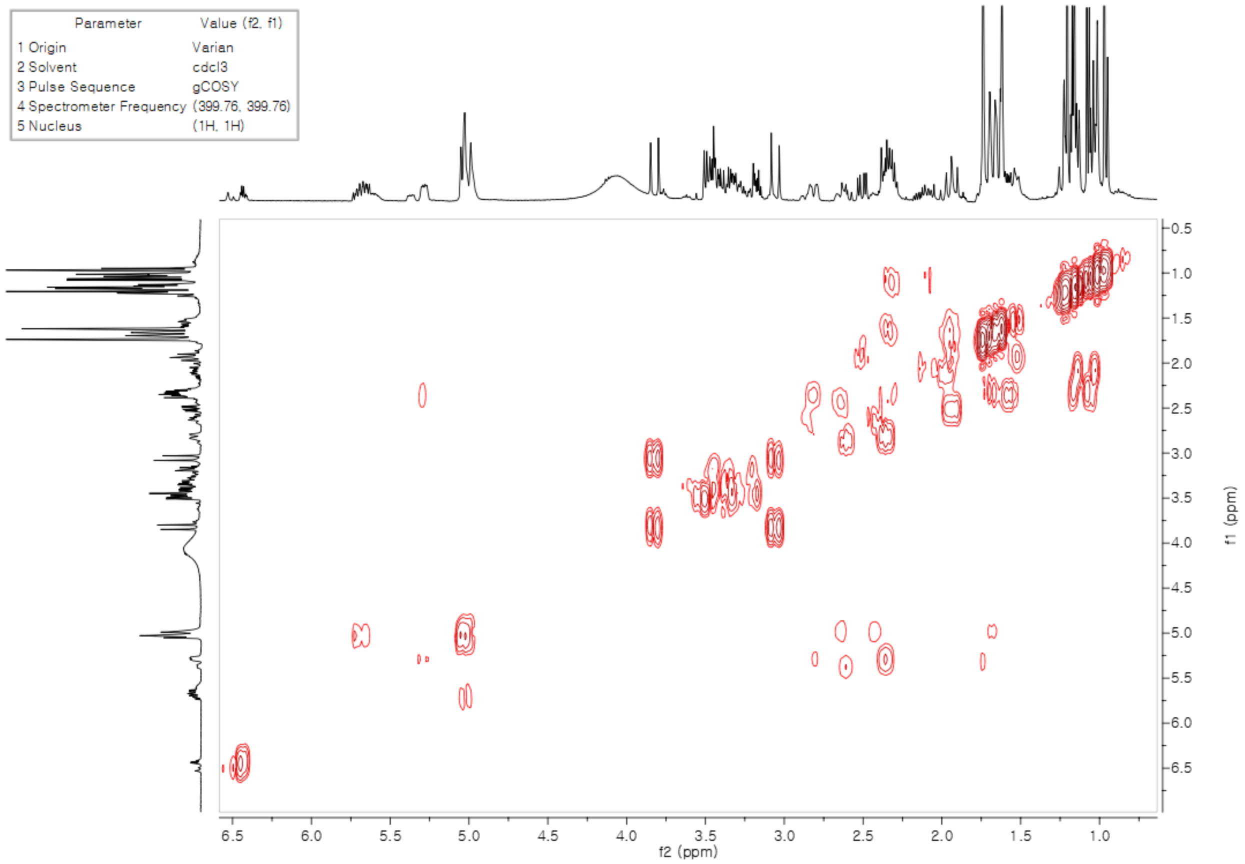

Compound 28a ^{1}H NMR

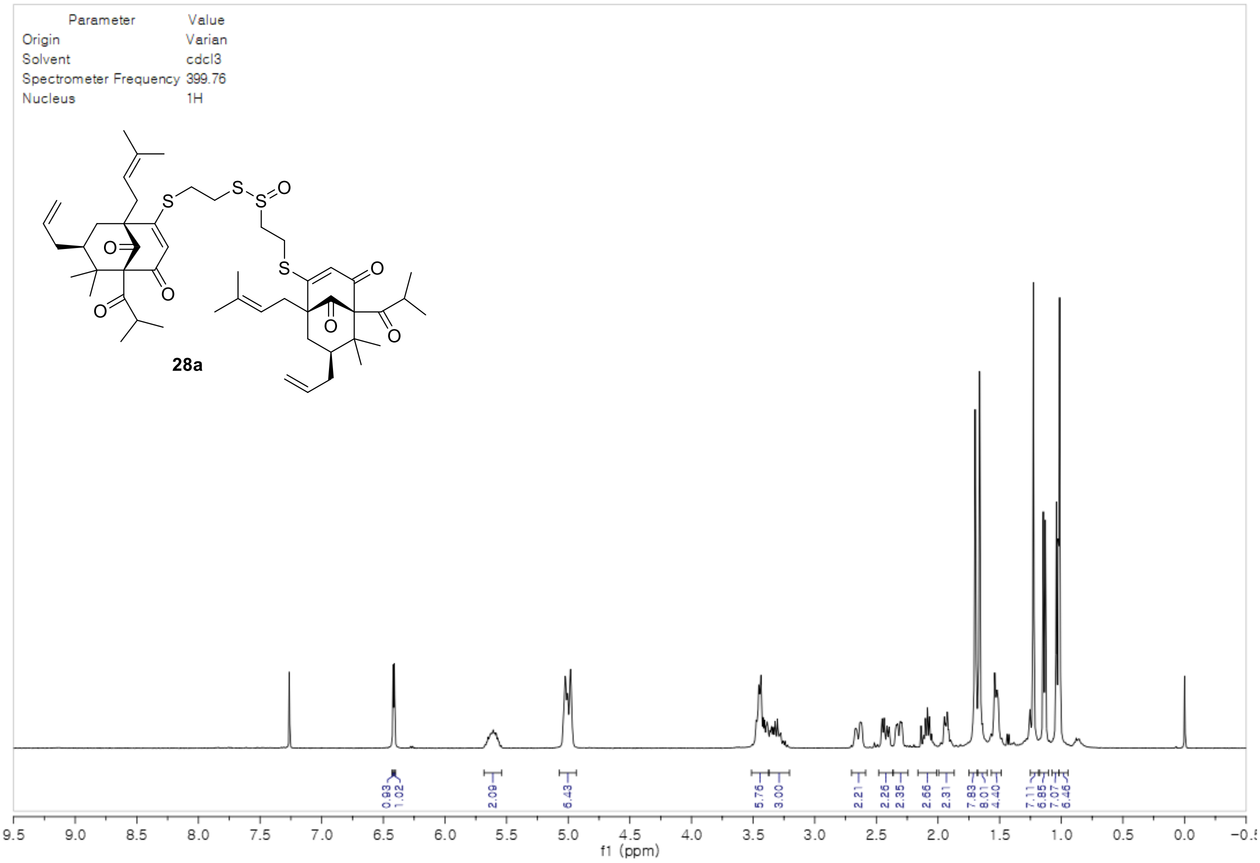

Compound 28a ^{13}C NMR

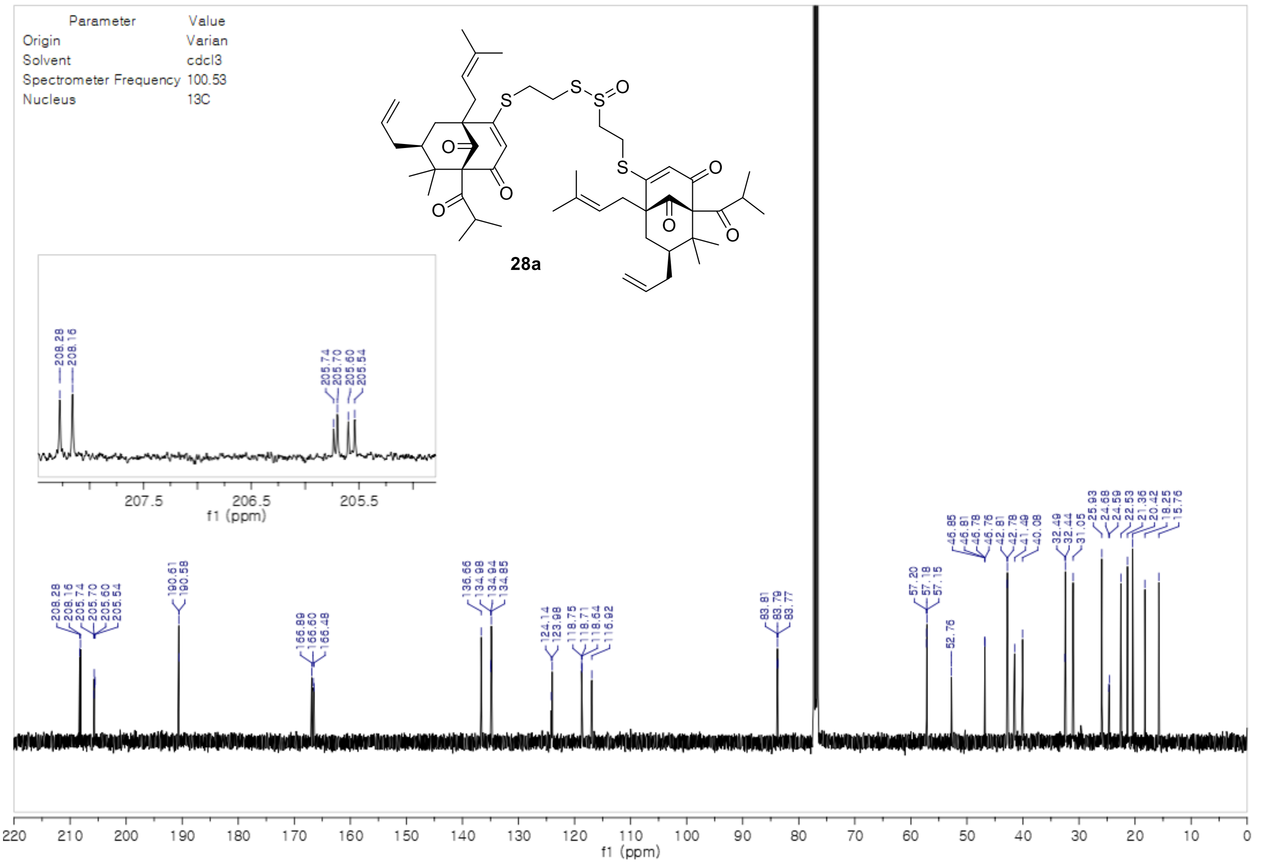

Compound 28b ^{1}H NMR

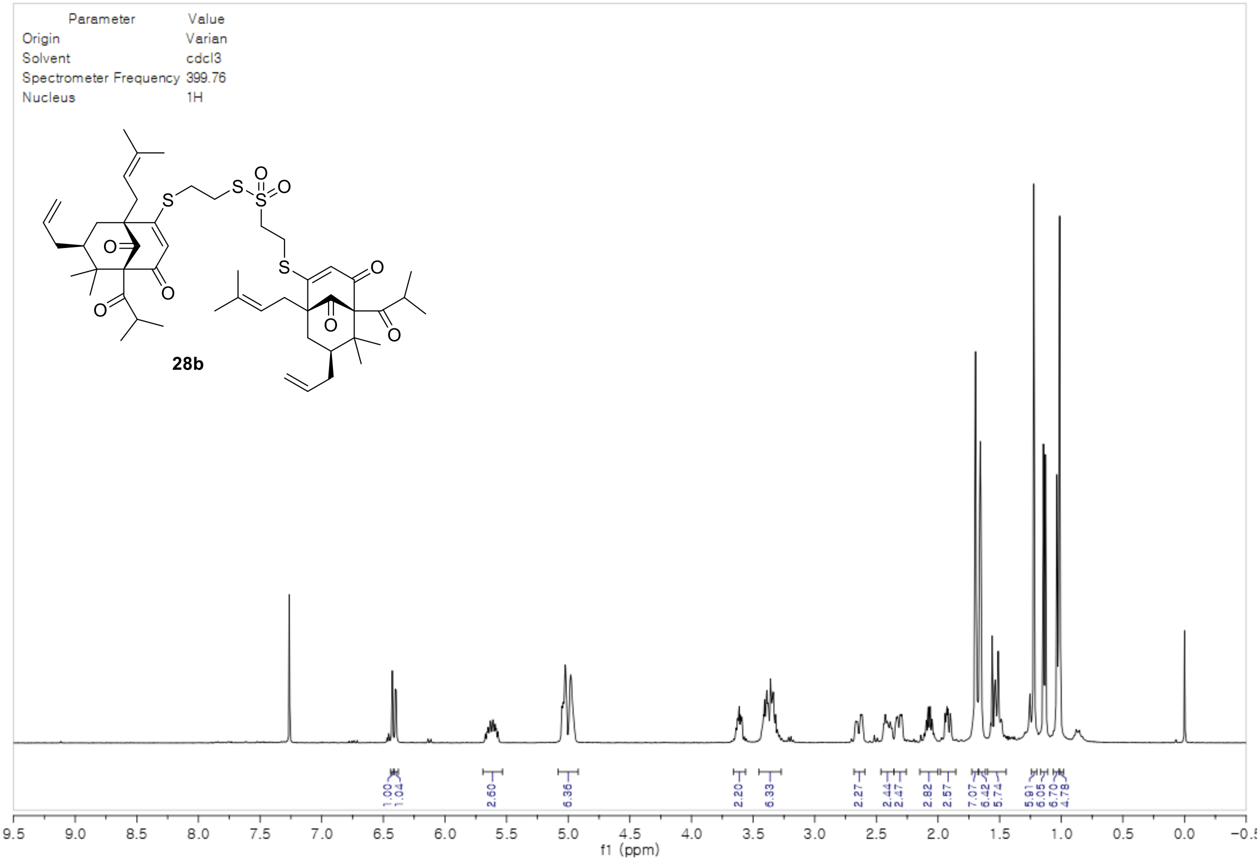

Compound 28b ^{13}C NMR

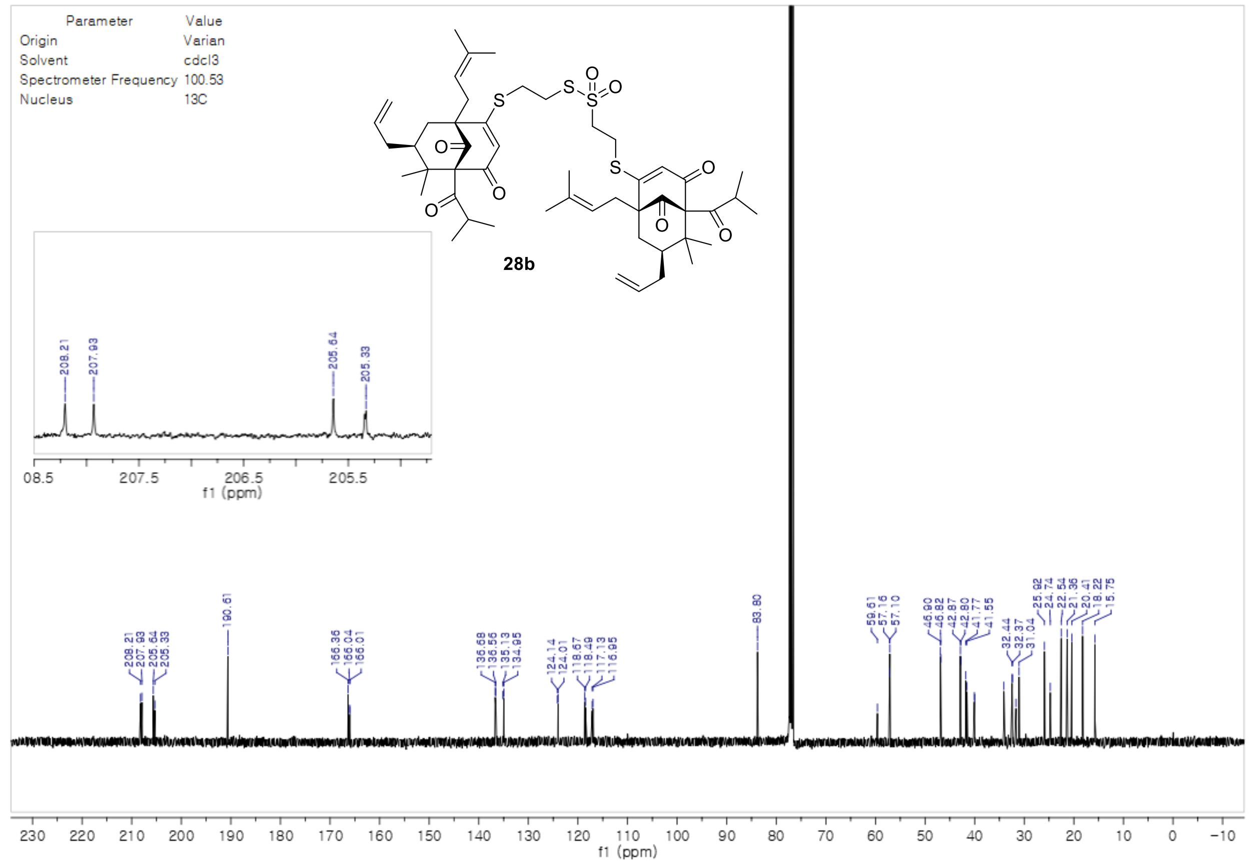

Compound 29 ^{1}H NMR

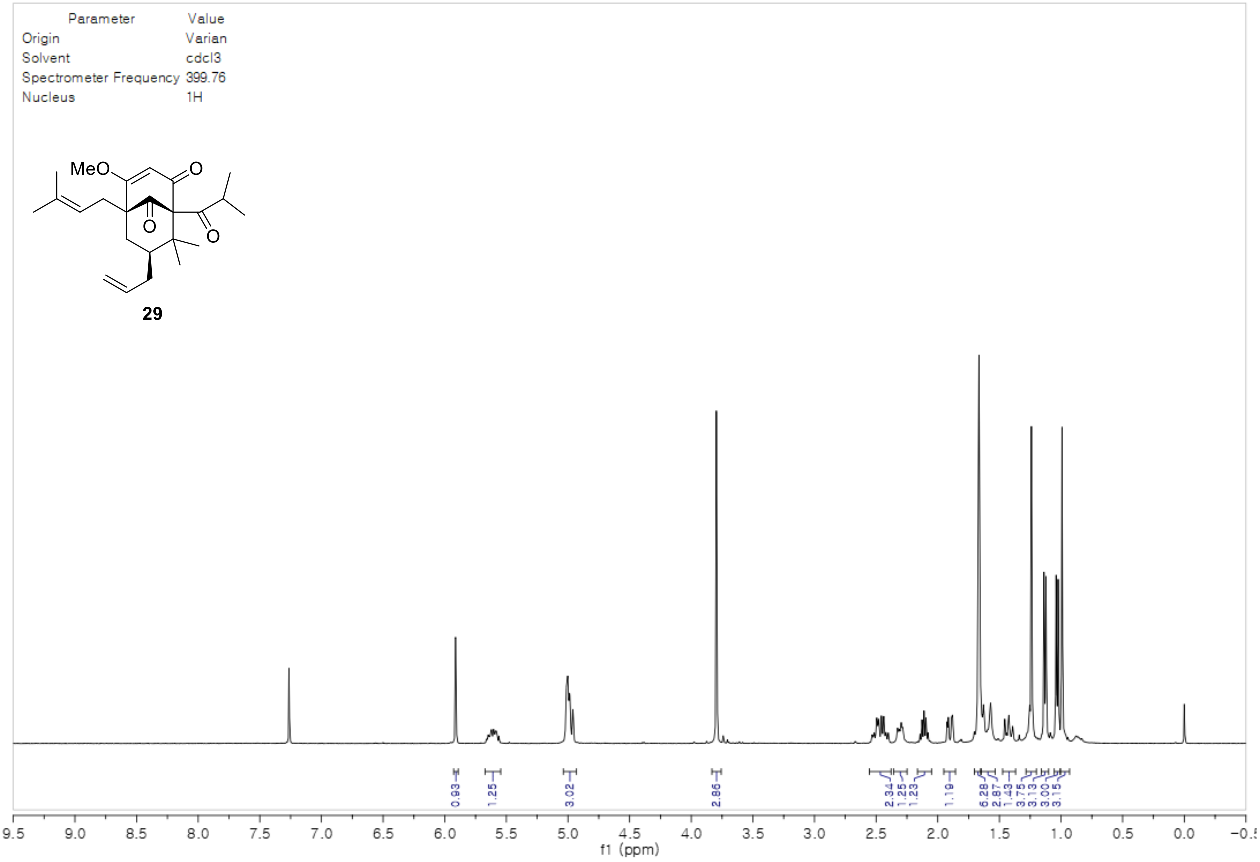

Compound 29 ^{13}C NMR

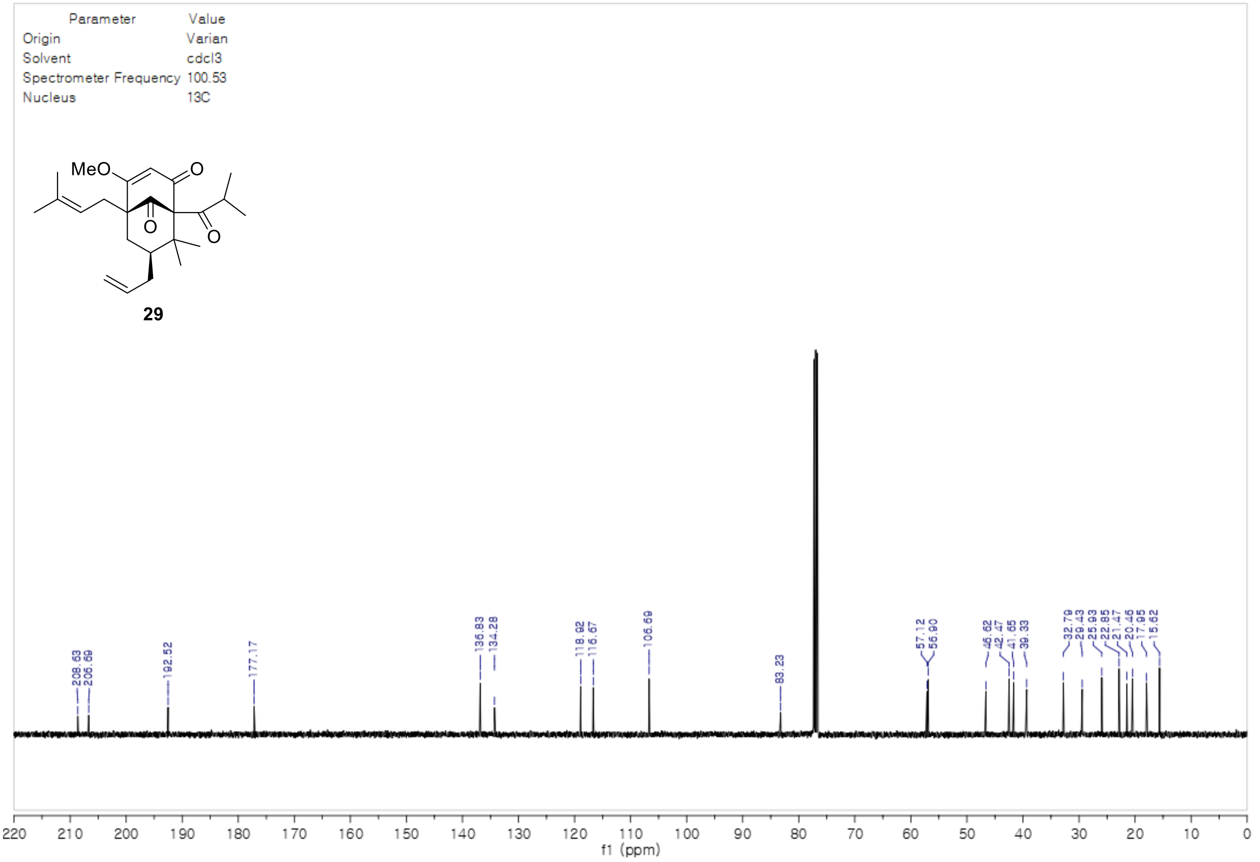

Compound 29’ ^{1}H NMR

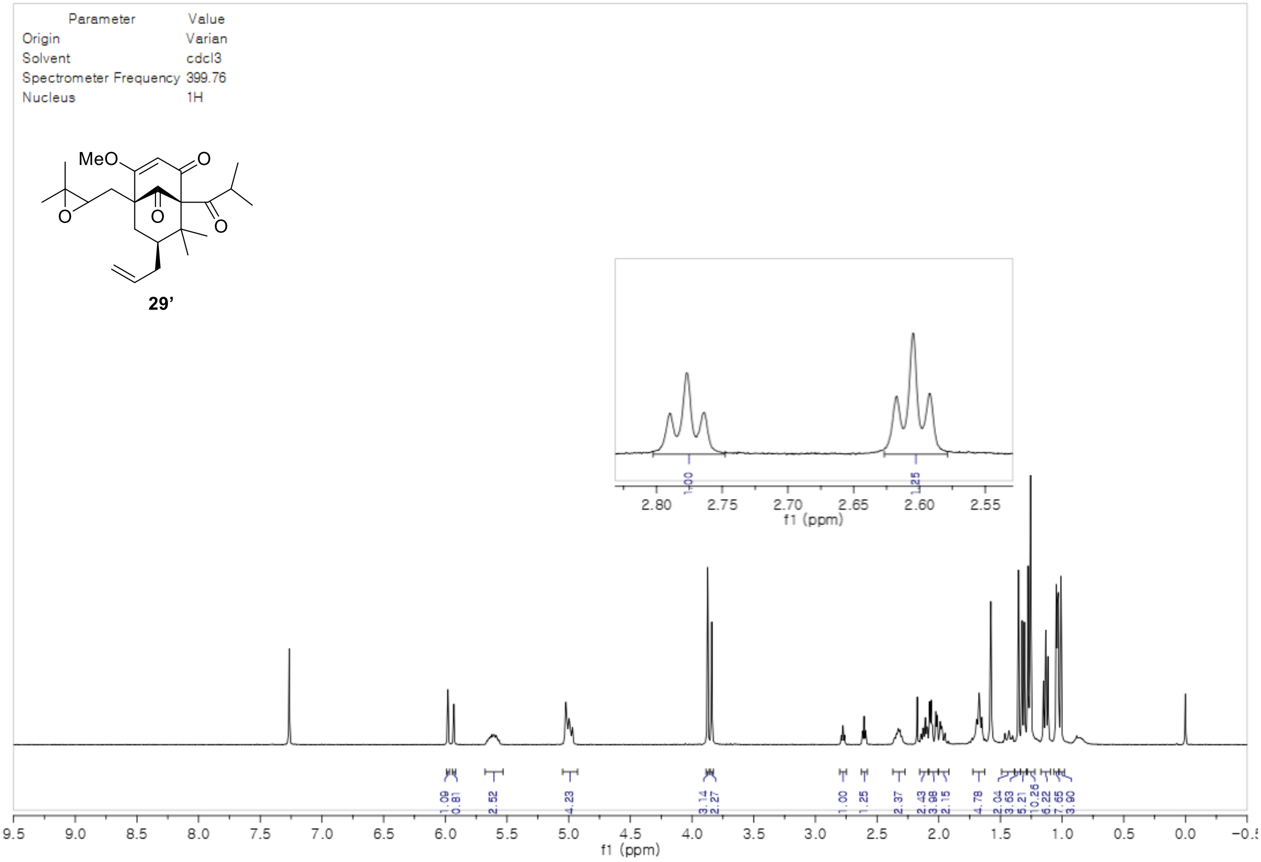

Compound 29’ ^{13}C NMR

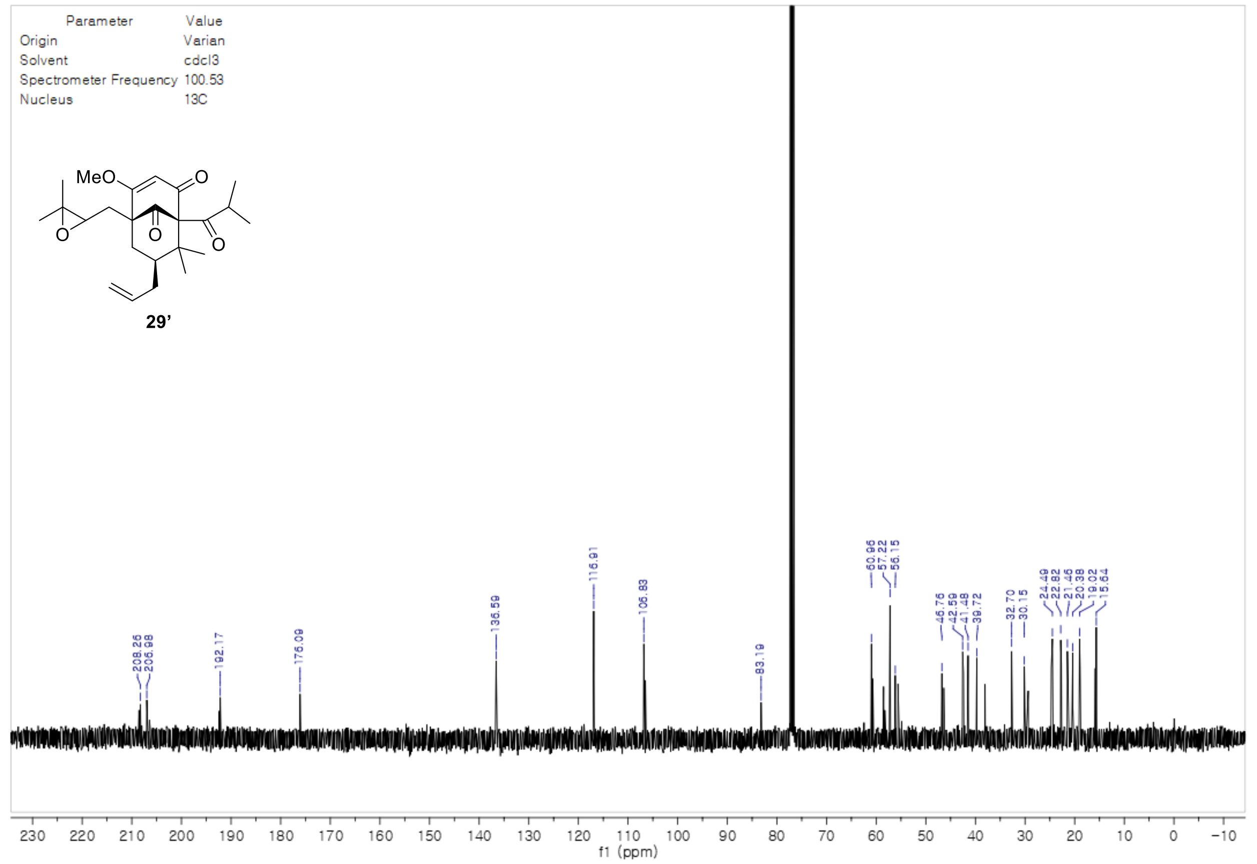

6. X-ray Crystallographic information

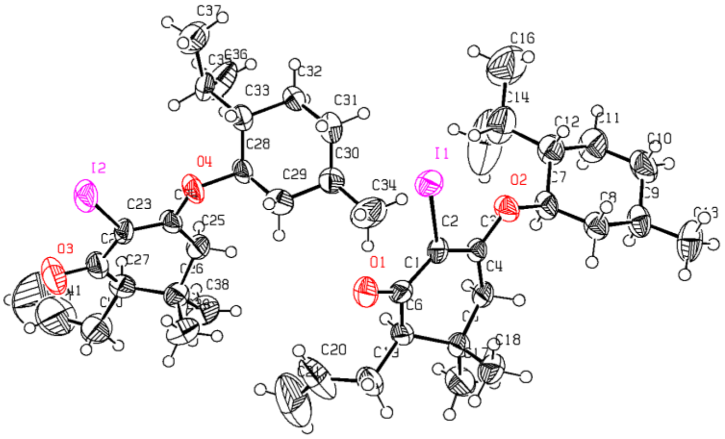

Table S9. Crystal data and structure refinement for **JDS_1_040_A_P43.**

Identification code	JDS_1_040_A_P43	
Empirical formula	C21 H33 I O2	
Formula weight	444.37	
Temperature	223(2) K	
Wavelength	0.71073 Å	
Crystal system	Tetragonal	
Space group	$P4_3$	
Unit cell dimensions	a = 12.6863(15) Å	α= 90°.
	b = 12.6863(15) Å	β= 90°.
	c = 28.298(5) Å	γ= 90°.
Volume	4554.4(13) $Å^3$	
Z	8	
Density (calculated)	1.296 Mg/m^3	
Absorption coefficient	1.415 mm^{-1}	
F(000)	1824	
Crystal size	0.374 x 0.097 x 0.076 mm^3	
Theta range for data collection	2.156 to 28.720°.	
Index ranges	-17<=h<=17, -17<=k<=17, -38<=l<=38	
Reflections collected	112204	
Independent reflections	11675 [R(int) = 0.1207]	
Completeness to theta = 25.242°	100.0 %	
Absorption correction	Semi-empirical from equivalents	
Max. and min. transmission	1.0000 and 0.8185	
Refinement method	Full-matrix least-squares on F^2	
Data / restraints / parameters	11675 / 3 / 443	
Goodness-of-fit on F^2	1.033	
Final R indices [I>2sigma(I)]	R1 = 0.0496, wR2 = 0.0894	

R indices (all data)	R1 = 0.1156, wR2 = 0.1122
Absolute structure parameter	0.025(11)
Extinction coefficient	n/a
Largest diff. peak and hole	0.860 and -1.048 e.Å^{-3}

Table S10. Atomic coordinates (x 10^4) and equivalent isotropic displacement parameters (Å^{2}x 10^3) for JDS_1_040_A_P43. U(eq) is defined as one third of the trace of the orthogonalized U^{ij} tensor.

	x	y	z	U(eq)
O(1)	5089(6)	4976(6)	4564(2)	57(2)
C(1)	4736(7)	5065(8)	4971(3)	43(2)
C(2)	4140(7)	4238(7)	5180(3)	41(2)
C(3)	3762(7)	4286(7)	5637(3)	34(2)
C(4)	4016(8)	5219(8)	5944(3)	44(2)
C(5)	4994(8)	5854(8)	5782(3)	46(2)
C(6)	4872(8)	6095(7)	5248(3)	45(2)
O(2)	3148(5)	3498(5)	5791(2)	48(2)
C(7)	2690(8)	3502(8)	6268(3)	47(2)
C(8)	3450(8)	2961(8)	6600(3)	48(2)
C(9)	3002(9)	2882(8)	7105(3)	55(3)
C(10)	1962(10)	2296(9)	7079(4)	73(3)
C(11)	1172(9)	2832(12)	6735(4)	82(4)
C(12)	1666(8)	2888(10)	6229(4)	62(3)
C(13)	3770(10)	2320(10)	7429(3)	76(4)
C(14)	877(9)	3321(13)	5855(5)	86(4)
C(15)	386(15)	4328(17)	5998(6)	165(10)
C(16)	53(12)	2422(17)	5726(7)	143(8)
I(1)	3738(1)	2924(1)	4765(1)	53(1)
C(17)	5030(11)	6872(9)	6076(4)	73(4)
C(18)	6009(9)	5216(10)	5885(4)	65(3)
C(19)	5752(9)	6783(9)	5028(4)	65(3)
C(20)	5326(14)	7564(13)	4695(7)	127(7)
C(21)	5265(19)	8487(16)	4664(8)	182(10)
O(3)	1376(8)	10896(6)	2744(3)	91(3)
C(22)	1110(9)	10498(9)	3130(4)	57(3)
C(23)	969(8)	9386(8)	3189(3)	47(2)
C(24)	743(8)	8932(8)	3605(3)	45(2)
C(25)	643(8)	9574(8)	4044(3)	52(3)
C(26)	1230(9)	10640(9)	4023(4)	54(3)
C(27)	883(9)	11191(8)	3549(4)	57(3)
O(4)	637(6)	7887(5)	3618(2)	61(2)
C(28)	273(8)	7307(7)	4036(3)	46(2)
C(29)	1235(8)	6968(8)	4319(4)	55(3)
C(30)	935(8)	6209(8)	4724(4)	59(3)

C(31)	351(10)	5283(9)	4514(4)	74(3)
C(32)	-609(9)	5641(9)	4245(4)	67(3)
C(33)	-356(8)	6394(8)	3840(3)	49(2)
C(34)	1912(9)	5892(11)	5001(6)	97(5)
C(35)	-1352(9)	6737(9)	3547(3)	58(3)
C(36)	-2221(11)	7191(13)	3851(5)	99(5)
C(37)	-1743(12)	5837(10)	3236(5)	92(5)
I(2)	1199(1)	8419(1)	2590(1)	72(1)
C(38)	901(10)	11273(10)	4466(4)	76(4)
C(39)	2394(9)	10472(10)	4026(4)	74(4)
C(40)	1304(12)	12322(9)	3487(5)	84(4)
C(41)	660(20)	12979(16)	3177(10)	172(11)
C(42)	-100(20)	13410(20)	3101(14)	258(18)

Table S11. Bond lengths [Å] and angles [°] for JDS_1_040_A_P43.

O(1)-C(1)	1.241(9)	C(11)-C(12)	1.566(14)
C(1)-C(2)	1.422(12)	C(11)-H(11A)	0.9800
C(1)-C(6)	1.534(12)	C(11)-H(11B)	0.9800
C(2)-C(3)	1.380(10)	C(12)-C(14)	1.556(16)
C(2)-I(1)	2.102(9)	C(12)-H(12)	0.9900
C(3)-O(2)	1.339(10)	C(13)-H(13A)	0.9700
C(3)-C(4)	1.505(11)	C(13)-H(13B)	0.9700
C(4)-C(5)	1.549(13)	C(13)-H(13C)	0.9700
C(4)-H(4A)	0.9800	C(14)-C(15)	1.48(2)
C(4)-H(4B)	0.9800	C(14)-C(16)	1.59(2)
C(5)-C(17)	1.538(14)	C(14)-H(14)	0.9900
C(5)-C(18)	1.548(15)	C(15)-H(15A)	0.9700
C(5)-C(6)	1.550(12)	C(15)-H(15B)	0.9700
C(6)-C(19)	1.547(13)	C(15)-H(15C)	0.9700
C(6)-H(6)	0.9900	C(16)-H(16A)	0.9700
O(2)-C(7)	1.472(10)	C(16)-H(16B)	0.9700
C(7)-C(8)	1.511(13)	C(16)-H(16C)	0.9700
C(7)-C(12)	1.519(14)	C(17)-H(17A)	0.9700
C(7)-H(7)	0.9900	C(17)-H(17B)	0.9700
C(8)-C(9)	1.539(12)	C(17)-H(17C)	0.9700
C(8)-H(8A)	0.9800	C(18)-H(18A)	0.9700
C(8)-H(8B)	0.9800	C(18)-H(18B)	0.9700
C(9)-C(13)	1.516(14)	C(18)-H(18C)	0.9700
C(9)-C(10)	1.517(15)	C(19)-C(20)	1.471(19)
C(9)-H(9)	0.9900	C(19)-H(19A)	0.9800
C(10)-C(11)	1.553(16)	C(19)-H(19B)	0.9800
C(10)-H(10A)	0.9800	C(20)-C(21)	1.18(2)
C(10)-H(10B)	0.9800	C(20)-H(20)	0.9400

C(21)-H(21A)	0.9400	C(38)-H(38A)	0.9700
C(21)-H(21B)	0.9400	C(38)-H(38B)	0.9700
O(3)-C(22)	1.248(11)	C(38)-H(38C)	0.9700
C(22)-C(23)	1.432(14)	C(39)-H(39A)	0.9700
C(22)-C(27)	1.505(14)	C(39)-H(39B)	0.9700
C(23)-C(24)	1.343(12)	C(39)-H(39C)	0.9700
C(23)-I(2)	2.112(9)	C(40)-C(41)	1.459(18)
C(24)-O(4)	1.332(11)	C(40)-H(40A)	0.9800
C(24)-C(25)	1.492(12)	C(40)-H(40B)	0.9800
C(25)-C(26)	1.544(14)	C(41)-C(42)	1.13(2)
C(25)-H(25A)	0.9800	C(41)-H(41)	0.9400
C(25)-H(25B)	0.9800	C(42)-H(42A)	0.9400
C(26)-C(39)	1.493(15)	C(42)-H(42B)	0.9400
C(26)-C(38)	1.546(14)		
C(26)-C(27)	1.574(14)	O(1)-C(1)-C(2)	120.8(8)
C(27)-C(40)	1.542(15)	O(1)-C(1)-C(6)	120.7(8)
C(27)-H(27)	0.9900	C(2)-C(1)-C(6)	118.4(7)
O(4)-C(28)	1.467(10)	C(3)-C(2)-C(1)	122.8(8)
C(28)-C(33)	1.512(13)	C(3)-C(2)-I(1)	118.3(6)
C(28)-C(29)	1.522(14)	C(1)-C(2)-I(1)	118.8(6)
C(28)-H(28)	0.9900	O(2)-C(3)-C(2)	118.3(7)
C(29)-C(30)	1.544(14)	O(2)-C(3)-C(4)	121.5(7)
C(29)-H(29A)	0.9800	C(2)-C(3)-C(4)	120.1(8)
C(29)-H(29B)	0.9800	C(3)-C(4)-C(5)	114.2(7)
C(30)-C(31)	1.511(15)	C(3)-C(4)-H(4A)	108.7
C(30)-C(34)	1.521(14)	C(5)-C(4)-H(4A)	108.7
C(30)-H(30)	0.9900	C(3)-C(4)-H(4B)	108.7
C(31)-C(32)	1.506(15)	C(5)-C(4)-H(4B)	108.7
C(31)-H(31A)	0.9800	H(4A)-C(4)-H(4B)	107.6
C(31)-H(31B)	0.9800	C(17)-C(5)-C(18)	108.2(9)
C(32)-C(33)	1.528(13)	C(17)-C(5)-C(4)	107.5(8)
C(32)-H(32A)	0.9800	C(18)-C(5)-C(4)	109.7(8)
C(32)-H(32B)	0.9800	C(17)-C(5)-C(6)	111.4(8)
C(33)-C(35)	1.572(14)	C(18)-C(5)-C(6)	111.7(8)
C(33)-H(33)	0.9900	C(4)-C(5)-C(6)	108.1(7)
C(34)-H(34A)	0.9700	C(1)-C(6)-C(19)	110.9(8)
C(34)-H(34B)	0.9700	C(1)-C(6)-C(5)	110.0(7)
C(34)-H(34C)	0.9700	C(19)-C(6)-C(5)	115.5(8)
C(35)-C(36)	1.513(17)	C(1)-C(6)-H(6)	106.7
C(35)-C(37)	1.525(16)	C(19)-C(6)-H(6)	106.7
C(35)-H(35)	0.9900	C(5)-C(6)-H(6)	106.7
C(36)-H(36A)	0.9700	C(3)-O(2)-C(7)	121.7(7)
C(36)-H(36B)	0.9700	O(2)-C(7)-C(8)	108.5(7)
C(36)-H(36C)	0.9700	O(2)-C(7)-C(12)	105.6(8)
C(37)-H(37A)	0.9700	C(8)-C(7)-C(12)	111.0(8)
C(37)-H(37B)	0.9700	O(2)-C(7)-H(7)	110.5
C(37)-H(37C)	0.9700	C(8)-C(7)-H(7)	110.5

C(12)-C(7)-H(7)	110.5
C(7)-C(8)-C(9)	111.8(8)
C(7)-C(8)-H(8A)	109.3
C(9)-C(8)-H(8A)	109.3
C(7)-C(8)-H(8B)	109.3
C(9)-C(8)-H(8B)	109.3
H(8A)-C(8)-H(8B)	107.9
C(13)-C(9)-C(10)	110.9(9)
C(13)-C(9)-C(8)	110.7(9)
C(10)-C(9)-C(8)	107.9(8)
C(13)-C(9)-H(9)	109.1
C(10)-C(9)-H(9)	109.1
C(8)-C(9)-H(9)	109.1
C(9)-C(10)-C(11)	112.2(9)
C(9)-C(10)-H(10A)	109.2
C(11)-C(10)-H(10A)	109.2
C(9)-C(10)-H(10B)	109.2
C(11)-C(10)-H(10B)	109.2
H(10A)-C(10)-H(10B)	107.9
C(10)-C(11)-C(12)	109.5(10)
C(10)-C(11)-H(11A)	109.8
C(12)-C(11)-H(11A)	109.8
C(10)-C(11)-H(11B)	109.8
C(12)-C(11)-H(11B)	109.8
H(11A)-C(11)-H(11B)	108.2
C(7)-C(12)-C(14)	114.8(10)
C(7)-C(12)-C(11)	107.4(9)
C(14)-C(12)-C(11)	112.3(10)
C(7)-C(12)-H(12)	107.3
C(14)-C(12)-H(12)	107.3
C(11)-C(12)-H(12)	107.3
C(9)-C(13)-H(13A)	109.5
C(9)-C(13)-H(13B)	109.5
H(13A)-C(13)-H(13B)	109.5
C(9)-C(13)-H(13C)	109.5
H(13A)-C(13)-H(13C)	109.5
H(13B)-C(13)-H(13C)	109.5
C(15)-C(14)-C(12)	113.1(13)
C(15)-C(14)-C(16)	114.0(14)
C(12)-C(14)-C(16)	109.0(12)
C(15)-C(14)-H(14)	106.8
C(12)-C(14)-H(14)	106.8
C(16)-C(14)-H(14)	106.8
C(14)-C(15)-H(15A)	109.5
C(14)-C(15)-H(15B)	109.5
H(15A)-C(15)-H(15B)	109.5
C(14)-C(15)-H(15C)	109.5
H(15A)-C(15)-H(15C)	109.5
H(15B)-C(15)-H(15C)	109.5
C(14)-C(16)-H(16A)	109.5
C(14)-C(16)-H(16B)	109.5
H(16A)-C(16)-H(16B)	109.5
C(14)-C(16)-H(16C)	109.5
H(16A)-C(16)-H(16C)	109.5
H(16B)-C(16)-H(16C)	109.5
C(5)-C(17)-H(17A)	109.5
C(5)-C(17)-H(17B)	109.5
H(17A)-C(17)-H(17B)	109.5
C(5)-C(17)-H(17C)	109.5
H(17A)-C(17)-H(17C)	109.5
H(17B)-C(17)-H(17C)	109.5
C(5)-C(18)-H(18A)	109.5
C(5)-C(18)-H(18B)	109.5
H(18A)-C(18)-H(18B)	109.5
C(5)-C(18)-H(18C)	109.5
H(18A)-C(18)-H(18C)	109.5
H(18B)-C(18)-H(18C)	109.5
C(20)-C(19)-C(6)	111.9(10)
C(20)-C(19)-H(19A)	109.2
C(6)-C(19)-H(19A)	109.2
C(20)-C(19)-H(19B)	109.2
C(6)-C(19)-H(19B)	109.2
H(19A)-C(19)-H(19B)	107.9
C(21)-C(20)-C(19)	138(2)
C(21)-C(20)-H(20)	111.1
C(19)-C(20)-H(20)	111.1
C(20)-C(21)-H(21A)	120.0
C(20)-C(21)-H(21B)	120.0
H(21A)-C(21)-H(21B)	120.0
O(3)-C(22)-C(23)	122.3(10)
O(3)-C(22)-C(27)	120.3(10)
C(23)-C(22)-C(27)	117.4(8)
C(24)-C(23)-C(22)	123.5(9)
C(24)-C(23)-I(2)	119.0(7)
C(22)-C(23)-I(2)	117.5(7)
O(4)-C(24)-C(23)	118.2(8)
O(4)-C(24)-C(25)	120.8(8)
C(23)-C(24)-C(25)	121.0(9)
C(24)-C(25)-C(26)	113.9(8)
C(24)-C(25)-H(25A)	108.8
C(26)-C(25)-H(25A)	108.8
C(24)-C(25)-H(25B)	108.8
C(26)-C(25)-H(25B)	108.8
H(25A)-C(25)-H(25B)	107.7

C(39)-C(26)-C(25)	110.6(10)	C(32)-C(33)-H(33)	106.9
C(39)-C(26)-C(38)	109.7(9)	C(35)-C(33)-H(33)	106.9
C(25)-C(26)-C(38)	107.0(9)	C(30)-C(34)-H(34A)	109.5
C(39)-C(26)-C(27)	110.2(9)	C(30)-C(34)-H(34B)	109.5
C(25)-C(26)-C(27)	106.7(8)	H(34A)-C(34)-H(34B)	109.5
C(38)-C(26)-C(27)	112.6(9)	C(30)-C(34)-H(34C)	109.5
C(22)-C(27)-C(40)	112.7(10)	H(34A)-C(34)-H(34C)	109.5
C(22)-C(27)-C(26)	111.0(9)	H(34B)-C(34)-H(34C)	109.5
C(40)-C(27)-C(26)	114.4(9)	C(36)-C(35)-C(37)	112.1(11)
C(22)-C(27)-H(27)	106.0	C(36)-C(35)-C(33)	113.0(9)
C(40)-C(27)-H(27)	106.0	C(37)-C(35)-C(33)	111.0(10)
C(26)-C(27)-H(27)	106.0	C(36)-C(35)-H(35)	106.8
C(24)-O(4)-C(28)	123.6(7)	C(37)-C(35)-H(35)	106.8
O(4)-C(28)-C(33)	104.7(7)	C(33)-C(35)-H(35)	106.8
O(4)-C(28)-C(29)	108.2(8)	C(35)-C(36)-H(36A)	109.5
C(33)-C(28)-C(29)	113.6(8)	C(35)-C(36)-H(36B)	109.5
O(4)-C(28)-H(28)	110.0	H(36A)-C(36)-H(36B)	109.5
C(33)-C(28)-H(28)	110.0	C(35)-C(36)-H(36C)	109.5
C(29)-C(28)-H(28)	110.0	H(36A)-C(36)-H(36C)	109.5
C(28)-C(29)-C(30)	111.6(8)	H(36B)-C(36)-H(36C)	109.5
C(28)-C(29)-H(29A)	109.3	C(35)-C(37)-H(37A)	109.5
C(30)-C(29)-H(29A)	109.3	C(35)-C(37)-H(37B)	109.5
C(28)-C(29)-H(29B)	109.3	H(37A)-C(37)-H(37B)	109.5
C(30)-C(29)-H(29B)	109.3	C(35)-C(37)-H(37C)	109.5
H(29A)-C(29)-H(29B)	108.0	H(37A)-C(37)-H(37C)	109.5
C(31)-C(30)-C(34)	113.4(9)	H(37B)-C(37)-H(37C)	109.5
C(31)-C(30)-C(29)	108.3(9)	C(26)-C(38)-H(38A)	109.5
C(34)-C(30)-C(29)	110.3(9)	C(26)-C(38)-H(38B)	109.5
C(31)-C(30)-H(30)	108.2	H(38A)-C(38)-H(38B)	109.5
C(34)-C(30)-H(30)	108.2	C(26)-C(38)-H(38C)	109.5
C(29)-C(30)-H(30)	108.2	H(38A)-C(38)-H(38C)	109.5
C(32)-C(31)-C(30)	111.1(10)	H(38B)-C(38)-H(38C)	109.5
C(32)-C(31)-H(31A)	109.4	C(26)-C(39)-H(39A)	109.5
C(30)-C(31)-H(31A)	109.4	C(26)-C(39)-H(39B)	109.5
C(32)-C(31)-H(31B)	109.4	H(39A)-C(39)-H(39B)	109.5
C(30)-C(31)-H(31B)	109.4	C(26)-C(39)-H(39C)	109.5
H(31A)-C(31)-H(31B)	108.0	H(39A)-C(39)-H(39C)	109.5
C(31)-C(32)-C(33)	113.5(9)	H(39B)-C(39)-H(39C)	109.5
C(31)-C(32)-H(32A)	108.9	C(41)-C(40)-C(27)	114.0(13)
C(33)-C(32)-H(32A)	108.9	C(41)-C(40)-H(40A)	108.7
C(31)-C(32)-H(32B)	108.9	C(27)-C(40)-H(40A)	108.7
C(33)-C(32)-H(32B)	108.9	C(41)-C(40)-H(40B)	108.7
H(32A)-C(32)-H(32B)	107.7	C(27)-C(40)-H(40B)	108.7
C(28)-C(33)-C(32)	108.3(8)	H(40A)-C(40)-H(40B)	107.6
C(28)-C(33)-C(35)	113.9(8)	C(42)-C(41)-C(40)	150(4)
C(32)-C(33)-C(35)	113.6(8)	C(42)-C(41)-H(41)	105.0
C(28)-C(33)-H(33)	106.9	C(40)-C(41)-H(41)	105.0

C(41)-C(42)-H(42A)	120.0	H(42A)-C(42)-H(42B)	120.0
C(41)-C(42)-H(42B)	120.0		

Symmetry transformations used to generate equivalent atoms:

Table S12. Anisotropic displacement parameters ($Å^2$x 10^3) for JDS_1_040_A_P43. The anisotropic displacement factor exponent takes the form: $-2\pi^2[h^2 a^{*2}U^{11} + ... + 2 h k a^* b^* U^{12}]$

	U^{11}	U^{22}	U^{33}	U^{23}	U^{13}	U^{12}
O(1)	75(5)	75(5)	22(3)	-1(3)	12(3)	-16(4)
C(1)	41(5)	56(6)	32(5)	0(4)	-3(4)	-10(4)
C(2)	48(5)	51(6)	23(4)	2(4)	1(4)	-3(4)
C(3)	40(5)	39(5)	21(4)	0(3)	4(3)	0(4)
C(4)	59(6)	51(6)	22(4)	-1(4)	-2(4)	-5(5)
C(5)	59(6)	48(6)	32(5)	1(4)	-2(4)	-11(5)
C(6)	50(6)	53(6)	33(4)	4(4)	1(4)	-16(5)
O(2)	58(4)	53(4)	34(3)	1(3)	12(3)	-12(3)
C(7)	64(7)	53(6)	23(4)	0(4)	16(4)	-3(5)
C(8)	58(6)	51(6)	35(5)	1(4)	9(4)	-3(5)
C(9)	80(8)	53(6)	33(5)	3(4)	11(5)	6(5)
C(10)	91(9)	76(8)	53(6)	16(6)	19(6)	-18(7)
C(11)	64(8)	133(12)	48(7)	19(7)	18(6)	-14(8)
C(12)	50(7)	85(8)	50(6)	13(6)	10(5)	-5(6)
C(13)	112(10)	85(9)	31(5)	2(5)	5(6)	28(7)
C(14)	48(7)	144(13)	67(8)	27(8)	3(6)	8(8)
C(15)	154(17)	250(30)	93(13)	28(13)	35(12)	144(18)
C(16)	78(12)	220(20)	131(16)	39(16)	-25(11)	-32(13)
I(1)	64(1)	62(1)	34(1)	-9(1)	1(1)	-14(1)
C(17)	111(10)	64(8)	43(6)	-13(5)	7(6)	-30(7)
C(18)	71(8)	86(9)	37(5)	1(5)	-8(5)	-9(6)
C(19)	81(8)	68(7)	44(6)	6(6)	10(6)	-25(6)
C(20)	148(16)	87(11)	145(16)	50(12)	32(13)	-31(11)
C(21)	250(30)	126(17)	170(20)	84(17)	1(19)	-16(17)
O(3)	143(8)	70(6)	61(5)	19(4)	20(5)	-14(5)
C(22)	72(8)	56(7)	42(6)	5(5)	7(5)	-11(6)
C(23)	65(7)	49(6)	26(4)	2(4)	0(4)	-5(5)
C(24)	53(6)	46(6)	34(5)	-3(4)	12(4)	-11(5)
C(25)	70(7)	51(6)	34(5)	-9(4)	1(5)	-8(5)
C(26)	58(7)	57(7)	48(6)	-6(5)	-1(5)	-5(5)
C(27)	62(7)	47(6)	62(7)	1(5)	-5(5)	-10(5)
O(4)	104(6)	48(4)	31(3)	0(3)	18(4)	-21(4)
C(28)	57(6)	48(6)	32(5)	5(4)	9(4)	-10(5)
C(29)	54(6)	52(6)	59(6)	-7(5)	8(5)	-4(5)
C(30)	50(6)	66(7)	60(6)	13(6)	-5(5)	-7(5)

C(31)	90(9)	69(8)	62(7)	27(6)	-14(6)	-12(7)
C(32)	66(7)	71(8)	63(7)	31(6)	-17(6)	-28(6)
C(33)	55(6)	47(6)	43(5)	9(4)	2(5)	-2(5)
C(34)	65(8)	101(11)	125(13)	14(9)	-42(8)	1(7)
C(35)	78(8)	61(7)	34(5)	12(5)	-12(5)	-4(6)
C(36)	88(10)	137(14)	71(9)	-14(9)	-33(8)	36(9)
C(37)	117(12)	75(9)	84(9)	-1(7)	-43(9)	2(8)
I(2)	115(1)	70(1)	31(1)	-3(1)	11(1)	-17(1)
C(38)	82(9)	74(8)	71(8)	-29(7)	2(7)	7(7)
C(39)	68(8)	81(9)	72(8)	-18(7)	-16(6)	-5(7)
C(40)	107(11)	55(8)	91(9)	0(7)	-23(8)	-3(7)
C(41)	220(30)	85(15)	210(30)	60(16)	-70(20)	-35(16)
C(42)	250(40)	190(30)	330(50)	20(30)	-60(30)	70(30)

Table S13. Hydrogen coordinates (x 10^4) and isotropic displacement parameters ($Å^2$x 10 ^{3}) for JDS_1_040_A_P43.

	x	y	z	U(eq)
H(4A)	4135	4970	6268	53
H(4B)	3405	5691	5950	53
H(6)	4206	6494	5212	54
H(7)	2552	4233	6374	56
H(8A)	4114	3356	6608	58
H(8B)	3602	2252	6482	58
H(9)	2874	3602	7228	67
H(10A)	1649	2264	7395	88
H(10B)	2092	1572	6973	88
H(11A)	1008	3544	6847	98
H(11B)	514	2426	6725	98
H(12)	1849	2159	6134	74
H(13A)	3546	2404	7754	114
H(13B)	4468	2619	7389	114
H(13C)	3787	1576	7350	114
H(14)	1289	3466	5565	104
H(15A)	-71	4210	6268	247
H(15B)	-26	4605	5737	247
H(15C)	931	4829	6082	247
H(16A)	-534	2448	5945	215
H(16B)	394	1739	5747	215
H(16C)	-203	2530	5407	215
H(17A)	4438	7318	5992	109
H(17B)	5684	7244	6013	109
H(17C)	4992	6698	6410	109

H(18A)	6075	5104	6223	97
H(18B)	6617	5603	5771	97
H(18C)	5971	4540	5726	97
H(19A)	6132	7149	5281	78
H(19B)	6255	6328	4863	78
H(20)	5015	7232	4432	152
H(21A)	5545	8917	4904	218
H(21B)	4936	8793	4400	218
H(25A)	918	9166	4311	62
H(25B)	-106	9709	4104	62
H(27)	106	11254	3566	69
H(28)	-186	7763	4232	55
H(29A)	1580	7593	4451	66
H(29B)	1739	6618	4108	66
H(30)	451	6585	4940	70
H(31A)	821	4896	4301	88
H(31B)	136	4803	4768	88
H(32A)	-1093	5991	4465	80
H(32B)	-971	5021	4118	80
H(33)	114	6009	3620	59
H(34A)	1712	5420	5256	146
H(34B)	2242	6517	5132	146
H(34C)	2404	5537	4793	146
H(35)	-1121	7307	3332	69
H(36A)	-2806	7403	3653	148
H(36B)	-1956	7799	4022	148
H(36C)	-2457	6661	4075	148
H(37A)	-2028	5281	3433	138
H(37B)	-1161	5563	3050	138
H(37C)	-2289	6096	3025	138
H(38A)	1039	10857	4747	114
H(38B)	156	11437	4449	114
H(38C)	1304	11923	4480	114
H(39A)	2597	10114	4315	111
H(39B)	2749	11148	4009	111
H(39C)	2594	10046	3756	111
H(40A)	1345	12659	3798	101
H(40B)	2021	12286	3359	101
H(41)	1090	13108	2911	206
H(42A)	-654	13388	3322	310
H(42B)	-183	13785	2818	310

Table S14. Torsion angles [°] for JDS_1_040_A_P43.

O(1)-C(1)-C(2)-C(3)	177.7(9)	C(5)-C(6)-C(19)-C(20)	-139.3(12)
C(6)-C(1)-C(2)-C(3)	-6.2(13)	C(6)-C(19)-C(20)-C(21)	115(3)
O(1)-C(1)-C(2)-I(1)	-6.0(12)	O(3)-C(22)-C(23)-C(24)	176.0(11)
C(6)-C(1)-C(2)-I(1)	170.2(6)	C(27)-C(22)-C(23)-C(24)	-6.3(16)
C(1)-C(2)-C(3)-O(2)	175.6(8)	O(3)-C(22)-C(23)-I(2)	-1.3(15)
I(1)-C(2)-C(3)-O(2)	-0.7(11)	C(27)-C(22)-C(23)-I(2)	176.5(8)
C(1)-C(2)-C(3)-C(4)	-2.8(14)	C(22)-C(23)-C(24)-O(4)	-179.6(10)
I(1)-C(2)-C(3)-C(4)	-179.1(6)	I(2)-C(23)-C(24)-O(4)	-2.4(13)
O(2)-C(3)-C(4)-C(5)	161.1(8)	C(22)-C(23)-C(24)-C(25)	-1.2(16)
C(2)-C(3)-C(4)-C(5)	-20.5(12)	I(2)-C(23)-C(24)-C(25)	176.0(7)
C(3)-C(4)-C(5)-C(17)	170.3(8)	O(4)-C(24)-C(25)-C(26)	155.2(9)
C(3)-C(4)-C(5)-C(18)	-72.2(10)	C(23)-C(24)-C(25)-C(26)	-23.1(15)
C(3)-C(4)-C(5)-C(6)	49.9(11)	C(24)-C(25)-C(26)-C(39)	-69.5(12)
O(1)-C(1)-C(6)-C(19)	-17.9(12)	C(24)-C(25)-C(26)-C(38)	171.1(9)
C(2)-C(1)-C(6)-C(19)	166.0(9)	C(24)-C(25)-C(26)-C(27)	50.4(12)
O(1)-C(1)-C(6)-C(5)	-146.8(9)	O(3)-C(22)-C(27)-C(40)	-15.9(16)
C(2)-C(1)-C(6)-C(5)	37.0(12)	C(23)-C(22)-C(27)-C(40)	166.4(10)
C(17)-C(5)-C(6)-C(1)	-174.8(9)	O(3)-C(22)-C(27)-C(26)	-145.8(11)
C(18)-C(5)-C(6)-C(1)	64.0(10)	C(23)-C(22)-C(27)-C(26)	36.5(14)
C(4)-C(5)-C(6)-C(1)	-56.9(10)	C(39)-C(26)-C(27)-C(22)	63.4(12)
C(17)-C(5)-C(6)-C(19)	58.8(12)	C(25)-C(26)-C(27)-C(22)	-56.7(11)
C(18)-C(5)-C(6)-C(19)	-62.4(11)	C(38)-C(26)-C(27)-C(22)	-173.8(9)
C(4)-C(5)-C(6)-C(19)	176.7(9)	C(39)-C(26)-C(27)-C(40)	-65.6(13)
C(2)-C(3)-O(2)-C(7)	-178.4(8)	C(25)-C(26)-C(27)-C(40)	174.3(10)
C(4)-C(3)-O(2)-C(7)	0.0(13)	C(38)-C(26)-C(27)-C(40)	57.1(14)
C(3)-O(2)-C(7)-C(8)	-90.2(10)	C(23)-C(24)-O(4)-C(28)	-172.7(9)
C(3)-O(2)-C(7)-C(12)	150.7(8)	C(25)-C(24)-O(4)-C(28)	8.9(15)
O(2)-C(7)-C(8)-C(9)	-177.1(8)	C(24)-O(4)-C(28)-C(33)	145.5(9)
C(12)-C(7)-C(8)-C(9)	-61.5(11)	C(24)-O(4)-C(28)-C(29)	-93.0(11)
C(7)-C(8)-C(9)-C(13)	178.9(9)	O(4)-C(28)-C(29)-C(30)	-171.4(8)
C(7)-C(8)-C(9)-C(10)	57.3(11)	C(33)-C(28)-C(29)-C(30)	-55.6(11)
C(13)-C(9)-C(10)-C(11)	-177.9(9)	C(28)-C(29)-C(30)-C(31)	55.8(12)
C(8)-C(9)-C(10)-C(11)	-56.5(12)	C(28)-C(29)-C(30)-C(34)	-179.6(10)
C(9)-C(10)-C(11)-C(12)	58.8(14)	C(34)-C(30)-C(31)-C(32)	179.8(10)
O(2)-C(7)-C(12)-C(14)	-56.6(12)	C(29)-C(30)-C(31)-C(32)	-57.5(12)
C(8)-C(7)-C(12)-C(14)	-174.1(9)	C(30)-C(31)-C(32)-C(33)	59.2(14)
O(2)-C(7)-C(12)-C(11)	177.7(9)	O(4)-C(28)-C(33)-C(32)	170.5(8)
C(8)-C(7)-C(12)-C(11)	60.3(12)	C(29)-C(28)-C(33)-C(32)	52.6(11)
C(10)-C(11)-C(12)-C(7)	-58.2(14)	O(4)-C(28)-C(33)-C(35)	-62.1(11)
C(10)-C(11)-C(12)-C(14)	174.7(11)	C(29)-C(28)-C(33)-C(35)	-180.0(8)
C(7)-C(12)-C(14)-C(15)	-71.4(16)	C(31)-C(32)-C(33)-C(28)	-54.4(13)
C(11)-C(12)-C(14)-C(15)	51.6(17)	C(31)-C(32)-C(33)-C(35)	177.9(10)
C(7)-C(12)-C(14)-C(16)	160.8(11)	C(28)-C(33)-C(35)-C(36)	-71.0(13)
C(11)-C(12)-C(14)-C(16)	-76.2(15)	C(32)-C(33)-C(35)-C(36)	53.7(14)
C(1)-C(6)-C(19)-C(20)	94.8(13)	C(28)-C(33)-C(35)-C(37)	162.1(10)

C(32)-C(33)-C(35)-C(37)	-73.3(13)	C(26)-C(27)-C(40)-C(41)	-155.0(17)
C(22)-C(27)-C(40)-C(41)	76.8(19)	C(27)-C(40)-C(41)-C(42)	67(6)

Symmetry transformations used to generate equivalent atoms:

7. References

1. L. Hadjiarapoglou, A. de Meijere, H.-J. Seitz, I. Klein, D. Spitzner, *Tetrahedron Lett.* **1994**, *35*, 3269-3272.
2. G. Stork, R. L. Danheiser, *J. Org. Chem.* **1973**, *38*, 1775-1776.
3. G. B. Dudley, K. S. Takaki, D. D. Cha, R. L. Danheiser, *Org. Lett.* **2000**, *2*, 3407-3410.
4. M. O. Ganiu, A. H. Cleveland, J. L. Paul, R. Kartika, *Org. Lett.* **2019**, *21*, 5611-5615.
5. T. B. Poulsen, L. Bernardi, *J. Am. Chem. Soc.* **2007**, *129*, 441-449.
6. V. A. Vasin, V. V. Razin, *Synlett* **2001**, 658-660.
7. D. K. Rayabarapu, J. A. Tunge, *J. Am. Chem. Soc*. **2005**, *127*, 13510-13511.
8. K. Kato, A. Nishimura, Y. Yamamoto, H. Akita, *Tetrahedron Lett.* **2001**, *42*, 4203-4205.
9. S. E. Denmark, R. A. Stavenger, A.-M. Faucher, J. P. Edwards, *J. Org. Chem.* **1997**, *62*, 3375-3389.
10. L. Miao, I. Haque, M. R. Manzoni, W. S. Tham, S. R. Chemler, *Org. Lett.* **2010**, *12*, 4739-4741.
11. N. Biber, K. Möws, B. Plietker, *Nat. Chem*. **2011**, *3*, 938-942.
12. H. F. Sneddon, A. van den Heuvel, A. Hirsch, R. A. Booth, D. M. Shaw, M. J. Gaunt, S. V. Ley, *J. Org. Chem.* **2006**, *71*, 2715-2725.
13. K. Yamada, Y. Igarashi, T. Betsuyaku, M. Kitamura, K. Hirata, K. Hioki, M. Kunishima, *Org. Lett.* **2018**, *20*, 2015-2019.
14. V. K. Aggarwal, Z. Gültekin, R. S. Grainger, H. Adams, P. L. Spargo, *J. Chem. Soc. Perkin Trans. 1* **1998**, 2771–2782.
15. A. Horn, U. Kazmaier, *Eur. J. Org. Chem.* **2018**, 2531-2536.
